# Late-Stage Aromatic C–H Bond Functionalization for Cysteine/Selenocysteine Bioconjugation

**DOI:** 10.1021/jacs.5c08936

**Published:** 2025-08-19

**Authors:** Zhenguang Zhao, Jian Huang, Yao Cai, Tai-Ping Zhou, Fatina Khatib, Daphna Shimon, Binju Wang, Norman Metanis

**Affiliations:** † Institute of Chemistry, 98519 The Hebrew University of Jerusalem, Jerusalem 9190401, Israel; ‡ Department of Chemistry and Chemical Biology, Harvard University, Cambridge, Massachusetts 02138, United States; § School of Chemistry and Chemical Engineering, Jinggangshan University, Ji’an, Jiangxi 343009, China; ∥ State Key Laboratory of Physical Chemistry of Solid Surfaces and Fujian Provincial Key Laboratory of Theoretical and Computational Chemistry, College of Chemistry and Chemical Engineering, 12466 Xiamen University, Xiamen 361005, China; ⊥ The Center for Nanoscience and Nanotechnology, The Hebrew University of Jerusalem, Jerusalem 9190401, Israel; # Casali Center for Applied Chemistry, The Hebrew University of Jerusalem, Jerusalem 9190401, Israel

## Abstract

Bioconjugation of peptides and proteins has become an indispensable tool in fundamental biological research and drug development. Herein, we report a copper-mediated efficient cysteine/selenocysteine-specific bioconjugation through direct C–H functionalization of electron-rich arenes under biocompatible reaction conditions. In this method, a series of commercial electron-rich arenes, including natural products and drug molecules, are conjugated to cysteine/selenocysteine-containing peptides and proteins. Furthermore, we show that this new bioconjugation method allows the efficient stapling of peptides, as well as the cross-linking of different peptides to a single arene, all in high yields. The tunable electron density of small molecules enables the selective modification of selenocysteine in the presence of cysteine residues. Finally, mechanistic studies suggest that the conjugation proceeds via a proton-coupled electron transfer (PCET) process and substrate radical binding to the copper for C–Se/S bond formation. This approach provides an efficient strategy for the late-stage functionalization of complex small molecules to generate peptide/protein conjugates.

## Graphical abstract

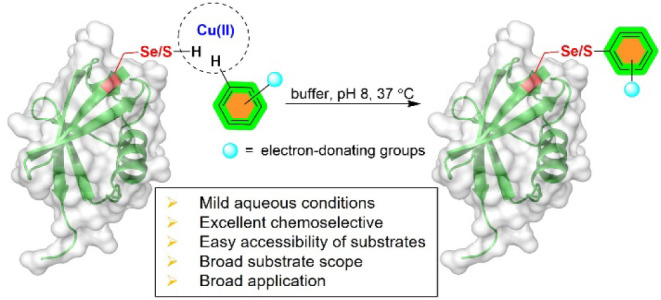

## Introduction

Peptide and protein bioconjugation has significantly advanced our understanding of biological processes,^1^ such as the actions of complex biomolecules,^2^ the development of therapeutics e.g., antibody-drug conjugates (ADCs),^3^ and the construction of new functional materials.^4^ However, achieving efficient and chemoselective functionalization of peptides and proteins remains challenging due to the presence of diverse reactive functional groups—including thiols, acids, alcohols, and amines—especially under mild biocompatible conditions.^5^ To address these challenges, chemists have developed chemo- and regioselective reactions for modifying naturally occurring amino acids in peptides and proteins.^5a^ Cysteine (Cys, C) is frequently targeted^6^ due to its high intrinsic reactivity^7^ and relatively low abundance in proteins.^8^ Similarly, selenocysteine (Sec, U), the 21st encoded amino acid, presents an attractive alternative for chemoselective functionalization^9,10^ to Cys and its even lower abundance.^11^ Nevertheless, many existing strategies require the preinstallation of reactive functional groups on the target amino acids (Figure 1a)^12^ or payload reagents (Figure 1b,c)^13^ if not commercially available.

**Figure 1:**
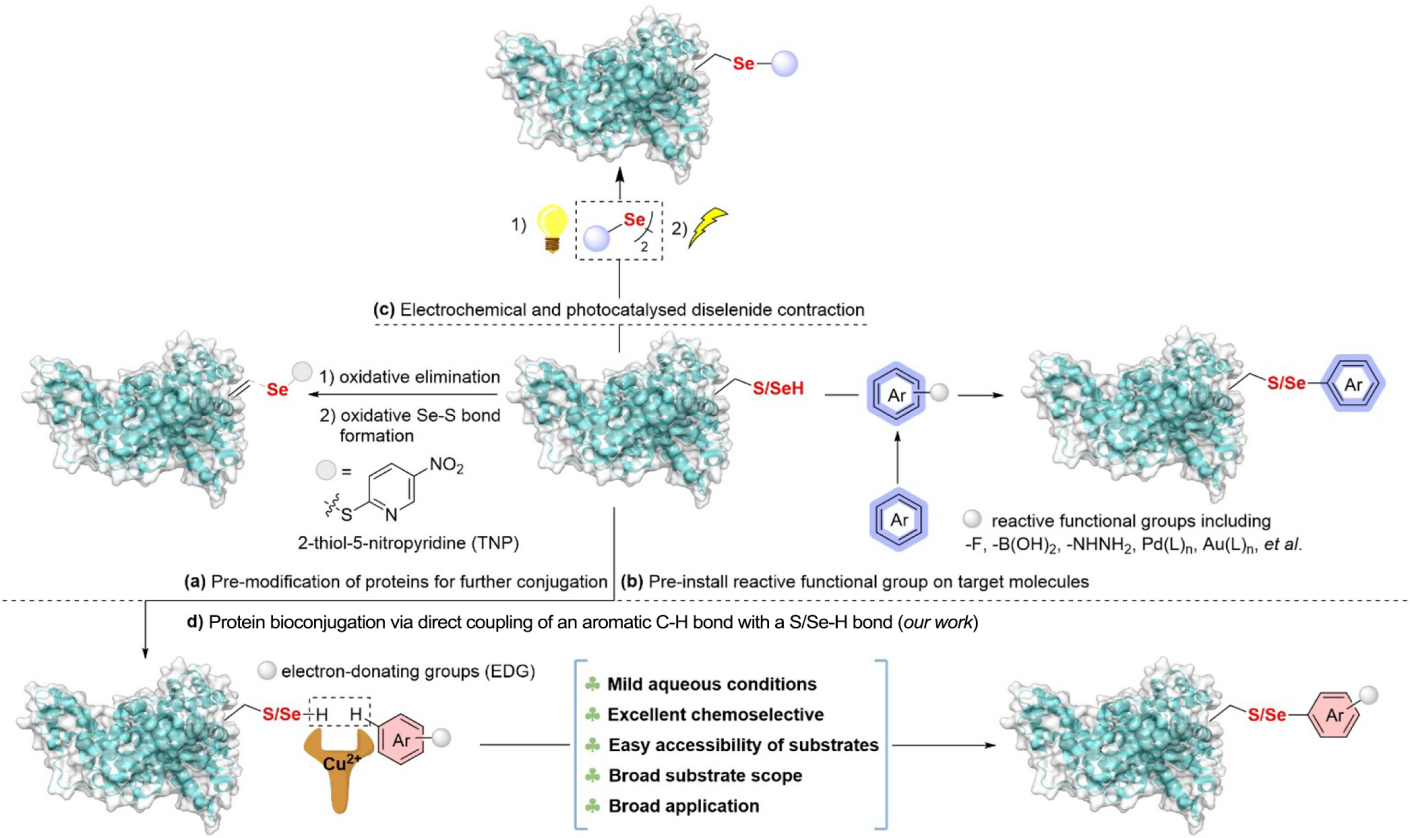
Representative strategies of Sec/Cys-specific bioconjugation. (a) Premodification of Cys/Sec to create a reactive site in target proteins. (b) An additional step to install a reactive functional group on target molecules for Cys/Sec bioconjugation. (c) Electrochemical and photocatalyzed diselenide contraction for Sec modification. (d) Direct aromatic C–H bond functionalization with free Cys/Sec for protein diversification.

The direct coupling of an aromatic C–H bond in a complex molecule with the S/Se–H bond of Cys/Sec represents a straightforward and ideal approach for protein bioconjugation. Direct C–H bond thiolation of arenes with thiols^14^ and selenylation with diaryl selenides^15^ has been demonstrated. Unfortunately, these techniques are typically performed between small molecules and under harsh conditions, which are incompatible for proteins. A more recent method involves the direct replacement of an aromatic C–H bond in electron-rich arenes with the electrophilic Sec, enabling the chemoselective modification of peptides and proteins (Figure 1a).^12a^ However, to induce the electrophilic character in Sec, it must be prefunctionalized with 2-thiol-5-nitropyridine (TNP), which carries the risk of disrupting other Cys residues in the protein.^12a^

We previously reported the chemoselective modification of Sec in peptides and proteins using various hydrazine compounds (Figure 1b).^16^ This efficient transformation was enabled by copper-mediated *in situ* generation of alkyl/aryl radicals from hydrazine substrates. Despite the broad scope and biocompatible conditions of this approach, it required the presence of a hydrazine moiety to facilitate the radical intermediate formation, followed by coupling with Sec. However, the need to preinstalled hydrazine on target molecules may reduce the overall efficiency of protein modification. Additionally, further modification of hydrazine molecules is challenging due to their sensitivity to oxidation. Encouragingly, conjugating *N*-heterocycles to Sec in oligopeptides has been achieved via a photocatalytic reaction, although it was implemented in acetonitrile.^17^ A direct coupling of a C–H bond in target molecules with a S/Se–H bond in Cys/Sec under mild conditions is highly desired (Figure 1d), which would provide an efficient solution for constructing peptide/protein-natural products or drug conjugates, eliminating the need for substrate prefunctionalization.

Here we report an efficient chemoselective modification of peptides and proteins through Cys/Sec-bioconjugation with a series of electron-donating arenes (Figure 1d). This method leverages a copper-mediated coupling reaction between an aromatic C–H bond and the S/Se–H bond under biocompatible conditions, enabling the bioconjugation of peptides and proteins.

## Results and Discussion

In our previous efforts to study chemoselective radical-mediated arylation of Sec,^16^ we explored the use of electron-rich resorcinol (**2a**) as an alternative for the hydrazine substrates to generate the appropriate aromatic radical for Sec bioconjugation. Although resorcinol remained inert toward the Sec-containing peptide (TF **U** GK) in a neutral aqueous buffer, even after a prolonged reaction time, intriguingly, we observed a moderate conjugation of resorcinol to Sec in the presence of CuSO_4_ at pH 7 (Figures S1 and S2). To systematically optimize Sec bioconjugation with resorcinol, we prepared a Sec-containing model peptide, LG **U** ALG-NH_2_ (**1**, isolated as a diselenide dimer under atmospheric conditions due to the lower p*K*_a_ and redox potential of Sec),^9,10^ to better assess the regioselectivity in its conjugation with resorcinol. We tested various metal ion additives for the conjugation of **1** (1 mM) and **2a** (5 mM) at 37 °C in phosphate buffer (PB) (Figure S5), and an appreciable 93% conjugation was achieved using Cu­(OTf)_2_ (1 mM) in only 15 min (Figure 2a, entry 1). After characterization by LC-MS, 1D and 2D NMR experiments, including H–H correlation spectroscopy (COSY, Figure 2b) and heteronuclear single quantum correlation (HSQC) (Figures 2b and S110–S114), it was indicated that the C–H bond at C-4 of resorcinol was substituted by a C–Se bond in the primary monosubstituted conjugate **3a**_**1**_. Additionally, a disubstituted conjugate **3a**_**2**_ was produced in 6% conversion (Figure S10). A pH screening study revealed the Sec conjugation with resorcinol proceeded sluggishly under acidic conditions (Figure S3). When excess peptide **1** reacted with **2a**, disubstituted conjugates predominated, and a trisubstituted conjugate **3a**_**3**_ was also observed (Figure S7), suggesting the potential for peptide stapling, dimerization, and cross-linking different peptides and proteins (see below). Given the challenges associated with incorporating Sec into proteins,^18^ we expected that a Cys bioconjugation might also be feasible using the same strategy. To this end, Sec was replaced with Cys in the model peptide **4** (LG **C** ALG-NH_2_), and its reaction with **2a** (3 mM) under optimized conditions resulted in the formation of a single monosubstituted conjugate **5a** with 89% conversion within 1 h (Figures 2c and S24).

**Figure 2:**
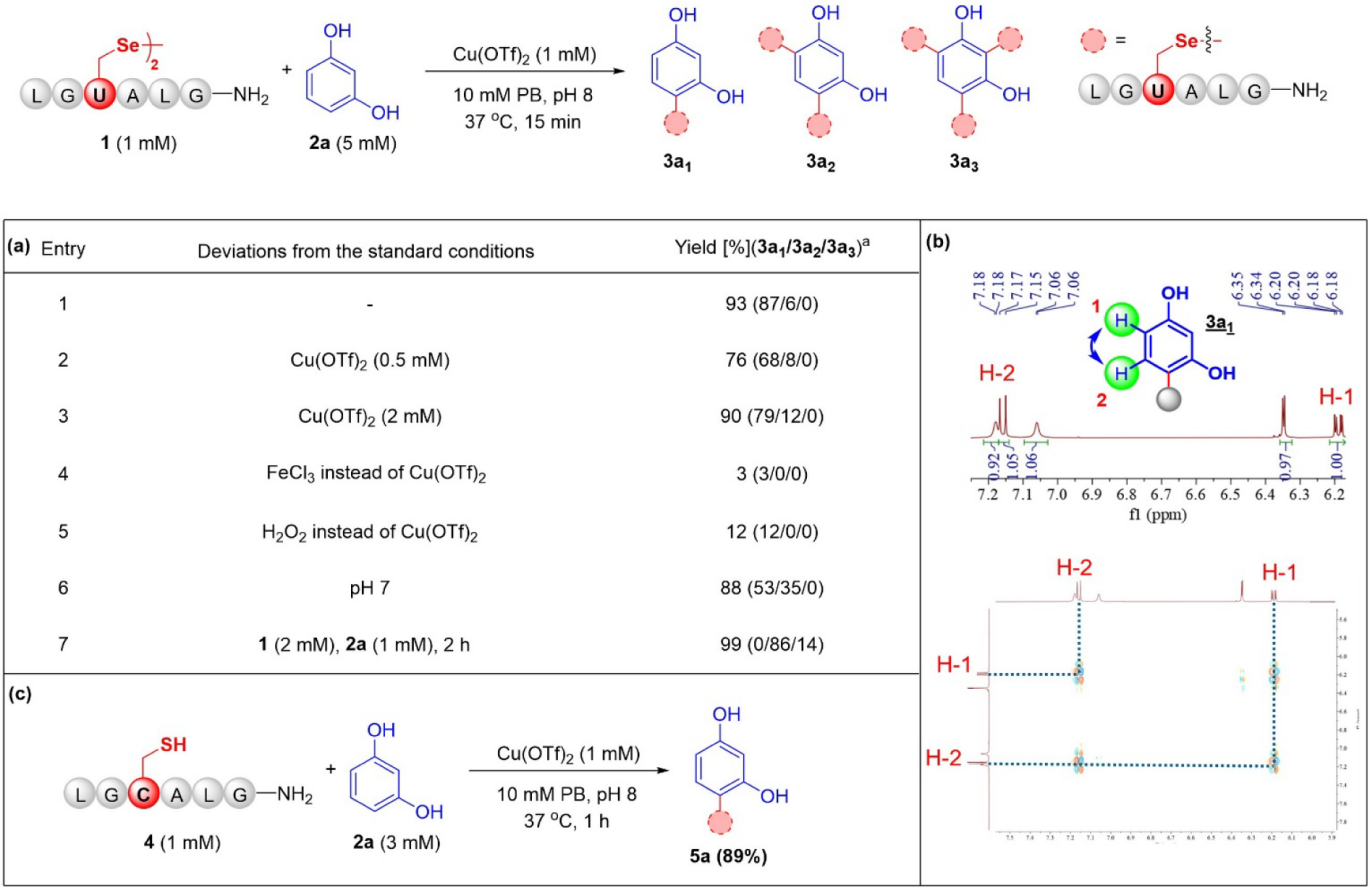
Development of direct aromatic C–H bond functionalization with Cys/Sec for peptide diversification. (a) Optimization of reaction conditions with Sec-containing peptide. (b) The characterization of conjugate **3a**_**1**_ by NMR. Further details can be found in the Supporting Information. ^a^Yields were calculated based on the integrated areas of HPLC peaks (at 220 nm). (c) The optimal reaction system is transferable to Cys-containing peptide functionalization.

To establish a general method for Cys/Sec-bioconjugation via aromatic C–H functionalization, we evaluated the scope of electron-rich arenes using peptides **1** (LG **U** ALG-NH_2_) and **4** (LG **C** ALG-NH_2_) in parallel. As shown in Figure 3, a series of resorcinol derivatives (**3b**–**3f**, Figures S11–S15 and S25–S29) demonstrated broad compatibility in modifying both Cys and Sec residues. For Sec, occasional disubstituted conjugates were observed, while Cys yielded exclusively monosubstituted conjugates. Phenol (**2m**) and 1,3,5-trimethoxybenzene (**2n**) were found to be inert to both Cys and Sec under the optimized conditions (Figures S22 and S23). Strong electron-donating groups (**2g**–**2i**) enhanced the reactivity to both Cys/Sec (Figures S16–S18 and S30–S32). However, *m*-cresol (**2j**) and 3,5-dimethylphenol (**2k**) were ineffective for Cys conjugation (Figures S33 and S34). Additionally, *m*-phenylenediamine (**2l**), a diamino analogue of resorcinol (**2a**), exhibited no reactivity toward Cys under the same conditions (Figure S35).

**Figure 3:**
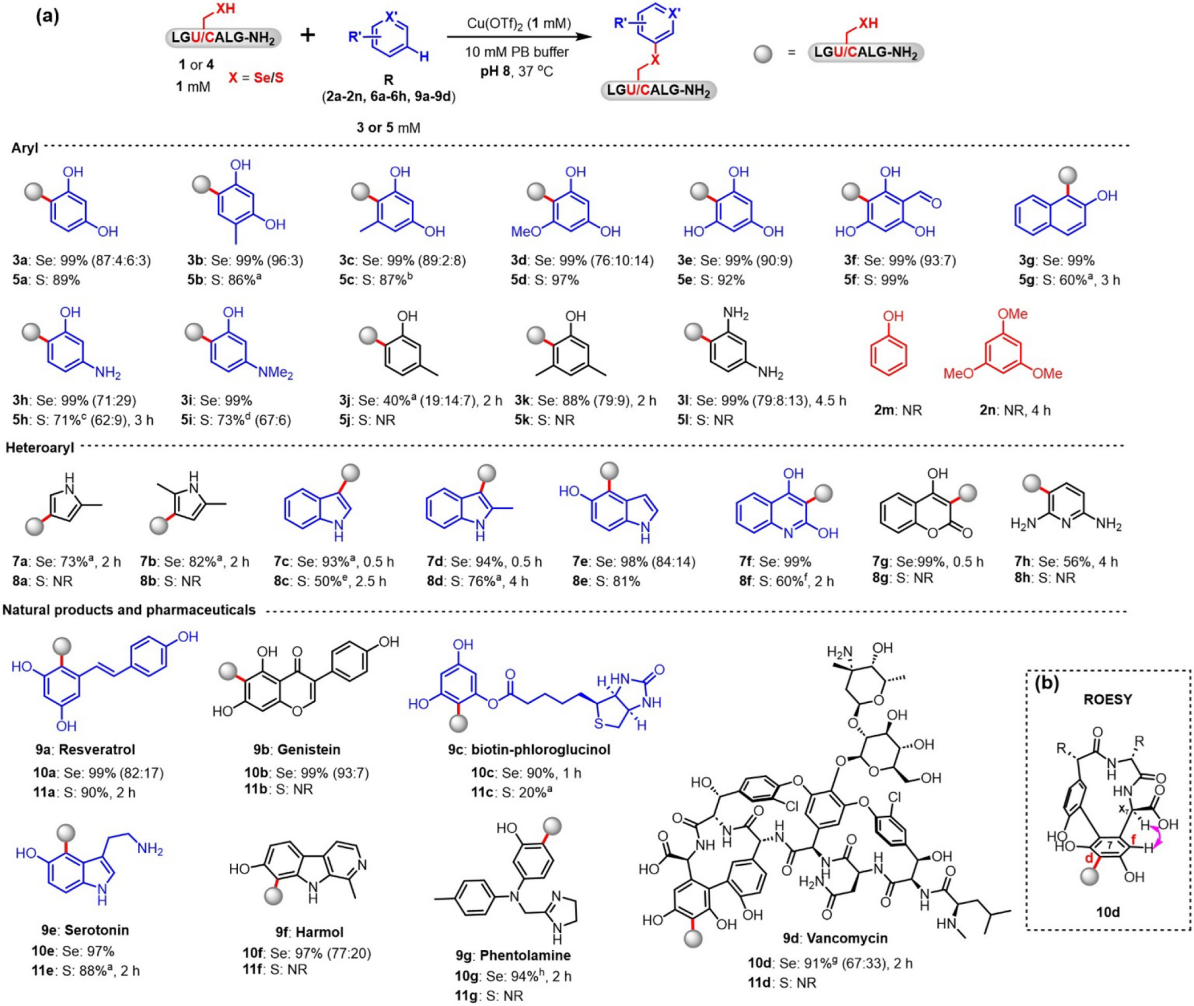
(a) Sec/Cys-specific conjugation with a series of small molecules. The conversions shown in parentheses were calculated based on the integrated areas of HPLC peaks. Each conjugate is shown with a *red* bond highlighting the new linkage formed in the reaction and a *gray* ball as the Sec or Cys peptide. Standard conditions for Sec-peptide **1**: 1 mM peptide **1** with respect to the selenol monomer, 1 equiv copper, and 5 equiv small molecule, 15 min; standard conditions for Cys-peptide **4**: 1 mM peptide **4**, 1 equiv copper, and 3 equiv small molecule, 1 h. ^a^1 equiv bipyridine was used. ^b^0.5 equiv copper and bipyridine were used. ^c^1 equiv bipyridine was used, and the reaction was conducted in a degassed solution. ^d^3 equiv copper, bipyridine, and 10 equiv small molecule were used. ^e^1 equiv copper, bipyridine, and 10 equiv small molecule were used, and the reaction was conducted in a degassed solution. ^f^5 equiv copper, bipyridine, and 10 equiv **6f** were used, and the reaction was conducted in a degassed solution. ^g^1 equiv copper and bipyridine, and 1.5 equiv **9d** were used. ^h^1 mM peptide **1**, 1 equiv copper and bipyridine, and 1.5 equiv **9g** were used. (b) ROESY for vancomycin in the peptide–vancomycin conjugate **10d**.

We also explored the compatibility of the modification reaction with various heteroaromatic compounds with different scaffolds, including pyrrole (**6a**-**b**), indole (**6c**-**e**), quinolinediol (**6f**), coumarin (**6g**) and pyridine (**6h**), and found the reaction to be with wide scope of substrates (Figures 3 and S36–S50).

Intriguingly, all the products were singly monosubstituted conjugates, with the exception of **7e**. These findings demonstrate that the reactivity of electron-rich arenes toward Cys and Sec can be tuned by incorporating different electron-donating groups, enabling selective modification of Sec over Cys in peptides, or even sequential modifications of these residues.

Peptide-drug conjugates (PDCs) have emerged as a promising direction for cancer therapy^19^ due to their small molecular weight and low immunogenicity.^20^ A critical aspect of PDC development is the selective conjugation of drug molecules to a targeted peptide. To this end, we investigated the potential of aromatic C–H functionalization of Cys/Sec residues to facilitate the construction of PDCs with complex functional molecules. Natural products like resveratrol (**9a**),^21^ genistein (**9b**),^22^ serotonin (**9e**)^23^ and β-carboline harmol (**9f**), and drug molecules like vancomycin (**9d**)^24^ and phentolamine (**9g**),^25^ were all readily linked to peptide **1** via Sec conjugation (Figures S52–S53 and S55–S58), and minimal disubstitutions were observed in the resulting conjugates **(10a–10b**, **10f** and **10d)**. Additionally, the conjugation of biotin (**9c**) to peptide **1**, achieved through coupling with phloroglucinol and Sec, demonstrated the feasibility of Sec functionalization for nonaromatic molecules (Figure S54). Meanwhile, resveratrol (**9a**) and serotonin (**9e**) were also smoothly conjugated to Cys residues with excellent yields (Figures S59 and S63). These findings highlight the potential of Cys/Sec-based aromatic C–H functionalization as a versatile strategy for generating PDCs with diverse functional molecules.

To assess the applicability of this conjugation approach to complex target molecules, we aimed to evaluate the chemoselectivity and the impact of the chemical environment on conjugation reactivity. First, two negative controls of Sec/Cys-free peptides, **12** and **13**, while containing aromatic residues Trp and Tyr, were found to remain inert toward **2e** under the standard conditions (Figures S66 and S67), even after extended reaction time. Two additional peptides containing Sec (**14**) or Cys (**15**), respectively, were reacted with **2a** or **2e**. Both reactions yielded single monosubstituted conjugates with good conversion, despite the presence of other reactive amino acids in their sequences (Figures 4a, S68, and S69). MS/MS analysis of **15e** confirmed conjugation at the Cys residue (Figure 4a). Given the complex chemical environment in most biomolecules, we evaluated its impact on our conjugation efficiency. As shown in Figure 4b, disubstituted products (two peptides linked to one small molecule) were observed in both Sec- and Cys-conjugations when Sec/Cys was located at the N-terminus or adjacent to amino acids with small steric hindrance. In contrast, only single monosubstituted products were obtained when Sec/Cys in the middle sequence was buried by amino acids with larger steric hindrance. These data demonstrate a significant impact of the chemical environment on conjugation reactivity, providing insights into the design of controllable conjugate production.

**Figure 4:**
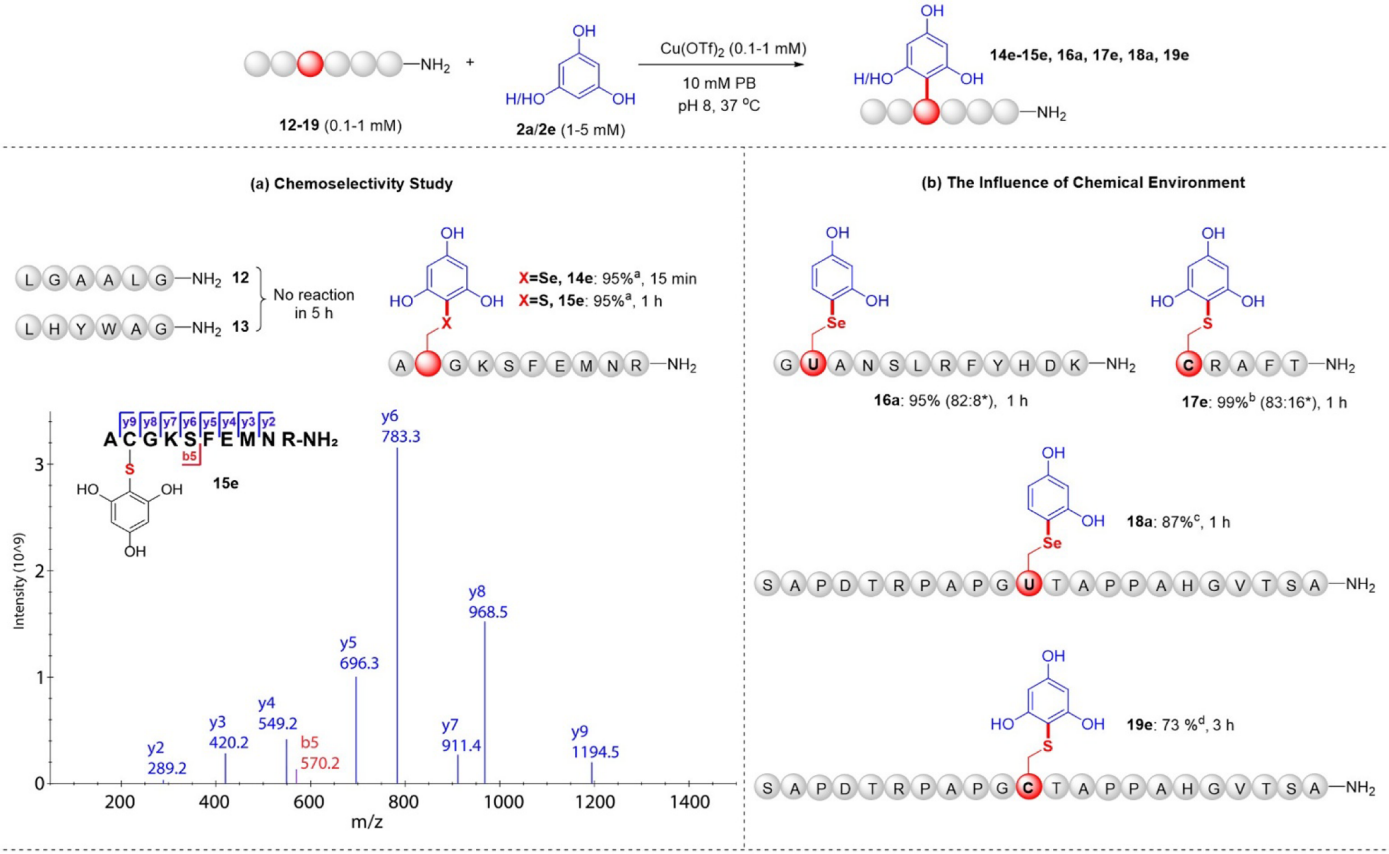
Sec/Cys-specific modification in the presence of other reactive residues. (a) Chemoselectivity study with MS/MS spectra of **15e**. ^a^Peptide (0.1 mM), **2e** (1 mM), and Cu­(OTf)_2_ (2 equiv) were used. (b) Evaluate the influence of chemical environment on conjugation reactivity. ^b^Cu­(OTf)_2_ (0.3 equiv) was used. ^c^Cu­(OTf)_2_ (8 equiv) was used. ^d^Peptide (0.1 mM), **2e** (1 mM), and Cu­(OTf)_2_ (3 equiv) were used. *Refers to disubstituted conjugates: two peptides conjugated with the identical molecule. The conversions were calculated based on the integrated areas of HPLC peaks.

Peptide cyclization is a useful strategy for stabilizing short and flexible peptides into well-defined bioactive stapled peptides, which can have improved biological properties, including increased resistance to proteolytic degradation, enhanced cell permeability and higher-affinity binding toward its intended biological target.^26^ To evaluate the applicability of our method for constructing stapled peptides, we used phloroglucinol **2e** as the cross-linking agent to staple Sec and/or Cys at various positions within the peptides with the following sequences: G **U** ANKHTWYL **U** A-NH_2_ (**20**), G **U** ALNKFQEKSRMKYRWKHR **C** G-NH_2_ (**21**), and G **C** ANKHTWYL **C** A-NH_2_ (**22**). As shown in Figure 5a, the cyclization of peptides **20** and **22** was achieved through the stapling of Sec-Sec (Figure S75) and Cys-Cys (Figure S79), respectively, with the yield of 90% and 89%, although 25% oxidation (+32 Da) in the case of stapled peptide **22e** was observed. Staple Sec-Cys in peptide **21** was also successfully constructed with 94% conversion (Figure S77). To confirm the stapled residues in the peptides, stapled peptides **20e**, **21e**, and **22e** were digested by trypsin and subjected to HPLC-ESI MS analysis (Figures S76, S78, and S80). The analysis indicated conjugation exclusively at the Sec/Cys over the rest of the amino acids.

**Figure 5:**
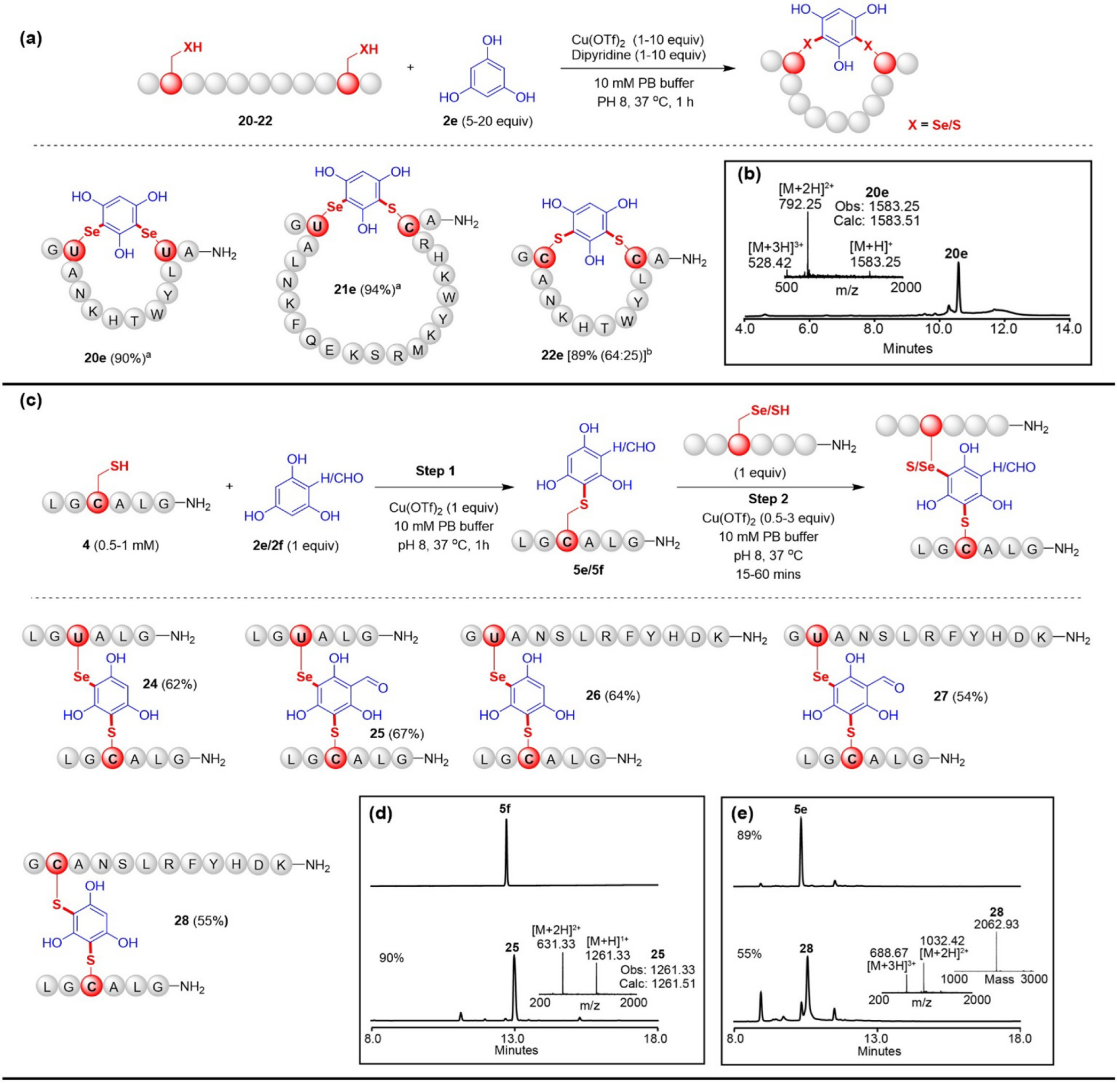
(a) Peptides stapling with Sec/Cys conjugation. The conversions shown in parentheses were calculated based on the integrated areas of HPLC peaks. ^a^Reaction conditions: 0.5 mM peptide, 1 equiv Cu­(OTf)_2_ and 5 equiv **2e** were used. ^b^Reaction condition: 0.1 mM peptide, 10 equiv Cu­(OTf)_2_ and bipyridine, and 20 equiv **2e** were used, and the reaction was conducted in a degassed solution, 25% is attributed to the oxidized conjugates (+32 Da). (b) Representative crude HPLC and ESI-MS spectrum for the stapled peptide **20e**. (c) One-pot stepwise cross-linking of different peptides. (d) Representative HPLC and ESI-MS spectrum of cross-linked conjugates **25** prepared using purified **5f**. (e) Representative HPLC and ESI-MS spectrum of the cross-linked conjugates **28** in one pot.

In addition, ICP-MS analysis showed that less than 1 ppm copper remained in purified peptide conjugates (Table S6).

The reaction of phloroglucinol **2e** with an equimolar mixture of peptides **1** and **4** yielded a single Sec-conjugate **3e**, with no Cys-conjugation observed. The distinct reactivity of Sec and Cys toward electron-rich arenes suggests that cross-linking different peptides can be achieved through a stepwise conjugation of “Se-aryl-S” in one pot. We initially performed the reaction of Cys-containing peptide **4** with phloroglucinol **2e**, and LC-MS indicated quantitative conversion within 1 h. Subsequently, an additional Sec/Cys-containing peptide and fresh copper were added to complete the cross-linking reaction in one pot (Figure 5c). Using this strategy, we produced a series of cross-linked peptides (**24**–**28**) in good yields (Figures 5c–e and S81–S85). Notably, purifying the conjugates produced in the initial step and then using these purified conjugates for the subsequent cross-linking reaction significantly enhances the conversion of the final cross-linked products compared to performing the cross-linking reaction in one pot. For example, up to 90% conversion could be achieved in the production of cross-linked products **25** using peptide **1** and purified conjugate **5f** (Figure 5d), compared to 67% conversion observed when conducted in a one-pot manner without an intermediate purification step (Figure S82).

To further examine our approach’s robustness, we turned our attention to functional modification of proteins (Figure 6). To achieve this goal, the protein ubiquitin(2–76)­(Q2U) (**29**) and ubiquitin(2–76)­(Q2C) (**30**) were prepared by chemical protein synthesis, including Fmoc-SPPS,^27^ native chemical ligation (NCL)^28^ Fmoc-protected selenazolidine,^29^ and deselenization^30^ processes (Figures S89 and S90). Also, the Sec-containing (**37**) and the Cys-containing (**38**) variants of the trihelical affibody protein Z_HER2_ were synthesized using Fmoc-SPPS. In addition to these, we utilized protein expression to obtain ubiquitin(1–76)­(M1S, Q2C) (**34**) and wildtype (WT) ubiquitin(1–76)­(M1S) (**35**) (Figures S93 and S94). The treatment of ubiquitin(2–76)­(Q2U) **29** with 20 equiv of serotonin **9e**, Cu­(OTf)_2_, and bipyridine for 3 h yielded the conjugated product **31** with a 93% yield (Figure 6). The remaining 7% was attributed to the deselenization of **29**, likely due to the extended reaction time in a basic solution (Figure S95). Using Vancomycin **9d**, a potent pharmaceutical molecule, as a labeling reagent for **29**, a 79% conversion was achieved within 10 h (Figures 6 and S96). Subsequently, the functionalization of ubiquitin(2–76)­(Q2C) **30** with **9e** resulted in the conjugate **33** with a 50% conversion within 3 h (Figures 6 and S97), which was validated by the 50% conjugation of expressed ubiquitin(1–76)­(M1S, Q2C) **34** with **9e** (Figure S98). WT ubiquitin(1–76)­(M1S) **35**, used as a negative control, remained inert toward **9e** under the same conditions (Figure S99). These results demonstrate the excellent chemoselectivity of our developed conjugation chemistry for Sec and Cys. Furthermore, Z_HER2_, a 58-amino-acid affibody molecule, exhibits selective binding and high affinity to HER2,^31^ consequently, its bioconjugates hold promising applications in diagnosing and treating various types of cancers.^32^ Hence, Z_HER2_ containing a C-terminal Sec (**37**) or Cys (**38**) was used as alternative protein model to further exemplify the biocompatibility of our approach. **9e** was successfully installed on the proteins **37** and **38** with 80% (**39**) and 70% (**41**) conversion (Figures 6, S100, and S102), respectively. While 40% conjugation of **37** with **9d** (Figures 6 and S101), the reaction of **37** with **9d** was significantly more sluggish.

**Figure 6:**
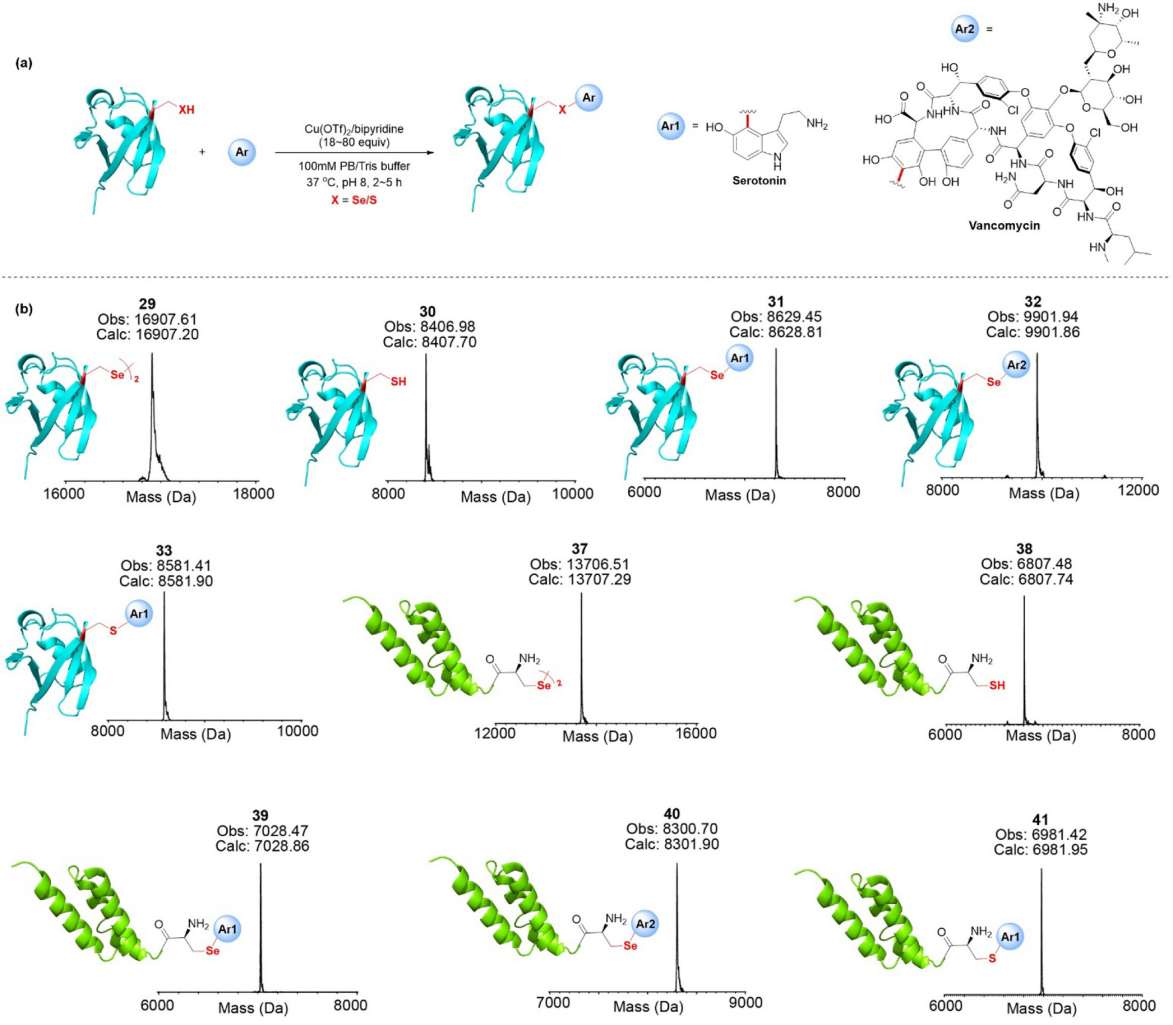
Protein modification. (a) General scheme for protein modifications. (b) ESI-MS spectra for both unmodified and modified proteins.

To obtain insight into the mechanism of copper-mediated Sec/Cys-specific conjugation with electron-rich arenes, we conducted extensive investigations combining experimental studies and performed density functional theory (DFT)-based calculation. Resorcinol can readily form free radicals in the presence of Cu­(II) and O_2_,^33^ however, radical scavengers such as TEMPO, DMPO, BHT, and 1,1-diphenylethylene, did not inhibit the reaction under standard conditions (Figure S108). While the reaction was significantly inhibited when conducted in DMSO with DMPO as a radical scavenger, we did not observe the formation of DMPO-resorcinol adduct (Figure S108). Yet, using electron paramagnetic resonance (EPR) spectroscopy, we observed a signal highly indicative of an aryl carbon radical in the presence of both Cu­(II) and phloroglucinol **2e**, which diminished by exposing to peptides containing either Cys or Sec (Figures S103–S107), but not Ala. These results may suggest an aromatic carbon-centered radical intermediate is involved during the reaction mechanism.

We next investigated the process of aromatic radical reaction with Sec. Free phenyl radicals can be readily captured by Sec to form Se–C bonds, as confirmed by EPR study in previous work.^16^ To determine whether Sec conjugation with electron-rich aromatic radicals occurs through a substrate radical binding to copper or direct capture by Sec, we performed DFT calculations (see Supporting Information for details). The Sec-peptide-Cu­(II) complex^16^ **1a** is reduced by **2a** via an exergonic proton-coupled electron transfer (PCET, Scheme S8a) process^34^ facilitated by a water cluster (the reaction becomes more favorable as the number of water molecules in the cluster increases, see Scheme S9), generating the Cu­(I)-coordinated Sec-peptide (**1a**_**1**_) and the radical of **2a** (**2a·**). Next, **1a**_**1**_ and **2a·** form the reaction complex (**RC**) in the substrate radical binding route, which is exergonic by 7.1 kcal/mol (Figure 7). Analysis of the spin population of **RC** reveals that the spin populations on Cu and C1 are 0.43 and 0.13, respectively (Figure 7). The radical properties of **2a·** are delocalized to Cu­(I) ions and aromatic ring, as indicated by the spin population results, explaining why radical signals were difficult to detect experimentally. Subsequently, the Se1–C1 bond forms via **TS1** with a free energy barrier of 19.5 kcal/mol, which is significantly favorable compared to the substrate radical-free route (directly captured by Sec, see Scheme S8), leading to intermediate **Int1**. The path from **RC** to **Int1** is a concerted process through **TS1**, which corresponds to Se1–Se2 bond homolytic and Se1–C1 bond formation. Finally, **Int1** undergoes tautomerization, yielding the product **Int2** that is 9.1 kcal/mol lower than **1a**_**1**_ and **2a·**, indicating thermodynamically favorable process for the entire reaction (Figure 7).

**Figure 7:**
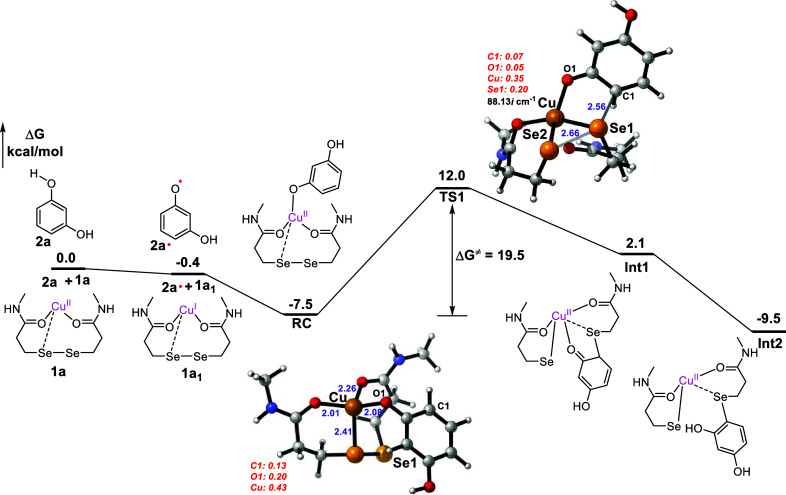
DFT-calculated energy profile on Cu­(II)-mediated modification reaction of Sec-peptide. Distances are given in Å. The spin population on key atoms is shown in *red* italics. The imaginary frequencies for transition states are also shown.

In short, the DFT calculations support our conclusion that substrate **2a** undergoes PCET first, followed by binding to the Cu ion center, and subsequent Se–C bond formation. Overall, the reaction is exergonic by 9.5 kcal/mol with a rate-determining barrier of 19.5 kcal/mol, indicating that the reaction could proceed facilely. Beyond the radical-based mechanism, we also explored two additional pathways (**B** and **C**, see Scheme S8) for the reaction process, inspired by Buchwald and Pentelute’s work^12a,12b^ (Scheme S8), but were found to be less feasible compared to our proposed mechanism (Figure S109), and therefore were excluded. While this mechanism focuses on a monomeric copper catalyst, alternative pathways involving multicopper clusters may also be possible. In the case of Cys bioconjugation, comparable efficiency was observed when the Cys disulfide dimer, but not reduced Cys, especially under N_2_ atmosphere (Figure S110), was used as the starting material (Figure S24b), suggesting that the reaction proceeds via the same mechanistic pathway as proposed for the Sec. This was further supported by DFT calculations (see Schemes S9 and S10).

## Conclusions

In conclusion, we have described a method that is both efficient and broadly applicable for the chemoselective modification of Sec and/or Cys-containing peptides and proteins under mild conditions. This method exhibits excellent selectivity and compatibility, as demonstrated by the use of a series of commercial small molecules with different scaffolds and different peptide and protein sequences. The selective functionalization of Sec can also be achieved by tuning the electron density of aromatic molecules in the presence of Cys, allowing for targeted biochemical reactions. EPR and radical trapping experiments, as well as DFT calculations, support that the conjugation approach is driven by the radical-mediated arylation of Sec and/or Cys with electron-rich (hetero)­arenes in the presence of Cu­(II) ions. Given these results, we believe our chemical method will be widely applied in the future for modifying proteins and generating therapeutically valuable protein conjugates. Ongoing research in our group is focused on investigating the labeling and adapting of more intricate biological molecules both *in vivo* and *in vitro* using this technique.

## Supplementary Material



## Notes

Materials and methods, characterization data, and synthesis procedures (PDF)
