# Supplementary material for: Late-Stage Aromatic C–H Bond Functionalization for Cysteine/Selenocysteine Bioconjugation
Source: J Am Chem Soc. 2025 Aug 19;147(35):31811–20. doi: 10.1021/jacs.5c08936 (PMC12412152; doi:10.1021/jacs.5c08936)
Supplement: Supplementary file 1 [file ja5c08936_si_001.pdf]

Late-Stage Aromatic C–H Bond Functionalization for Cysteine/Selenocysteine Bioconjugation

Zhenguang Zhao,[1,3,⊥] Jian Huang,[1,4,⊥] Yao Cai,[1,⊥] Tai-Ping Zhou,[2,⊥] Fatina Khatib,[1]

Daphna Shimon,[1,*] Binju Wang,[2,*] and Norman Metanis[1,5,6,*]

[1]Institute of Chemistry, The Hebrew University of Jerusalem, Jerusalem 9190401, Israel.
[2]State Key Laboratory of Physical Chemistry of Solid Surfaces and Fujian Provincial Key Laboratory of Theoretical and Computational Chemistry, College of Chemistry and Chemical Engineering, Xiamen University, Xiamen 361005, China.
[3]Department of Chemistry and Chemical Biology, Harvard University, Cambridge, MA 02138, USA
[4]School of Chemistry and Chemical Engineering, Jinggangshan University, Ji'an, Jiangxi 343009, China
[5]The Center for Nanoscience and Nanotechnology, [6]Casali Center for Applied Chemistry, The Hebrew University of Jerusalem, Jerusalem 9190401, Israel.

Supporting Information

Table of Contents

1. General Materials and Methods S2
2. Experimental Section S3
3. Optimization Reaction Conditions S10
4. The Modification of Model Peptide **1** and **4** with Different Small Molecules S19
5. Sec/Cys-specific Modification in the Presence of Other Reactive Residues S48
6. Peptide Stapling S53
7. Stepwise Cross-coupling S57
8. Protein Synthesis S62
9. Protein Expression S70
10. Protein Modification S72
11. EPR Experiment S78
12. Density Functional Theory (DFT) Calculations S86
13. ICP-MS S100
14. NMR Spectra S101
15. HRMS of Protein and Protein Conjugates S135
References S148

1. General Materials and Methods

1) Reagents and materials

Unless otherwise stated, all commercial reagents and solvents were used without additional purification. All Fmoc-amino acids were purchased from CS Bio Co. (Menlo Park, CA) or Matrix Innovation (Quebec City, Canada). *N,N,N',N'*-Tetramethyl-O-(6-chloro-1H-benzotriazol-1-yl)uronium hexafluorophosphate (HCTU) and Ethyl cyano(hydroxyimino)acetate (OxymaPure) were purchased from Luxembourg Biotechnologies Ltd. (Rehovot, Israel). $Na_2HPO_4 \cdot 12H_2O$, 4-mercaptophenylacetic acid (MPAA), *N,N′*-Diisopropylcarbodiimide (DIC), triisopropylsilane (TIPS), tris(2-carboxyethyl)phosphine hydrochloride (TCEP·HCl), 2,2′-Dithiobis (5-nitropyridine) (DTNP), sodium ascorbate (NaAsc), $Cu(OTf)_2$, Seleno-*L*-cystine were purchased from Sigma-Aldrich (Rehovot, IL). EDTA, disodium salt dihydrate was purchased from J. T. Baker. TentaGel® R RAM resin (loading 0.18 mmol/g), H-Rink Aminde-Chemmatrix® resin and 2-Chlorotrityl resin (loading 1.8 mmol/g) were purchased from Rapp Polymer GmbH (Germany), GL Biochemical (China) or Chem-impex (USA). All solvents: *N,N*-dimethylformamide (DMF), Diethyl ether (Et_2O), dichloromethane (DCM), acetonitrile (MeCN), *N,N*-diisopropylethyl amine (DIEA), Trifluoroacetic acid (TFA) and piperidine (Pip) were purchased from Bio-Lab (Jerusalem, IL) and were peptide synthesis, HPLC or ULC-grade. Buffers for all the reactions were prepared by using MilliQ water (Millipore, Merck). ^{1}H- and ^{13}C-NMR spectra were recorded on a Bruker 400 or 500 MHz instruments with chemical shifts reported in ppm relative to the residual deuterated solvent. Fmoc-Sec(Mob)-OH synthesis was reported previously.[2]

2) High Performance Liquid Chromatography (HPLC).

Analytical reversed-phase HPLC (RP-HPLC) was performed on Waters Alliance HPLC with 220 nm UV detection using XSelect C18 column (3.5 μm, 130 Å, 4.6 × 150 mm), XSelect C18 column (5.0 μm, 130 Å, 4.6 × 150 mm) and XBridge C4 column (3.5 μm, 4.6 × 150 mm). Semi-preparative RP-HPLC was performed on a Waters LCQ150 system using a XSelect C18 column (5 μm, 10 × 150 mm) or XBridge C4 column (5 μm, 10 × 150 mm).

Preparative RP-HPLC was performed on a Waters LCQ150 system using a XSelect C18 column (5 μm, 30 × 250 mm) or XBridge C4 column (5 μm, 19 × 150 mm).

Linear gradients of ACN (with 0.1 % TFA, buffer B) in water (with 0.1 % TFA, buffer A) were used for all systems to elute bound peptides. The flow rates were 1 mL/min (analytical), 3.4 mL/min (Semi-preparative), and 20 mL/min (preparative).

Methods for HPLC

Method A HPLC condition: XSelect C18 column (5.0 μm, 130 Å, 4.6 × 150 mm), column temp: 30 ^{o}C, gradient of 5%-70% ACN in H_2O with 0.1 TFA over 25 min.

Method B HPLC condition: XSelect C18 column (3.5 μm, 130 Å, 4.6 × 150 mm), column temp: 25 ^{o}C, gradient of 5%-60% ACN in H_2O with 0.1 TFA over 25 min.

Method C HPLC condition: XSelect C18 column (3.5 μm, 130 Å, 4.6 × 150 mm), column temp: 25 ^{o}C, gradient of 5%-50% ACN in H_2O with 0.1 TFA over 25 min.

Method D HPLC condition: XSelect C18 column (5.0 µm, 130 Å, 4.6 × 150 mm), column temp: 30 °C, gradient of 5%-40% ACN in H_2O with 0.1 TFA over 25 min.
Method E HPLC condition: XBridge C4 column (3.5 µm, 4.6 × 150 mm), column temp: 30 °C, gradient of 5%-70% ACN in H_2O with 0.1 TFA over 25 min.
Method F HPLC condition: XBridge C4 column (3.5 µm, 4.6 × 150 mm), column temp: 25 °C, gradient of 5%-60% ACN in H_2O with 0.1 TFA over 25 min.

The yields were determined by calculating the peak area integration from the HPLC spectra at a wavelength of 220 nm.

3) Electrospray Ionization Mass Spectrometry (ESI-MS), Matrix-assisted laser desorption/ionization (MALDI) and High-Resolution Mass Spectrometry (HR-MS).

Small peptides and protein ESI mass spectra data were obtained on a LCQ Fleet Ion Trap mass spectrometer (Thermo Scientific) in the positive mode or Bruker autoflex MALDI TOF/TOF system (α-Cyano-4-hydroxycinnamic acid matrix was used). The HR-MS were recorded on an Agilent 6520 QTOF analyzer (Agilent Technologies, Inc., United States) with a dual electrospray ionization source. The raw data were deconvoluted using MagTran v1.03.

4) MS/MS analysis of labelled peptide conjugates.

Tandem MS analysis was performed on Q Exactive-HF mass spectrometer (Thermo Fisher Scientific, Waltham, MA USA) coupled on-line to an Ultimate 3000 Dionex (Thermo Fisher Scientific, Waltham, MA USA) UHPLC.

2. Experimental Section

1) General Procedure for Fmoc Solid-Phase Peptide Synthesis.

Peptides were synthesized manually by Fmoc-SPPS typically on a 0.25 mmol scale. Fmoc-deprotection was carried out with 20% piperidine in DMF (10 min, 2×). Fmoc-amino acids (1 mmol in 5 mL of DMF, 4 equiv) were activated with HATU or HCTU (1 mmol in 5 mL of DMF, 4 equiv) and DIEA (2 mmol in 5 mL of DMF, 8 equiv) for 5 min and allowed to couple for 30 min with constant shaking. Fmoc-Sec(Mob)-OH coupling was performed by DIC/OxymaPure procedure using 2 equiv of Fmoc-Sec(Mob)-OH. The resulting resins were washed with DMF (3×), DCM (3×) and dried. The peptide was cleaved off resin using a TFA:triisopropylsilane (TIPS):H_2O (95:2.5:2.5) cocktail or, if Mob-protecting group was present added more 2 equiv DTNP (when Sec is present)[2] for 3 h. The cleavage mixture was filtered, and the resin was washed with TFA. After the TFA was evaporated through N_2 bubbling, peptide was precipitated with cold ether, centrifuged, resuspended in 30% ACN in H_2O, and lyophilized to dryness.
The crude peptide was dissolved in 30% ACN in H_2O containing 0.1% TFA and purified by multiple injections of 70 mg each on prep RP-HPLC (XSelect C18 column, 5 µm, 30 × 250 mm) using a gradient of 10%-50% B over 70 min. HPLC fractions

containing only product materials were confirmed by ESI-MS analysis, combined, and then lyophilized. Peptides purified by HPLC are listed in Table S1.

Table S1. Peptide sequences.

Peptide	Sequence
**1a**	TFUGK-NH_2 (dimer)
**1**	LGUALG-NH_2 (dimer)
**4**	LGCALG-NH_2
**12**	LGAALG-NH_2
**13**	LHYWAG-NH_2
**14**	AUGKSFEMNR-NH_2
**15**	ACGKSFEMNR-NH_2
**16**	GUANSLRFYHDK-NH_2 (dimer)
**17**	CRAFT-NH_2
**18**	SAPDTRPAPGUTAPPAHGVTSA-OH (dimer)
**19**	SAPDTRPAPGCTAPPAHGVTSA-NH_2
**20**	GUANKHTWYLUA-NH_2
**21**	GUALNKFQEKSRMKYRWKHRCG-NH_2
**22**	GCANKHTWYLCA-NH_2
**23**	GCANSLRFYHDK-NH_2

2) **HPLC and ESI-MS analytical data for purified peptides.**

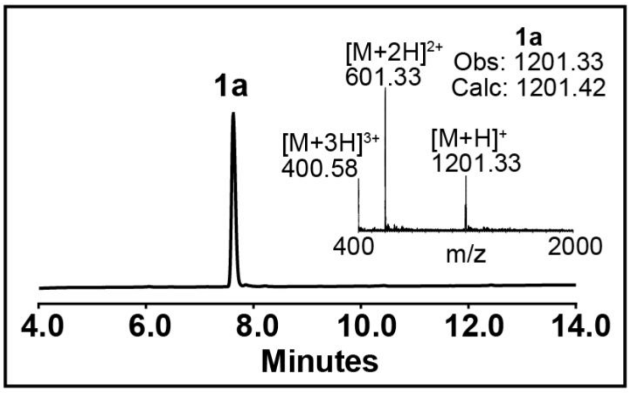

Peptide 1a. HPLC **method A** and ESI-MS of **1a** ($[M+H]^+$ obs. 1201.33, calc. 1201.42).

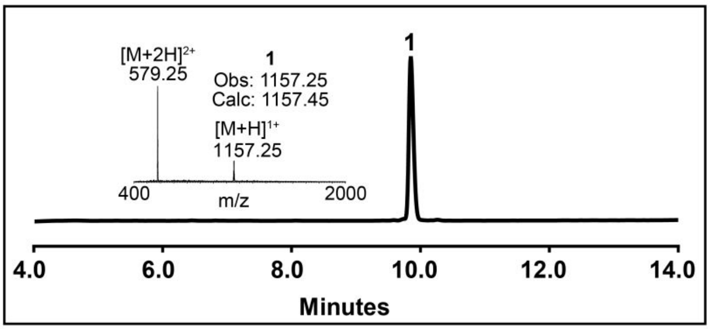

Peptide 1. HPLC **method A** and ESI-MS of **1** ([M+H]$^+$ obs. 1157.25, calc. 1157.45).

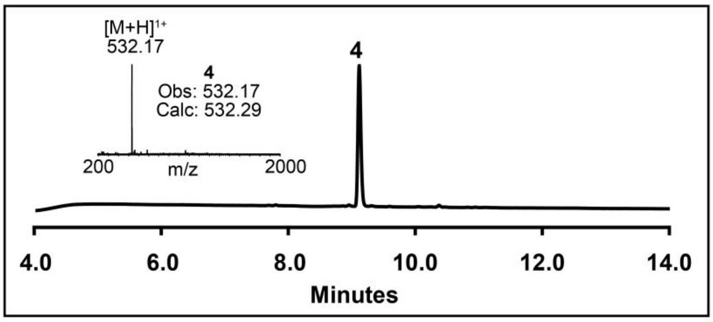

Peptide 4. HPLC **method B** and ESI-MS of **4** ([M+H]$^+$ obs. 532.17, calc. 532.29).

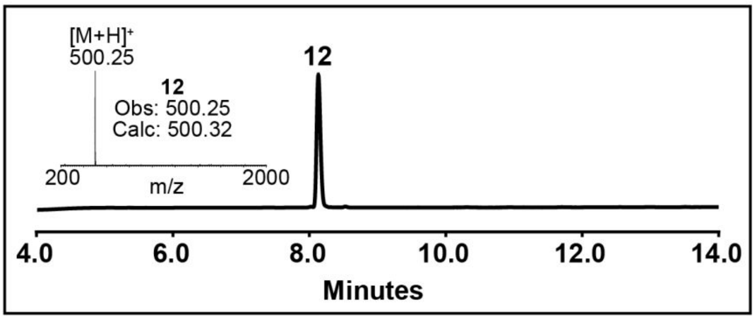

Peptide 12. HPLC **method B** and ESI-MS of **12** ([M+H]$^+$ obs. 500.25, calc. 500.32).

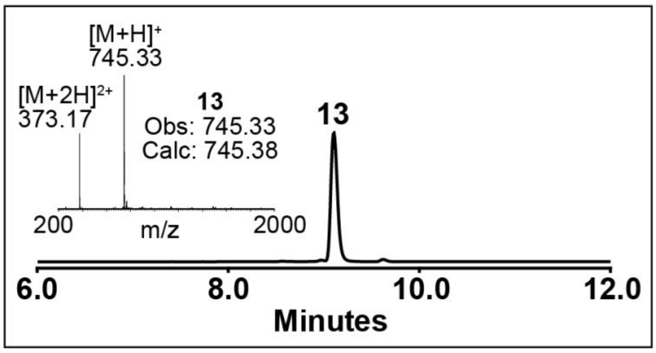

Peptide 13. HPLC **method B** and ESI-MS of **13** ([M+H]$^+$ obs. 745.33, calc. 745.38).

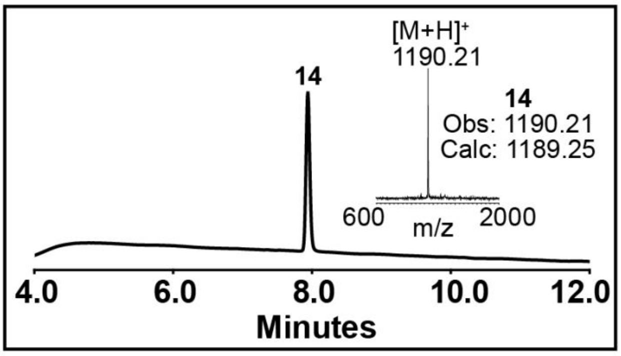

Peptide 14. HPLC **method A** and ESI-MS of **14** ([M+H]$^+$ obs. 1190.21, calc. 1189.25).

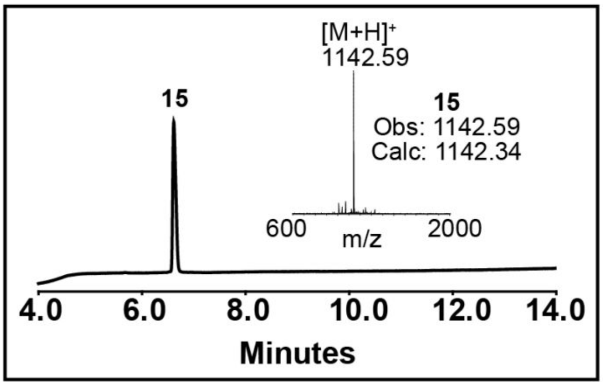

Peptide 15. HPLC **method A** and ESI-MS of **15** ([M+H]$^+$ obs. 1142.59, calc. 1142.34).

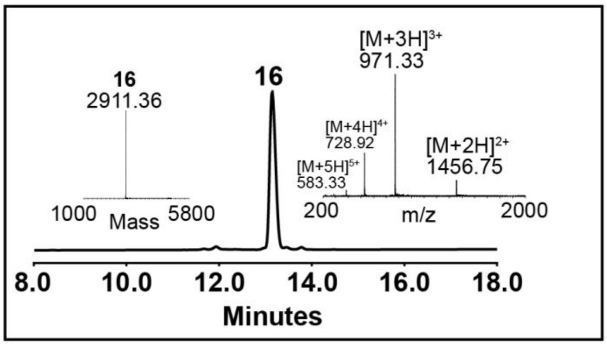

Peptide 16. HPLC **method C** and ESI-MS of **16** (mass obs. 2911.36 Da, calc. 2910.99 Da).

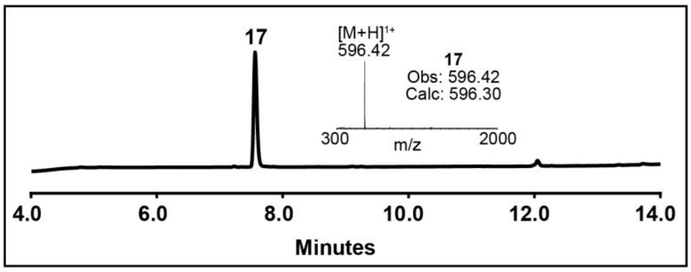

Peptide 17. HPLC **method A** and ESI-MS of **17** ([M+H]$^+$ obs. 596.42, calc. 596.30).

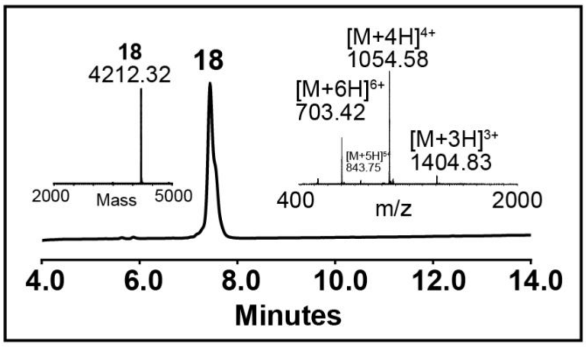

Peptide 18. HPLC **method C** and ESI-MS of **18** (mass obs. 4212.32 Da, calc. 4212.36

Da).

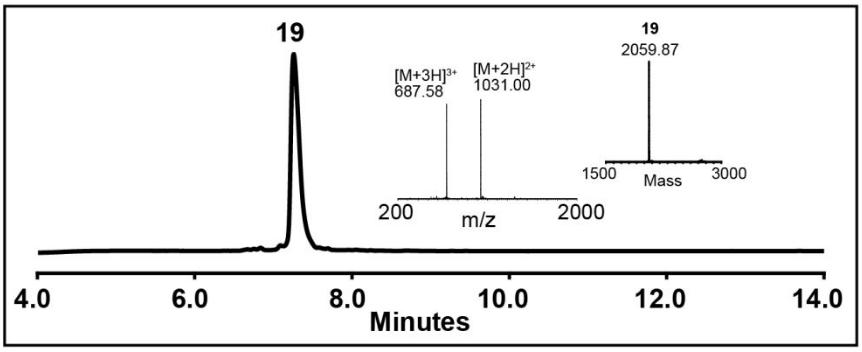

Peptide 19. HPLC **method A**, ESI-MS of **19** (mass obs. 2059.87 Da, calc. 2060.28 Da).

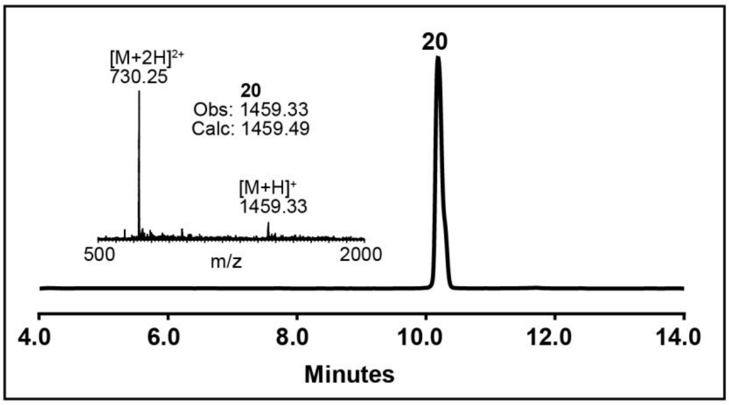

Peptide 20. HPLC **method B** and ESI-MS of **20** ($[M+H]^+$ obs. 1459.33, calc. 1459.49).

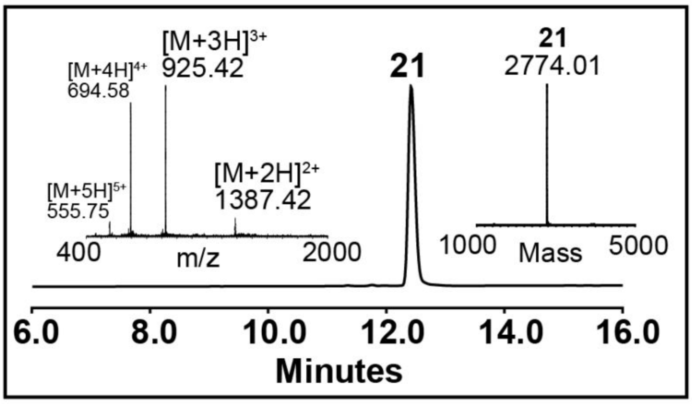

Peptide 21. HPLC **method A**, ESI-MS of **21** (mass obs. 2774.01 Da, calc. 2773.16 Da).

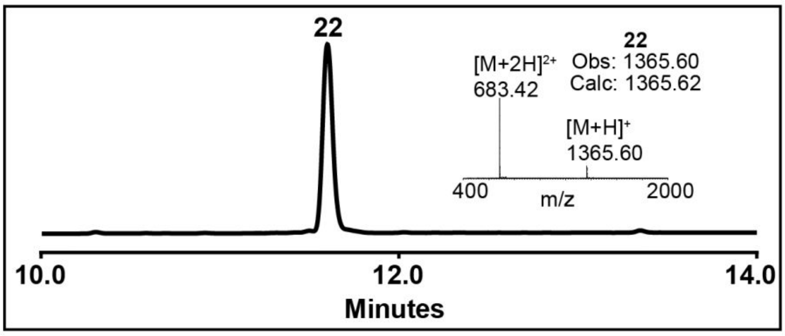

Peptide 22. HPLC **method C**, ESI-MS of **22** ([M+H]$^+$ obs. 1365.60, calc. 1365.62).

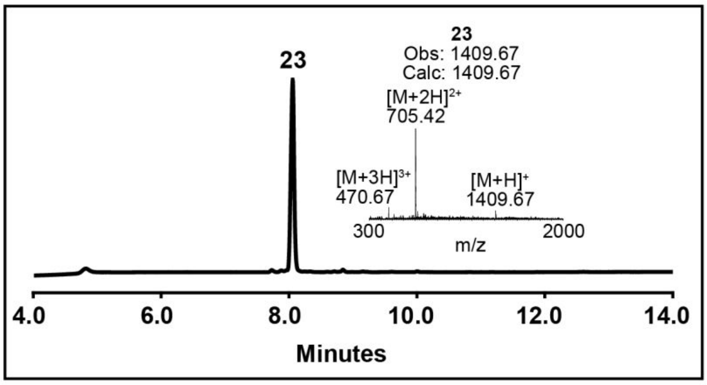

Peptide 23. HPLC **method C**, ESI-MS of **23** ([M+H]$^+$ obs. 1409.67, calc. 1409.67).

3) General procedure (A) for copper-mediated Synthesis of Arylated Sec containing peptides

A 1.5 mL Eppendorf tube was charged with 880 or 870 μL of deionized H_2O, 50 μL of 200 mM phosphate buffer (PB, pH 8.0, final conc. 10 mM), 10 μL of $Cu(OTf)_2$ stock solution (100 mM stock solution in H_2O, final conc. 1 mM), 0 μL or 10 μL of 2,2'-bipyridine stock solution (100 mM stock solution in EtOH, final conc. 0 or 1 mM) and 50 μL of small molecule stock solution (100 mM stock solution in H_2O, final conc. 5 mM), 10 μL of peptide (100 mM stock solution in H_2O, final conc. 1 mM). The resulting reaction mixture was capped, vortexed for 30 seconds, and placed in a 37 °C water bath for the indicated time (15 min - 2 h). The reaction progress was monitored by HPLC and ESI-MS.

4) General procedure (B) for copper-mediated Synthesis of Arylated Cys containing peptides

A 1.5 mL Eppendorf tube was charged with 900 or 890 μL of deionized H_2O, 50 μL of 200 mM PB (pH 8.0, final conc. 10 mM), 10 μL of $Cu(OTf)_2$ stock solution (100 mM stock solution in H_2O, final conc. 1 mM), 0 μL or 10 μL of 2,2'-bipyridine stock solution (100 mM stock solution in EtOH, final conc. 0 or 1 mM) and 30 μL of small molecular stock solution (100 mM stock solution in H_2O, final conc. 3 mM), 10 μL of peptide (100 mM stock solution in H_2O, final conc. 1 mM). The resulting reaction mixture was capped, vortexed for 30 seconds, and placed in a 37 °C water bath for the indicated time (1-5 h). The reaction progress was monitored by HPLC and ESI-MS.

3. Optimization Reaction Conditions

3.1 The finding of direct aromatic C-H functionalization for Sec-specific conjugation

1. 0.5 mM TFUGK-NH_2 **1a** and 1 mM resorcinol **2a** were incubated at room temp in 15 mM PB (pH 7). After 22 h, no conjugate was observed by LC-MS.

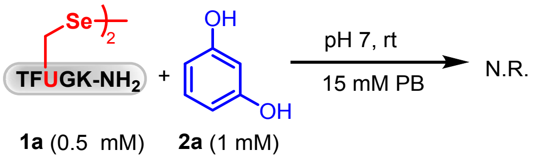

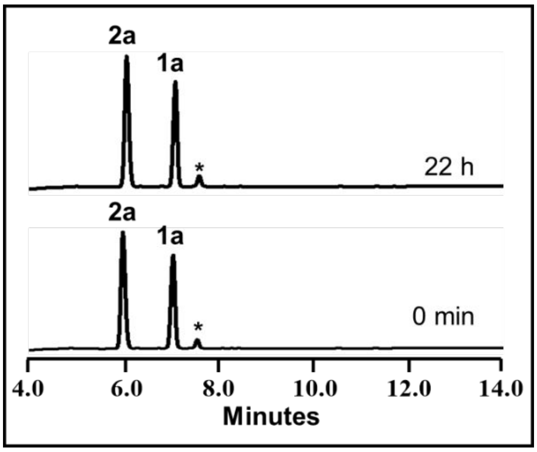

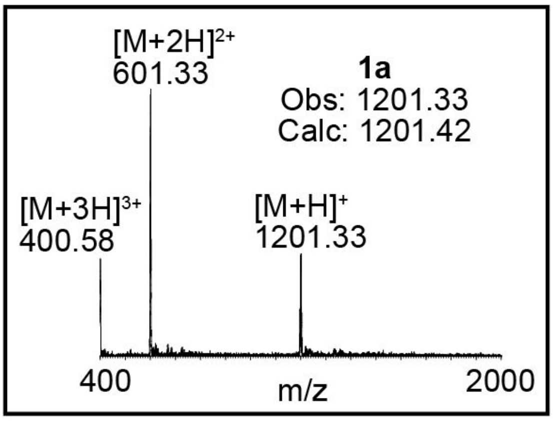

Figure S1. Resorcinol is inert toward Sec in the absence of copper.

2. 0.5 mM TFUGK-NH_2 **1a**, 1 mM resorcinol **2a** and 0.5 equiv $CuSO_4$ were incubated at room temp in 15 mM PB (pH 7). After 17 h, Sec-conjugates were observed with 98% conversion by LC-MS. * is impurity from peptide **1a**.

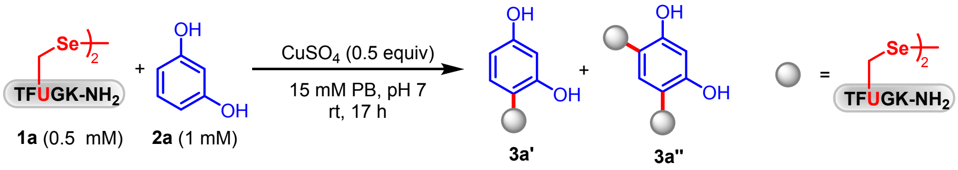

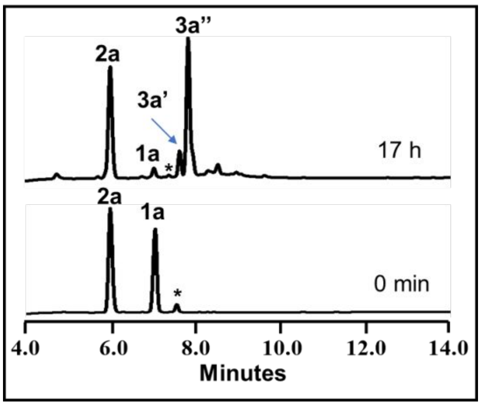

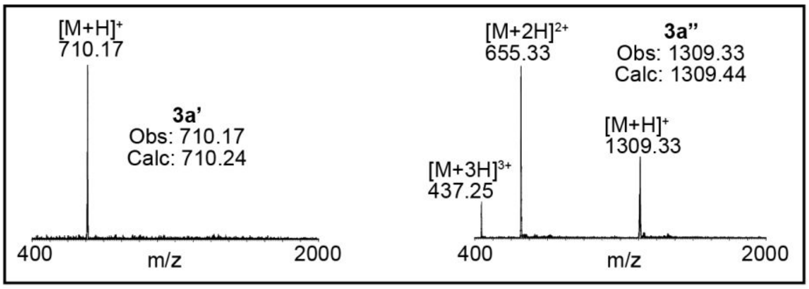

Figure S2. Copper-mediated Sec-conjugation with resorcinol

3.2 The Optimization of Reaction Conditions for Model Peptide 1 and 4 Modification

According to general approach for peptide modification, the stock solution of model peptide **1** (100 mM), model peptide **4** (100 mM), small molecule **2a** (100 mM) and $Cu(OTf)_2$ (100 mM) were prepared for the optimization of reaction conditions.

*1. The effect of pH on model peptide **1** modification*

10 μL $Cu(OTf)_2$ stock solution (1 μmol, final conc. 1 mM) and 50 μL **2a** stock solution (5 μmol, final conc. 5 mM) were dissolved in 880 μl of deionized H_2O, 50 μL of 200 mM PB (pH 8.0, final conc. 10 mM) were added into four different Eppendorf tubes, then the reaction solution was adjusted to pH 6, 7, 8 and 9, respectively, by 0.5 M NaOH or 0.5 M HCl. Lastly, 10 μL of peptide **1** stock solution (1 μmol, final conc. 1 mM) were added dropwise to corresponding above reaction mixtures, respectively, and the reactions were incubated at 37 °C for 15 min. The reaction progress was monitored by HPLC **method A** and ESI-MS. The results are shown in Fig. S3. # is a deselenization side-product from **1**. * is inferred to be 2,6-disubsitituted conjugate due to its identical mass to **3a₂**, however, NMR characterization was not possible due to the low conversion.

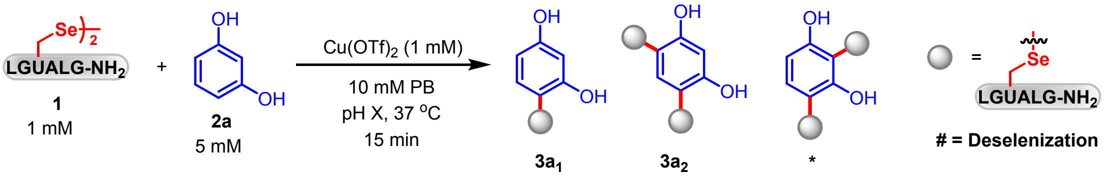

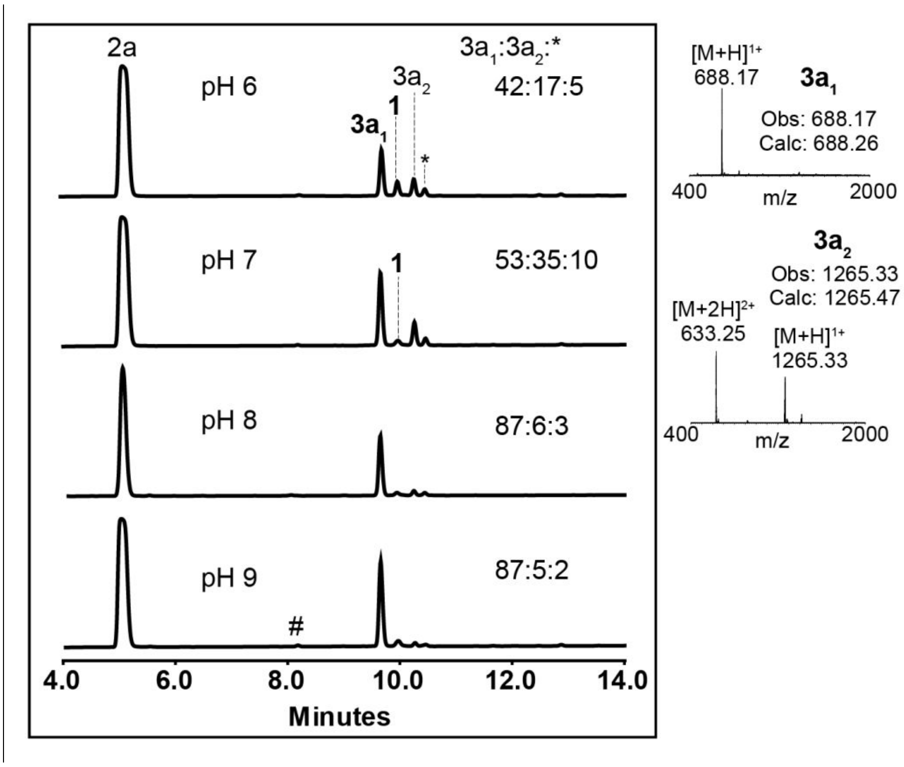

Figure S3. The effect of pH on model peptide **1** modification.

*2. The effect of the amount of **2a** for model peptide **1** modification*

10 μL $Cu(OTf)_2$ stock solution (1 μmol, final conc. 1 mM), and 10 μL, 20 μL, 30 μL , 40 μL and 50 μL **2a** stock solution (1-5 μmol, final conc. 1-4 mM) were dissolved in 840 μl, 850 μl, 860μl, 870μl and 880 μl of deionized H_2O, 50 μL of 200 mM PB (pH 8.0, final conc. 10 mM) were added into five different Eppendorf tubes, then the reaction solution was adjusted to pH 8 by 0.5 M NaOH. Lastly, 10 μL peptide **1** stock solution (1 μmol, final conc. 1 mM) were added dropwise to corresponding above reaction mixtures, respectively, and incubated at 37 °C for 15 min. The reaction progress was monitored by HPLC **method A** and ESI-MS. The results are shown in Fig. S4. * is inferred to be 2,6-disubsititued conjugate due to its identical mass to $\mathbf{3a_2}$, however, NMR characterization was not possible due to the low conversion.

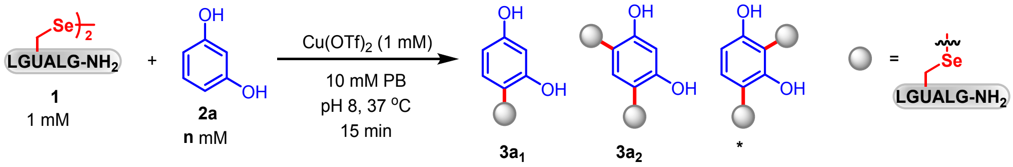

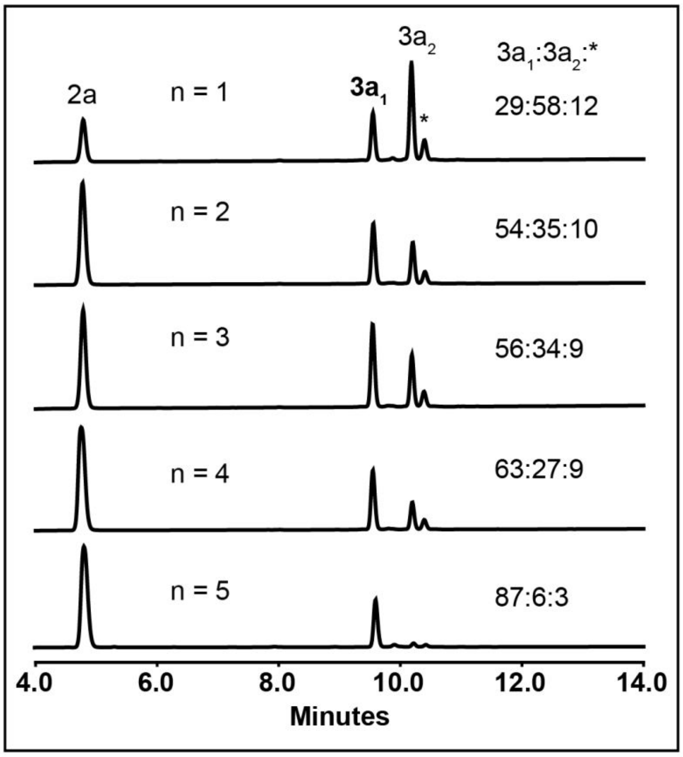

Figure S4. The effect of the amount of **2a** for model peptide **1** modification.

3. *The effect of different catalyst on model peptide **1** modification*

10 µL *catalyst* stock solution (1 µmol, final conc. 1 mM) and 50 µL **2a** stock solution (5 µmol, final conc. 5 mM) were dissolved in 880 µL of deionized H_2O, 50 µL of 200 mM PB (pH 8.0, final conc. 10 mM) were added into eleven different Eppendorf tubes, then the reaction solution was adjusted to pH 8 by 0.5 M NaOH. Lastly, 10 µL peptide **1** stock solution (1 µmol, final conc. 1 mM) were added to corresponding above reaction mixtures, respectively, and incubated at 37 °C for 15 min. The reaction progress was monitored by HPLC **method A** and ESI-MS. The results are shown in Fig. S5. # is a deselenization side-product from **1**. * is inferred to be 2,6-disubsitituted conjugate due to its identical mass to **3a**$_2$, however, NMR characterization was not possible due to the low conversion.

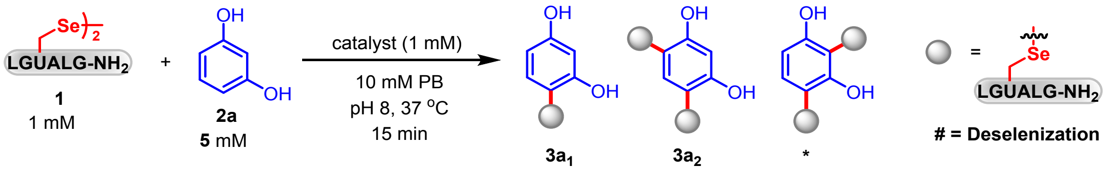

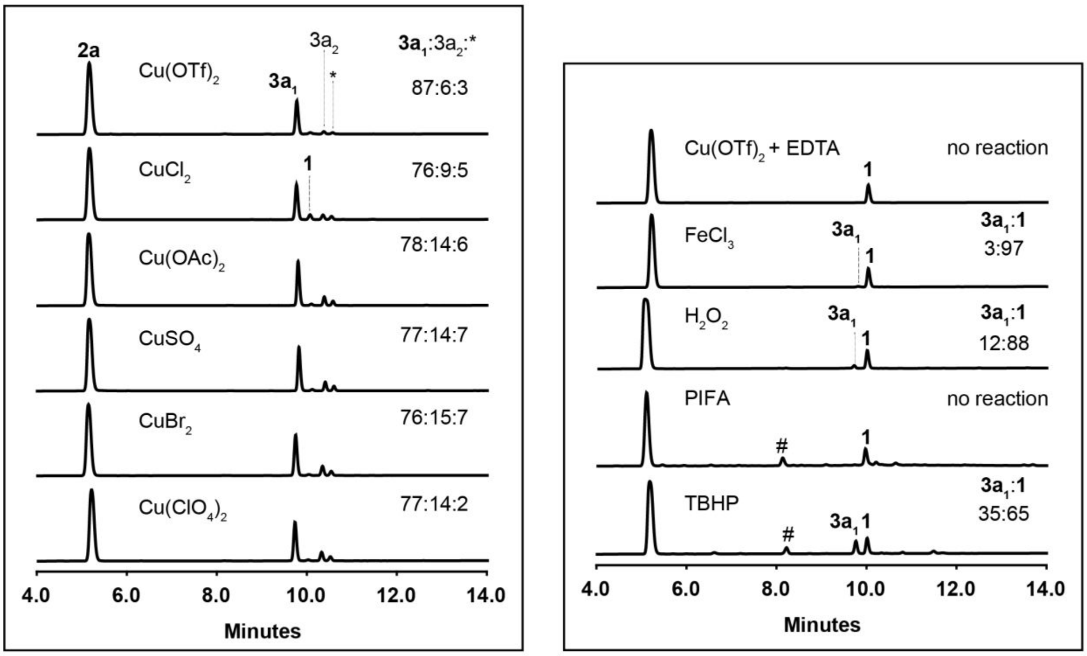

Figure S5. The effect of different catalyst on model peptide **1** modification.

*4. The effect of the amounts of $Cu(OTf)_2$ on model peptide **1** modification*

5 μL (0.5 equiv), 10 μL (1.0 equiv), 20 μL (2.0 equiv), 30 μL (3.0 equiv) and 40 μL (4.0 equiv) $Cu(OTf)_2$ stock solution and 50 μL **2a** stock solution (5 μmol, final conc. 5 mM) were dissolved in 885 μL, 880 μL , 870 μL, 860 μL and 850 μL of deionized H_2O, 50 μL of 200 mM PB (pH 8.0, final conc. 10 mM) were added into five different Eppendorf tubes, then the reaction solution was adjusted to pH 8 by 0.5 M NaOH. Lastly, 10 μL peptide **1** stock solution (1 μmol, final conc. 1 mM) were added to corresponding above reaction mixtures, respectively, and incubated at 37 °C for 15 min. The reaction progress was monitored by HPLC **method A** and ESI-MS. The results are shown in Fig. S6. * is inferred to be 2,6-disubsitituted conjugate due to its identical mass to **3a₂**, however, NMR characterization was not possible due to the low conversion.

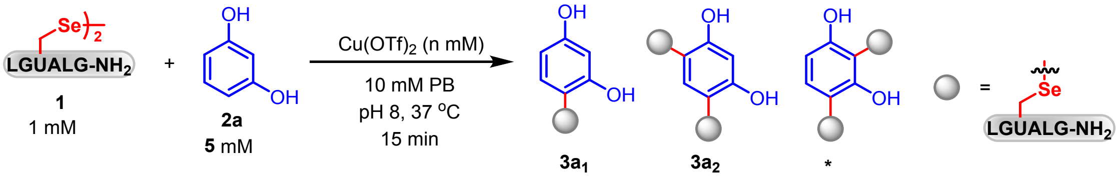

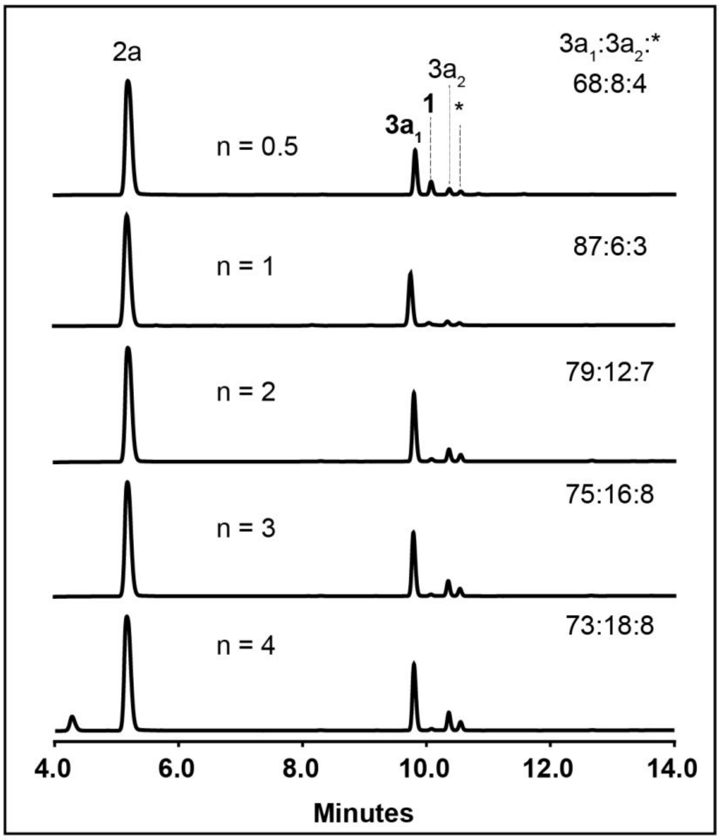

Figure S6. The effect of the amounts of $Cu(OTf)_2$ on model peptide **1** modification.

*5. The effect of various concentration of **2a** and peptide **1***

0.5 μL, 1 μL, 2 μL, 5 μL, 10 μL $Cu(OTf)_2$ stock solution (100 mM) and 2.5 μL, 5 μL, 10 μL, 25 μL, 50 μL **2a** stock solution (100 mM) were dissolved in 946.5 μL, 943 μL , 936 μL, 915 μL, 880 μL of deionized H_2O, 50 μL of 200 mM PB Buffer (pH 8.0, final conc. 10 mM), were added into five different Eppendorf tubes, then the reaction solution was adjusted to pH 8 by 0.5 M NaOH. Lastly, 0.5 μL (final conc. 50 μM), 1 μL (final conc. 100 μM), 2 μL (final conc. 200 μM), 5 μL (final conc. 500 μM) and 10 μL (final conc. 1 mM) of peptide **1** stock solution (100 mM) were added dropwise to corresponding above reaction mixtures, respectively, and incubated at 37 °C for 15 min. The reaction progress was monitored by HPLC **method A** and LC-MS. The results are shown in Fig. S7. * is inferred to be 2,6-disubsitituted conjugate due to its identical mass to **3a₂**, however, full NMR characterization was not possible due to the low conversion.

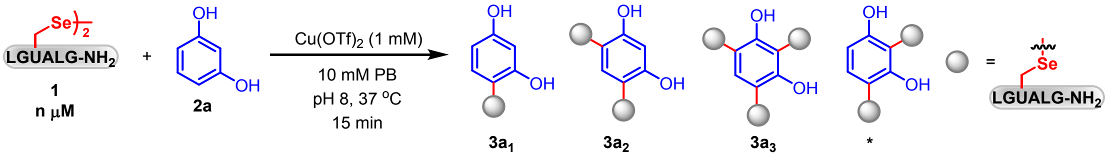

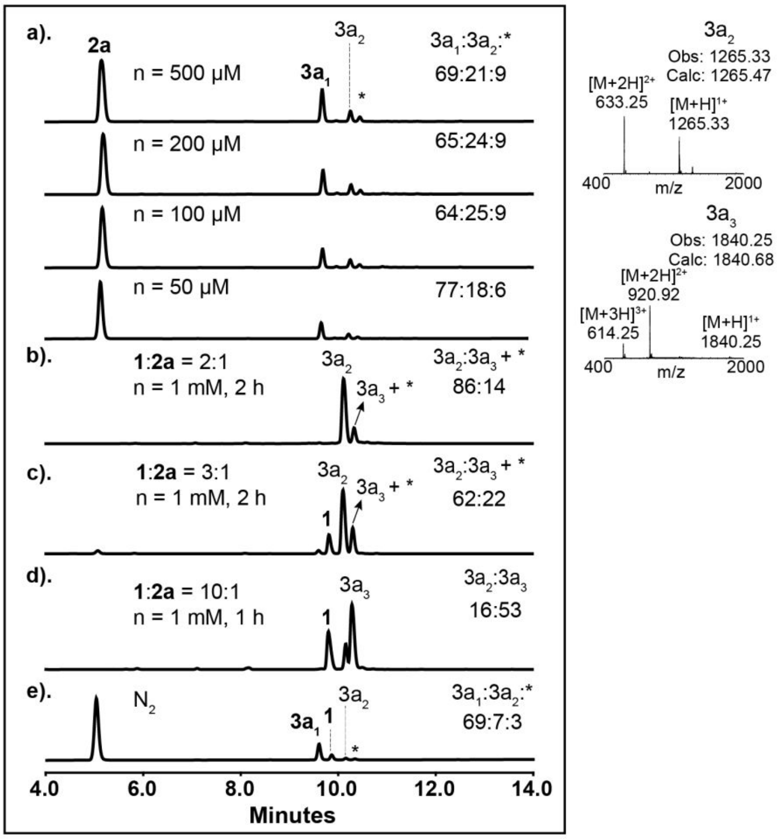

Figure S7. The effect of various concentration of **2a** and peptide **1**. **a**). Different concentrations of peptide **1**. **b**). Modification of 1 mM peptide **1** with 0.5 mM **2a**. **c**). Modification of 1 mM peptide **1** with 0.333 mM **2a**. **d**). Modification of 1 mM peptide **1** with 0.1 mM **2a**. **e**). The reaction was conducted with 1 mM peptide **1**, 5 mM **2a** and 1 mM $Cu(OTf)_2$ under N_2 atmosphere.

6. *The effect of the amounts of **2a** on model peptide **4** modification*

10 μL (1.0 equiv) $Cu(OTf)_2$ stock solution and 5 μL (final conc. 0.5 mM), 10μL (final conc. 1 mM), 20 μL (final conc. 2 mM), 30 μL (final conc. 3 mM) and 50 μL (final conc. 5 mM) **2a** stock solution (100 mM) were dissolved in 925 μL, 920 μL , 910 μL, 900 μL and 880 μL of deionized H_2O, 50 μL of 200 mM PB (pH 8.0, final conc. 10 mM), were added into five different Eppendorf tubes, then the reaction solution was adjusted to pH 8 by 0.5 M NaOH. Lastly, 10 μL peptide **4** stock solution (1 μmol, final conc. 1 mM) were added dropwise to corresponding above reaction mixtures, respectively, and incubated at 37 °C for 1 h. The reaction progress was monitored by HPLC. HPLC condition: XSelect C18 column (5 μm, 130 Å, 4.6 × 150 mm), column temp: 30 ^{o}C, gradient of 5%-60% ACN in H_2O with 0.1 TFA over 25 min. and characterized by ESI-MS. The results are shown in Fig. S8. * is an oxidation side-product from **4** and **4’** is the dimer of **4**.

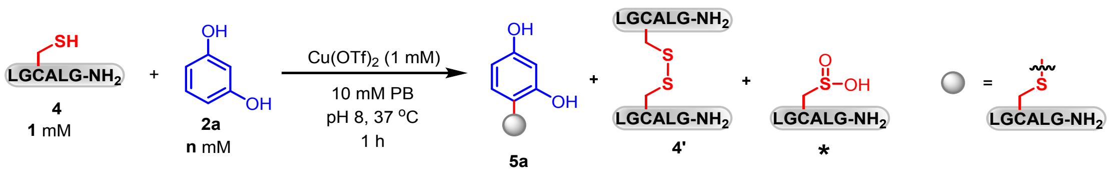

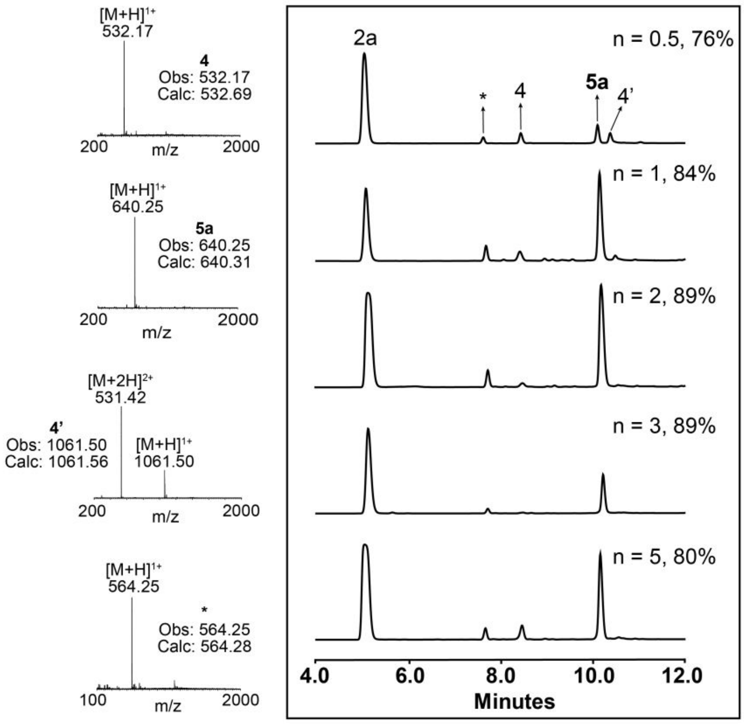

Figure S8. The effect of the amounts of **2a** on model peptide **4** modification.

*7. The effect of the amounts of $Cu(OTf)_2$ on model peptide **4** modification*

5 µL (0.5 equiv), 10 µL (1.0 equiv), 20 µL (2.0 equiv) and 30 µL (3.0 equiv) $Cu(OTf)_2$ stock solution and 30 µL **2a** stock solution (3 µmol, final conc. 3 mM) were dissolved in 905 µL, 900 µL, 890 µL and 880 µL of deionized H_2O, 50 µL of 200 mM PB Buffer (pH = 8.0), were added into four different Eppendorf tubes, then the reaction solution was adjusted to pH 8 by 0.5 M NaOH. Lastly, 10 µL peptide **4** stock solution (1 µmol, final conc. 1 mM) were added dropwise to corresponding above reaction mixtures, respectively, and incubated at 37 °C for 1h. The reaction progress was monitored by HPLC. HPLC condition: XSelect C18 column (5 µm, 130 Å, 4.6 × 150 mm), column temp: 30 °C, gradient of 5%-60% ACN in H_2O with 0.1 TFA over 25 min. and characterized by ESI-MS. The results are shown in Fig. S9. * is an oxidation side-product of **4** and **4’** is the dimer of **4**.

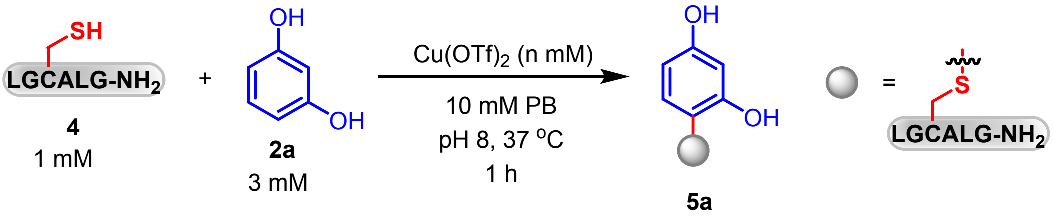

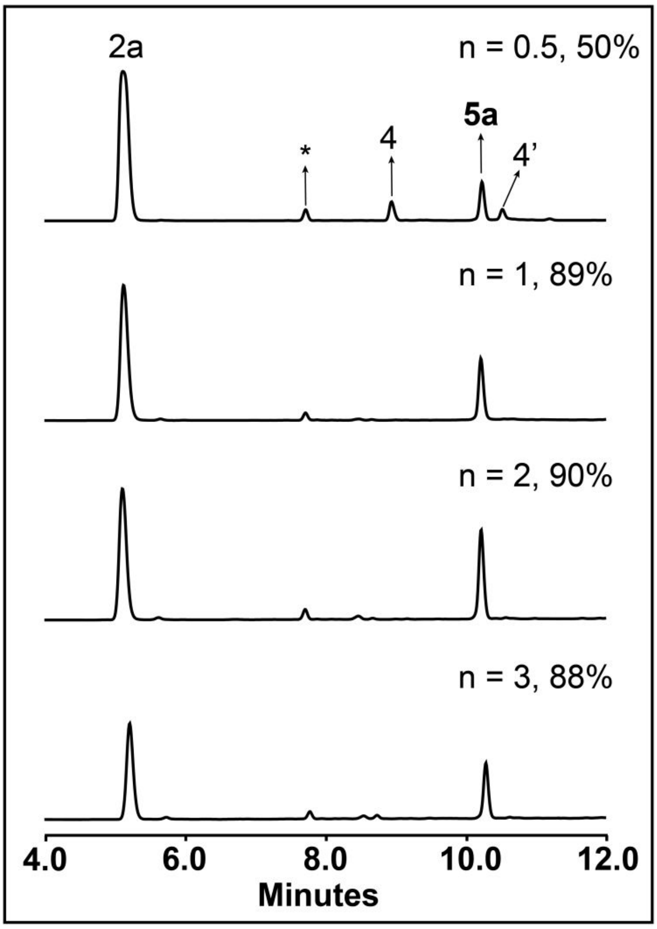

Figure S9. The effect of the amounts of $Cu(OTf)_2$ on model peptide **4** modification.

4. The Modification of Model Peptide 1 and 4 with Different Small Molecules

According to general approach for peptide modification, the stock solution of model peptide **1** (100 mM), peptide **4** (100 mM), small molecular (100 mM), bipyridine (100 mM dissolved in EtOH) and $Cu(OTf)_2$ (100 mM) were prepared for peptide modification.

*1. The modification of model peptide **1** with **2a**.*
($3a_1$): Prepared according to the general procedure A, the reaction progress was monitored by HPLC **method A**, conversion: 99% (**$3a_1$**:**$3a_2$**:* 87:6:3). **$3a_1$** was characterized by ESI-MS ($[M+H]^+$ obs. 688.17, calc. 688.26) and NMR (Fig. S110-S114). The results are shown in Fig. S10. The structures of **$3a_2$**, and * is inferred from the MS data and plausible steric hindrance, further characterization was not possible due to the low conversion.

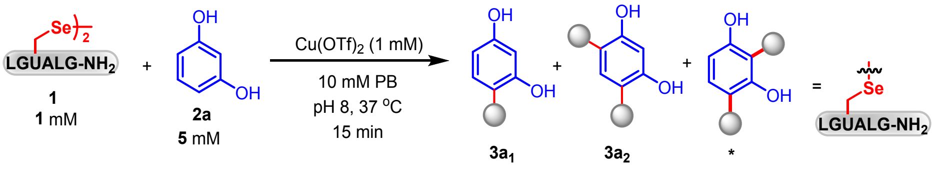

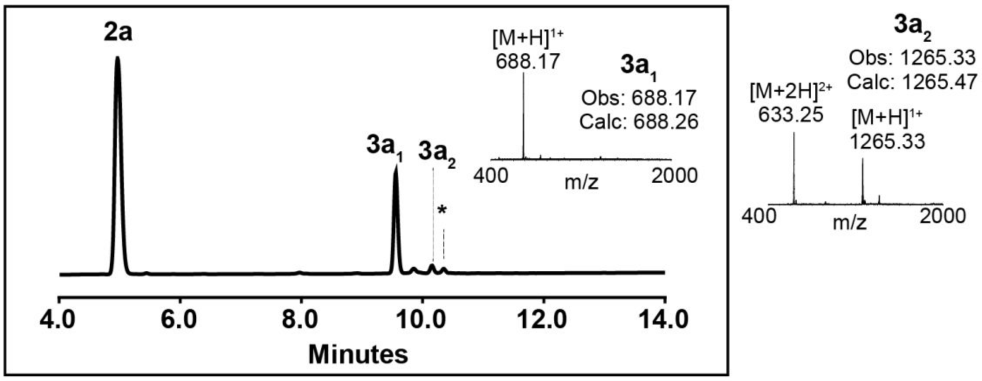

Figure S10. The modification of model peptide **1** with **2a**.

*2. The modification of model peptide **1** with **2b**.*
($3b_1$): Prepared according to general procedure A, the reaction progress was monitored by HPLC **method A**, conversion: 99% (**$3b_1$**:**$3b_2$** 96:3). **$3b_1$** was characterized by ESI-MS ($[M+H]^+$ obs. 702.08, calc. 702.27) and NMR (Fig. S115-S117). The results are shown in Fig. S11. The structure of **3bb** is inferred from the MS data and plausible steric hindrance, further characterization was not possible due to the low conversion.

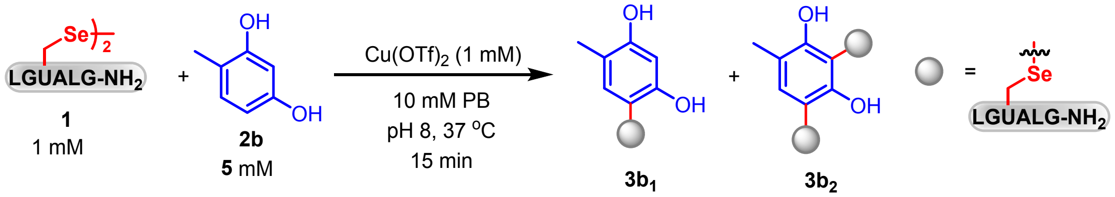

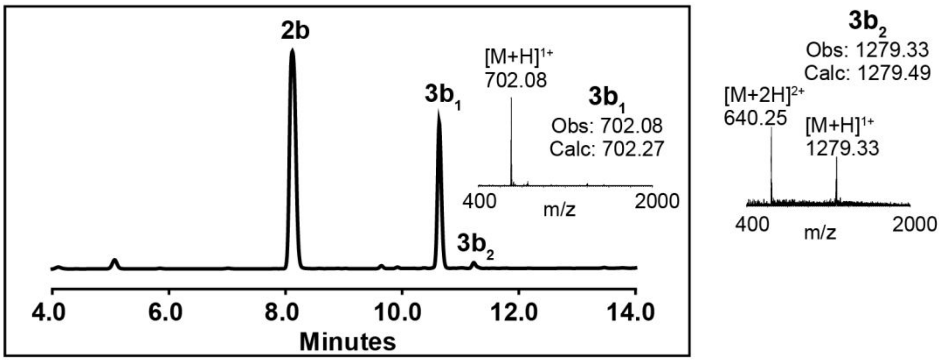

Figure S11. The modification of model peptide **1** with **2b**.

*3. The modification of model peptide **1** with **2c**.*

($3c_1$): Prepared according to the general procedure A, the reaction progress was monitored by HPLC **method A**, conversion: 99% (**$3c_1$:$3c_2$:$3c_2$*** 89:2:8). The product was characterized by ESI-MS ($[M+H]^+$ obs. 702.17, calc. 702.27) and NMR (Fig. S118-S120). The results are shown in Fig. S12. The structures of **$3c_2$** and **$3c_2$*** are inferred from the MS data and plausible steric hindrance, further characterization was not possible due to the low conversion.

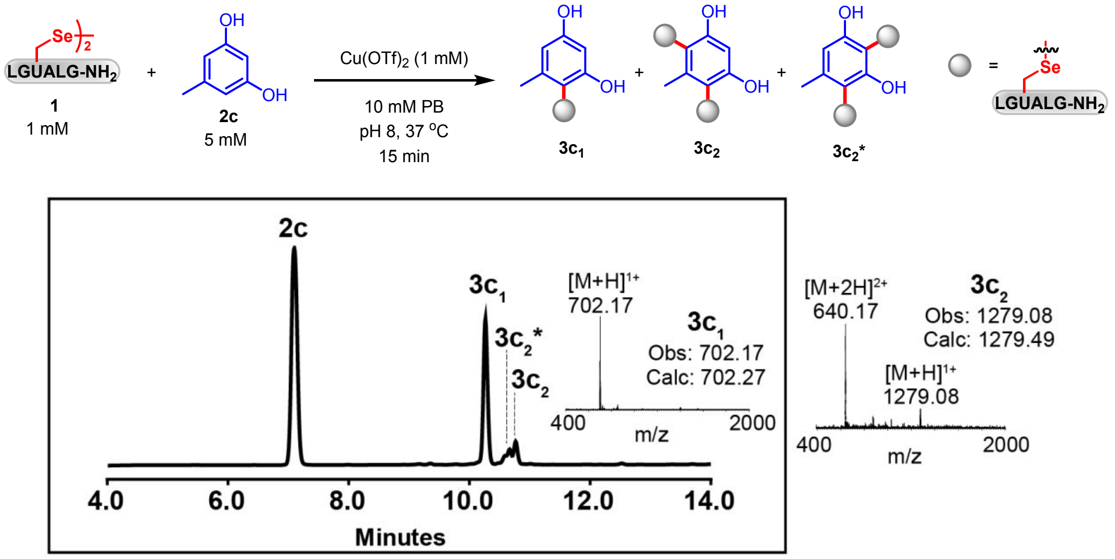

Figure S12. The modification of model peptide **1** with **2c**.

*4. The modification of model peptide **1** with **2d**.*

($3d_1$): Prepared according to the general procedure A, the reaction progress was monitored by HPLC **method A**, conversion: 99% (**$3d_1$:$3d_2$:$3d_2$*** 76:10:14). The product was characterized by ESI-MS ($[M+H]^+$ obs. 702.17, calc. 702.27) and NMR (Fig. S121). The results are shown in Fig. S13. The structures for **$3d_2$** and **$3d_2$*** are inferred from the MS data and plausible steric hindrance, further characterization was not possible due to the low conversion.

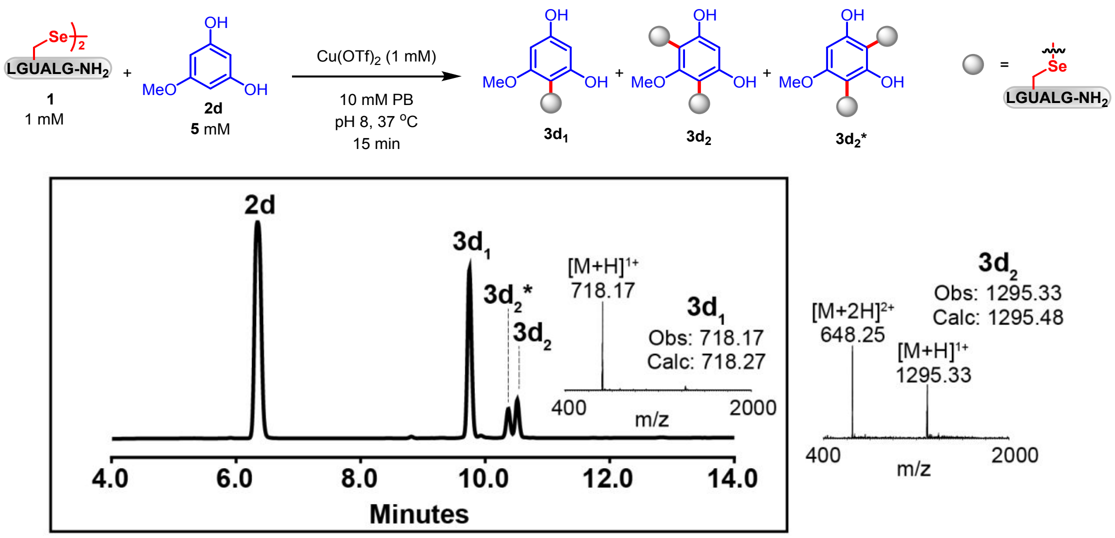

Figure S13. The modification of model peptide **1** with **2d**.

*5. The modification of model peptide **1** with **2e**.*

($3e_1$): Prepared according to the general procedure A, the reaction progress was monitored by HPLC **method A**, conversion: 99% (**$3e_1$:$3e_2$** 90:9). The products were characterized by ESI-MS (**$3e_1$**: $[M+H]^+$ obs. 704.17, calc. 704.25; **$3e_2$**: $[M+H]^+$ obs. 1281.25, calc. 1281.46). The results are shown in Fig. S14.

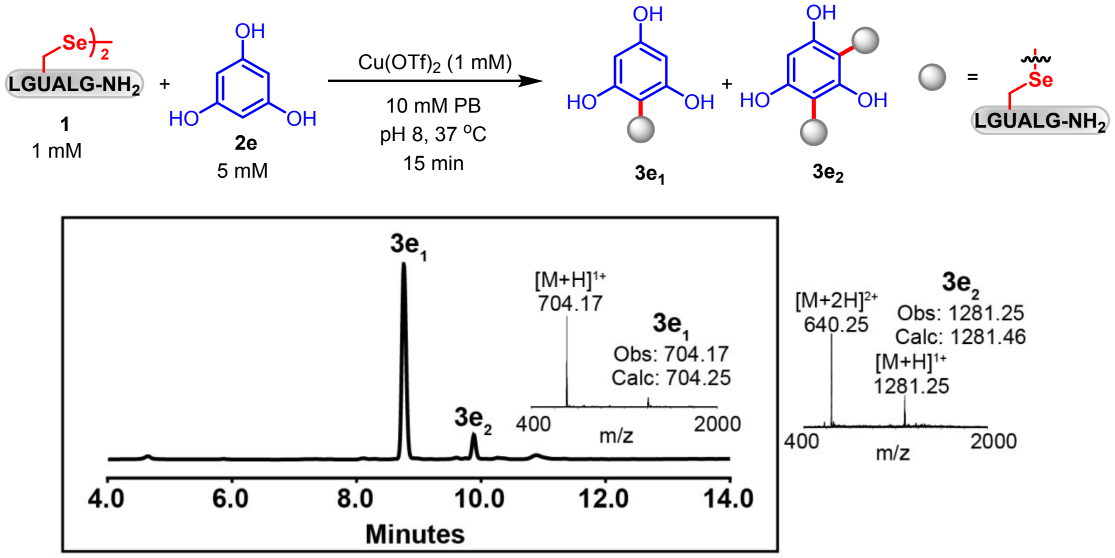

Figure S14. The modification of model peptide **1** with **2e**.

*6. The modification of model peptide **1** with **2f**.*

($3f_1$): Prepared according to general procedure A, the reaction progress was monitored by HPLC **method A**, conversion: 99% (**$3f_1$:$3f_2$** 93:7). The products were characterized by ESI-MS (**$3f_1$**: $[M+H]^+$ obs. 732.25, calc. 732.25; **$3f_2$**: $[M+H]^+$ obs. 1309.25, calc.

1309.46). The results are shown in Fig. S15.

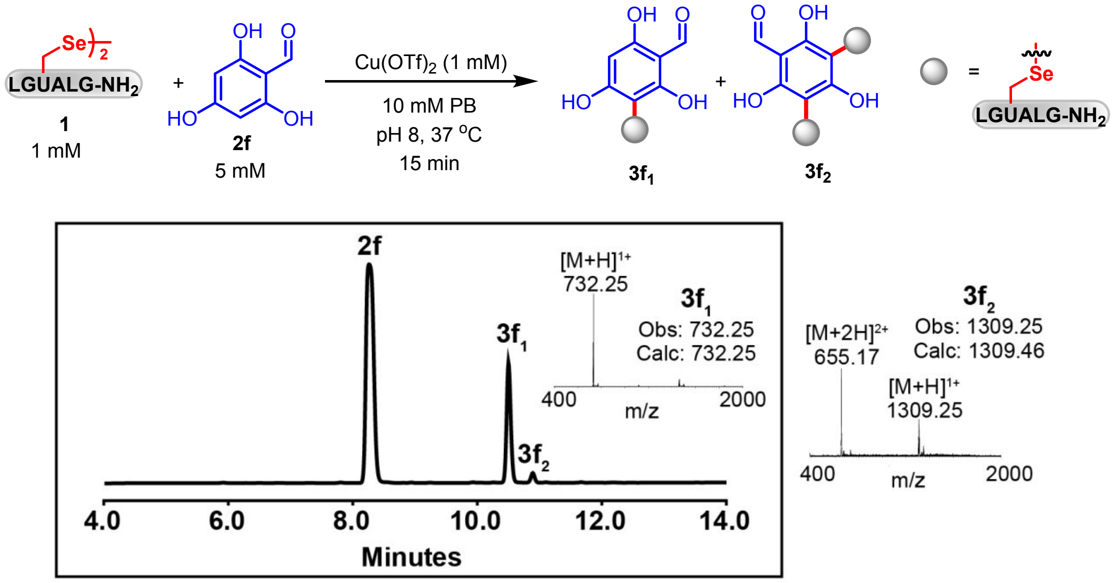

Figure S15. The modification of model peptide **1** with **2f**.

*7. The modification of model peptide **1** with **2g***

(3g): Prepared according to the general procedure A, the reaction progress was monitored by HPLC **method A**, conversion: 99%. The product was characterized by ESI-MS ($[M+H]^+$ obs. 722.25, calc. 722.28) and NMR (Fig. S122). The results are shown in Fig. S16.

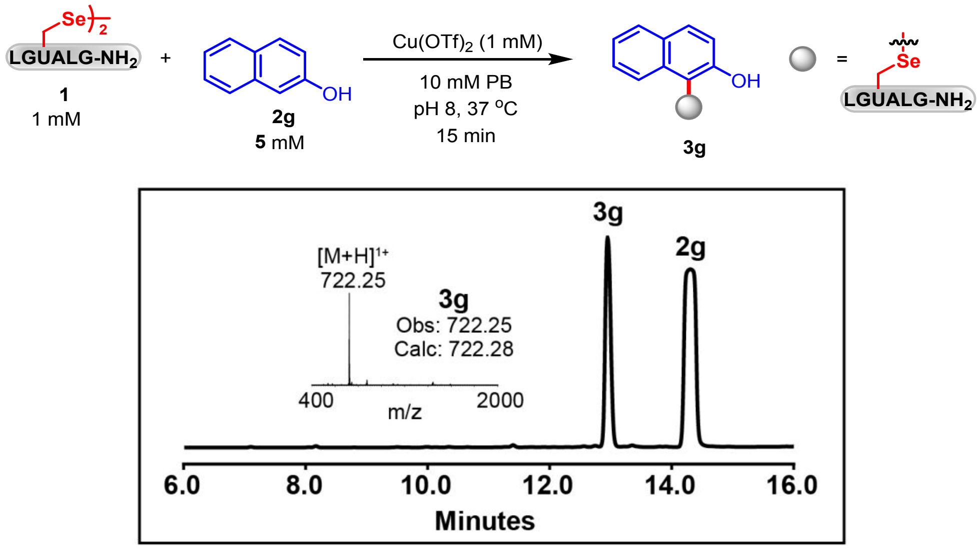

Figure S16. The modification of model peptide **1** with **2g**.

*8. The modification of model peptide **1** with **2h**.*

(3h): Prepared according to the general procedure A, the reaction progress was

monitored by HPLC **method A**, conversion: 99% (**3h**:**3h*** 71:29). **3h** was characterized by ESI-MS ($[M+H]^+$ obs. 687.17, calc. 687.27) and NMR (Fig. S123-S124). The results are shown in Fig. S17. **3h*** is inferred to be another conjugate at a different position on benzene ring, based on its identical mass to **3h.**

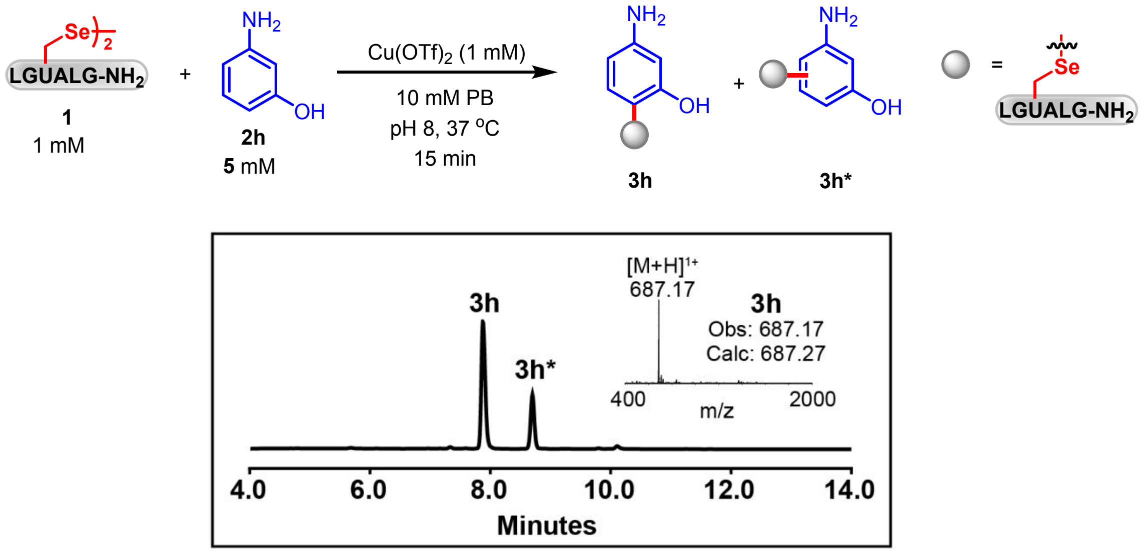

Figure S17. The modification of model peptide **1** with **2h**.

*9. The modification of model peptide **1** with **2i**.*

(3i): Prepared according to the general procedure A, the reaction progress was monitored by HPLC **method A**, conversion: 99%. The product was characterized by ESI-MS ($[M+H]^+$ obs. 715.08, calc. 715.30). The results are shown in Fig. S18.

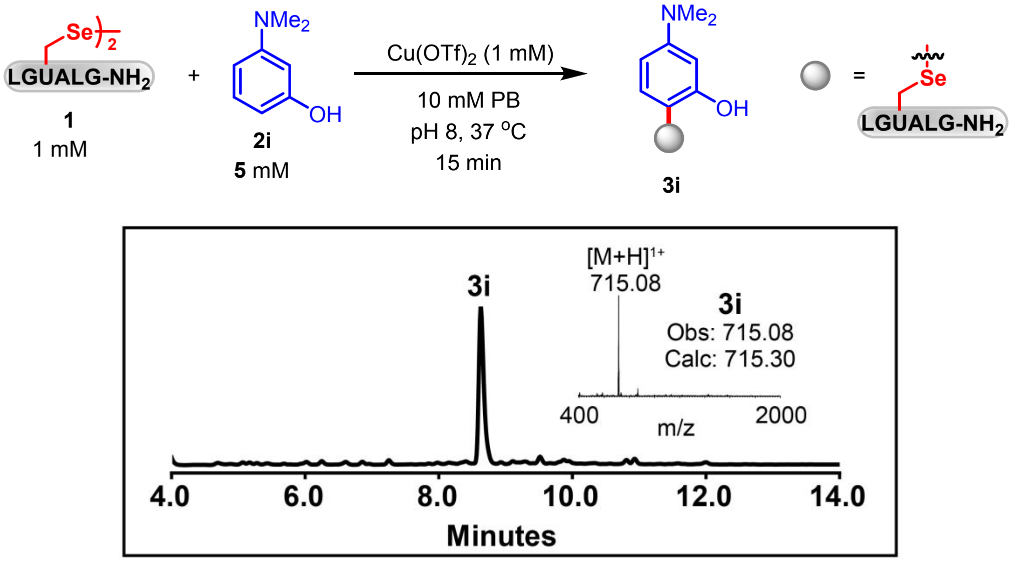

Figure S18. The modification of model peptide **1** with **2i**.

*10. The modification of model peptide **1** with **2j**.*

(3j): Prepared according to the general procedure A, the reaction progress was monitored by HPLC **method A**, conversion: 40%, including three monosubstituted conjugates (**3j$_1$**:**3j$_2$**:**3j$_3$** 19:14:7). The products were characterized by ESI-MS ($[M+H]^+$ obs. 686.17, calc. 686.28), further characterization was not possible due to the low conversion. The results are shown in Fig. S19. * is a oxidation side-product from **1** and # is a deselenization side-product from **1**.

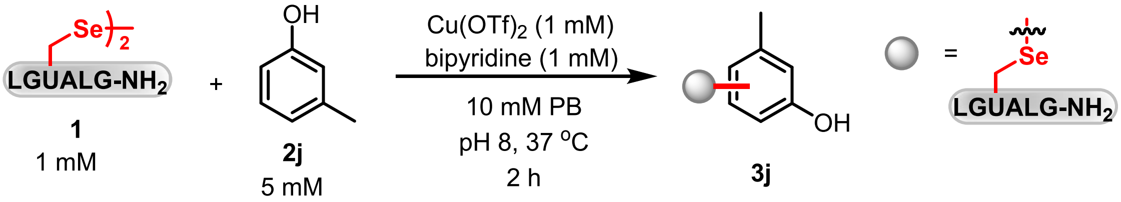

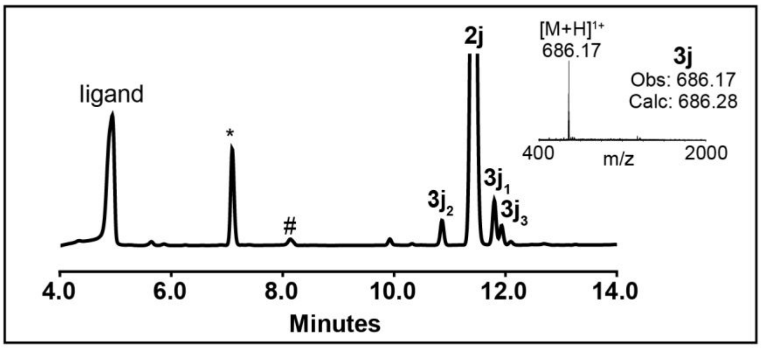

Figure S19. The modification of model peptide **1** with **2j**.

*11. The modification of model peptide **1** with **2k**.*

(3k): Prepared according to the general procedure A, the reaction progress was monitored by HPLC **method A**, conversion: 88% (**3k**:**3k*** 79:9). **3k** was characterized by ESI-MS ($[M+H]^+$ obs. 700.33, calc. 700.29) and NMR (Fig. S125-S126). The results are shown in Fig. S20. The structure of **3k*** is inferred from the MS data ($[M+H]^+$ obs. 700.33, calc. 700.29) and the identified **3k**.

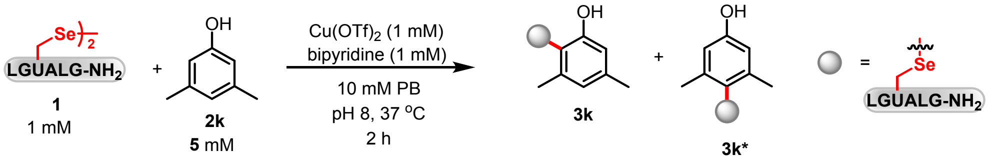

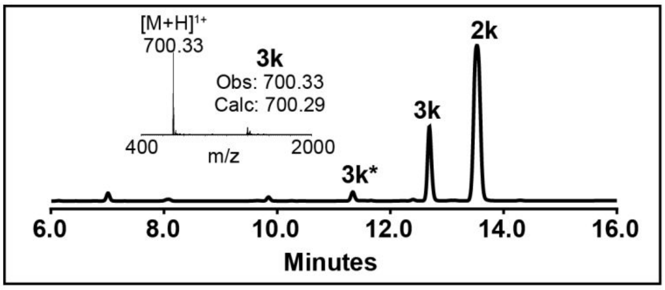

Figure S20. The modification of model peptide **1** with **2k**.

*12. The modification of model peptide **1** with **2l**.*

(3l): Prepared according to the general procedure A, the reaction progress was monitored by HPLC **method A**, conversion: 99% (**3l**:**3l*** 79:21). **3l** was characterized by ESI-MS ($[M+H]^+$ obs. 686.17, calc. 686.29). **3l*** were inferred to be another two monosubstituted conjugates, due to their identical mass to **3l**. Further characterization was not possible due to the low conversion. The results are shown in Fig. S21.

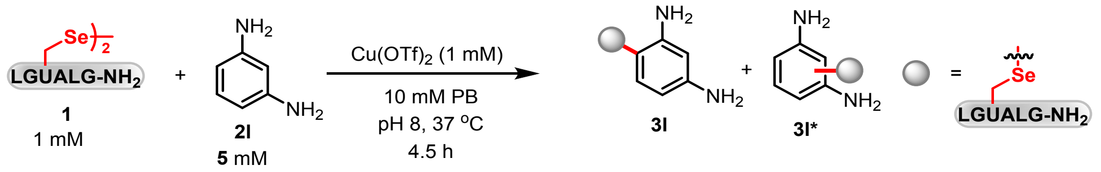

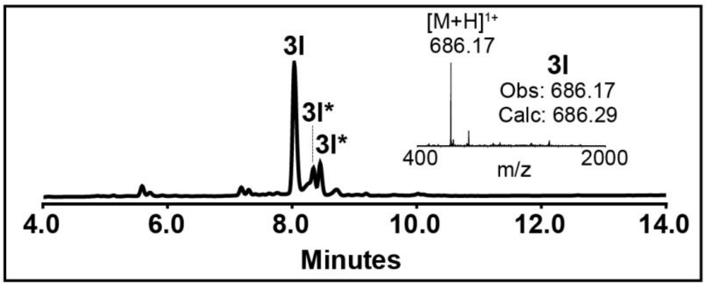

Figure S21. The modification of model peptide **1** with **2l**.

*13. The modification of model peptide **1** with **2m**.*

(3m): Prepared according to the general procedure A, the reaction progress was monitored by HPLC **method A**. There is no functionalization of peptide **1**. The results are shown in Fig. S22.

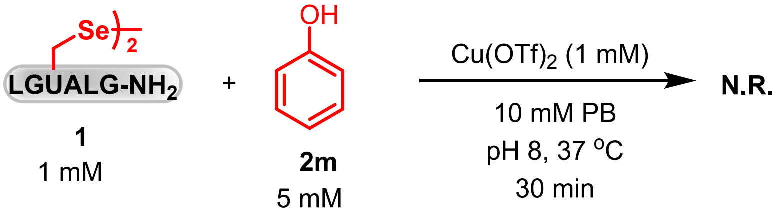

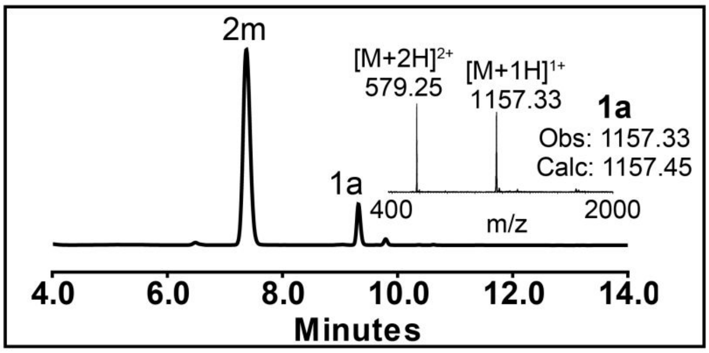

Figure S22. The modification of model peptide **1** with **2m**.

*14. The modification of model peptide **1** with **2n**.*

(3n): Prepared according to the general procedure A, the reaction progress was monitored by HPLC **method A**. There is no functionalization of peptide **1**. The results are shown in Fig. S23. * is an oxidation side-product from **1**.

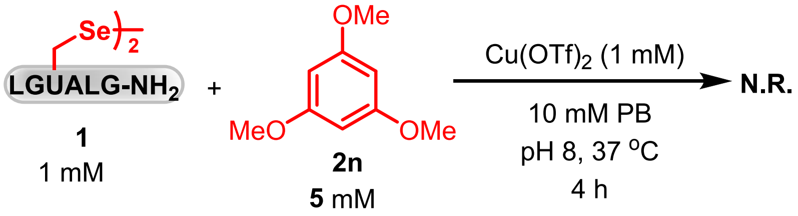

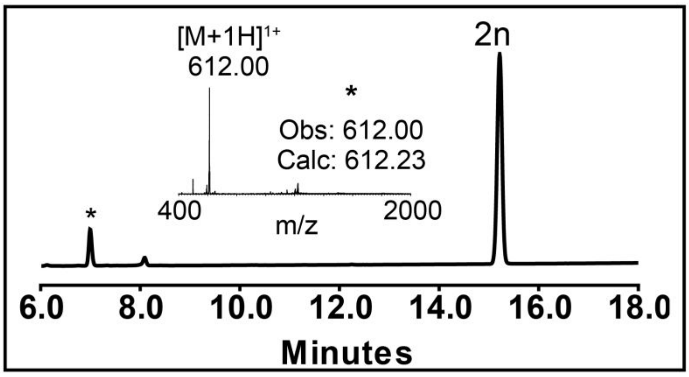

Figure S23. The modification of model peptide **1** with **2n**.

*15. The modification of model peptide **4** with **2a**.*

(5a): Prepared according to the general procedure B, the reaction progress was monitored by HPLC **method B**, conversion: 89%. The product was characterized by ESI-MS ($[M+H]^+$ obs. 640.25, calc. 640.31) and NMR (Fig. S127). The results are shown in Fig. S24. * is an oxidation side-product from **4**. The comparable modification of peptide **4** dimer (**4'**) was achieved, as shown in Fig. S24b. However, the reaction was significantly hindered under a nitrogen atmosphere (Fig. S24c), indicating a strong dependence on the presence of oxygen.

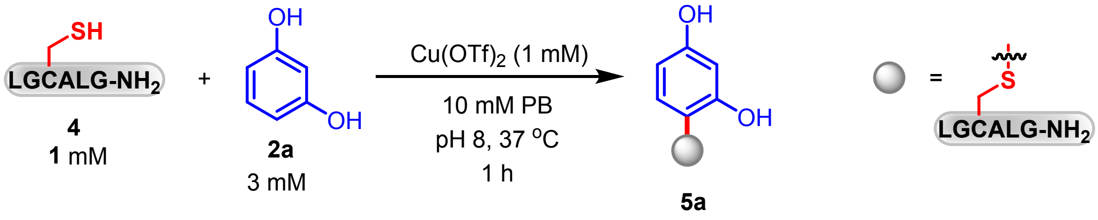

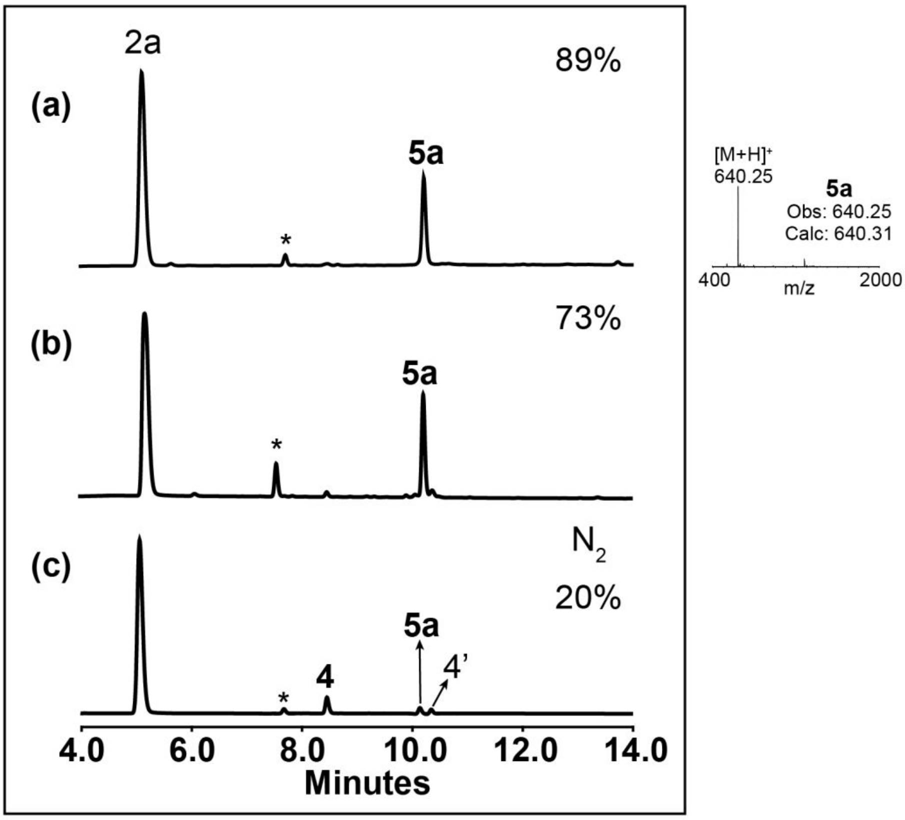

Figure S24. The modification of model peptide **4** with **2a**.

*16. The modification of model peptide **4** with **2b**.*

(5b): Prepared according to the general procedure B, the reaction progress was monitored by HPLC **method B**, conversion: 86%. The product was characterized by ESI-MS ($[M+H]^+$ obs. 654.25, calc. 654.33) and NMR (Fig. S128). The results are shown in Fig. S25. * is a oxidation side-product from **4**, **4'** is the dimer of **4**.

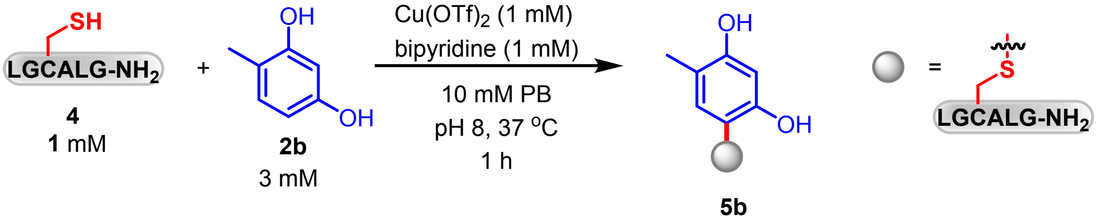

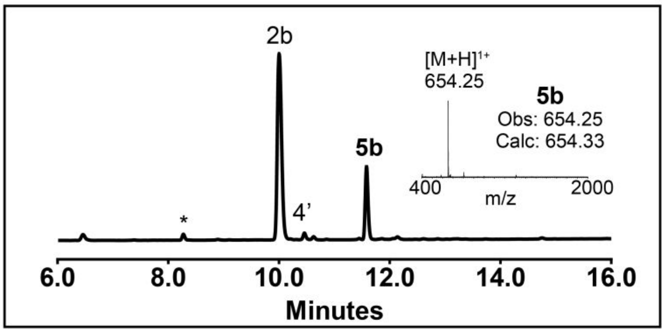

Figure S25. The modification of model peptide **4** with **2b**.

*17. The modification of model peptide **4** with **2c**.*

(5c): Prepared according to the general procedure B, the reaction progress was monitored by HPLC **method B**, conversion: 87%. The product was characterized by ESI-MS ($[M+H]^+$ obs. 654.33, calc. 654.33). The results are shown in Fig. S26. * is a oxidation side-product from **4** and **4'** is the dimer of **4**.

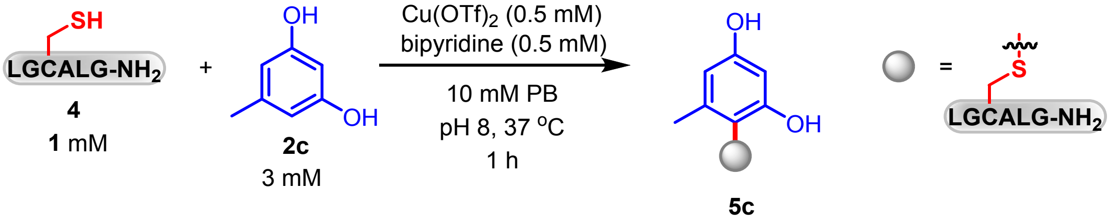

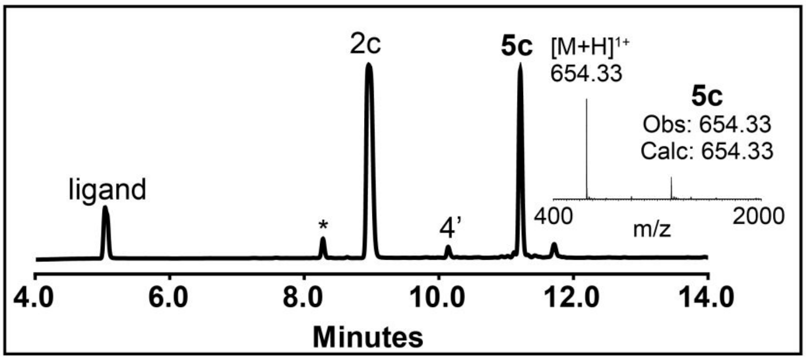

Figure S26. The modification of model peptide **4** with **2c**.

*18. The modification of model peptide **4** with **2d**.*

(5d): Prepared according to the general procedure B, the reaction progress was monitored by HPLC **method B**, conversion: 97%. The product was characterized by ESI-MS ($[M+H]^+$ obs. 670.17, calc. 670.32). The results are shown in Fig. S27. * is a oxidation side-product from **4** and **4'** is the dimer of **4**.

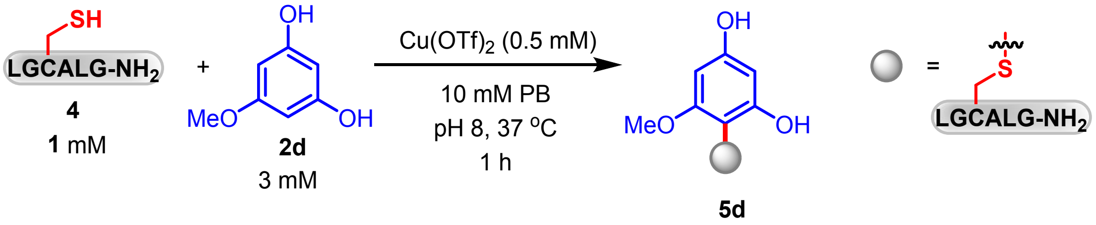

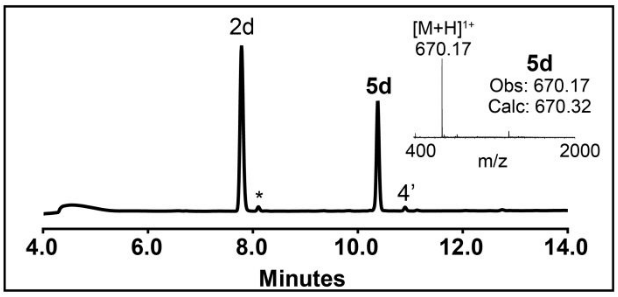

Figure S27. The modification of model peptide **4** with **2d**.

*19. The modification of model peptide **4** with **2e**.*

(5e): Prepared according to the general procedure B, the reaction progress was monitored by HPLC **method B**, conversion: 92%. The product was characterized by ESI-MS ($[M+H]^+$ obs. 656.25, calc. 656.31). The results are shown in Fig. S28. **4'** is the dimer of **4**.

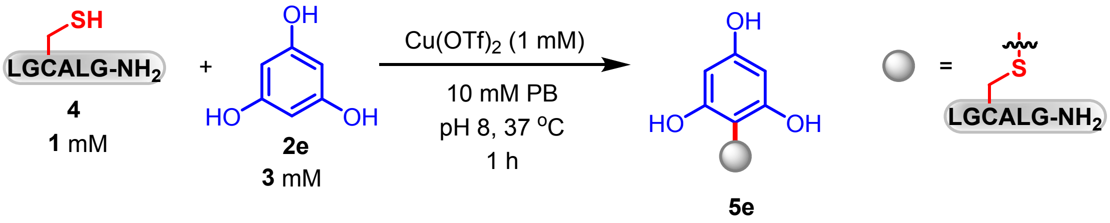

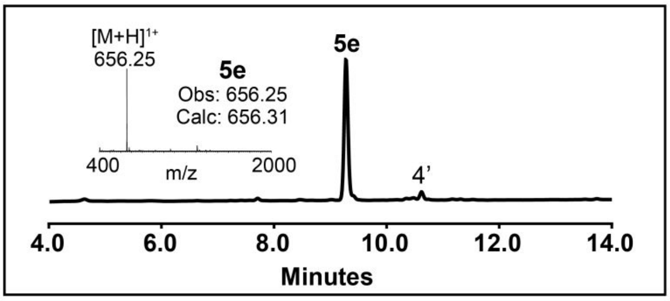

Figure S28. The modification of model peptide **4** with **2e**.

*20. The modification of model peptide **4** with **2f**.*

(5f): Prepared according to the general procedure B, the reaction progress was monitored by HPLC **method B**, conversion: 99%. The product was characterized by ESI-MS ($[M+H]^+$ obs. 684.25, calc. 684.30). The results are shown in Fig. S29. * is a oxidation side-product from **4** and **4'** is the dimer of **4**.

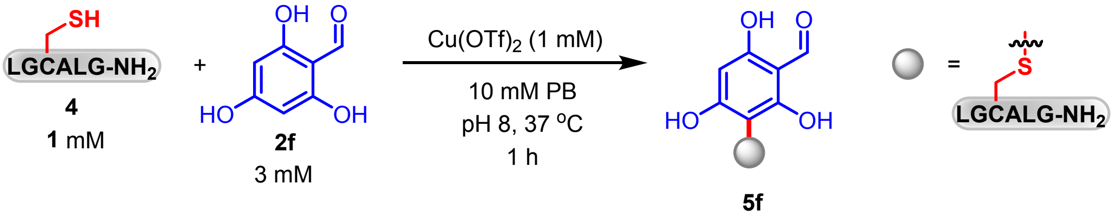

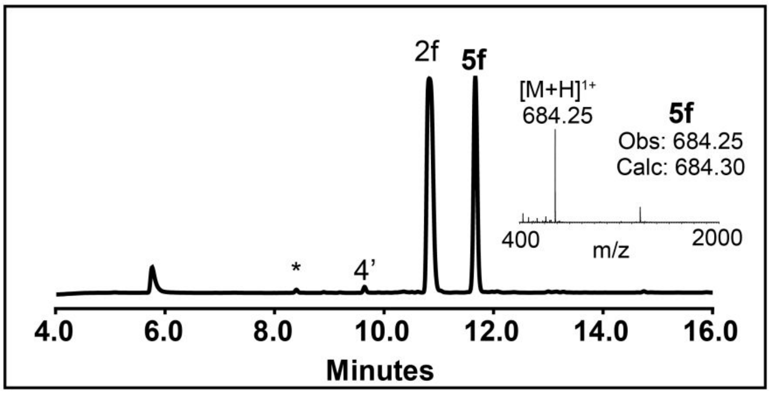

Figure S29. The modification of model peptide **4** with **2f**.

*21. The modification of model peptide **4** with **2g**.*

(5g): Prepared according to the general procedure B, the reaction progress was monitored by HPLC **method B**, conversion: 60%. The product was characterized by ESI-MS ($[M+H]^+$ obs. 674.25, calc. 674.33). The results are shown in Fig. S30. * is a oxidation side-product from **4**.

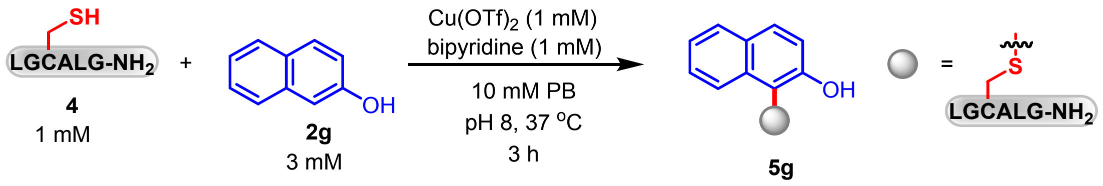

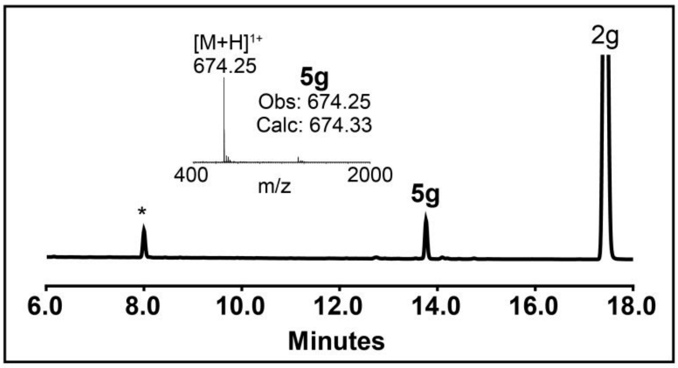

Figure S30. The modification of model peptide **4** with **2g**.

*22. The modification of model peptide **4** with **2h**.*

(5h): Prepared according to the general procedure B, conducting the reaction with degassed solution, the reaction progress was monitored by HPLC **method B**, conversion: 71% (**5h**:**5h*** 62:9). The product was characterized by ESI-MS ($[M+H]^+$ obs. 639.17, calc. 639.33). **5h*** inferred to be another conjugate at a different position on benzene ring, based on its identical mass to **5h**. The results are shown in Fig. S31. * is a oxidation side-product from **4**.

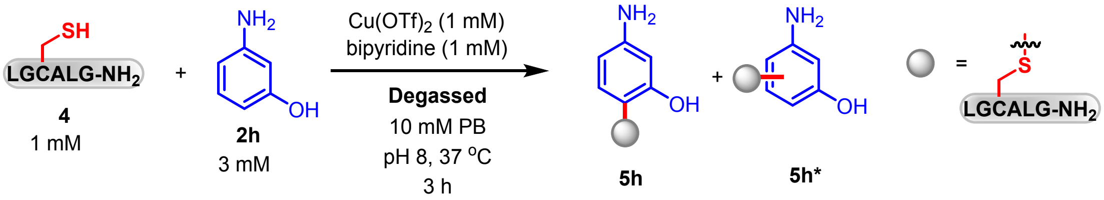

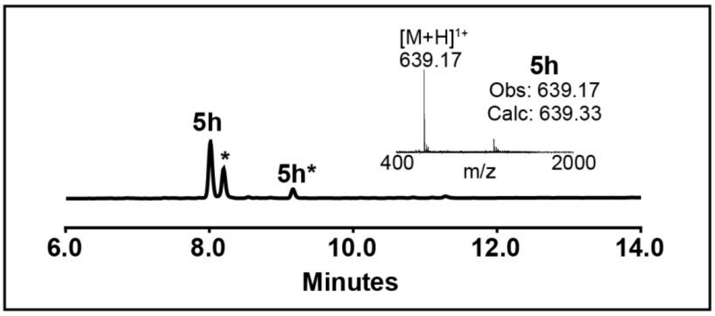

Figure S31. The modification of model peptide **4** with **2h**.

*23. The modification of model peptide **4** with **2i**.*

(5i): Prepared according to the general procedure B, the reaction progress was monitored by HPLC **method B**, conversion: 73% (**5i**:**5i*** 67:6). The product was characterized by ESI-MS ($[M+H]^+$ obs. 667.25, calc. 667.36). **5i*** is inferred to be another conjugate at a different position on benzene ring, based on its identical mass to **5i.** The results are shown in Fig. S32. * is a oxidation side-product from **4**.

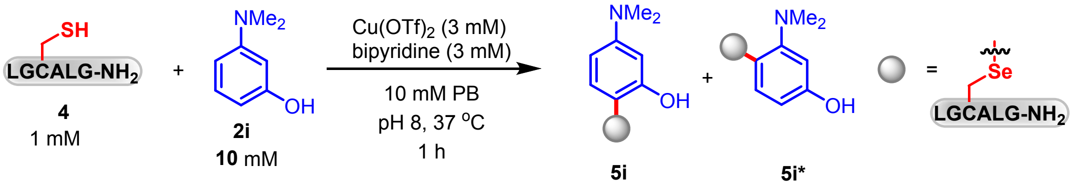

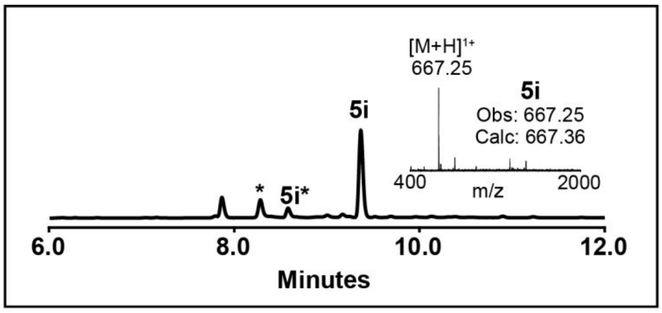

Figure S32. The modification of model peptide **4** with **2i**.

*24. The modification of model peptide **4** with **2j**.*

(5j): Prepared according to the general procedure B, the reaction progress was monitored by HPLC **method B**. There is no functionalization of peptide **4**. The results are shown in Fig. S33. * is a oxidation side-product from **4** and **4'** is the dimer of **4**.

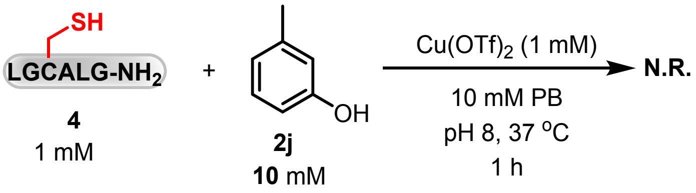

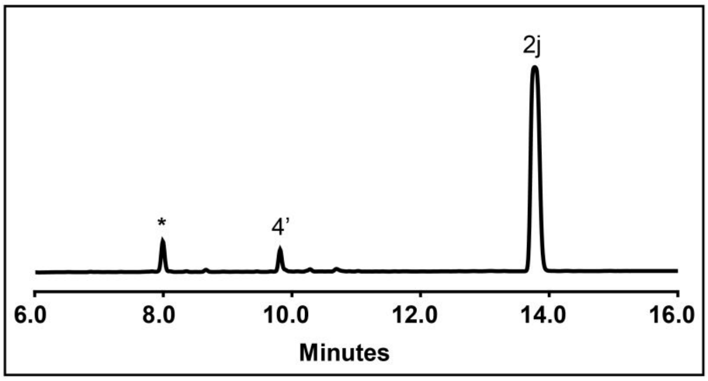

Figure S33. The modification of model peptide **4** with **2j**.

*25. The modification of model peptide **4** with **2k**.*

(5k): Prepared according to the general procedure B, the reaction progress was monitored by HPLC **method B**. There is no functionalization of peptide **4**. The results are shown in Fig. S34. * is a oxidation side-product from **4**.

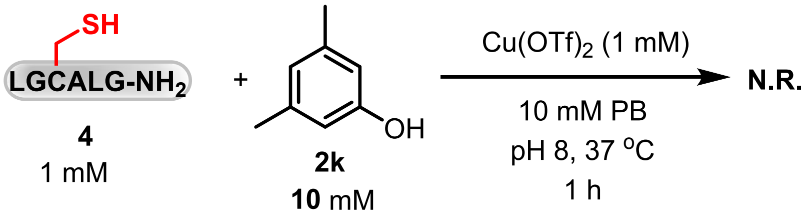

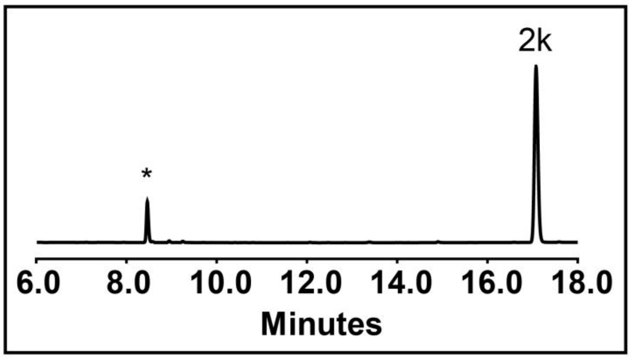

Figure S34. The modification of model peptide **4** with **2k**.

*26. The modification of model peptide **4** with **2l**.*

(5l): Prepared according to the general procedure B, the reaction progress was monitored by HPLC **method B**. There is no functionalization of peptide **4**. The results are shown in Fig. S35. * is a oxidation side-product from **4** and **4'** is the dimer of **4**.

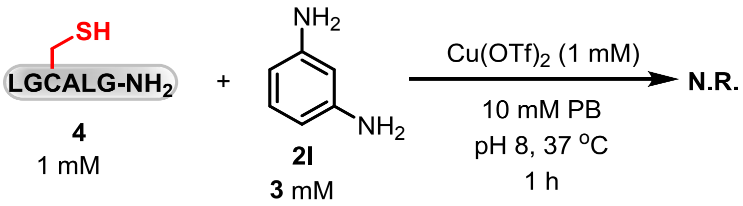

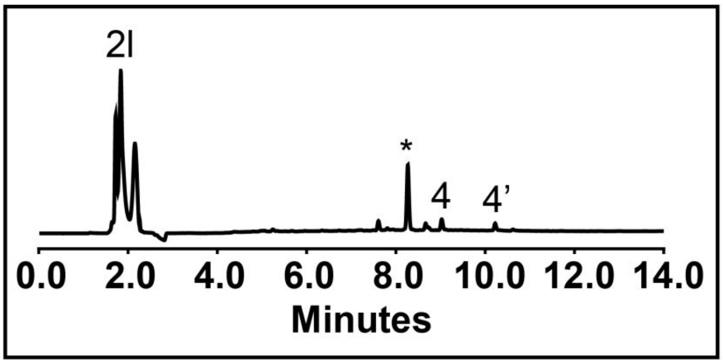

Figure S35. The modification of model peptide **4** with **2l**.

*27. The modification of model peptide **1** with **6a**.*

(7a): Prepared according to the general procedure A, the reaction progress was monitored by HPLC **method A**, conversion: 73%. The product was characterized by ESI-MS ([M+H]$^+$ obs. 659.08, calc. 659.28). The results are shown in Fig. S36.

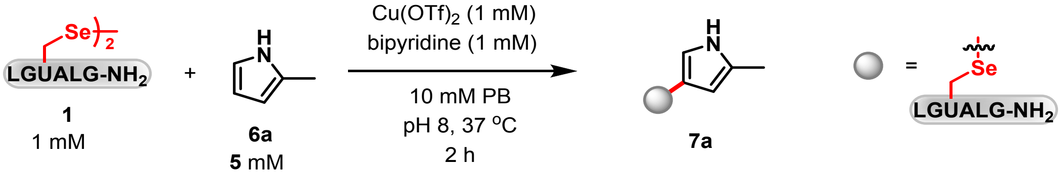

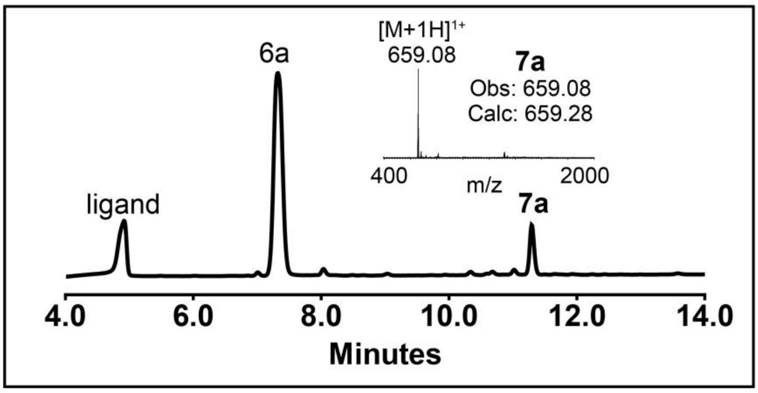

Figure S36. The modification of model peptide **1** with **6a**.

*28. The modification of model peptide **1** with **6b**.*

(7b): Prepared according to the general procedure A, the reaction progress was monitored by HPLC **method A**, conversion: 82%. The product was characterized by ESI-MS ([M+H]$^+$ obs. 673.08, calc. 673.29). The results are shown in Fig. S37.

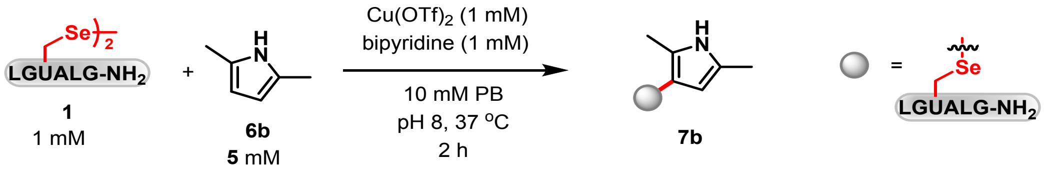

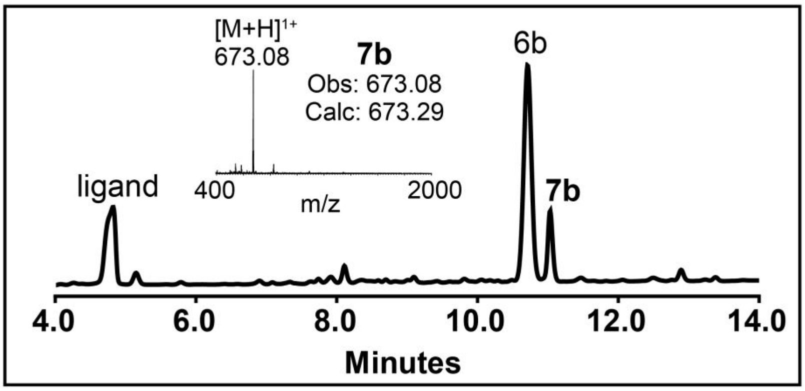

Figure S37. The modification of model peptide **1** with **6b**.

*29. The modification of model peptide **1** with **6c**.*

(7c): Prepared according to the general procedure A, the reaction progress was monitored by HPLC **method A**, conversion: 93%. The product was characterized by ESI-MS ($[M+H]^+$ obs. 695.08, calc. 695.28) and NMR (Fig. S129-S130). The results are shown in Fig. S38.

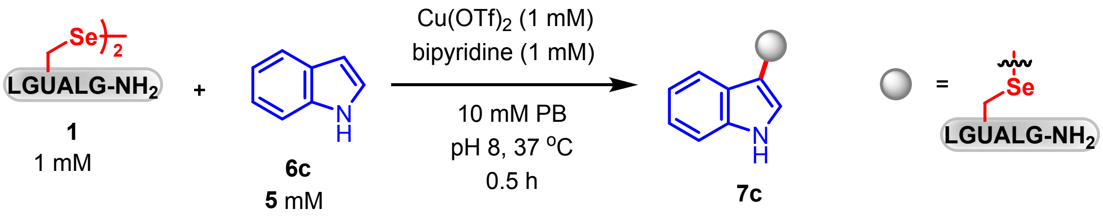

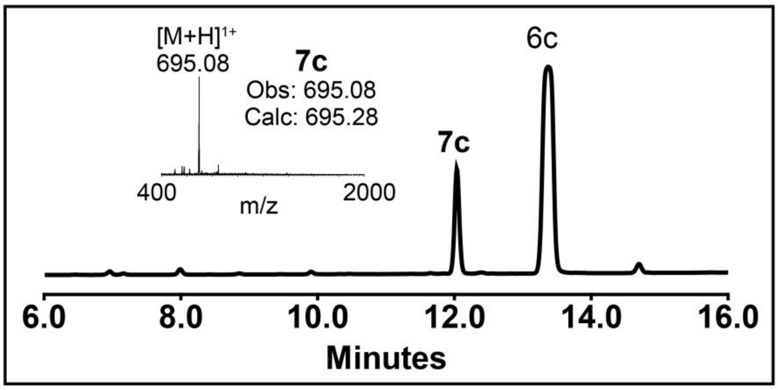

Figure S38. The modification of model peptide **1** with **6c**.

*30. The modification of model peptide **1** with **6d**.*

(7d): Prepared according to the general procedure A, the reaction progress was monitored by HPLC **method A**, conversion: 94%. The product was characterized by ESI-MS ($[M+H]^+$ obs. 709.08, calc. 709.29) and NMR (Fig. S131). The results are shown in Fig. S39.

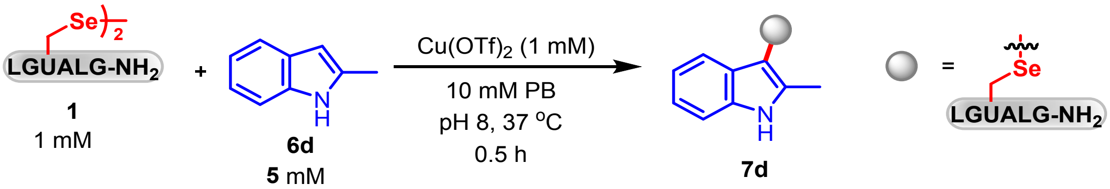

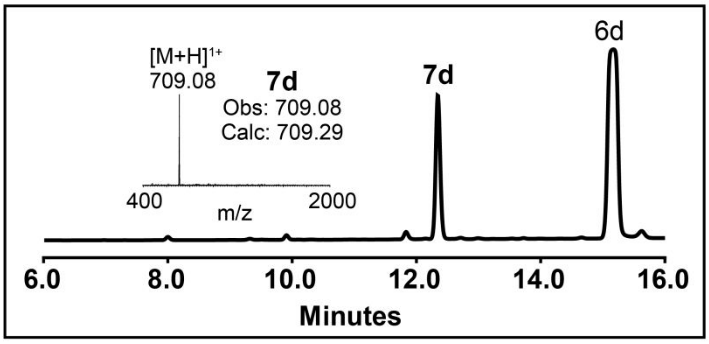

Figure S39. The modification of model peptide **1** with **6d**.

*31. The modification of model peptide **1** with **6e**.*

(7e): Prepared according to the general procedure A, the reaction progress was monitored by HPLC **method A**, conversion: 98% (**7e**:**7e*** 84:14). The product was characterized by ESI-MS ([M+H]$^+$ obs. 711.25, calc. 711.27). The results are shown in Fig. S40. **7e*** is inferred to be another conjugate at a different position on indole, based on its identical mass to **7e**.

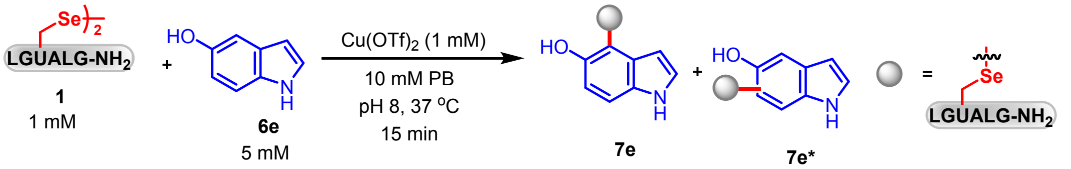

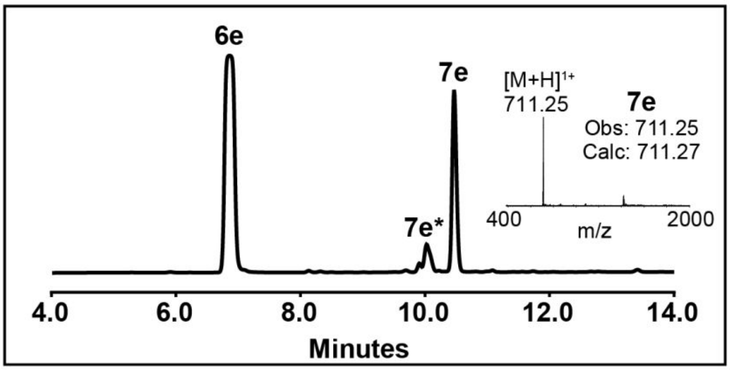

Figure S40. The modification of model peptide **1** with **6e**.

*32. The modification of model peptide **1** with **6f**.*

(7f): Prepared according to the general procedure A, the reaction progress was monitored by HPLC **method A**, conversion: 99%. The product was characterized by ESI-MS ([M+H]$^+$ obs. 739.25, calc. 739.27). The results are shown in Fig. S41.

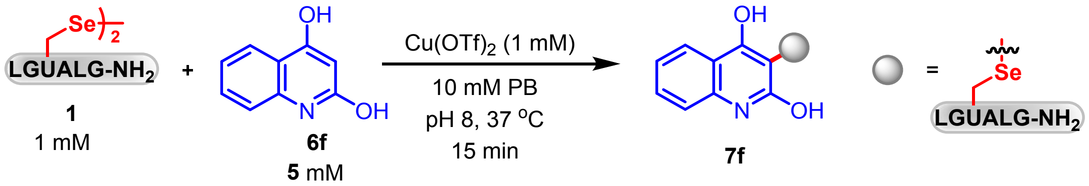

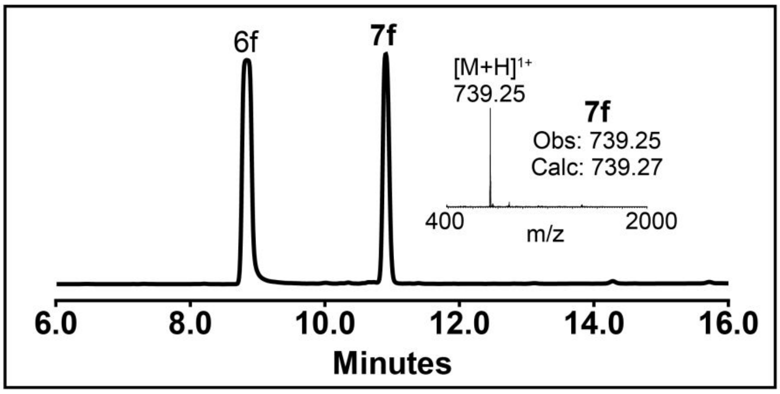

Figure S41. The modification of model peptide **1** with **6f**.

*33. The modification of model peptide **1** with **6g**.*

(7g): Prepared according to the general procedure A, the reaction progress was monitored by HPLC **method A**, conversion: 99%. The product was characterized by ESI-MS ($[M+H]^+$ obs. 740.25, calc. 740.25) and NMR (Fig. S132). The results are shown in Fig. S42.

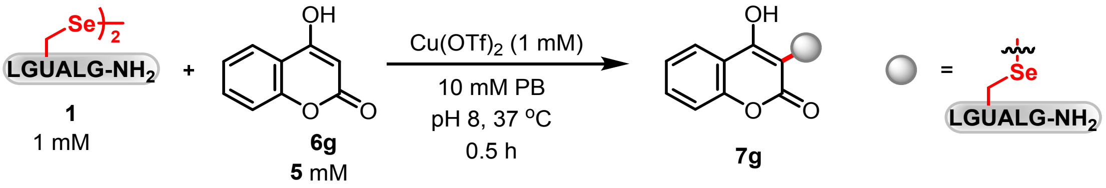

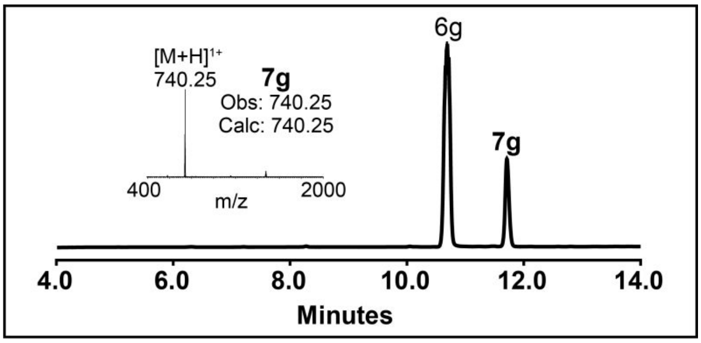

Figure S42. The modification of model peptide **1** with **6g**.

*34. The modification of model peptide **1** with **6h**.*

(7h): Prepared according to the general procedure A, the reaction progress was monitored by HPLC **method A**, conversion: 56%. The product was characterized by ESI-MS ($[M+H]^+$ obs. 687.17, calc. 687.28) and NMR (Fig. S133-S134). The results are shown in Fig. S43. * is a oxidation side-product from **1**.

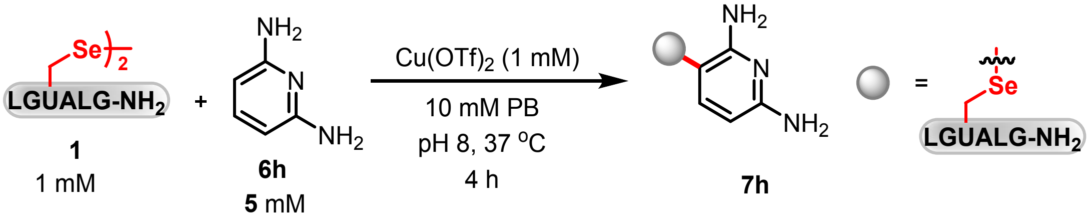

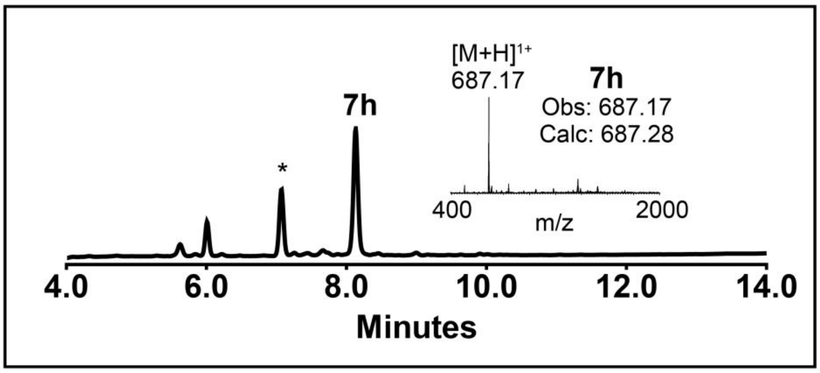

Figure S43. The modification of model peptide **1** with **6h**.

*35. The modification of model peptide **4** with **6a**.*

(6a): Prepared according to the general procedure B, the reaction progress was monitored by HPLC **method B**. There is no functionalization of peptide **4**. The results are shown in Fig. S44. * is a oxidation side-product from **4**.

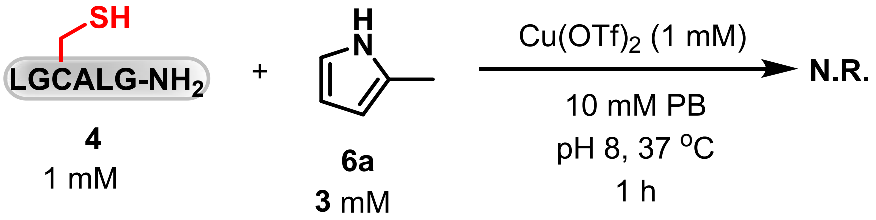

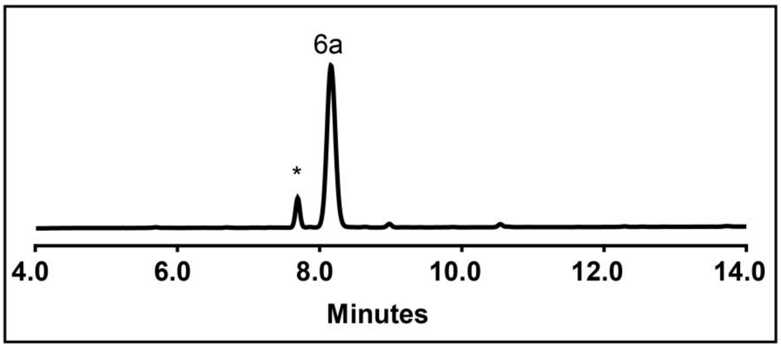

Figure S44. The modification of model peptide **4** with **6a**.

*36. The modification of model peptide **4** with **6b**.*

(6b): Prepared according to the general procedure B, the reaction progress was monitored by HPLC **method B**. There is no functionalization of peptide **4**. The results are shown in Fig. S45. * is a oxidation side-product from **4**.

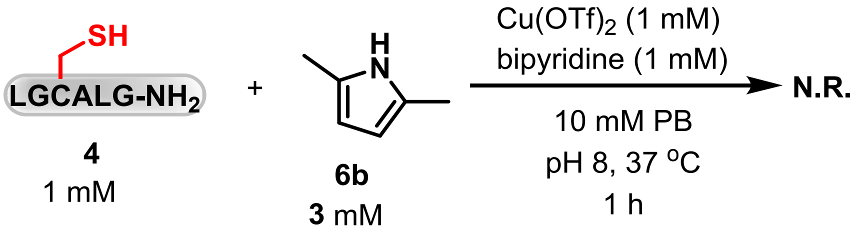

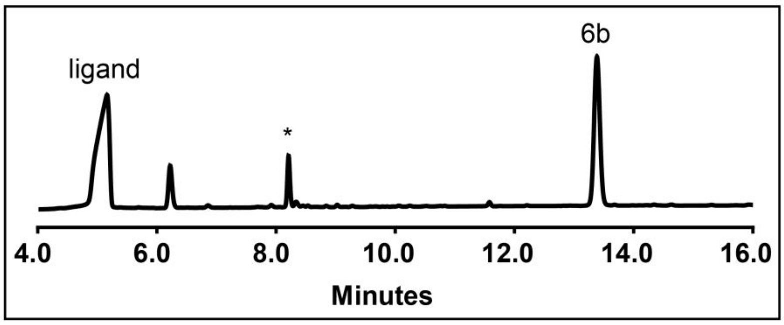

Figure S45. The modification of model peptide **4** with **6b**.

*37. The modification of model peptide **4** with **6c**.*

(8c): Prepared according to the general procedure B, conducting the reaction with degassed solution, the reaction progress was monitored by HPLC **method B**, conversion: 50%. The product was characterized by ESI-MS ([M+H]$^+$ obs. 647.25, calc. 647.33). The results are shown in Fig. S46. * is a oxidation side-product from **4**.

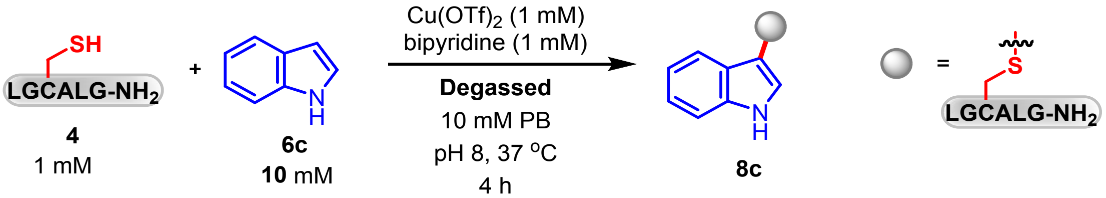

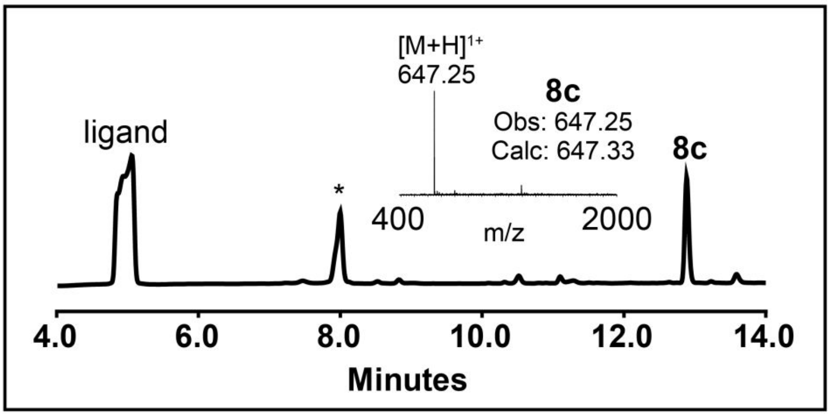

Figure S46. The modification of model peptide **4** with **6c**.

*38. The modification of model peptide **4** with **6d**.*

(8d): Prepared according to the general procedure B, the reaction progress was monitored by HPLC **method B**, conversion: 76%. The product was characterized by ESI-MS ([M+H]$^+$ obs. 661.17, calc. 661.35). The results are shown in Fig. S47. * is a oxidation side-product from **4**.

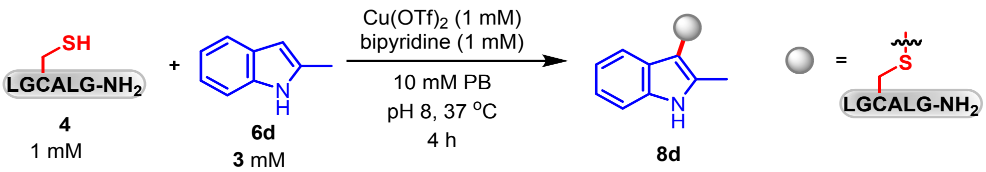

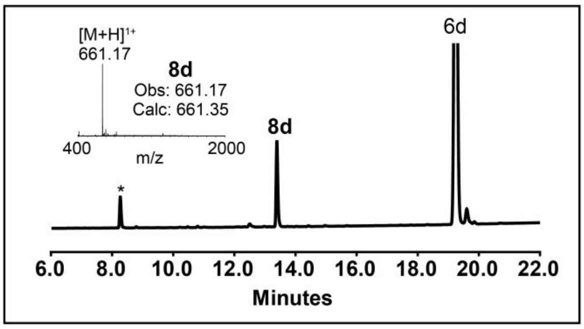

Figure S47. The modification of model peptide **4** with **6d**.

*39. The modification of model peptide **4** with **6e**.*

(8e): Prepared according to the general procedure B, the reaction progress was monitored by HPLC **method B**, conversion: 81%. The product was characterized by ESI-MS ($[M+H]^+$ obs. 663.25, calc. 663.33). The results are shown in Fig. S48. * is a oxidation side-product from **4**, & is a side-product from **6e**.

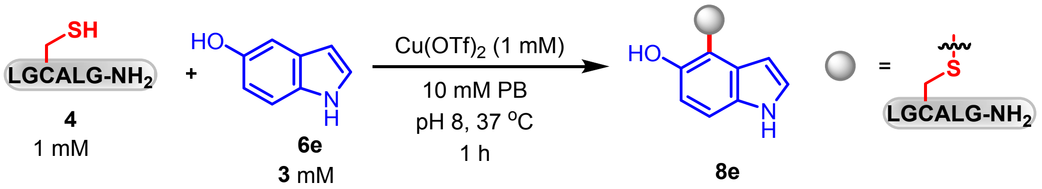

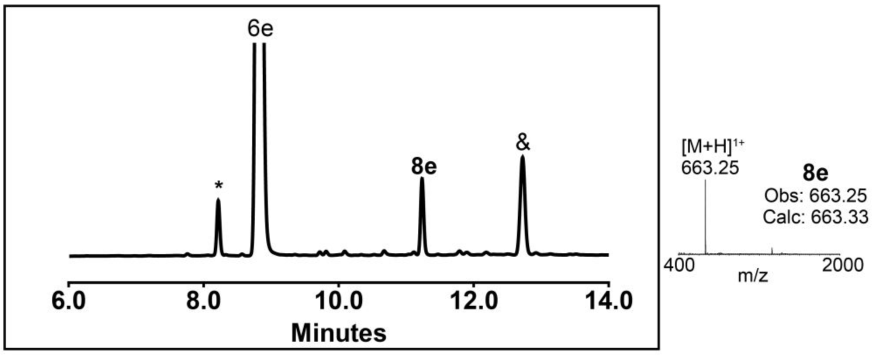

Figure S48. The modification of model peptide **4** with **6e**.

*40. The modification of model peptide **4** with **6f**.*

(6f): Prepared according to the general procedure B, conducting the reaction with degassed solution, the reaction progress was monitored by HPLC **method B**, conversion: 60%. The product was characterized by ESI-MS ($[M+H]^+$ obs. 691.17, calc. 691.32). The results are shown in Fig. S49. * is a oxidation side-product from **4**.

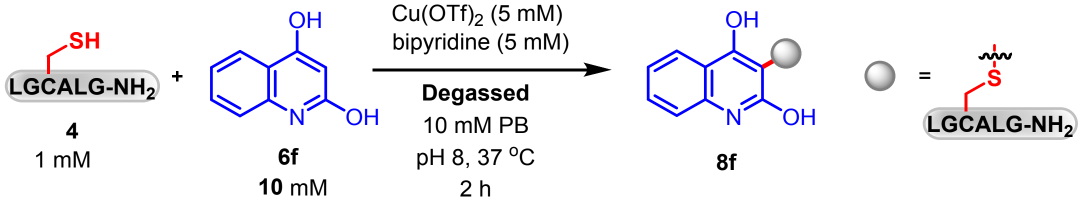

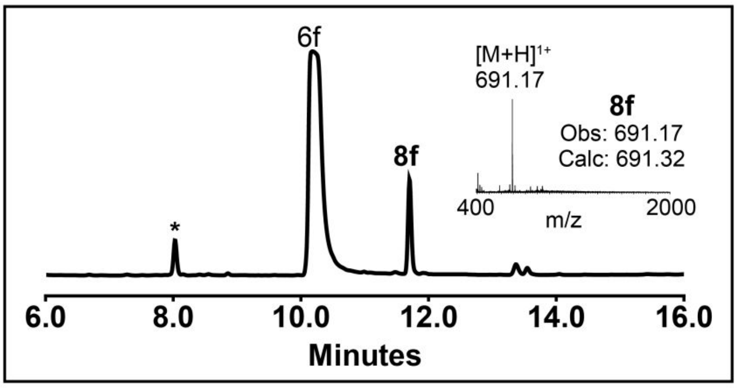

Figure S49. The modification of model peptide **4** with **6f**.

*41. The modification of model peptide **4** with **6g**.*

(6g): Prepared according to the general procedure B, the reaction progress was monitored by HPLC **method B**. There is no functionalization of peptide **4**. The results are shown in Fig. S50. * is a oxidation side-product from **4** and **4'** is the dimer of **4**.

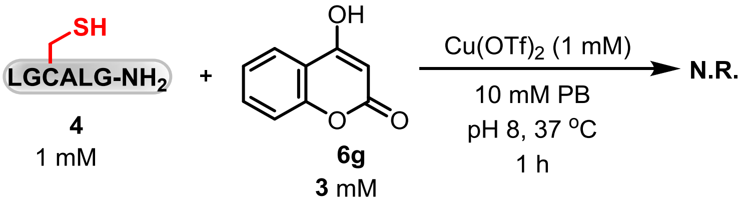

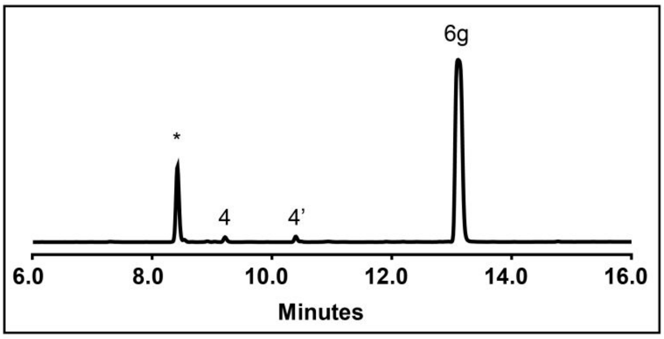

Figure S50. The modification of model peptide **4** with **6g**.

*42. The modification of model peptide **4** with **6h**.*

(6h): Prepared according to the general procedure B, the reaction progress was monitored by HPLC **method B**. There is no functionalization of peptide **4**. The results are shown in Fig. S51. * is a oxidation side-product from **4** and **4'** is the dimer of **4**.

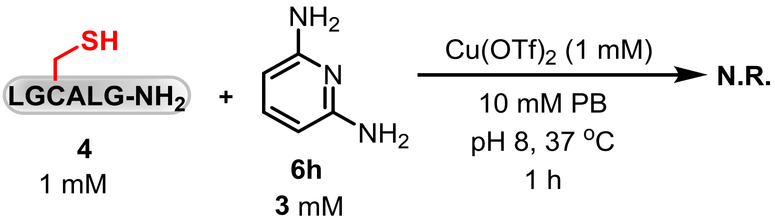

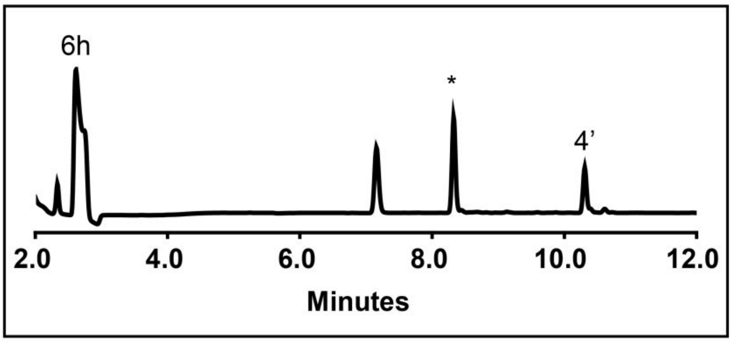

Figure S51. The modification of model peptide **4** with **6h**.

*43. The modification of model peptide **1** with **9a**.*

($10a_1$): Prepared according to the general procedure A, the reaction progress was monitored by HPLC **method A**, conversion: 99% (**$10a_1$:$10a_2$** 82:17). **$10a_1$** was characterized by ESI-MS ($[M+H]^+$ obs. 806.25, calc. 806.30) and NMR (Fig. S135). The results are shown in Fig. S52. The structure of **$10a_2$** is inferred from the MS data and plausible steric hindrance, further characterization is not possible due to low conversion.

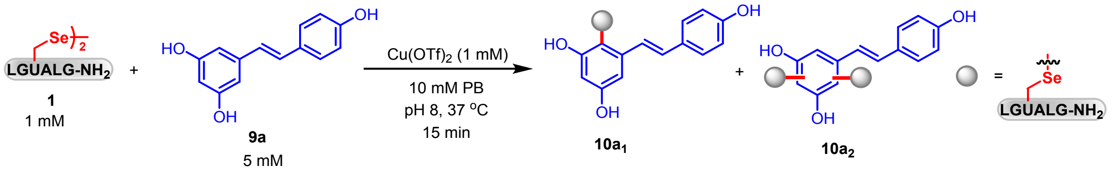

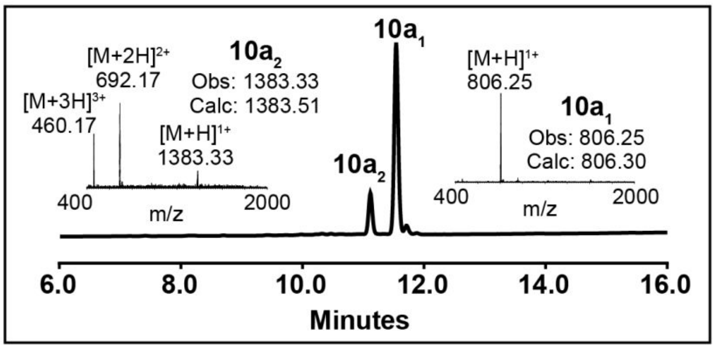

Figure S52. The modification of model peptide **1** with **9a**.

*44. The modification of model peptide **1** with **9b**.*

($10b_1$): Prepared according to the general procedure A, the reaction progress was monitored by HPLC **method A**, conversion: 99% (**$10b_1$:$10b_2$** 93:7). **$10b_1$** was characterized by ESI-MS ($[M+H]^+$ obs. 1425.17, calc. 1425.49) and NMR (Fig. S136-S138). The results are shown in Fig. S53. The structure of **$10b_2$** is inferred from the MS data and plausible steric hindrance, further characterization is not possible due to low conversion.

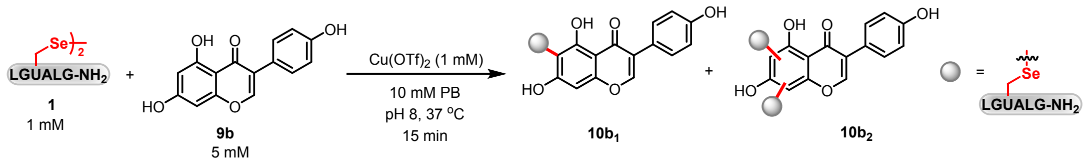

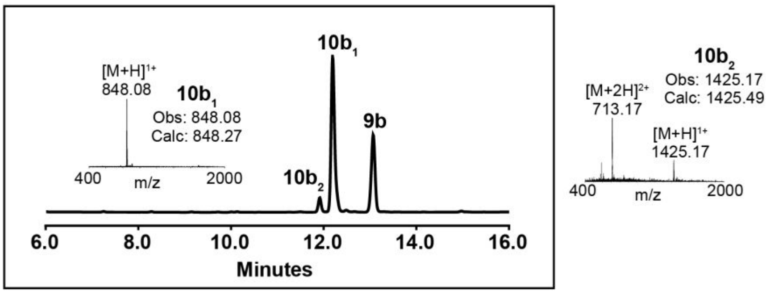

Figure S53. The modification of model peptide **1** with **9b**.

*45. The modification of model peptide **1** with **9c**.*

(10c): Prepared according to the general procedure A, the reaction progress was monitored by HPLC **method A**, conversion: 90%. The product was characterized by ESI-MS ($[M+H]^+$ obs. 930.25, calc. 930.33). The results are shown in Fig. S54.

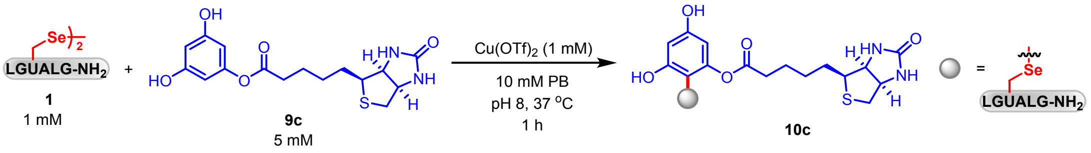

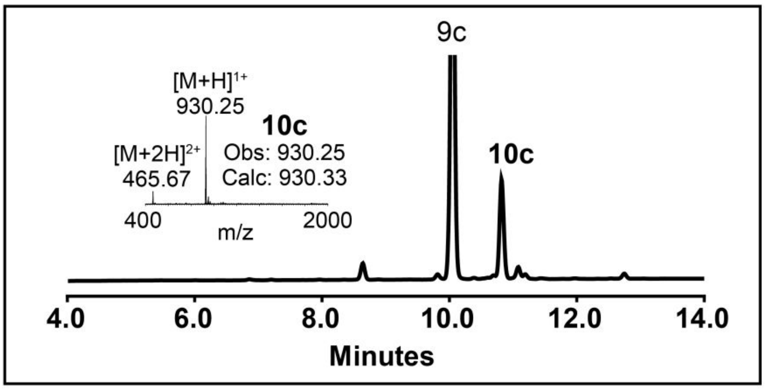

Figure S54. The modification of model peptide **1** with **9c**.

*46. The modification of model peptide **1** with **9d**.*

($10d_1$): Vancomycin **9d** underwent purification using a preparative C18 column prior to being used in the process. Prepared according to the general procedure **A**, the reaction progress was monitored by HPLC **method A**, conversion: 90% (**$10d_1$**:**$10d_2$** 67:33). **$10d_1$** was characterized by ESI-MS (mass obs. 2026.17 Da, calc. 2025.83 Da) and NMR (Fig. S139-S141). **$10d_2$** was characterized by ESI-MS (mass obs. 2602.29 Da, calc. 2602.40 Da). The results are shown in Fig. S55.

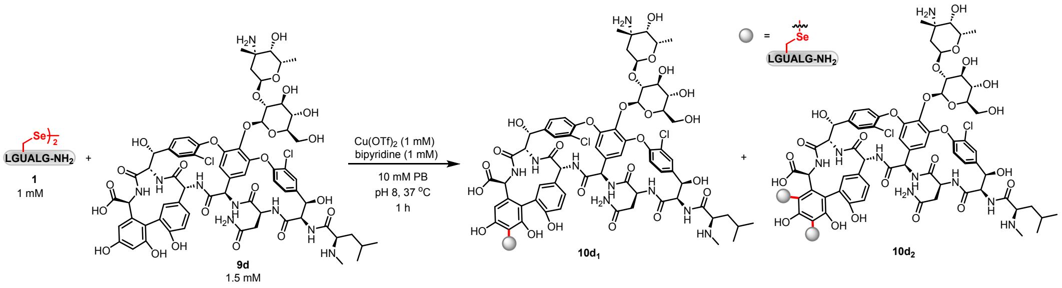

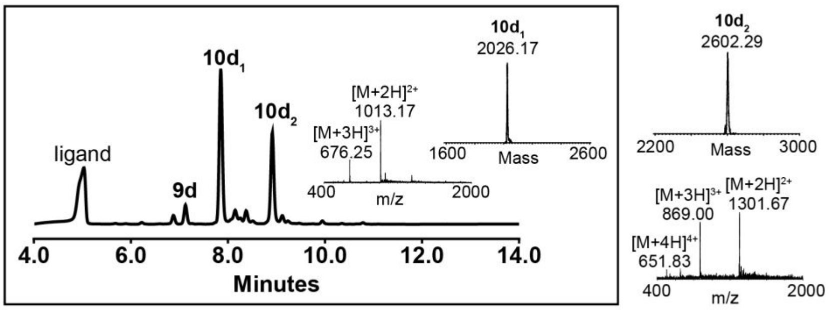

Figure S55. The modification of model peptide **1** with **9d**.

*47. The modification of model peptide **1** with **9e**.*

(10e): Prepared according to the general procedure A, the reaction progress was monitored by HPLC **method A**, conversion: 97%. The product was characterized by ESI-MS ($[M+H]^+$ obs. 754.25, calc. 754.31) and NMR (Fig. S142). The results are shown in Fig. S56.

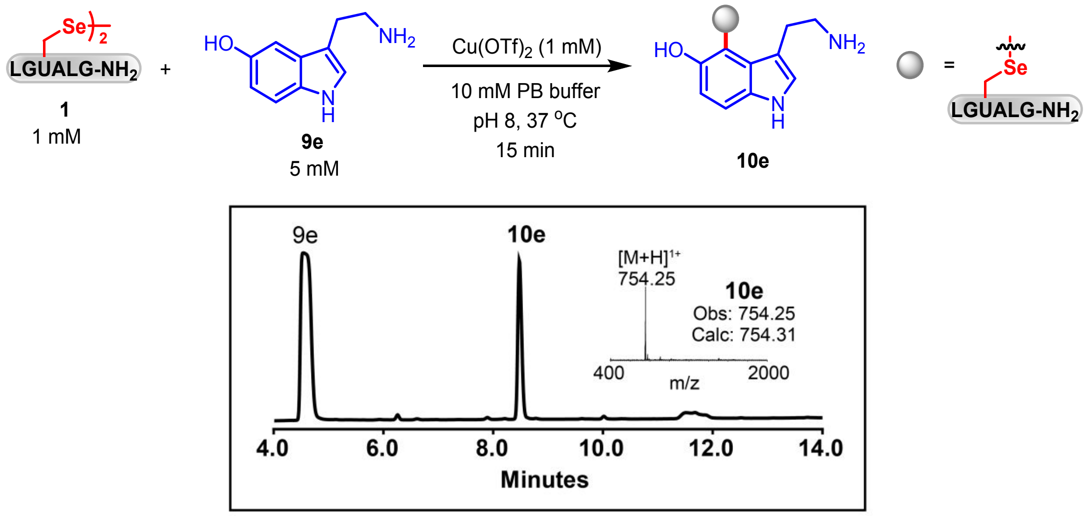

Figure S56. The modification of model peptide **1** with **9e**.

*48. The modification of model peptide **1** with **9f**.*

(10f): Prepared according to the general procedure A, the reaction progress was monitored by HPLC **method A,** conversion: 97% (**10f**:**10f*** 77:20). **10f** was characterized by ESI-MS ($[M+H]^+$ obs. 776.33, calc. 776.30) and NMR (Fig. S143-S144). **10f*** is inferred to be another conjugate at a different position on indole, based on its identical mass to **10f**. The results are shown in Fig. S57.

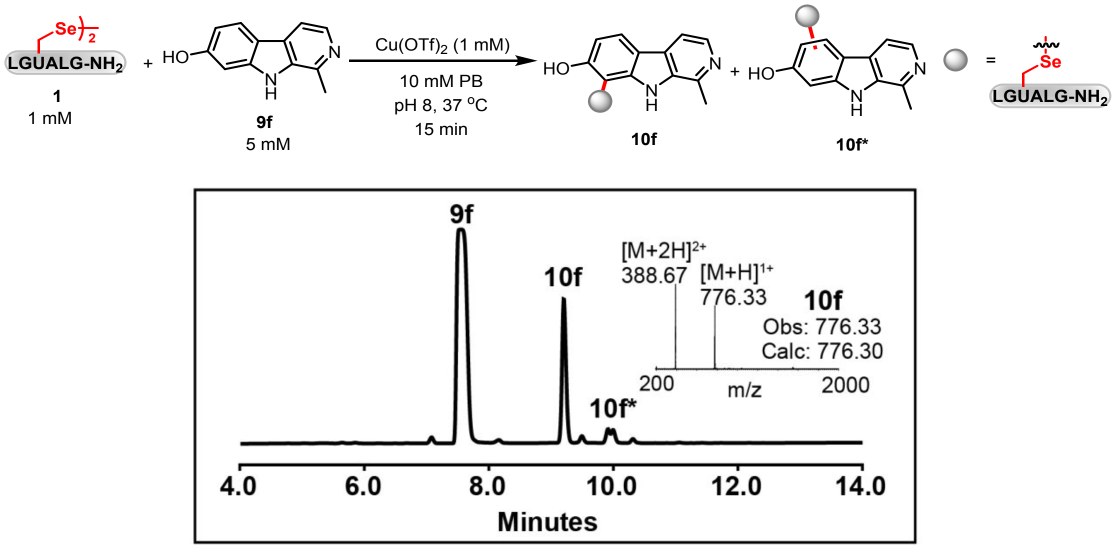

Figure S57. The modification of model peptide **1** with **9f**.

*49. The modification of model peptide **1** with **9g**.*

(10g): Prepared according to the general procedure A, the reaction progress was monitored by HPLC **method D**, conversion: 94%. The product was characterized by ESI-MS ($[M+H]^+$ obs. 859.42, calc. 859.37). The results are shown in Fig. S58.

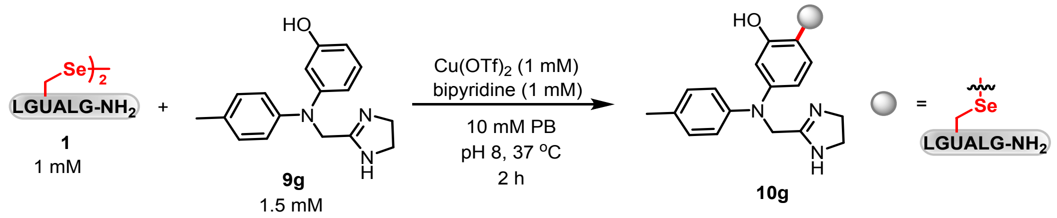

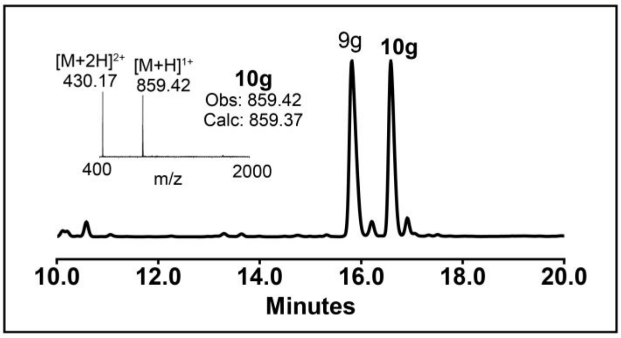

Figure S58. The modification of model peptide **1** with **9g**.

*50. The modification of model peptide **4** with **9a**.*

(11a): Prepared according to the general procedure B, the reaction progress was monitored by HPLC **method B**, conversion: 90%. The product was characterized by ESI-MS ($[M+H]^+$ obs. 758.33, calc. 758.35). The results are shown in Fig. S59. * is a oxidation side-product from **4**.

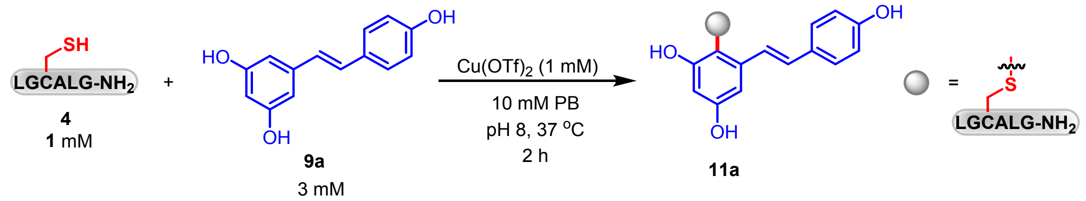

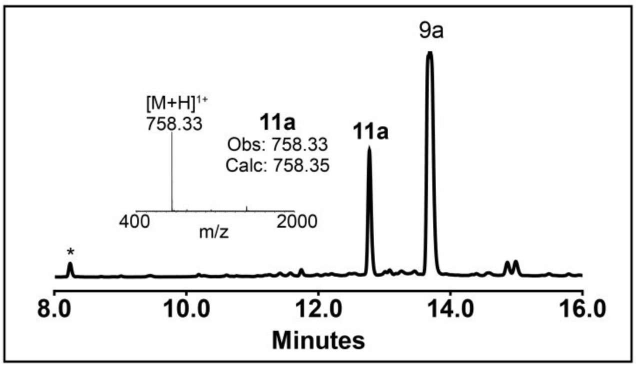

Figure S59. The modification of model peptide **4** with **9a**.

*51. The modification of model peptide **4** with **9b**.*

(9b): Prepared according to the general procedure B, the reaction progress was monitored by HPLC **method B**. There is no functionalization of peptide **4**. The results are shown in Fig. S60. * is a oxidation side-product from **4** and **4’** is the dimer of **4**.

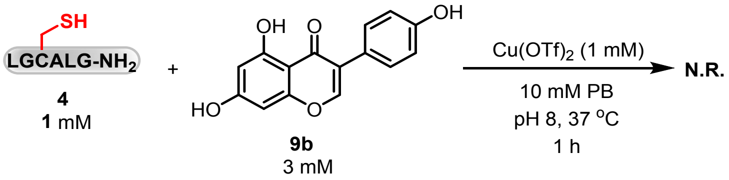

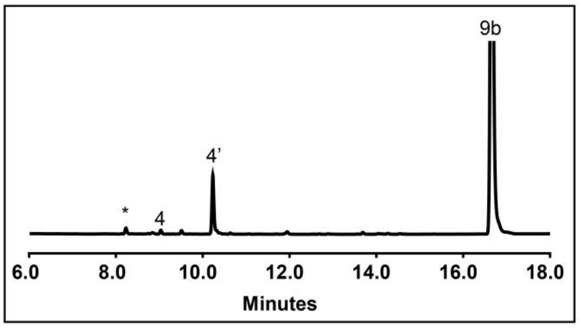

Figure S60. The modification of model peptide **4** with **9b**.

*52. The modification of model peptide **4** with **9c**.*

(11c): Prepared according to the general procedure B, the reaction progress was monitored by HPLC **method B**, conversion: 20%. The product was characterized by ESI-MS ($[M+H]^+$ obs. 882.25, calc. 882.38). The results are shown in Fig. S61. * is a oxidation side-product from **4** and **4’** is the dimer of **4**.

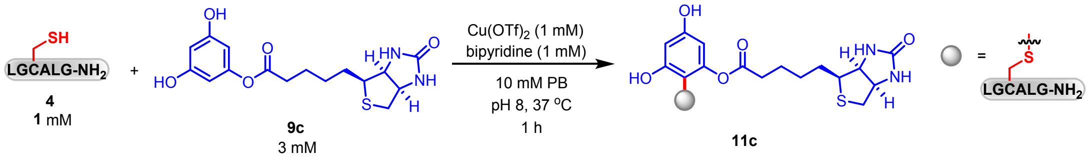

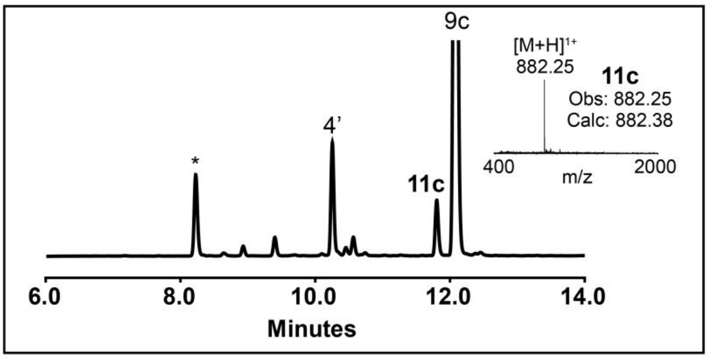

Figure S61. The modification of model peptide **4** with **9c**.

*53. The modification of model peptide **4** with **9d**.*

(9d): Prepared according to the general procedure **B**, the reaction progress was monitored by HPLC **method B**. There is no functionalization of peptide **4**. The results are shown in Fig. S62. **4'** is the dimer of **4**.

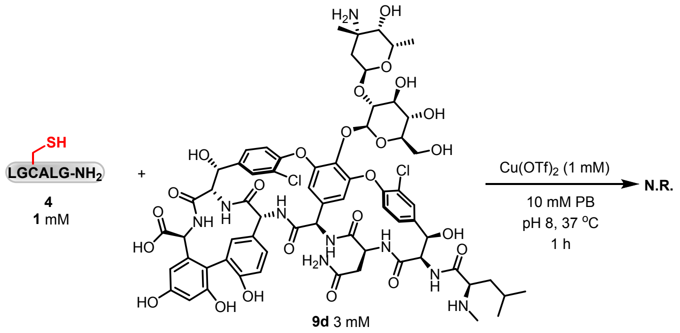

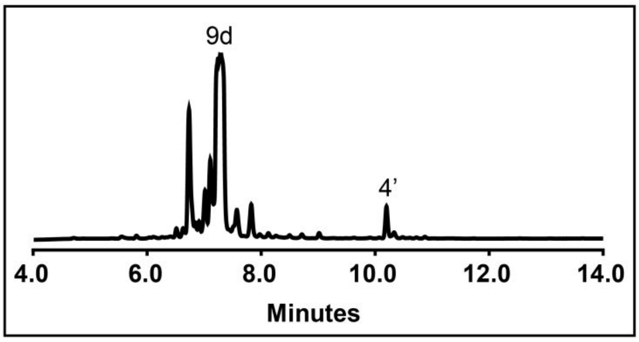

Figure S62. The modification of model peptide **4** with **9d**.

*54. The modification of model peptide **4** with **9e**.*

(11e): Prepared according to the general procedure B, the reaction progress was monitored by HPLC **method B**, conversion: 88%. The product was characterized by ESI-MS ($[M+H]^+$ obs. 706.33, calc. 706.37). The results are shown in Fig. S63. * is a oxidation side-product from **4**.

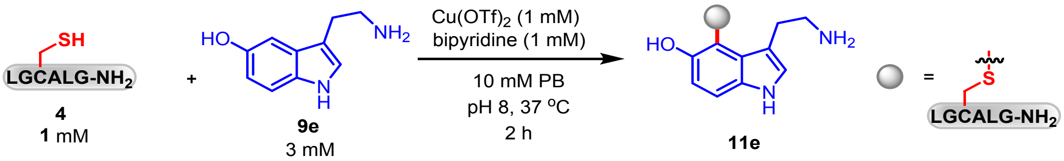

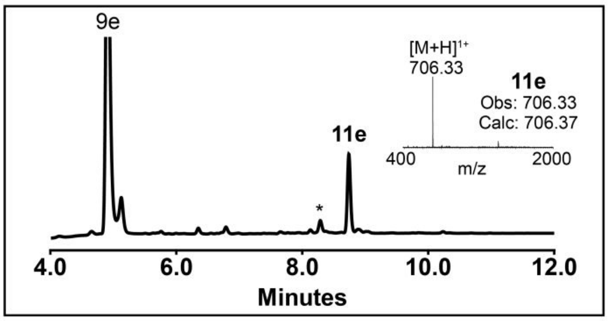

Figure S63. The modification of model peptide **4** with **9e**.

*55. The modification of model peptide **4** with **9f**.*

(9f): Prepared according to the general procedure B, the reaction progress was monitored by HPLC **method B**. There is no functionalization of peptide **4**. The results are shown in Fig. S64. * is a oxidation side-product from **4**.

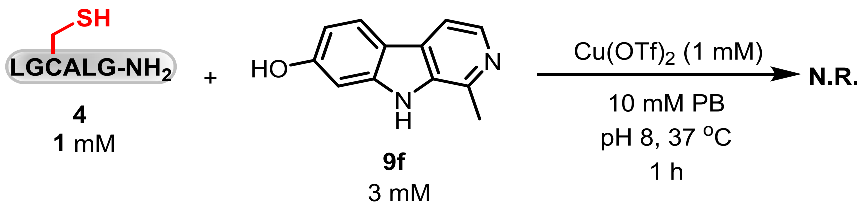

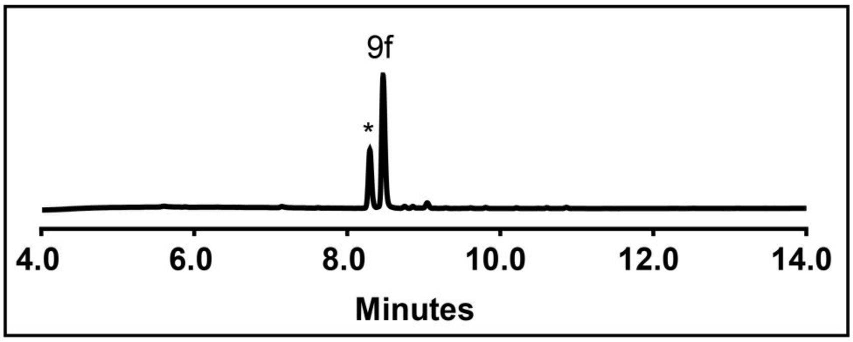

Figure S64. The modification of model peptide **4** with **9f**.

*56. The modification of model peptide **4** with **9g**.*

(9g): Prepared according to the general procedure **B**, the reaction progress was monitored by HPLC **method B**. There is no functionalization of peptide **4**. The results are shown in Fig. S65. * is a oxidation side-product from **4** and **4’** is the dimer of **4**.

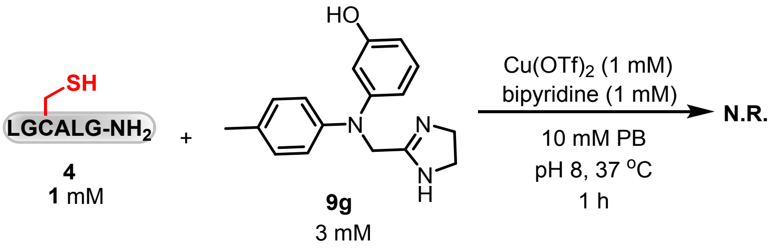

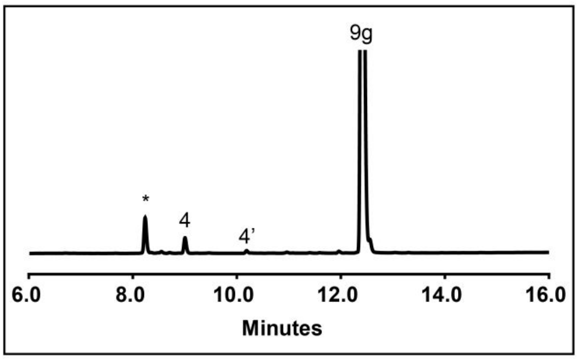

Figure S65. The modification of model peptide **4** with **9g**.

5. Sec/Cys-specific Modification in the Presence of Other Reactive Residues

According to general approach for peptide modification, the stock solution of different peptide (100 mM), **2a** or **2e** (100 mM) and $Cu(OTf)_2$ (100 mM) were prepared for peptide modification.

*1. Negative control reaction of peptide **12** with **2e**.*

Prepared according to the general procedure B, the reaction progress was monitored by HPLC **method B**. There is no functionalization of peptide **12**. The results are shown in Fig. S66.

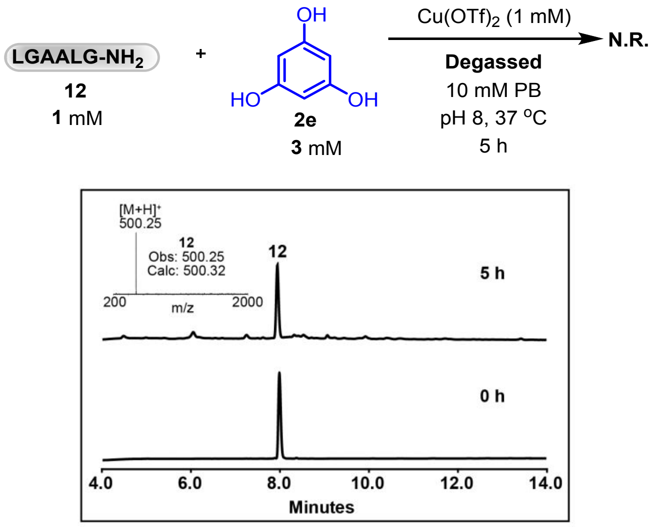

Figure S66. The negative control reaction of peptide **12** with **2e**.

*2. Negative control reaction of peptide **13** with **2e**.*

Prepared according to the general procedure B, conducting the reaction with degassed solution, the reaction progress was monitored by HPLC **method B**. There is no functionalization of peptide **13**. The results are shown in Fig. S67.

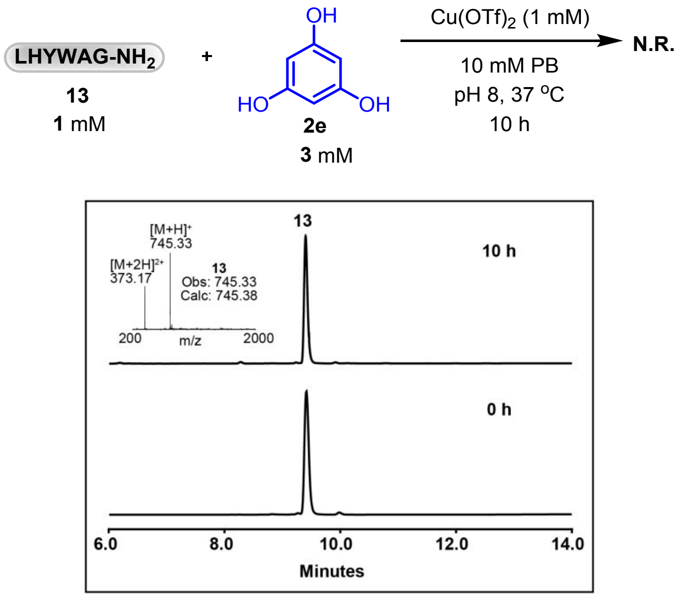

Figure S67. The negative control reaction of peptide **13** with **2e**.

*3. The modification of peptide **14** with **2e**.*

(14e): Prepared according to the general procedure A, the reaction progress was monitored by HPLC **method E**, conversion: 95%. The product was characterized by MALDI ($[M+H]^+$ obs. 1314.15, calc. 1313.48). The results are shown in Fig. S68.

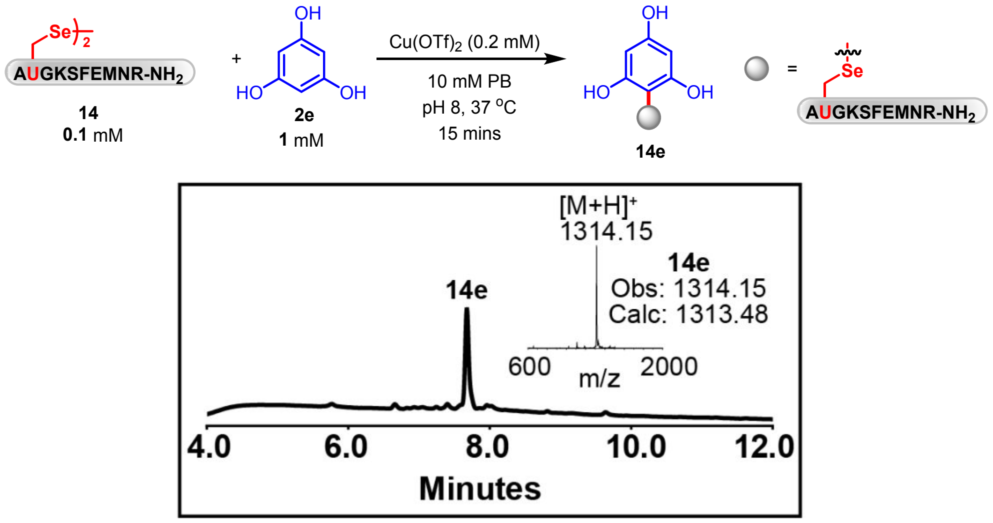

Figure S68. The modification of peptide **14** with **2e**.

*4. The modification of peptide **15** with **2e**.*

(15e): Prepared according to the general procedure B, the reaction progress was monitored by HPLC **method E**, conversion: 95%. The product was characterized by MALDI ($[M+H]^+$ obs. 1267.03, calc. 1266.43). The results are shown in Fig. S69.

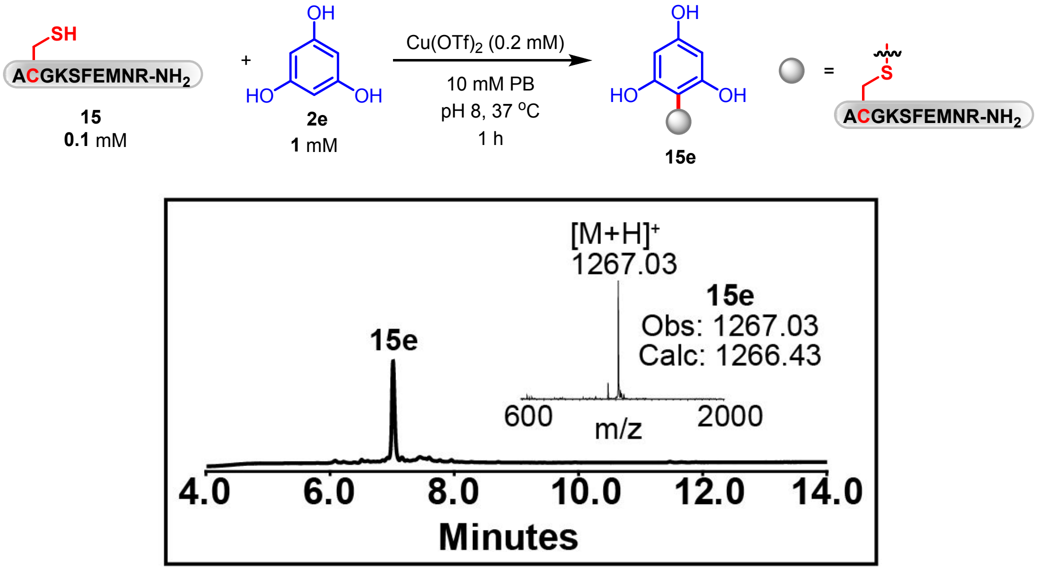

Figure S69. The modification of peptide **15** with **2e**.

5. MS/MS analysis of labelled conjugates ***15e****.*

The purified conjugates ***15e*** was lyophilized, and subsequently subjected to UPLC-MS analysis directly without performing trypsin digestion.

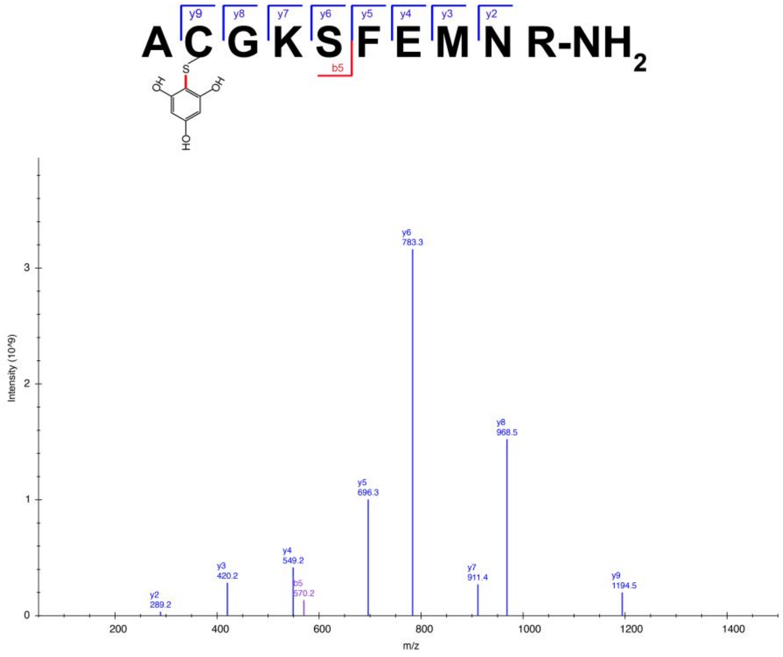

Figure S70. MS/MS spectrum of purified conjugates **15e**.

6. The modification of peptide ***16*** *with* ***2a****.*

($16a_1$ and $16a_2$): Prepared according to general procedure A, the reaction progress was monitored by HPLC **method A**, conversion: 90% (**$16a_1$:$16a_2$** 82:8). The products were characterized by ESI-MS (**$16a_1$**: $[M+H]^+$ obs. 1565.42, calc. 1565.64; **$16a_2$**: mass obs. 3019.52 Da, calc. 3019.08 Da). The results are shown in Fig. S71. # is deselenization form of **16**.

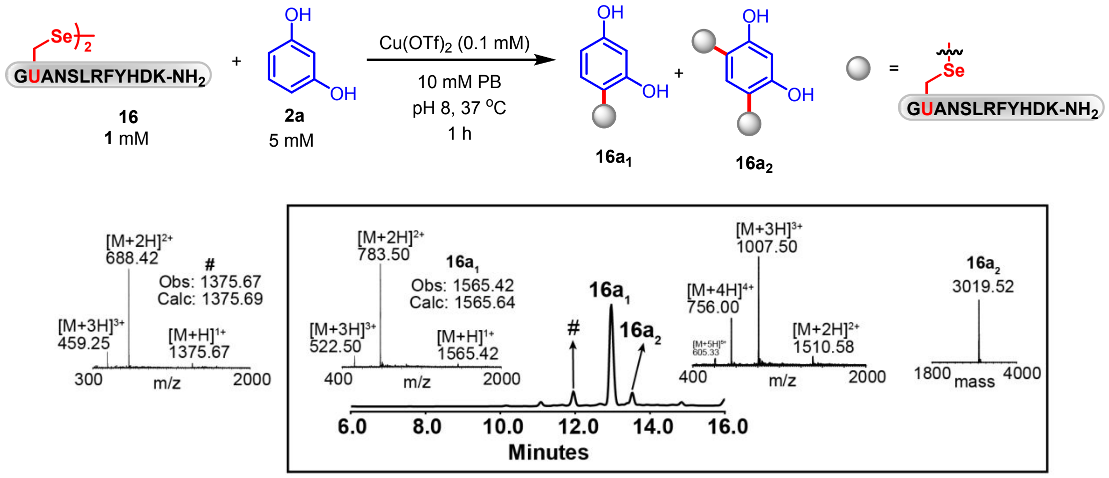

Figure S71. The modification of peptide **16** with **2a**.

*7. The modification of peptide **17** with **2e**.*

($17e_1$ and $17e_2$): Prepared according to the general procedure B, the reaction progress was monitored by HPLC **method C**, conversion: 99% (**$17e_1$:$17e_2$** 83:16). The products were characterized by ESI-MS (**$17e_1$**: $[M+H]^+$ obs. 720.42, calc. 720.31; **$17e_2$**: $[M+H]^+$ obs. 1313.01, calc. 1313.52). The results are shown in Fig. S72.

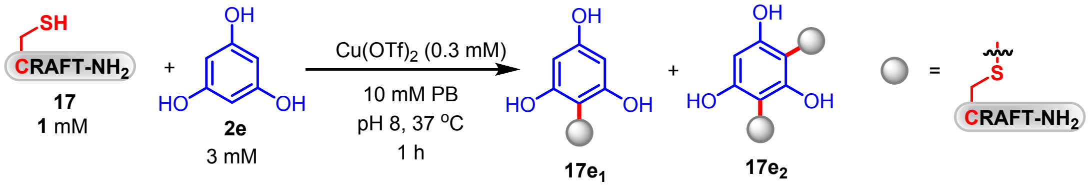

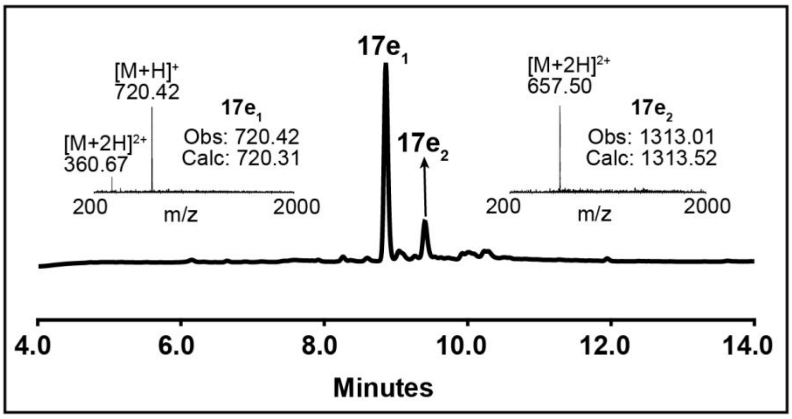

Figure S72. The modification of peptide **17** with **2e**.

*8. The modification of peptide **18** with **2a**.*

(18a): Prepared according to the general procedure A, the reaction progress was monitored by HPLC **method A**, conversion: 87%. The product was characterized by ESI-MS (mass obs. 2214.91 Da, calc. 2215.28 Da). The results are shown in Fig. S73.

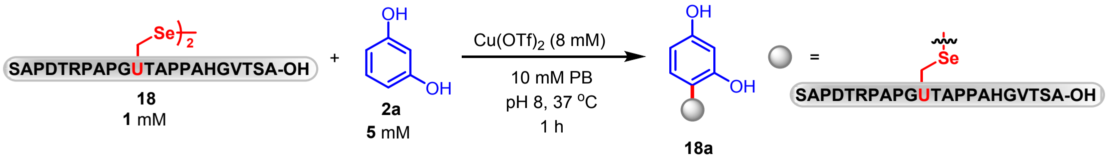

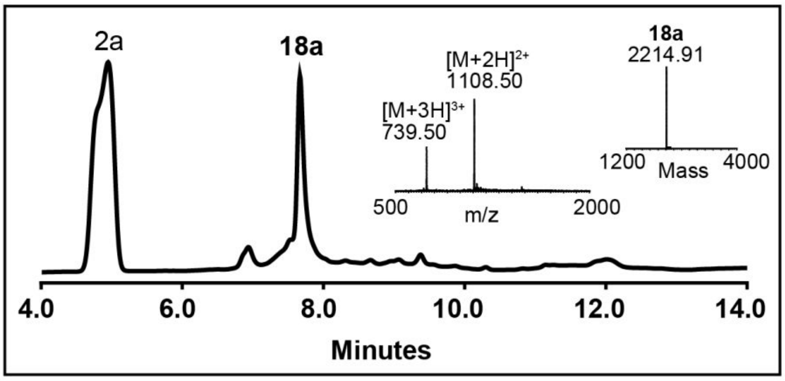

Figure S73. The modification of peptide **18** with **2a**.

*9. The modification of peptide **19** with **2e**.*

(19e): Prepared according to the general procedure B, the reaction progress was monitored by HPLC **method C**, conversion: 73%. The product was characterized by ESI-MS (mass obs. 2183.97 Da, calc. 2184.37 Da). The results are shown in Fig. S74. **19'** is the dimer of **19**.

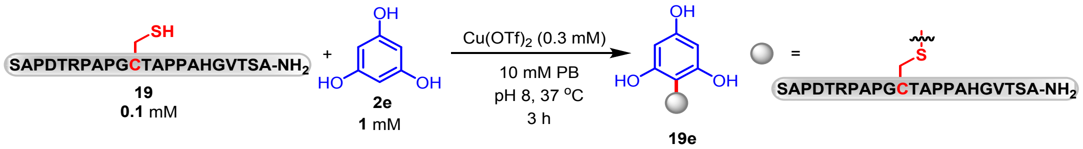

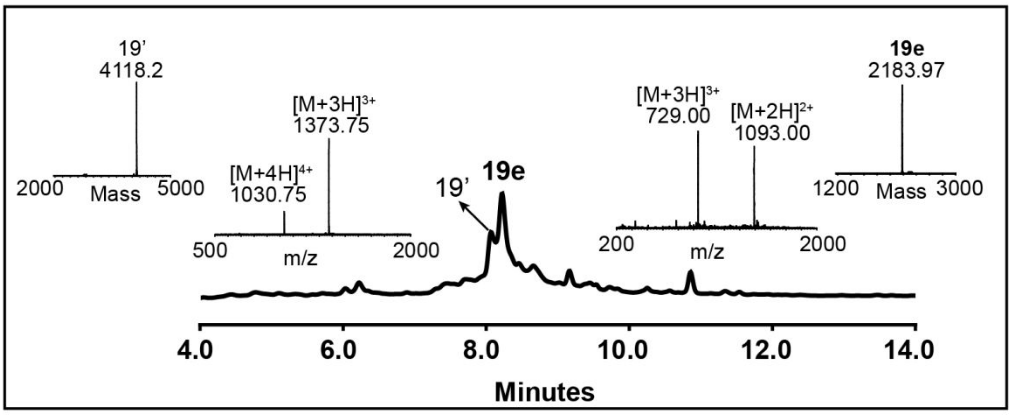

Figure S74. The modification of peptide **19** with **2e**.

6. Peptide Stapling

According to general approach for peptide modification, the stock solution of different peptide (100 mM), **2e** (100 mM) and $Cu(OTf)_2$ (100 mM) were prepared for peptide stapling.

Procedure for the trypsinization of stapled conjugates peptide for the analysis

To a 1.5 mL Eppendorf tube, a solution of stapled conjugate peptide (8 μL, 44 μg, 0.6 mM final concentration) was added with 21 μL TDW, and a solution of NH_4HCO_3 (10 μL, 0.1 M in TDW, 25 mM final concentration) to attain a pH value between ~7.5-8.0. To the resultant peptide solution a solution of trypsin (1.1 μL, 0.95 μg/μL, 1.1 μg) was added at 1:40 enzyme : substrate ratio. The mixture was then incubated at 37 ^{o}C for 30 mins. After 30 mins, the samples were desalted using C18 column and analyzed by ESI LC-MS. Same procedure was followed for the trypsin digestion of all peptide conjugates.

*1. The stapled reaction between peptide **20** and **2e**.*

(20e): Prepared according to the general procedure A, the reaction progress was monitored by HPLC **method A**, conversion: 90%. The product was characterized by ESI-MS ($[M+H]^+$ obs. 1583.50, calc. 1583.51). The results are shown in Fig. S75.

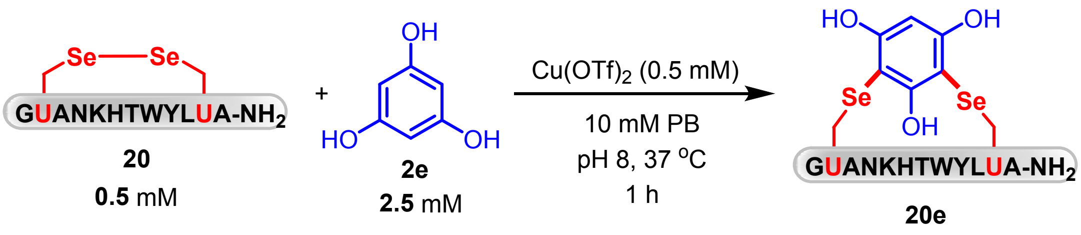

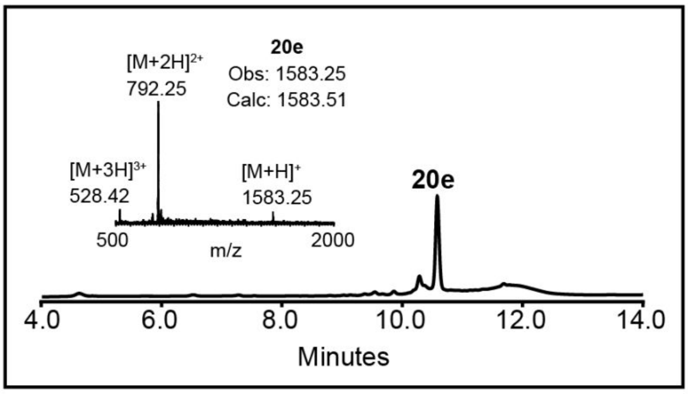

Figure S75. The stapled reaction between peptide **20** and **2e**.

*2. The trypsin digestion reaction of stapled conjugates **20e**.*

(20e*): Prepared according to the trypsin digestion procedure, the reaction progress was monitored by HPLC **method A**. The product was characterized by ESI-MS ($[M+H]^+$ obs. 1603.05, calc. 1602.50). The results are shown in Fig. S76.

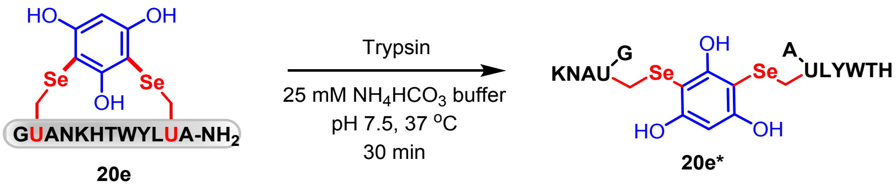

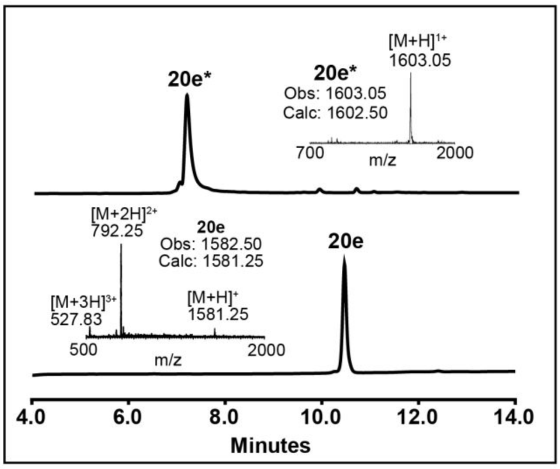

Figure S76. The trypsin digestion of stapled conjugates **20e**.

*3. The stapled reaction between peptide **21** and **2e**.*

(21e): Prepared according to the general procedure A, the reaction progress was monitored by HPLC **method A** conversion: 94%. The product was characterized by ESI-MS (mass obs. 2895.72 Da, calc. 2895.24 Da). The results are shown in Fig. S77. **21e'** is the oxidized form of **21e**.

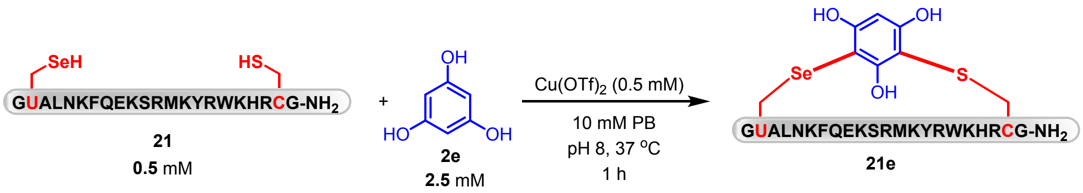

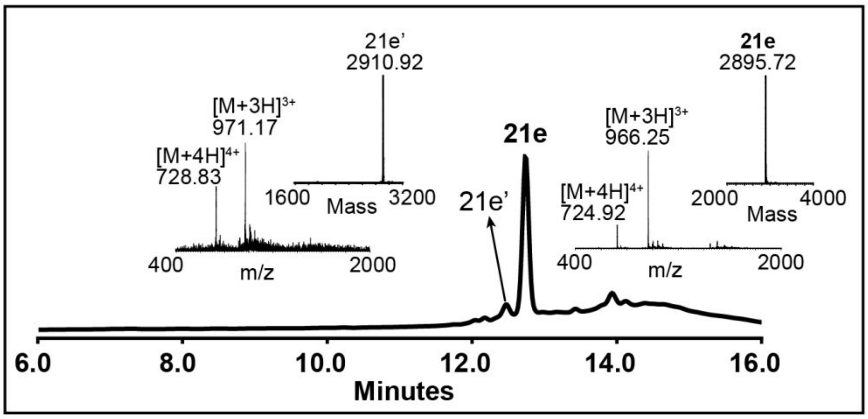

Figure S77. The stapled reaction between peptide **21** and **2e**.

*4. The trypsin digestion reaction of stapled conjugates **21e**.*

(21e*): Prepared according to the trypsin digestion procedure, the reaction progress was monitored by HPLC **method D**. The product was characterized by ESI-MS ($[M+H]^+$ obs. 952.17, calc. 952.31). The results are shown in Fig. S78.

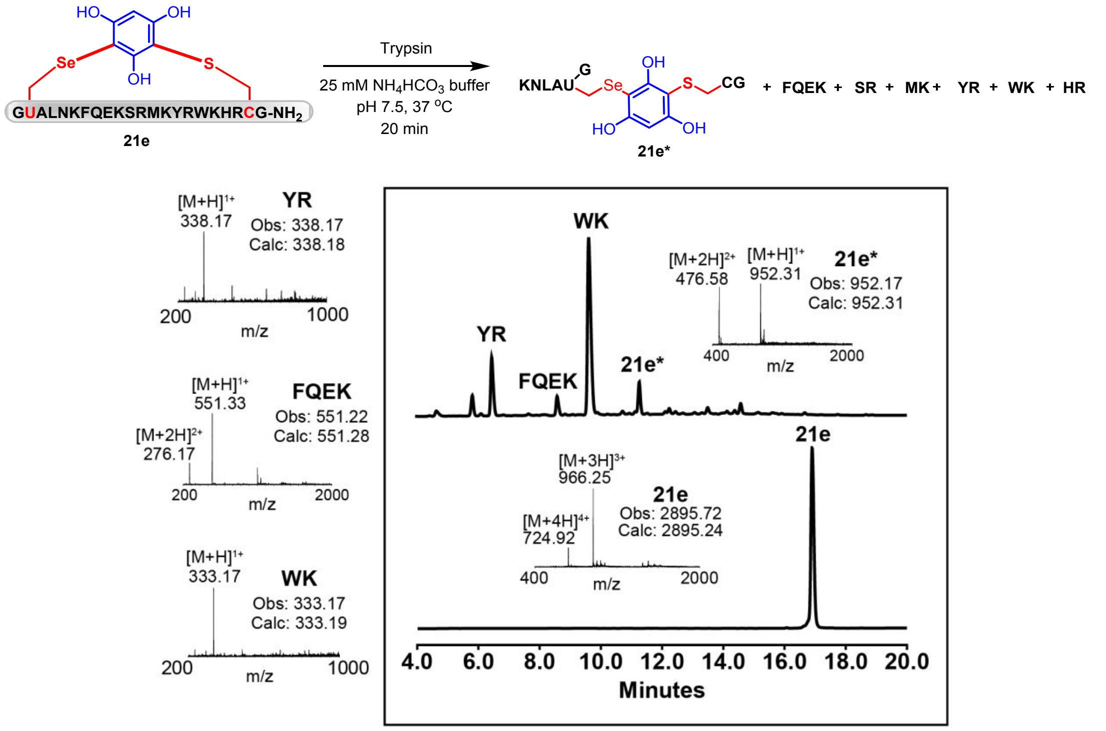

Figure S78. The trypsin digestion of stapled conjugates **21e**.

*5. The stapled reaction between peptide **22** and **2e**.*

(22e): Prepared according to the general procedure B, conducting the reaction with degassed solution, the reaction progress was monitored by HPLC **method C** conversion: 89% (**22e**:**22e'** 64:25). The product was characterized by ESI-MS ($[M+H]^+$ obs. 1487.50, calc. 1487.62). The results are shown in Fig. S79. **22e'** is the oxidized form of **22e**.

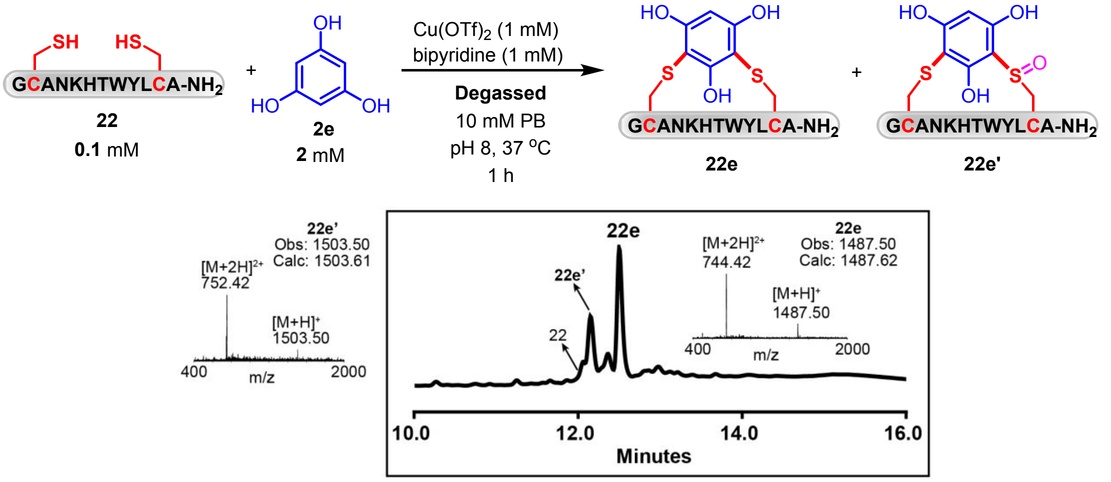

Figure S79. The stapled reaction between peptide **22** and **2e**.

*6. The trypsin digestion reaction of stapled conjugates **22e****.

(22e*): Prepared according to the trypsin digestion procedure, the reaction progress was monitored by HPLC. HPLC condition: XSelect C18 column (3.5 µm, 130 Å, 4.6 × 150 mm), column temp: 30 °C, gradient of 1%-40% ACN in H_2O with 0.1 TFA over 25 min. The product was characterized by ESI-MS ($[M+H]^+$ obs. 1505.25, calc. 1505.69). The results are shown in Fig. S80.

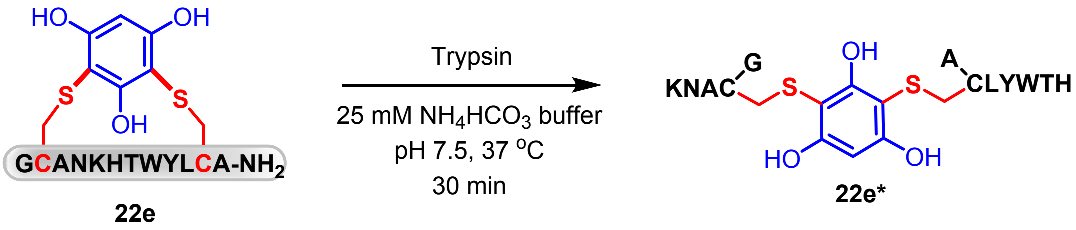

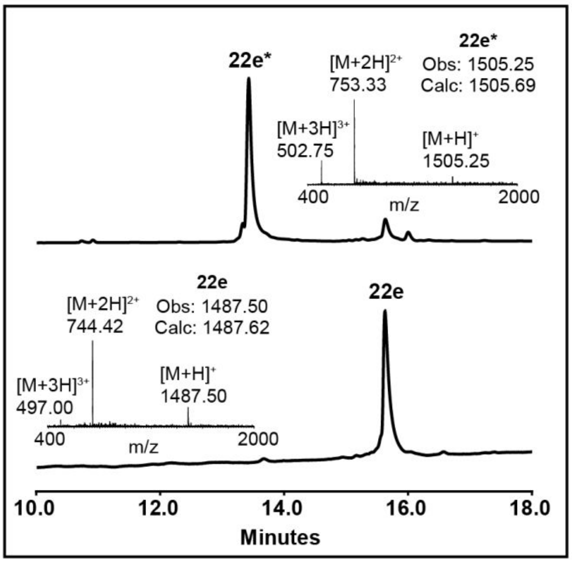

Figure S80. The trypsin digestion of stapled conjugates **22e**.

7. Stepwise Cross-coupling

According to general approach for peptide modification, the stock solution of different peptide (100 mM), **2e** (100 mM), **2f** (100 mM) and $Cu(OTf)_2$ (100 mM) were prepared for peptide modification.

General procedure for step-wise cross coupling: **Step 1**: follow the general procedure B to tether small molecule with peptide **4**. **Step 2**: Adding the corresponding amounts of Sec-containing peptide and additional copper into the reaction mixture, vortexed for 30 seconds, then place it in a 37 °C water bath for the indicated time (15 min-1 h). The reaction progress was monitored by HPLC and ESI-MS.

*1. The stepwise cross-coupling reaction between peptides **1** and **4** with **2e** in one pot.*

(24): Prepared according to the general procedure for stepwise cross coupling, the reaction progress was monitored by HPLC. HPLC condition: XSelect C18 column (3.5 µm, 130 Å, 4.6 × 150 mm), column temp: 30 ºC, gradient of 15%-45% ACN in H_2O with 0.1 TFA over 25 min. The product was characterized by ESI-MS ($[M+H]^+$ obs. 1233.25, calc. 1233.52). The results are shown in Fig. S81. * is a oxidation side-product from **4**.

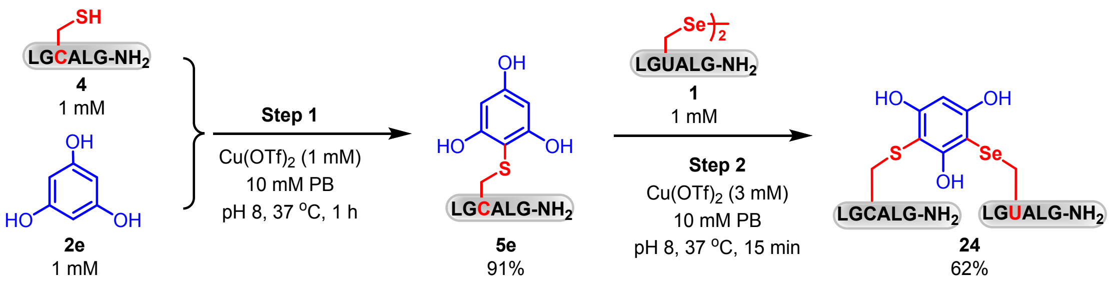

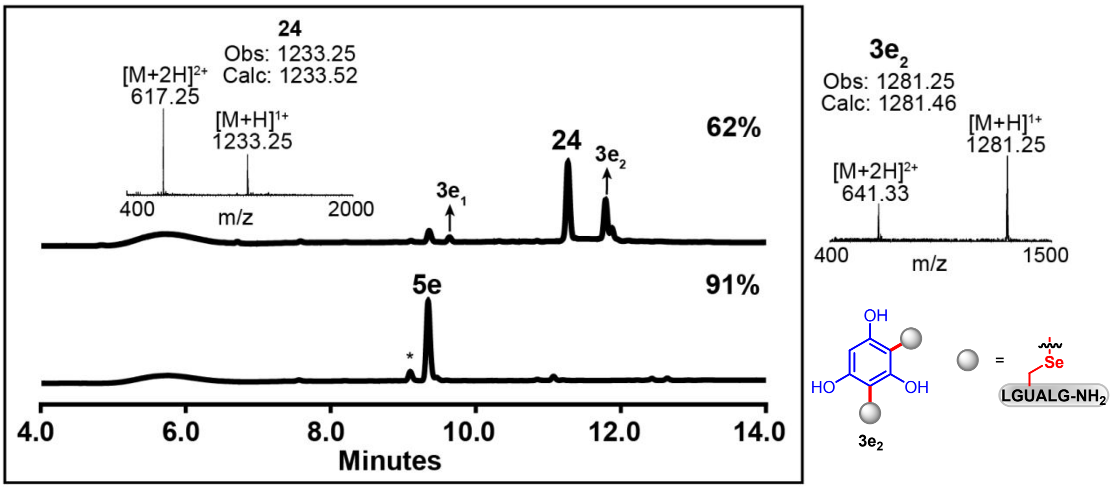

Figure S81. The step-wisely cross-coupling reaction between peptides **1** and **4** with **2e** in one pot.

*2. The stepwise cross-coupling reaction between peptides **1** and **4** with **2f**.*

(25): Prepared according to the general procedure for step-wise cross coupling, the reaction progress was monitored by HPLC. HPLC condition: XSelect C18 column (3.5 µm, 130 Å, 4.6 × 150 mm), column temp: 30 °C, gradient of 15%-45% ACN in H_2O with 0.1 TFA over 25 min. The product was characterized by ESI-MS ($[M+H]^+$ obs. 1261.33, calc. 1261.51). The results are shown in Fig. S82. * is the oxidation side-product from peptide **1**.

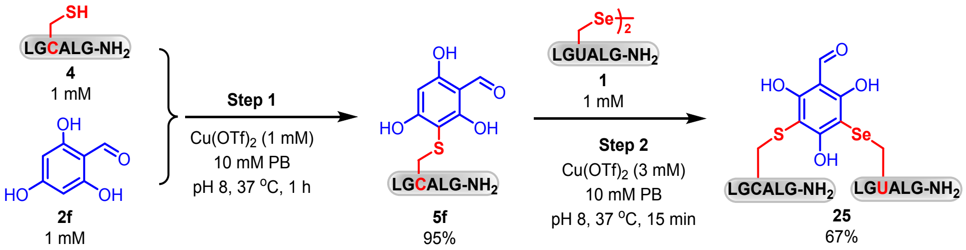

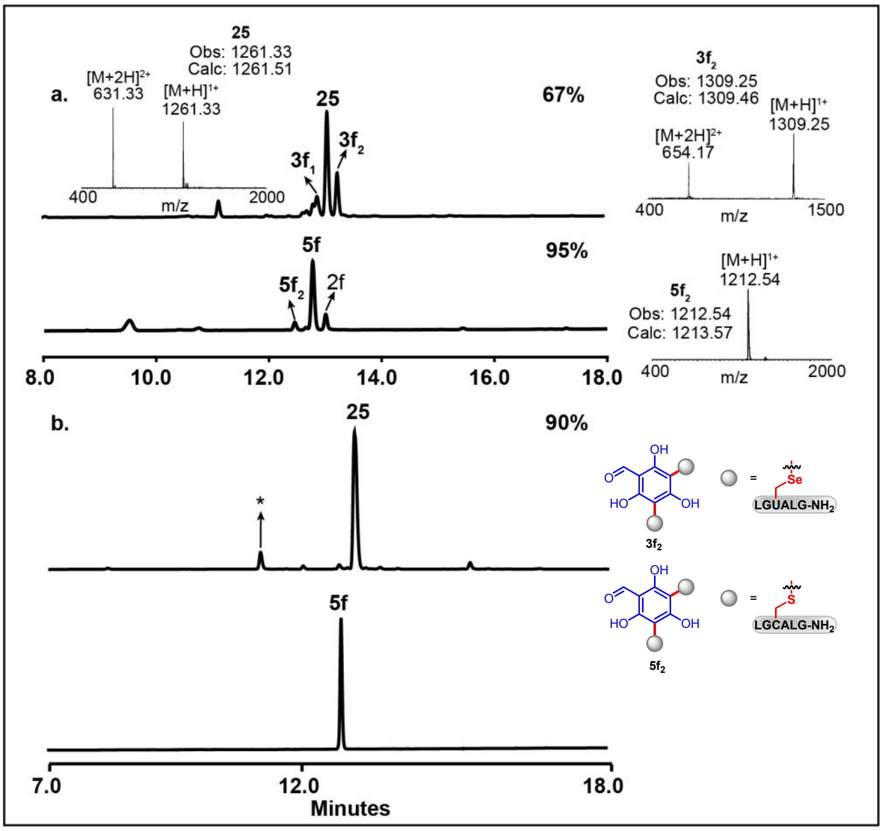

Figure S82. **a**. The stepwise cross-coupling of peptides **1** and **4** with **2f** in one pot. **b**. The cross-coupling reaction between peptides **1** with the purified conjugate **5f**.

*3. The stepwise cross-coupling reaction between peptides **4** and **16** with **2e** in one pot.*
(26): Prepared according to the general procedure for step-wise cross coupling, the reaction progress was monitored by HPLC **method C**. The product was characterized by ESI-MS (mass obs. 2110.83 Da, calc. 2110.25 Da). The results are shown in Fig. S83. * is a oxidation side-product from **4**.

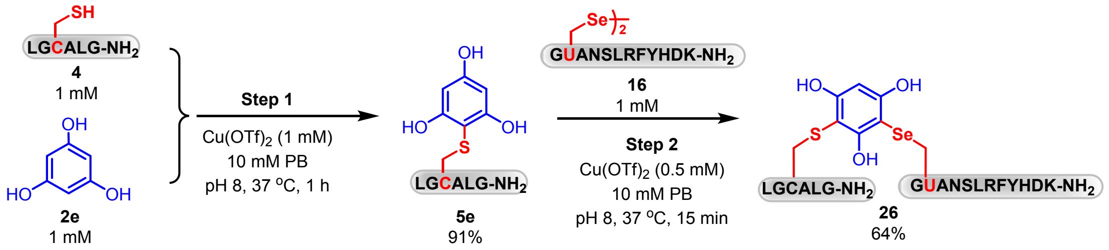

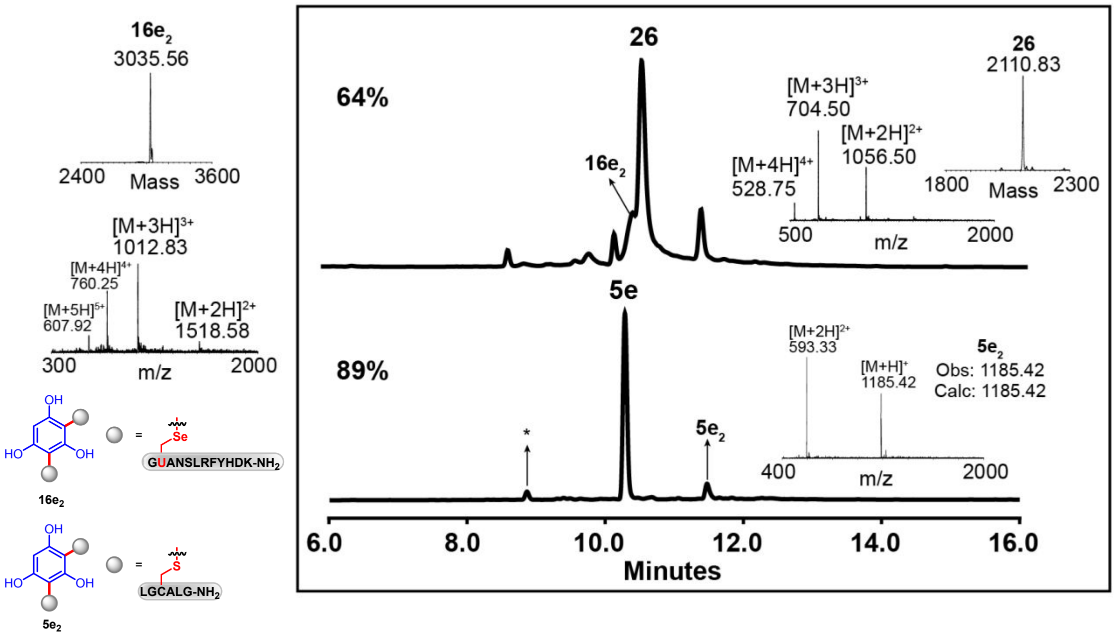

Figure S83. The stepwise cross-coupling reaction between peptides **4** and **16** with **2e** in one pot.

*4. The stepwise cross-coupling reaction between peptides **4** and **16** with **2f** in one pot.*

(27): Prepared according to the general procedure for step-wise cross coupling, the reaction progress was monitored by HPLC **method C**. The product was characterized by ESI-MS (mass obs. 2138.25 Da, calc. 2138.26 Da). The results are shown in Fig. S84. * is a oxidation side-product from **4**.

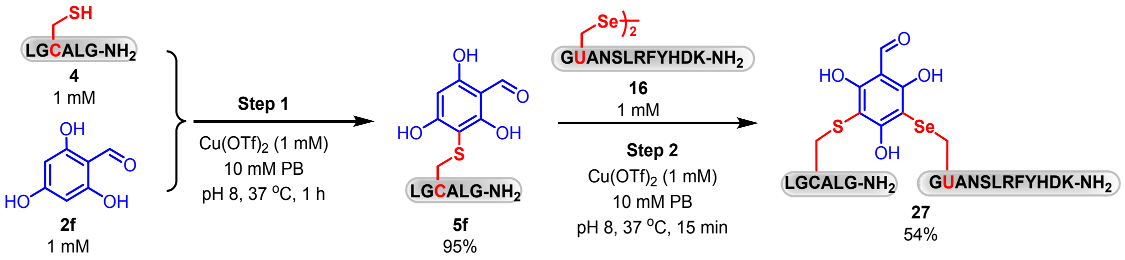

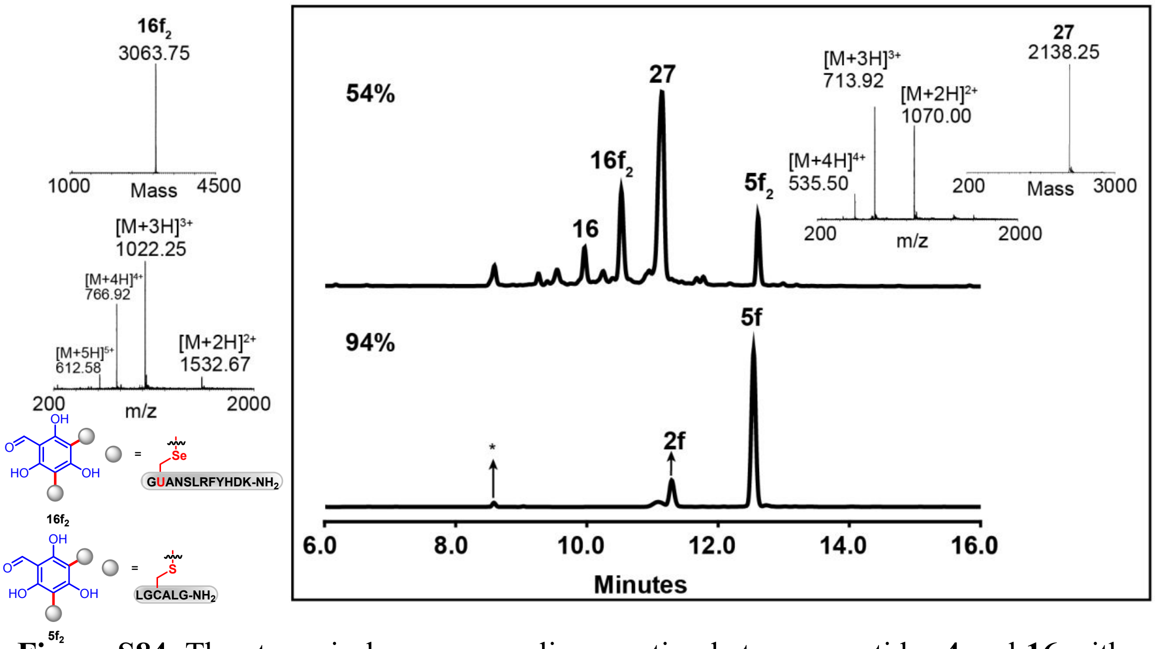

Figure S84. The step-wisely cross-coupling reaction between peptides **4** and **16** with **2f** in one pot.

*5. The stepwise cross-coupling reaction between peptides **4** and **23** with **2e** in one pot.*

(28): Prepared according to the general procedure for step-wise cross coupling, the reaction progress was monitored by HPLC **method C**. The product was characterized by ESI-MS (mass obs. 2062.93 Da, calc. 2063.34 Da). The results are shown in Fig. S85. * is a oxidation side-product from **4**.

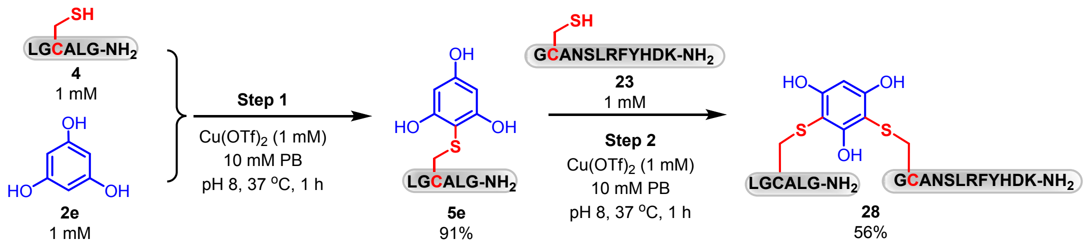

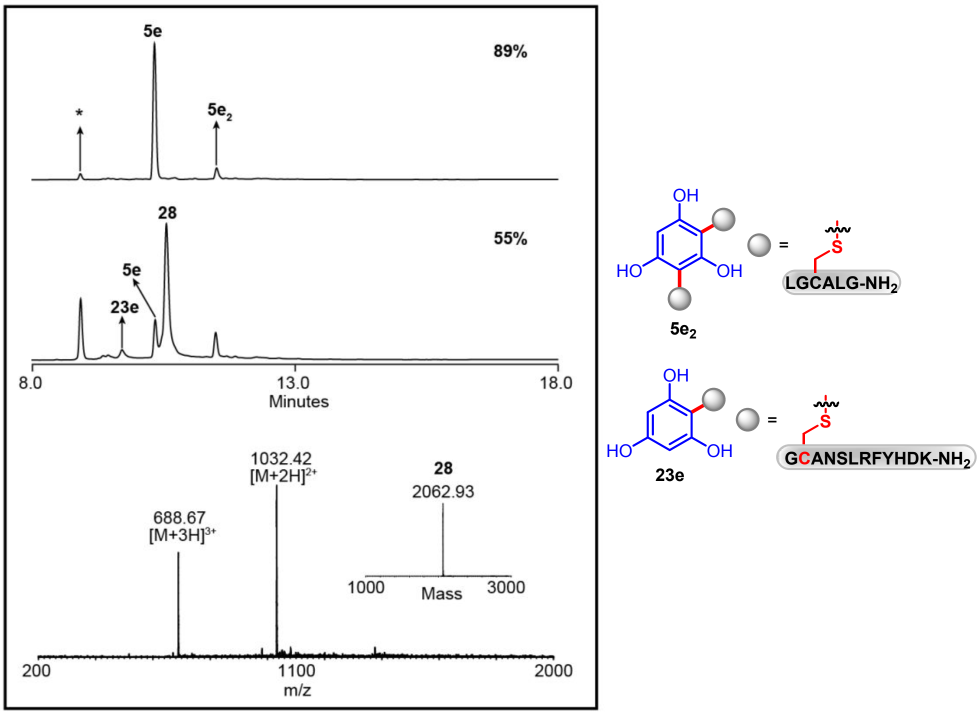

Figure S85. The stepwise cross-coupling reaction between peptides **4** and **23** with **2e** in one pot.

8. Protein Synthesis

The chemical synthesis of Ub(2-76)(Q2U) 29, Ub(2-76)(Q2C) 30.

The sequence of wild type Ub, Ub(1-76) (PDB:1UBQ):

```
         10          20           30         40
MQIFVKTLTG  KTITLEVEPS   DTIENVKAKI  QDKEGIPPDQ

         50          60           70
QRLIFAGKQL  EDGRTLSDYN  IQKESTLHLV  LRLRGG
```

We prepared Ub(2-76)(Q2U) **29** from two peptide segments with ligation reaction, deselenization processes and Fmoc-deprotected selenazolidine. Ub(2-76)(Q2C) **30** from two peptide segments with ligation reaction, deselenization processes. The ligation sites were bold and underlined.

1) The synthesis of Ub(2-45)(Q2U)-MPAA thioester (29a$_1$)

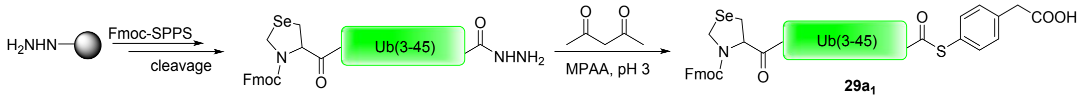

Scheme S1. The synthesis of Ub(2-45)(Q2U)-MPAA thioester (**29a$_1$**).

The procedure for the synthesis of C-terminal peptide hydrazides: 2-chlorotrityl chloride resin (0.5 mmol/g, 0.25 mmol scale) was swelled in DMF for 1 h. The resin was double treated with freshly prepared 10% hydrazine in DMF for 30 min and drained.[3] The resin was washed well with DMF and then treated with 10% MeOH in DMF for 30 min. This hydrazine functionalized resin can be utilized in standard peptide couplings.

The synthesis of Fmoc-Ub(2-45)(Q2U)-$NHNH_2$ was carried out on hydrazide functionalized 2-chlorotrityl chloride resin (0.5 mmol/g, 0.25 mmol scale) on automated peptide synthesizer. The first amino acid Phe was double coupled and subsequent steps were completed with standard Fmoc-SPPS. Fmoc-Sec(Mob)-OH replacing Gln2 was manually coupled at r.t for 2 h (activation: 2 equiv Fmoc-Sec(Mob)-OH, 2 equiv OxymaPure, and 1.9 equiv DIC in 1:1 mixture of CH_2Cl_2 and DMF at 0 °C for 5 min). After peptide chain assembly, the resin was washed well with DMF/DCM, and dried under vacuum. The peptide was cleaved according to the general procedure and lyophilized.

The thioesterification of Fmoc-Ub(2-45)(Q2U)-$NHNH_2$: The crude peptide was dissolved in 20 mL PB buffer (200 mM, 6 M Gn·HCl, pH 3) and treated with MPAA (200 mM) and 25 equiv acetylacetone (acac) for 3 h at 25 °C.[4] The reaction was monitored using analytical HPLC with method F, purified by preparative RP-HPLC and characterized with ESI-MS. RP-HPLC (C4 column) to give the corresponding Ub(2-45)(Q2U)-MPAA peptide thioester (**29a$_1$**) in ~30% yield. The product was

characterized by ESI-MS (mass obs. 5388.87 Da, calc. 5389.13 Da, Fig. S86).

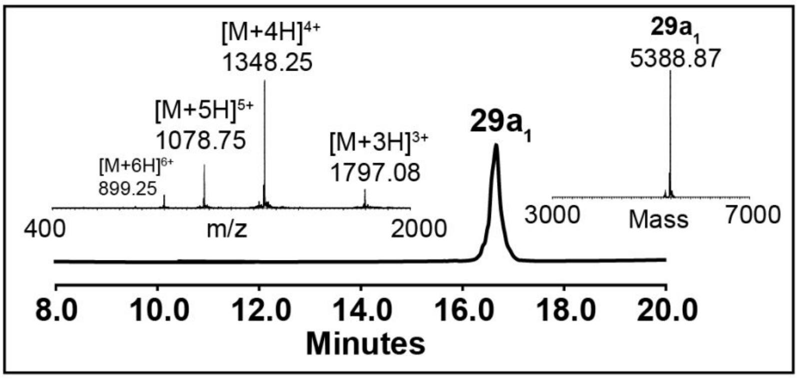

Figure S86. The HPLC and ESI-MS spectra of **29a$_1$**.

2) The synthesis of Ub(46-76)(A46U) monomer (29a$_2$)

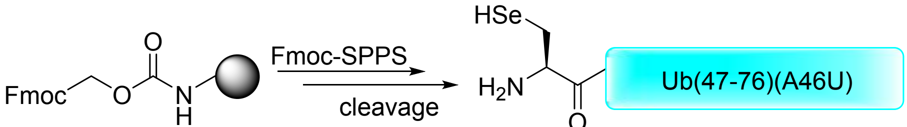

Scheme S2. The synthesis of Ub(46-76)(A46U) monomer (**29a$_2$**).

The synthesis of Ub(46-76)(A46U) monomer (**29a$_2$**) was carried out on H-RINK AMIDE-CHEMMATRIX resin (0.19 mmol/g, 0.25 mmol scale) on automated peptide synthesizer. The first amino acid Gly76 was double coupled and subsequent steps were completed with standard Fmoc-SPPS. Fmoc-Sec(Mob)-OH replacing Ala46 was manually coupled at r.t for 2 h (activation: 2 equiv Fmoc-Sec(Mob)-OH, 2 equiv OxymaPure, and 1.9 equiv DIC in 1:1 mixture of CH_2Cl_2 and DMF at 0 °C for 5 min). After peptide chain assembly, the peptide was cleaved according to the general procedure and lyophilized. The corresponding peptide Ub(46-76)(A46U) monomer (**29a$_2$**) was obtained in ~30% yield by preparative RP-HPLC. The HPLC analysis was carried with method F. The product was characterized by ESI-MS (with TCEP/sodium ascorbate, mass of the monomer obs. 3547.11 Da, calc. 3546.92 Da, Fig. S87).

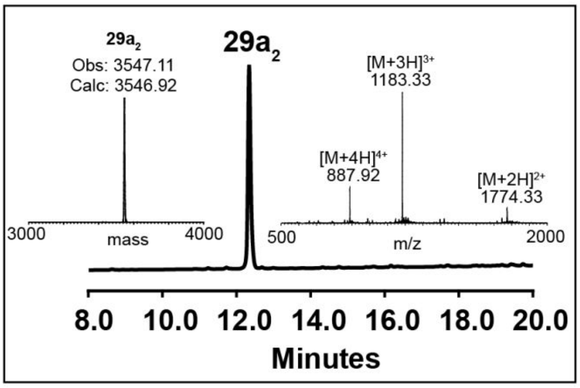

Figure S87. The HPLC of Ub(46-76)(A46U) (**29a$_2$**).

3) The synthesis of Ub(2-45)(Q2C)-MPAA thioester (30a$_1$)

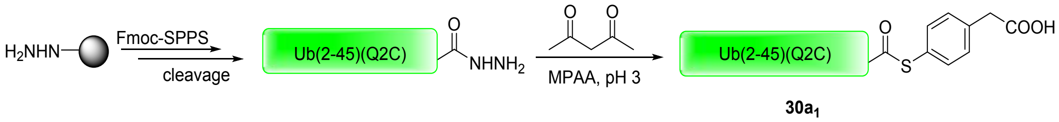

Scheme S3. The synthesis of Ub(2-45)(Q2C)-MPAA thioester (**30a$_1$**).

The procedure for the synthesis of C-terminal peptide hydrazides: 2-chlorotrityl chloride resin (0.5 mmol/g, 0.25 mmol scale) was swelled in DMF for 1 h. The resin was double treated with freshly prepared 10% hydrazine in DMF for 30 min and drained. The resin was washed well with DMF and then treated with 10% MeOH in DMF for 30 min. This hydrazine functionalized resin can be utilized in standard peptide couplings.

The synthesis of Ub(2-45)(Q2C)-NHNH$_2$ was carried out on hydrazide functionalized 2-chlorotrityl chloride resin[12] (0.5 mmol/g, 0.25 mmol scale) on automated peptide synthesizer. After peptide chain assembly, the resin was washed well with DMF/DCM, and dried under vacuum. The peptide was cleaved according to the general procedure and lyophilized.

The thioesterification of Ub(2-45)(Q2C)-NHNH$_2$: The crude peptide was dissolved in 20 mL PB buffer (200 mM, 6 M Gn·HCl, pH 3) and treated with MPAA (200 mM) and 25 equiv acetylacetone (acac) for 3 h at 25 °C. The reaction was monitored using analytical HPLC with method F, purified by preparative RP-HPLC and characterized with ESI-MS. RP-HPLC (C4 column) to give the corresponding Ub(2-45)(Q2C)-MPAA peptide thioester (**30a$_1$**) in ~30% yield. The product was characterized by ESI-MS (mass obs. 5106.77 Da, calc. 5107.96 Da, Fig. S88).

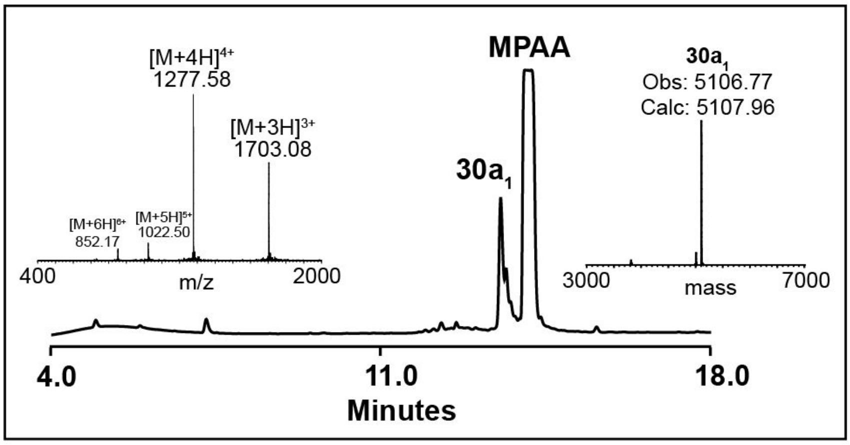

Figure S88. The HPLC for the thioesterification of Ub(2-45)(Q2C)-NHNH$_2$.

4) The NCL between Fmoc-Ub(2-45)(Q2U)-MPAA thioester (29a$_1$) and Ub(46-76)(A46U) monomer (29a$_2$) and deselenization of Ub(2-76)(Q2U, A46U) (29b) in one pot

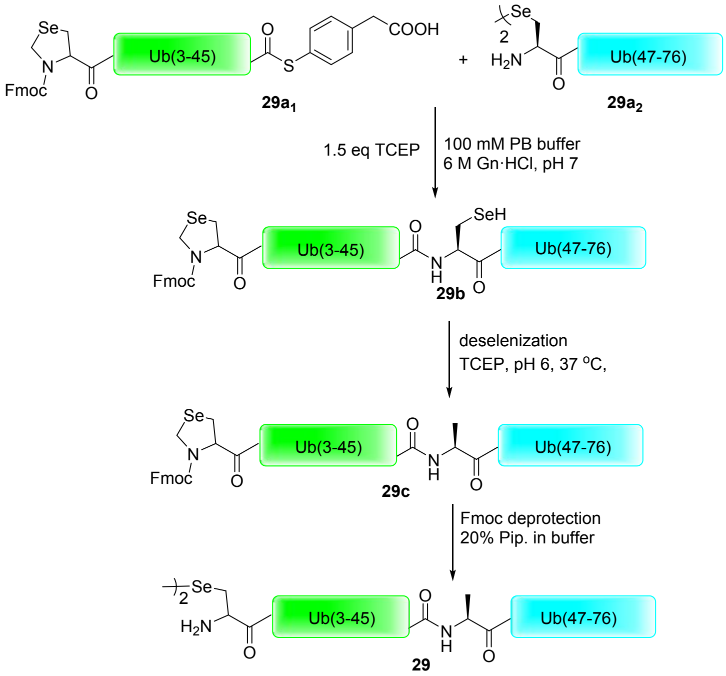

Scheme S4. The synthesis of Ub(2-76)(Q2U) **29**.

Ub(46-76)(A46U) monomer (**29a$_2$**, 9 mg, 2.6 µmol, 1 mM) was dissolved in 2.5 mL PB buffer (100 mM, 6 M Gn·HCl, pH 7) and the reaction mixtures were added to Fmoc-Ub(2-45)(Q2U)-MPAA thioester (**29a$_1$**, 14 mg, 2.6 µmol, 1 mM). Then add 1.2 mg TCEP to the buffer and check pH. The reaction mixtures were incubated at 37 °C for 3 h. The reaction progress was monitored by HPLC with a gradient of 5% B over 1 min then 5-70% B over 20 min by C4 column. After 3 h, to deselenize Fmoc-Ub(2-76)(Q2U, A46U) (**29b**) in one pot, 100 equiv TCEP in PB buffer (100 mM, pH 6) was incorporated into the mixture. **29b** can be fully deselenized by incubating the mixture at 37°C for 24 h. The reaction progress was monitored by HPLC with a gradient of 5% B over 1 min then 5-70% B over 20 min by C4 column and ESI-MS. After 24 h, ether was added to remove excess TCEP (x4 times) and lyophilized. The peptide was dissolved in 20% piperidine in the phosphate buffer at pH 10. After 6 h the Fmoc-deprotection reaction was completed to offer one-pot Fmoc deprotection and Sez opening. Final product Ub(2-76)(Q2U) **29** was isolated in 8% yield and characterized by ESI-MS (mass obs. 16907.61 Da, calc. 16907.20 Da, Fig. S89).

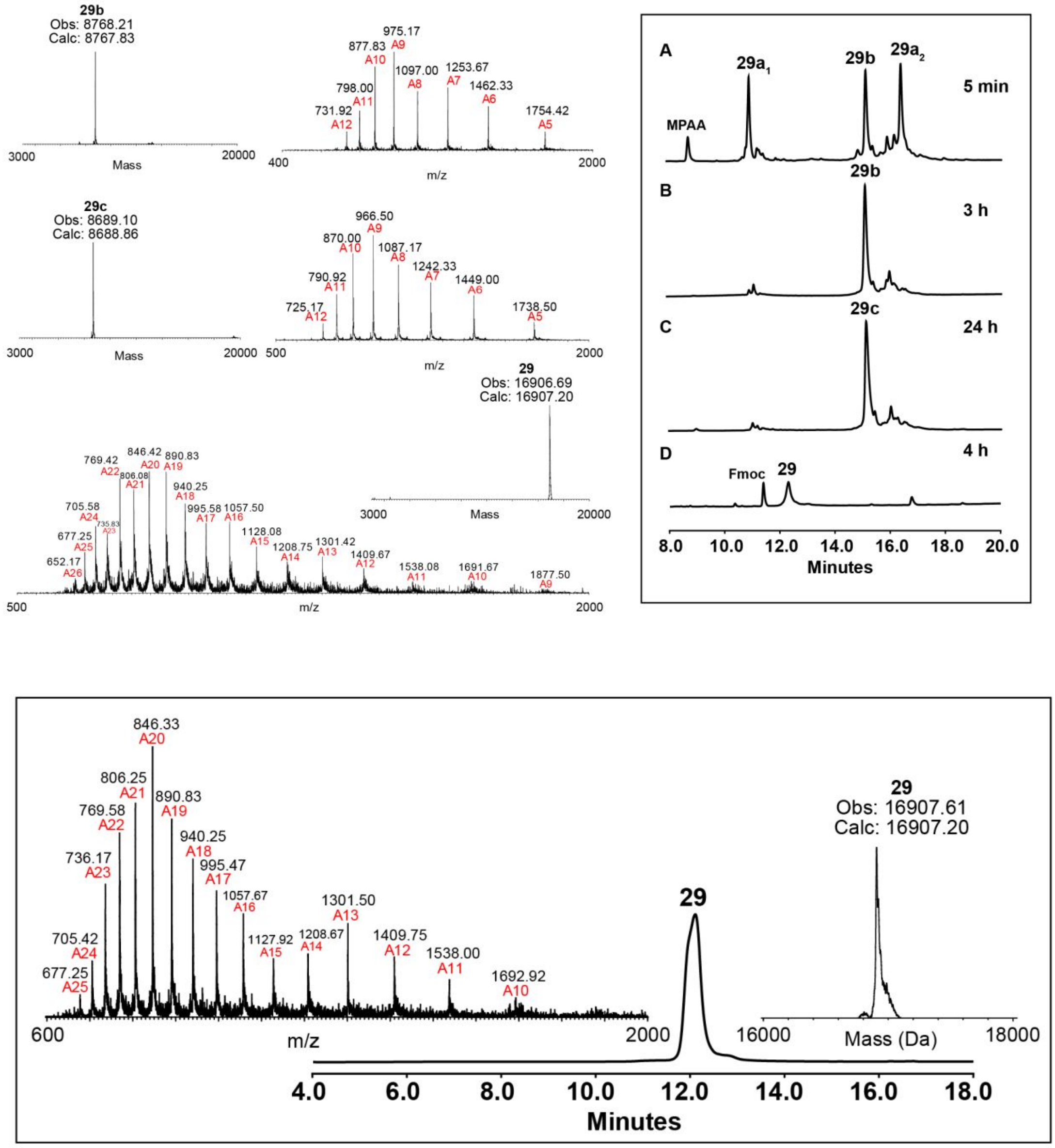

Figure S89. The synthesis of Ub(2-76)(Q2U) **29**, (analytic HPLC **method E**). **A**. NCL reaction between **29a$_1$** and **29a$_2$** for 5 mins. **B**. NCL reaction between **29a$_1$** and **29a$_2$** for 3 h. **C**. Deselenization reaction of **29b** for 24 h. **D**. Fmoc deprotection reaction of **29c** for 4 h.

5) The NCL between Ub(2-45)(Q2C)-MPAA thioester (30a₁) and Ub(46-76)(A46U) monomer (29a₂) and deselenization of Ub(2-76)(Q2C, A46U) (30a₂) in one pot

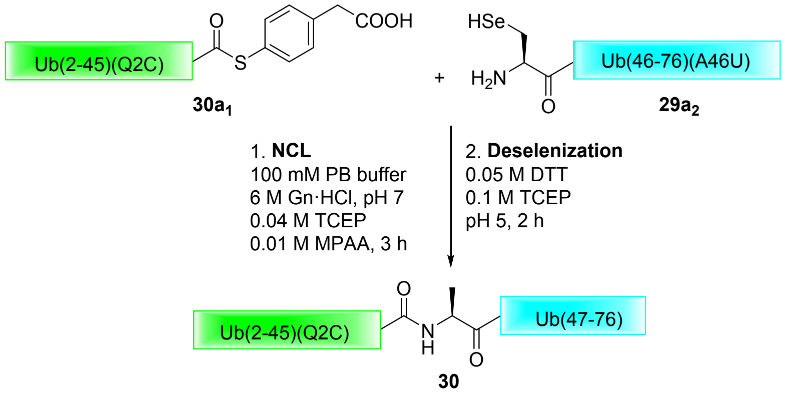

Scheme S5. The synthesis of Ub(2-76)(Q2C) **30**.

Ub(46-76)(A46U) monomer (**29a$_2$**, 4.3 mg, 1.2 μmol, 2 mM) was dissolved in 0.6 mL PB buffer (100 mM, 6 M Gn·HCl, 4 mM TCEP, pH 7) and the reaction mixtures were added to Ub(2-45)(Q2C)-MPAA thioester (**30a$_1$**, 3.1 mg, 0.6 μmol, 1 mM). The reaction mixture were incubated at 37 °C for 3 h. The reaction progress was monitored by HPLC with a gradient of 5% B over 1 min then 5-50% B over 20 min by C4 column. After 3 h, to deselenize Ub(2-76)(Q2C, A46U) (**30a$_2$**) in one pot, 10 mM DTT was first added to the reaction mixture and shaken for 10 mins. Subsequently, 100 equiv TCEP in PB buffer (100 mM, pH 5) was incorporated into the mixture. **30a$_2$** can be fully deselenized by incubating the mixture at 37°C for 2 h. The final product Ub(2-76)(Q2C) **30** was isolated in 25% yield and characterized by ESI-MS (mass obs. 8406.98 Da, calc. 8407.70 Da, Fig. S90).

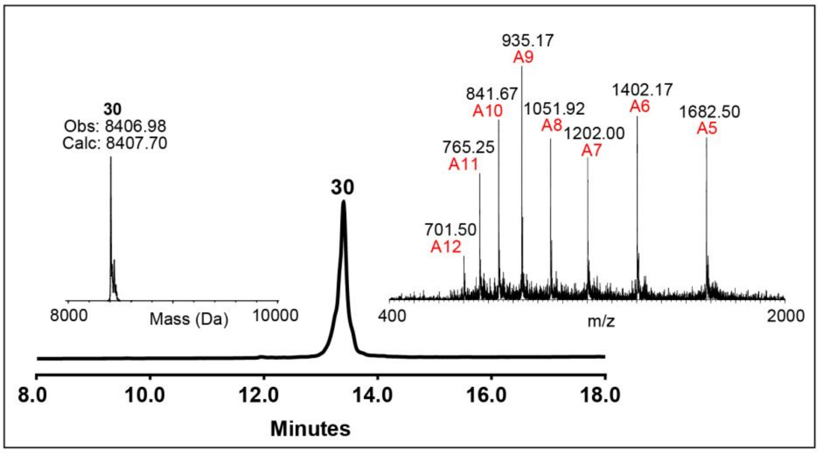

Figure S90. The final product Ub(2-76)(Q2C) **30**, (analytic HPLC using XSelect C4 column (3.5 μm, 130 Å, 4.6 × 150 mm) with a gradient of 5% B over 5 min then 5-50% B over 20 min at 220 nm).

The chemical synthesis of Sec-ZHER2 affibody 37 and Cys-ZHER2 affibody 38.

The sequence of wild type ZHER2 Affibody (PDB:2KZI):

10	20	30	40	50
VDNKFNKEM	RNAYWEIALL	PNLNNQQKRA	FIRSLYDDPS	QSANLLAEAK
59				
KLNDAQAPK				

1) The synthesis of Sec-ZHER2 affibody 37.

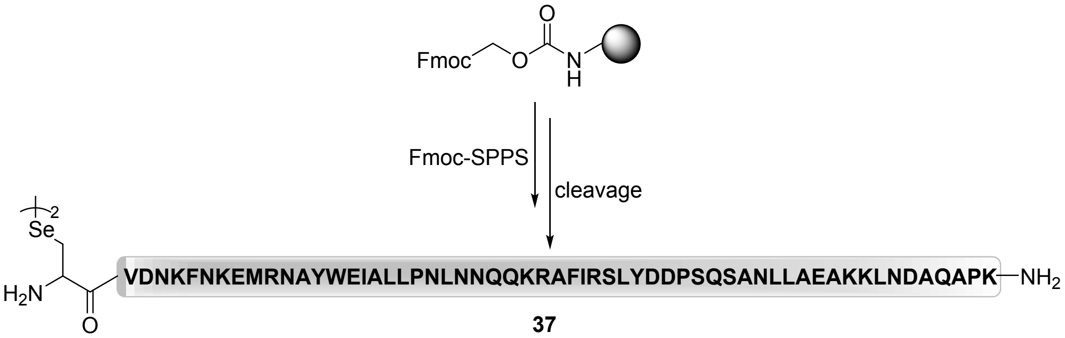

37

Scheme S6. The synthesis of Sec-ZHER2 affibody **37**.

The synthesis of Sec-ZHER2 affibody (**37**) was carried out on H-RINK AMIDE-CHEMMATRIX resin (0.19 mmol/g, 0.25 mmol scale) on automated peptide synthesizer. The first C-terminal amino acid Lys60 was double coupled and subsequent steps were completed with standard Fmoc-SPPS. The N-terminal Fmoc-Sec(Mob)-OH was manually coupled at r.t for 2 h (activation: 2 equiv Fmoc-Sec(Mob)-OH, 2 equiv OxymaPure, and 1.9 equiv DIC in 1:1 mixture of CH_2Cl_2 and DMF at 0 °C for 5 min). After peptide chain assembly, the peptide was cleaved according to the general procedure and lyophilized. The corresponding peptide Sec-ZHER2 affibody (**37**) was obtained in ~20% yield by preparative RP-HPLC. The HPLC analysis was carried with **method F**. The product was characterized by ESI-MS (mass obs. 13706.51 Da, calc. 13707.29 Da, Fig. S91).

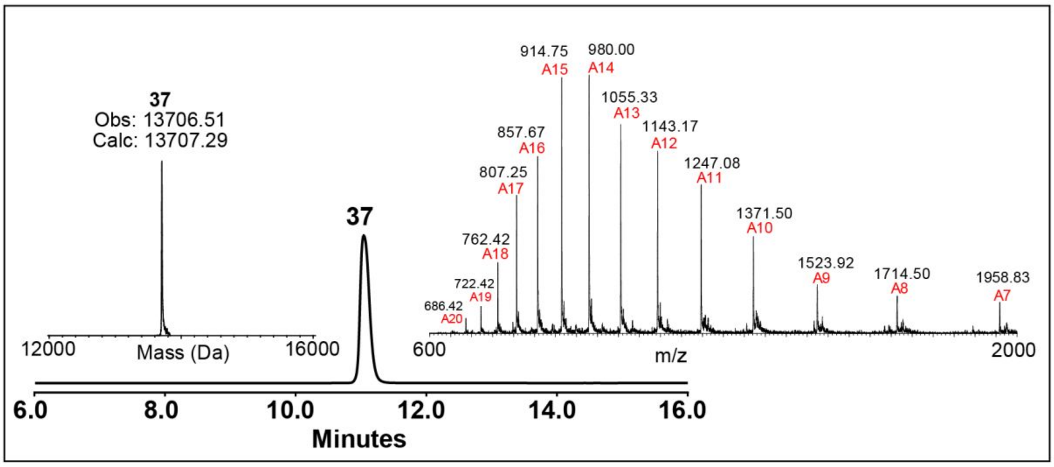

Figure S91. The synthesis of Sec-ZHER2 affibody **37**.

2) The synthesis of Cys-ZHER2 affibody 38.

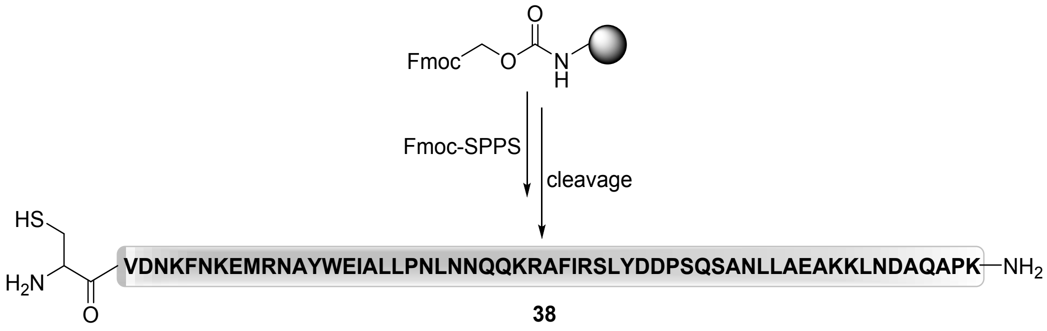

Scheme S7. The synthesis of Cys-ZHER2 affibody **38**.

The synthesis of Cys-ZHER2 affibody (**38**) was carried out on H-RINK AMIDE-CHEMMATRIX resin (0.19 mmol/g, 0.25 mmol scale) on automated peptide synthesizer. The first amino acid Lys59 was double coupled and subsequent steps were completed with standard Fmoc-SPPS. After peptide chain assembly, the peptide was cleaved according to the general procedure and lyophilized. The corresponding peptide Cys-ZHER2 affibody (**38**) was obtained in ~30% yield by preparative RP-HPLC. The HPLC analysis was carried with **method F**. The product was characterized by ESI-MS (mass obs. 6807.48 Da, calc. 6807.74 Da, Fig. S92).

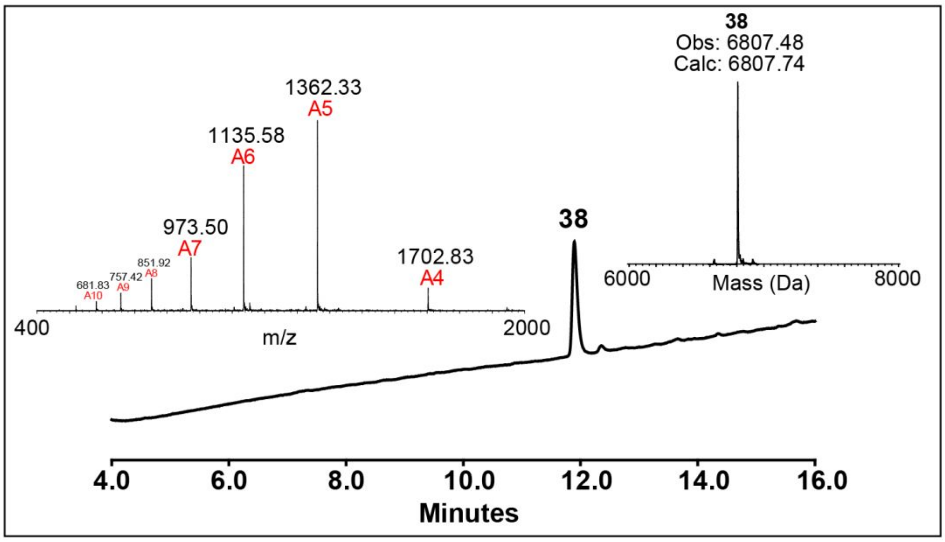

Figure S92. The synthesis of Cys-ZHER2 affibody **38**.

9. Protein Expression

Construct for Plasmid: A gene that encodes the sequences for Ub(1-76)(M1S, Q2C) mutant and Ub(1-76)(M1S) wild-type was inserted into linearized pETM11-SUMO3 vector via Gibson assembly. The gene was ordered from IDT as a gBlock® Gene Fragment.

Protein Expression. C43 cells served as a general host for expression. The culture of each recombinant E. coli strain was incubated in 2XYT medium (containing 1x NPS, 1% glucose and 50 μg/mL kanamycinat) at 37 ^oC until the cell density reached 0.6 at OD600. Protein expression was induced by the addition of Isopropyl β-D-1-thiogalactopyranoside (IPTG) to a final concentration of 0.1 mM, followed by further incubation at 37 ^oC for 4 h. The cells were harvested by centrifugation (10,000 rpm,15 min, 4^oC), the pellets were stored at -80 ^oC.

Protein Purification. 14 g E. coli cells were suspended in 70 mL of lysis buffer (1000 mM PBS, 150 mM NaCl, 0.2 mg/mL lysozyme, 50 μg/mL DNAase, 0.4 mM PMSF, 5 mM $MgCl_2$, 0.1 mM protease inhibitor and 10 mM imidazole, pH 8.0). The mixture was incubated on ice with shaking for 30 mins and then the cells were lysed by microfluidizer. DTT was added to the cell lysate to 1 mM final concentration. Cell debris was removed via centrifugation at 12,000 rpm for 30 min, 4 ^oC. The supernatant was incubated with 5 mL of Ni Sepharose resin (GE Healthcare) for 30 min. The Ni Sepharose column was pre-treated with 10 column volumes (CVs) of lysis buffer, followed by 10 CVs of wash buffer (1000 mM PBS, 150 mM NaCl, and 10 mM imidazole, pH 7), and 10 CVs of elution buffer (1000 mM PBS, 150 mM NaCl, and 500 mM imidazole, pH 7). The purity of proteins was determined by SDS-PAGE with a dye containing no thiols (Figure S93). Next, the SUMO protease ULP1 was introduced in a ratio with 1:50 ULP1:protein solution shaken for 19 h at 4 ^oC to cleave SUMO. Then a His Trap chelating column (GE Healthcare, USA) was used to remove His tag and SUMO. Desired protein fractions were concentrated through stirred ultrafiltration cells (Millipore). The purity of proteins was characterized via RP-HPLC and ESI-MS (Figure S94).

Yield (quantified using ε_{280} = 1490 M^{-1} cm^{-1}):

Ub(1-76)(M1S, Q2C) mutant (**34**): 30 mg/L

Ub(1-76)(M1S) wild-type (**35**): 32.4 mg/L

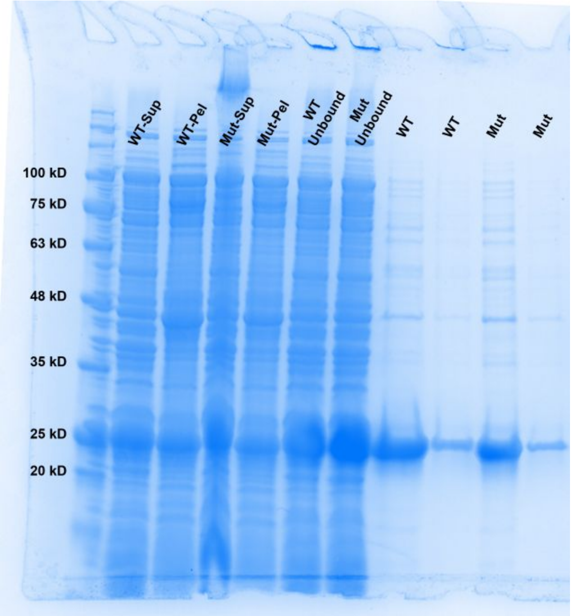

Figure S93. SDS-PAGE of fractions from NI and His-Trap purification for His6-TEV-SUMO3-Ub(1-76)(M1S) as wild-type (WT), His6-TEV-SUMO3-Ub(1-76)(M1S, Q2C) as mutant (Mut).

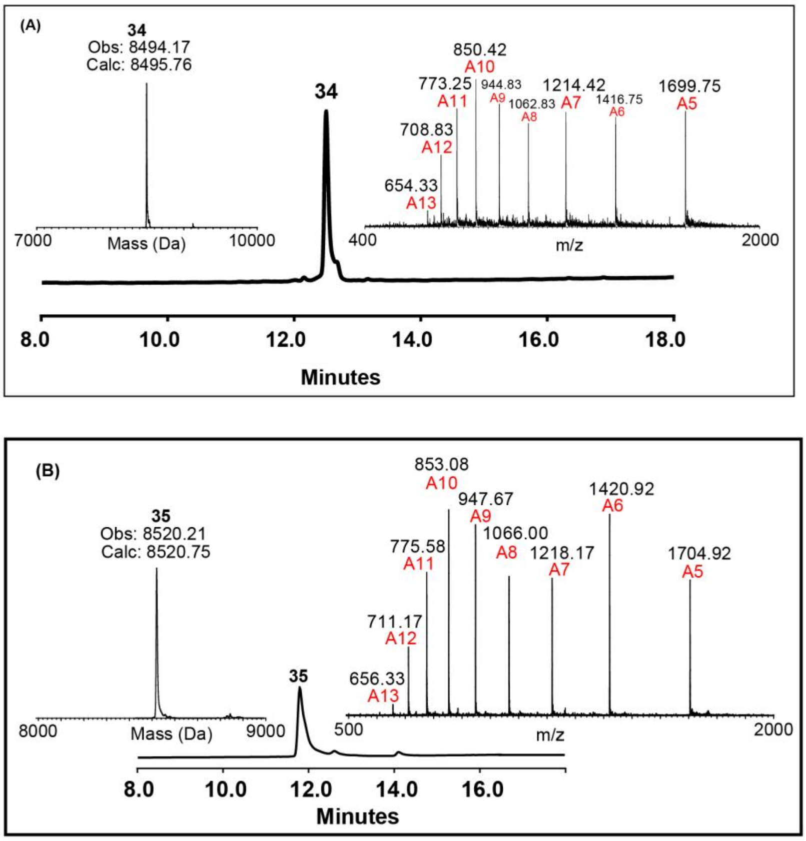

Figure S94. HPLC and ESI-MS data for (**A**). Expressed Ub(1-76)(M1S, Q2C) mutant (**34)** (mass obs. 8494.17 Da, cal. 8495.76 Da). (**B**). Expressed Ub(1-76)(M1S) WT (**35)** (mass obs. 8520.21 Da, cal. 8520.75 Da).

10. Protein Modification

According to general approach for protein modification, the stock solution of protein (1 mM, **29** and **30** dissolved in 0.6 M Gn·HCl, pH 7, other protein were dissolved in H_2O), small molecular (100 mM), bipyridine (100 mM, dissolved in EtOH) and $Cu(OTf)_2$ (100 mM) were prepared for protein modification.

1. The modification of Ub(2-76)(Q2U) ***29*** *with* ***9e****.*

(31): 5 µL Ub(2-76)(Q2U) **29** (dimer) stock solution were diluted dropwise in 39 µL TDW and waiting 0.5 h for folding, then diluted in 50 µL PB buffer (200 mM, pH 8), after that add 2µL **9e**, 2µL $Cu(OTf)_2$, 2µL bipyridine and mixtures. The reaction mixtures were incubated at 37 °C for 3h. The reaction progress was monitored by HPLC **method E**, conversion: 93%. The product was characterized by ESI-MS (mass obs. 8629.45 Da, calc. 8628.81 Da). The results are shown in Fig. S95. # is deselenization form of **29**.

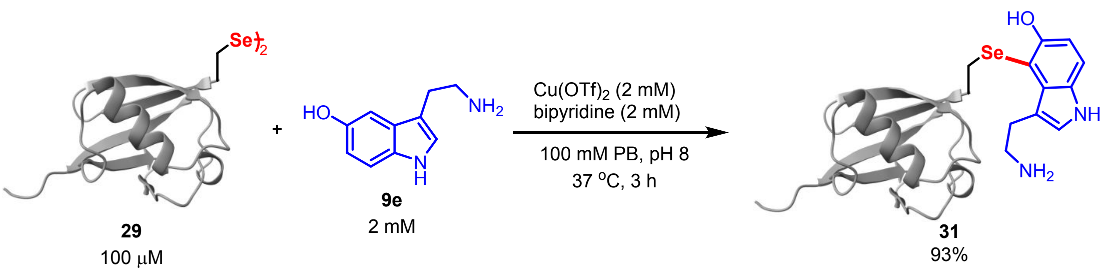

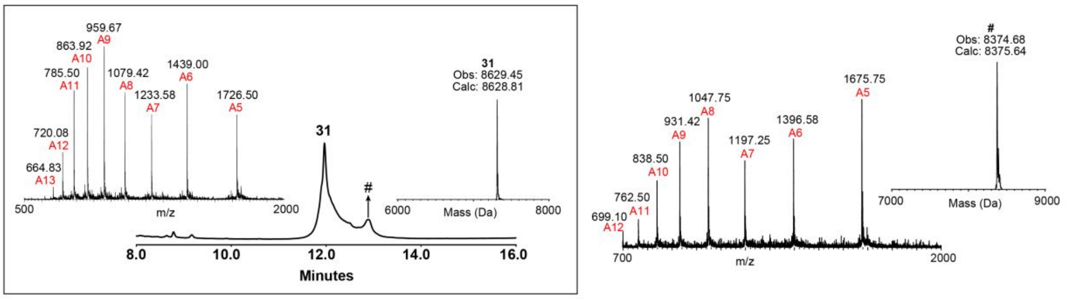

Figure S95. The modification of Ub(2-76)(Q2U) **29** with **9e**.

2. The modification of Ub(2-76)(Q2U) ***29*** *with* ***9d****.*

(32): 5 µL Ub(2-76)(Q2U) **29** (dimer) stock solution were diluted dropwise in 39 µL TDW and waiting 0.5 h for folding, then diluted in 50 µL Tris buffer (200 mM, pH 8), after that add 2µL **9d**, 2µL $Cu(OTf)_2$, 2µL bipyridine and mixtures. The reaction mixtures were incubated at 37 °C for 10 h. The reaction progress was monitored by HPLC **method E**, conversion: 79%. The product was characterized by ESI-MS (The product was characterized by ESI-MS (mass obs. 9901.94 Da, calc. 9901.86 Da). The results are shown in Fig. S96. # is deselenization form of **32**.

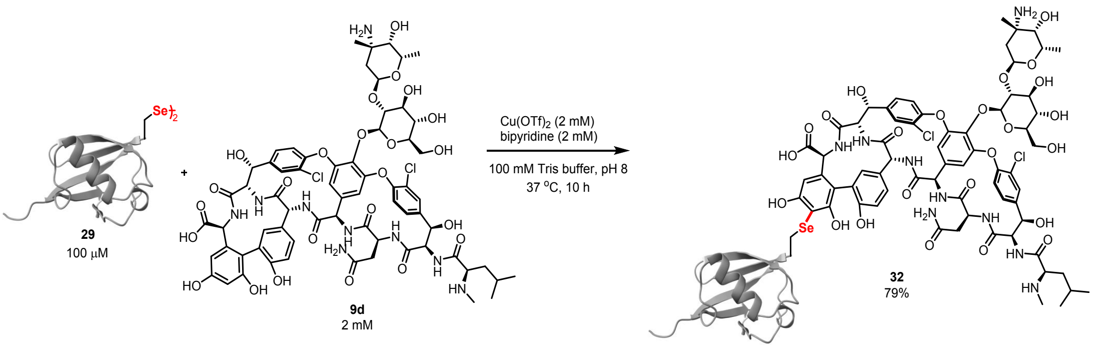

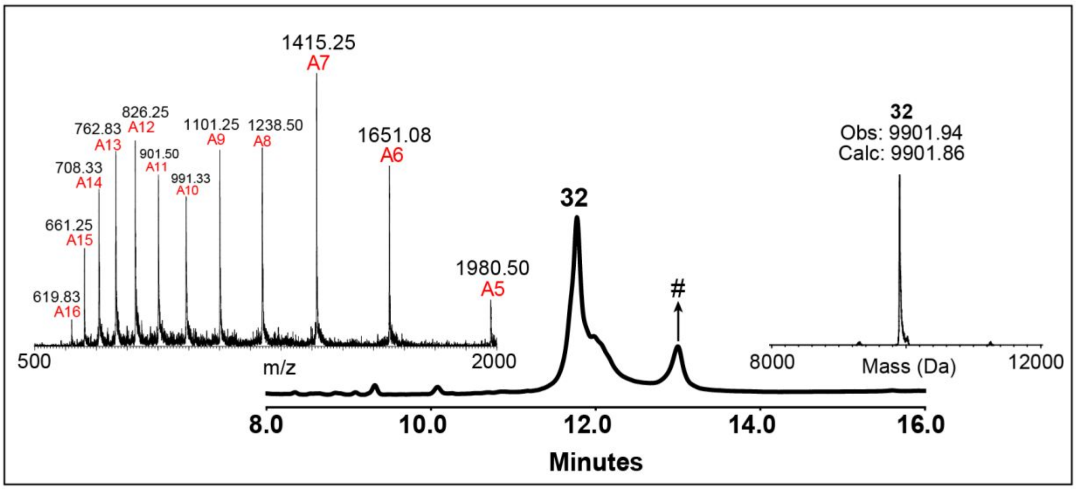

Figure S96. The modification of Ub(2-76)(Q2U) **29** with **9d**.

3. The modification of Ub(2-76)(Q2C) ***30*** *with* ***9e****.*

(33): 20 μL Ub(2-76)(Q2C) **30** stock solution were diluted dropwise in 16.8 μL TDW and waiting 0.5 h for folding, then diluted in 50 μL PB buffer (200 mM, pH 8), after that add 6 μL **9e**, 3.6 μL Cu(OTf)$_2$, 3.6 μL bipyridine and mixtures. The reaction mixtures were incubated at 37 ^{o}C for 3 h. The reaction progress was monitored by HPLC **method F**, conversion: 50%. The product was characterized by ESI-MS (The product was characterized by ESI-MS (mass obs. 8581.41 Da, calc. 8581.90 Da). The results are shown in Fig. S97.

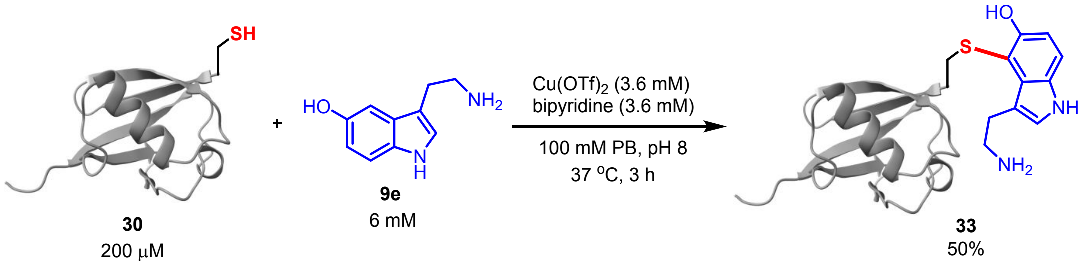

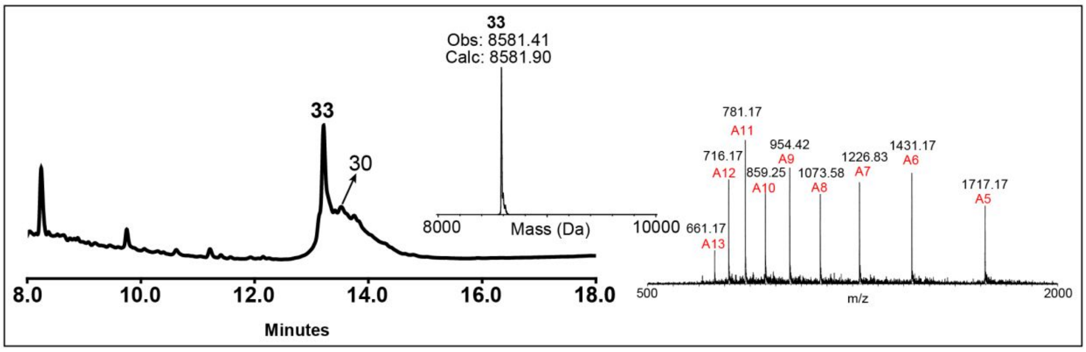

Figure S97. The modification of Ub(2-76)(Q2C) **30** with **9e**.

4. The modification of expressed Ub(1-76)(M1S, Q2C) ***34*** *with* ***9e****.*

(36): 20 μL expressed Ub(1-76)(M1S, Q2C) **34** stock solution were diluted dropwise in 27 μL TDW and waiting 0.5 h for folding, then diluted in 25 μL PB buffer (400 mM, pH 8), after that add 12 μL **9e**, 8 μL $Cu(OTf)_2$, 8 μL bipyridine and mixtures. The reaction mixtures were incubated at 37 °C for 4 h. The reaction progress was monitored by HPLC **method F**, conversion: 50%. The product was characterized by ESI-MS (The product was characterized by ESI-MS (mass obs. 8668.72 Da, calc. 8669.96 Da). The results are shown in Fig. S98.

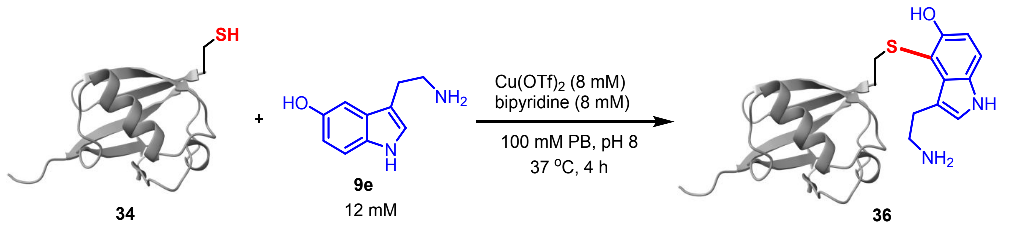

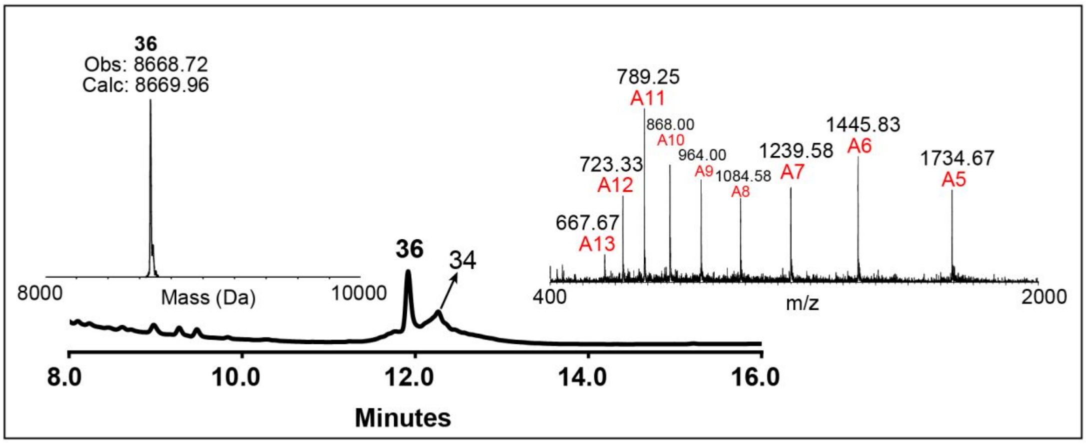

Figure S98. The modification of expressed Ub(1-76)(M1S, Q2C) **34** with **9e**.

5. The modification of expressed wild-type Ub(1-76)(M1S) ***35*** *with* ***9e****.*

(35): 20 μL expressed wild-type Ub(1-76)(M1S) **35** stock solution were diluted dropwise in 27 μL TDW and waiting 0.5 h for folding, then diluted in 25 μL PB buffer (400 mM, pH 8), after that add 12 μL **9e**, 8 μL $Cu(OTf)_2$, 8 μL bipyridine and mixtures. The reaction mixtures were incubated at 37 °C for 4 h. There is no functionalization of protein **35**. The reaction progress was monitored by HPLC **method F**. The product was characterized by ESI-MS (The start material was characterized by ESI-MS (mass obs. 8520.21 Da, calc. 8520.75 Da). The results are shown in Fig. S99.

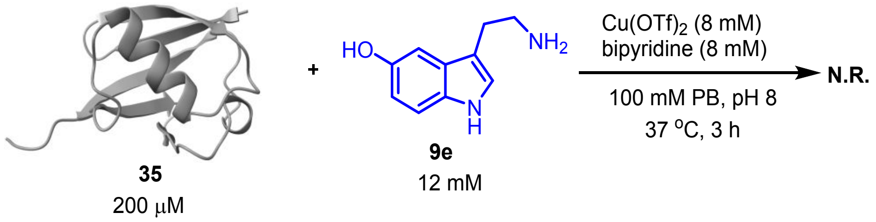

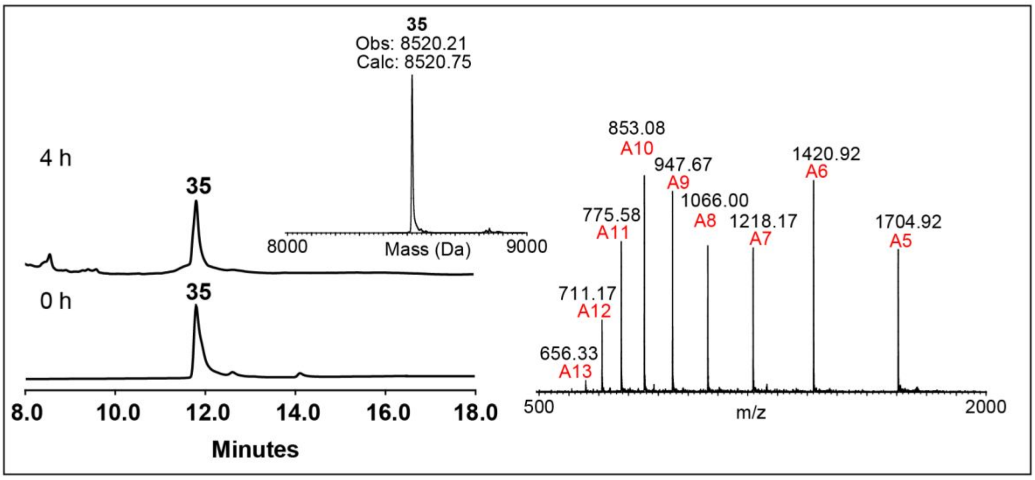

Figure S99. The modification of expressed wild-type Ub(1-76)(M1S) **35** with **9e**.

6. The modification of Sec-ZHER2 affibody ***37*** *with* ***9e****.*

(39): 5 μL Sec-ZHER2 affibody **37** (dimer) stock solution were diluted dropwise in 39 μL TDW and waiting 0.5 h for folding, then diluted in 50 μL Tris buffer (200 mM, pH 8), after that add 2μL **9e**, 2μL $Cu(OTf)_2$, 2μL bipyridine and mixtures. The reaction mixtures were incubated at 37 °C for 4 h. The reaction progress was monitored by HPLC **method E**, conversion: 80%. The product was characterized by ESI-MS (mass obs. 7028.47, calcDa. 7028.86 Da). The results are shown in Fig. S100.

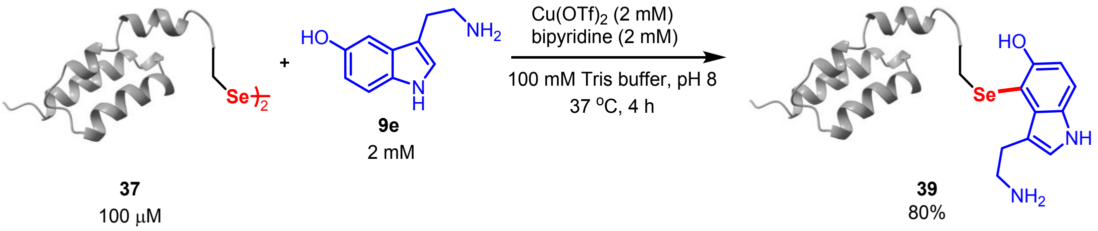

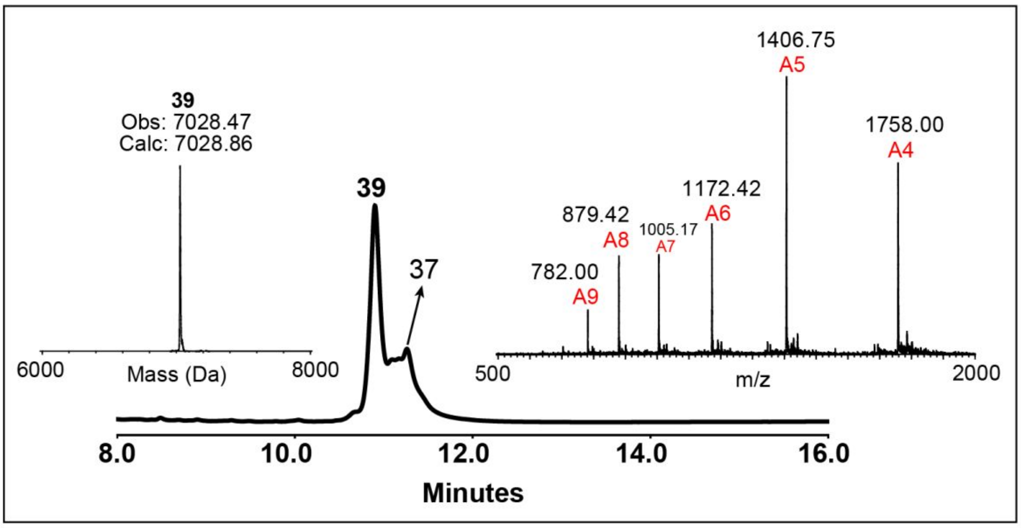

Figure S100. The modification of protein Sec-ZHER2 affibody **37** with **9e**.

*7. The modification of Sec-ZHER2 affibody **37** with **9d**.*

(40): 5 µL Sec-ZHER2 affibody **37** (dimer) stock solution were diluted dropwise in 39 µL TDW and waiting 0.5 h for folding, then diluted in 50 µL Tris buffer (200 mM, pH 8), after that add 2µL **9e**, 2µL Cu(OTf)$_2$, 2µL bipyridine and mixtures. The reaction mixtures were incubated at 37 ^{o}C for 12 h. The reaction progress was monitored by HPLC **method E**, conversion: 40%. The product was characterized by ESI-MS (mass obs. 8300.70 Da, calc. 8301.90 Da). The results are shown in Fig. S101. # is deselenization form of **37**.

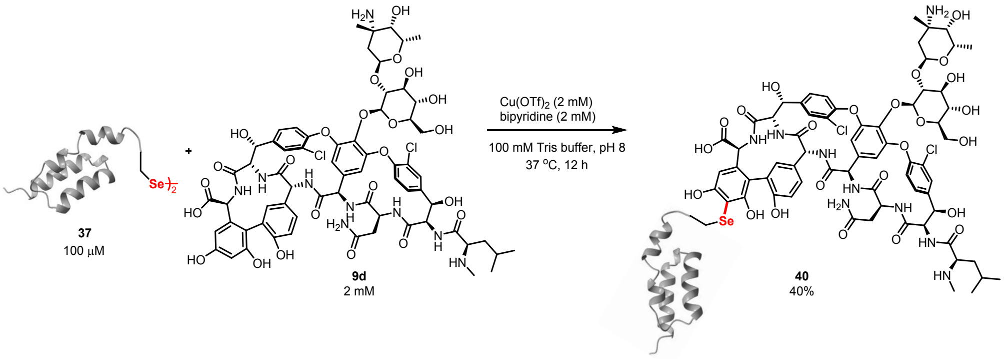

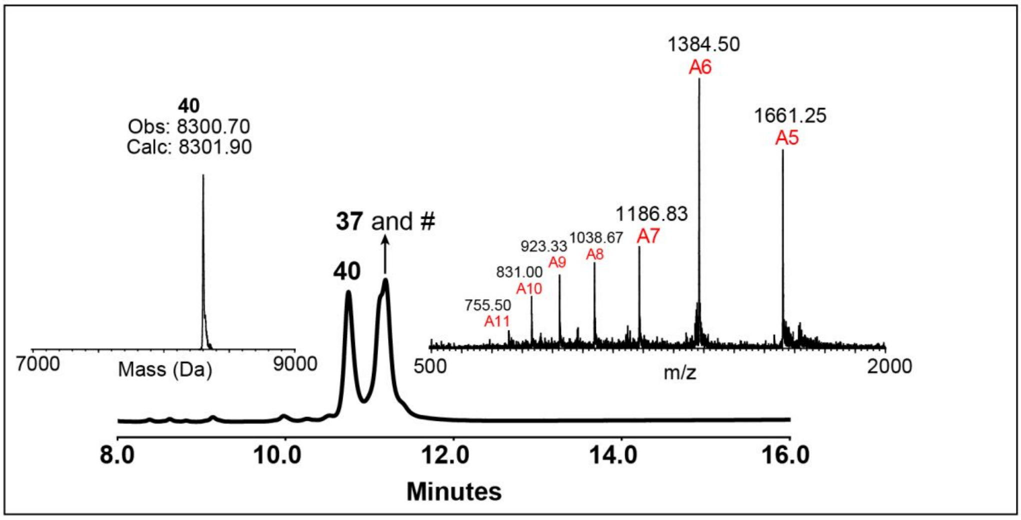

Figure S101. The modification of Sec-ZHER2 affibody **37** with **9d**.

*8. The modification of Cys-ZHER2 affibody **38** with **9e**.*

(41): 20 µL Cys-ZHER2 affibody **38** stock solution were diluted dropwise in 22 µL TDW and waiting 0.5 h for folding, then diluted in 10 µL Tris buffer (1000 mM, pH 8), after that add 16µL **9e**, 16µL $Cu(OTf)_2$, 16µL bipyridine and mixtures. The reaction mixtures were incubated at 37 ^oC for 3 h. The reaction progress was monitored by HPLC **method F**, conversion: 70%. The product was characterized by ESI-MS (mass obs. 6981.42 Da, calc. 6981.95 Da). The results are shown in Fig. S102. **38’** is dimer of **38**.

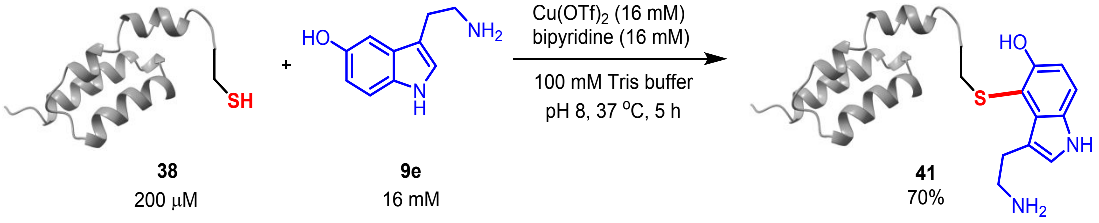

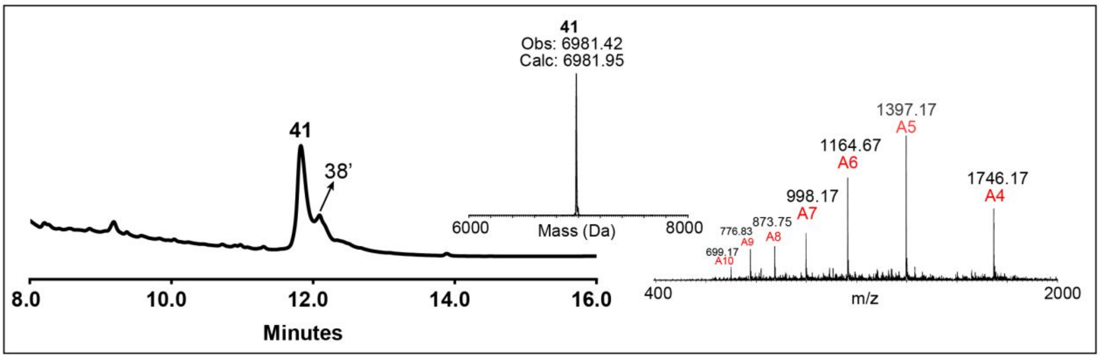

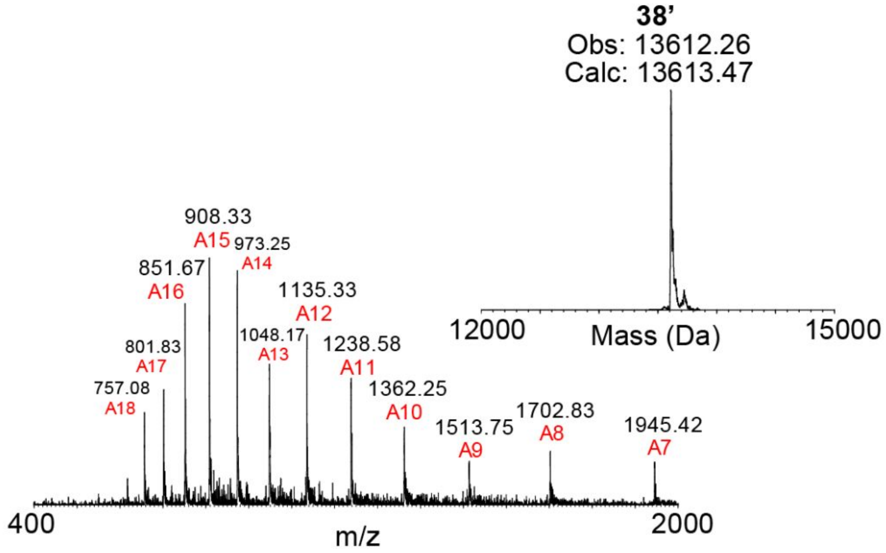

Figure S102. The modification of Cys-ZHER2 affibody **38** with **9e**.

11. EPR Experiment

Continuous wave (CW) electron paramagnetic resonance (EPR) spectra were recorded using a Bruker EMXplus spectrometer operating at X-band (9.5 GHz). The spectrometer is equipped with a Bruker high-sensitivity probehead. All experiments were performed at room temp. The solutions were mixed in Eppendorf tubes, and then a small amount was placed inside a glass capillary (O.D. 0.8 mm, I.D 0.6 mm). Each capillary was placed inside a 4mm EPR tube for the experiments.

For CW-EPR spectra of the DMPO spin adducts, the experimental conditions were: room temp, microwave frequency of 9.367 GHz, modulation frequency 100 kHz, modulation amplitude 0.25 G, receiver gain 30 dB, conversion time 3 ms, central field 3335 G, scan width 250 G, 10000 points and 70 scans.

Spin-trapping EPR experiments were performed, using 5,5-Dimethyl-1-pyrroline N-oxide (DMPO) as the spin trap, with a concentration of 150 mM in DDW.
DMPO for ESR spectroscopy was purchased from Sigma-Aldrich, and used without further purification. DMPO was added last to each sample, unless otherwise specified.

First, as control experiments, we recorded the EPR spectrum from DMPO and Cu, and then DMPO, Cu and phloroglucinol. Finally, we recorded the EPR spectra from samples that contained DMPO, Cu, phloroglucinol and a peptide. Three peptides were studied, one that contained Cys (peptide **4** LGCALG-NH_2), one that contained Sec (peptide **1** LGUALG-NH_2), and one that contained Ala (peptide **12** LGAALG-NH_2).

Fitting of the experimental EPR spectra was performed using EasySpin[5]. Each EPR spectrum was fitted with simulated lines for 3 or 4 spin adducts. The fitting parameters are given in Table S2.

Table S2. Table with EPR simulation parameters for the various spin adducts observed in this work.

**Radical**	**g value**	**A value [Gauss]**	**Nuclei in the simulation**	**Lorentzian broadening [Gauss]**
**OH**	2.00635	14.8, 14.8	^{14}N, ^{1}H	0.10
**CH_3**	2.00635	15.7, 22.7	^{14}N, ^{1}H	0.15
**N**	2.00635	15.2	^{14}N	0.12
**Aromatic carbon**	2.00615	17, 29	^{14}N, ^{1}H	0.10

In the control experiment, when the sample contained DMPO and Cu, three signals were observed, corresponding to DMPO-OH spin adduct, DMPO-CH_3 adduct as well as a nitroxide-like radical which corresponds to a broken DMPO ring[6]. These radicals were formed as a result of the sensitivity of DMPO to ambient light in the room, and

its reaction with water and copper. The signals from DMPO-OH and the nitroxide-like radical were very small.

Next, we recorded the EPR spectrum from a sample of DMPO, Cu and phloroglucinol. The same three radicals observed previously were observed here as well as an additional radical. The new spin adduct was identified as DMPO-aromatic carbon, based on the relatively large hyperfine interaction with the ^{1}H nucleus, which is similar to the hyperfine interactions listed in the literature for DMPO-phenyl radical spin adducts[6]. This aromatic carbon spin adduct was formed as a result of the interaction of Cu with the phloroglucinol.

Finally, we recorded the EPR spectra of DMPO, Cu, phloroglucinol and a peptide. Three peptides were studied, one that contained Cys, one that contained Sec, and one that contained Ala. The addition of the Ala containing peptide did not change the EPR spectrum, and a large signal from the DMPO-aromatic carbon spin adduct was still observed. However, when the peptide contained Cys or Sec, the signal of the DMPO-aromatic carbon spin adduct was significantly reduced. These results suggest that the Cys and Sec containing peptides react quickly with the aromatic carbon radical, and therefore reduce the amount of DMPO-aromatic carbon spin adduct observed.

The spectra are shown below. The relative signal intensity of each sample is given in Table S3.

Table S3. Table with the relative signal intensity for the various spin adducts observed in this work.

**Sample**	**signal of OH spin adduct (%)**	**signal of CH_3 spin adduct (%)**	**signal of nitroxide-like radical (%)**	**signal of aromatic carbon spin adduct (%)**
**DMPO and Cu**	7.9200	85.5742	6.5058	0
**DMPO, Cu and phloroglucinol**	6.6048	58.3870	6.9755	28.0327
**DMPO, Cu, phloroglucinol and Cys-containing peptide**	7.3246	72.7527	5.4151	14.5076

**DMPO, Cu, phloroglucinol and Sec-containing peptide**	6.6255	78.0952	6.5308	8.7485
**DMPO, Cu, phloroglucinol and Ala-containing peptide**	6.1968	71.1226	5.4974	17.1832

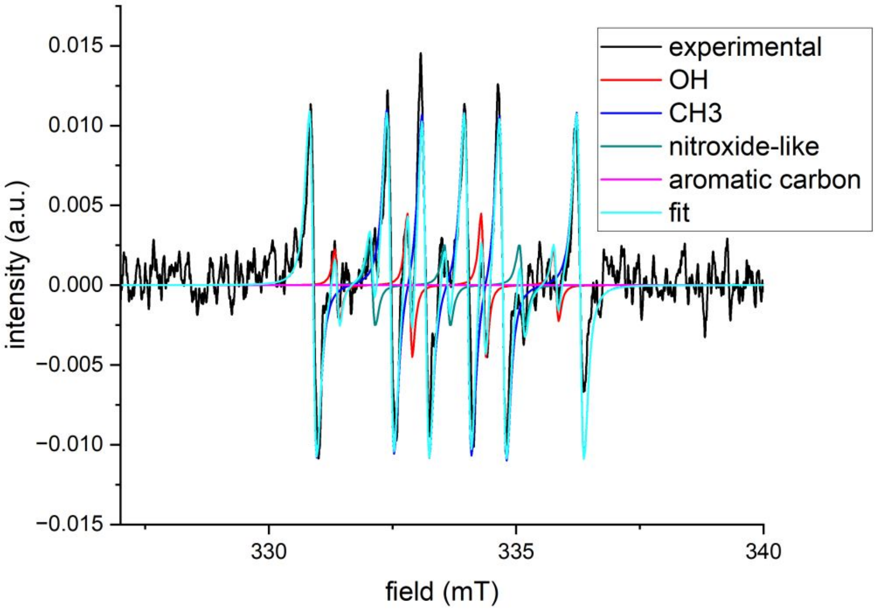

Figure S103. EPR spectrum of Cu and DMPO. The experimental line is shown in black, and the overall fit in cyan. The fit is made up of the following spin-adduct components: DMOH-OH (red), DMPO-CH_3 (blue), nitroxide-line (green) and DMPO-aromatic carbon (magenta). The fitting parameters are given in Table S3. Note that the spectrum is not normalized.

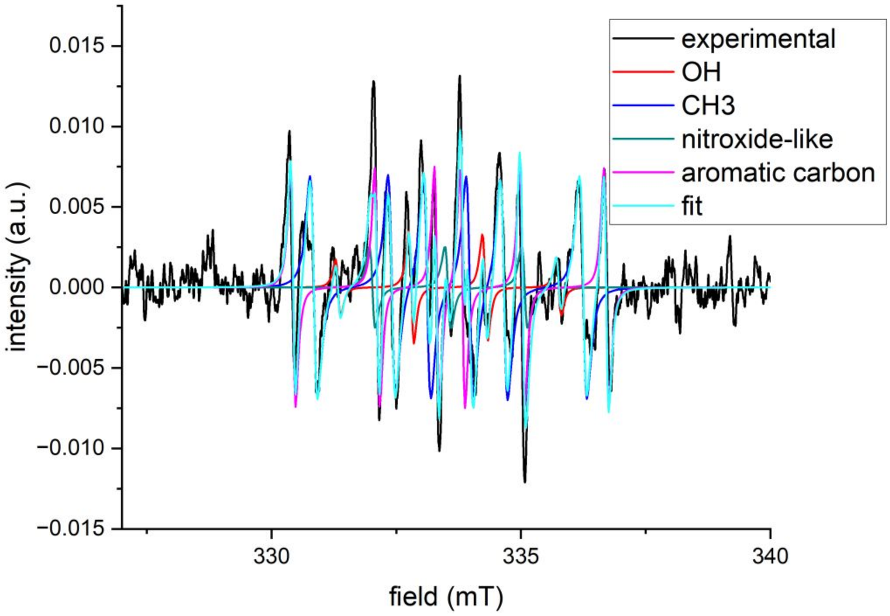

Figure S104. EPR spectrum of Cu, phloroglucinol and DMPO. The experimental line is shown in black, and the overall fit in cyan. The fit is made up of the following spin-adduct components: DMOH-OH (red), DMPO-CH_3 (blue), nitroxide-line (green) and DMPO-aromatic carbon (magenta). The fitting parameters are given in Table S3. Note that the spectrum is not normalized.

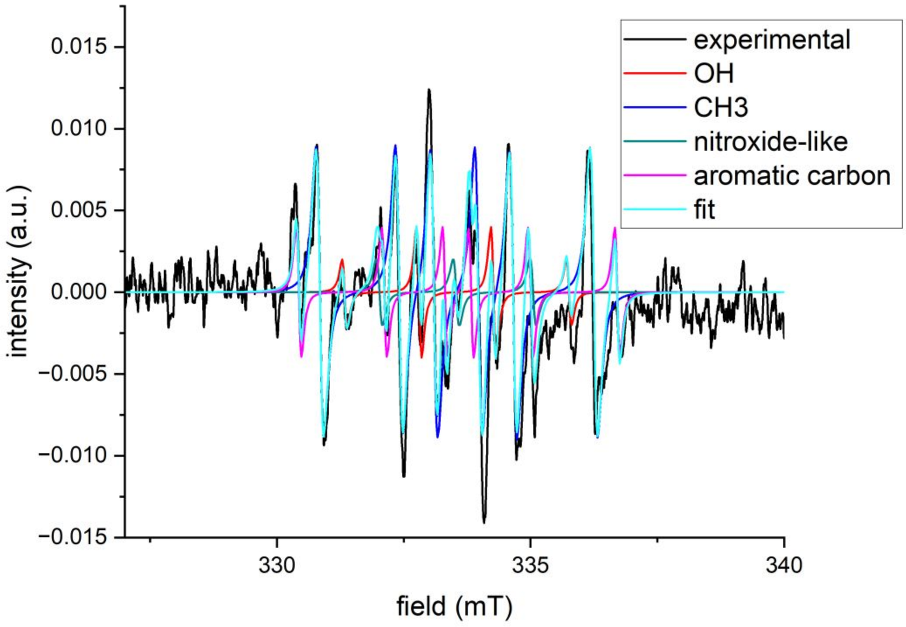

Figure S105. EPR spectrum of Cu, phloroglucinol, Cys-containing peptide and DMPO. The experimental line is shown in black, and the overall fit in cyan. The fit is made up

of the following spin-adduct components: DMOH-OH (red), DMPO-CH_3 (blue), nitroxide-line (green) and DMPO-aromatic carbon (magenta). The fitting parameters are given in Table S3. Note that the spectrum is not normalized.

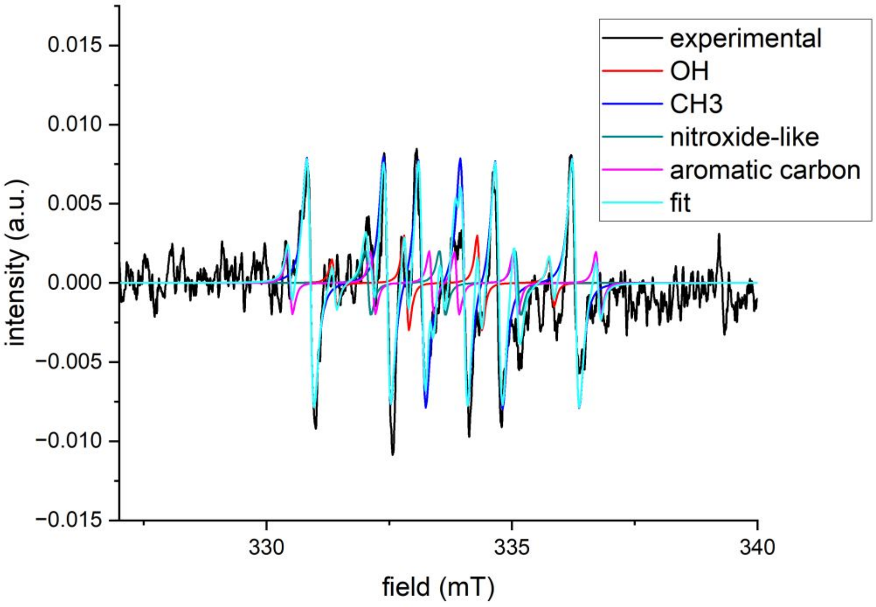

Figure S106. EPR spectrum of Cu, phloroglucinol, Sec-containing peptide and DMPO. The experimental line is shown in black, and the overall fit in cyan. The fit is made up of the following spin-adduct components: DMOH-OH (red), DMPO-CH_3 (blue), nitroxide-line (green) and DMPO-aromatic carbon (magenta). The fitting parameters are given in Table S3. Note that the spectrum is not normalized.

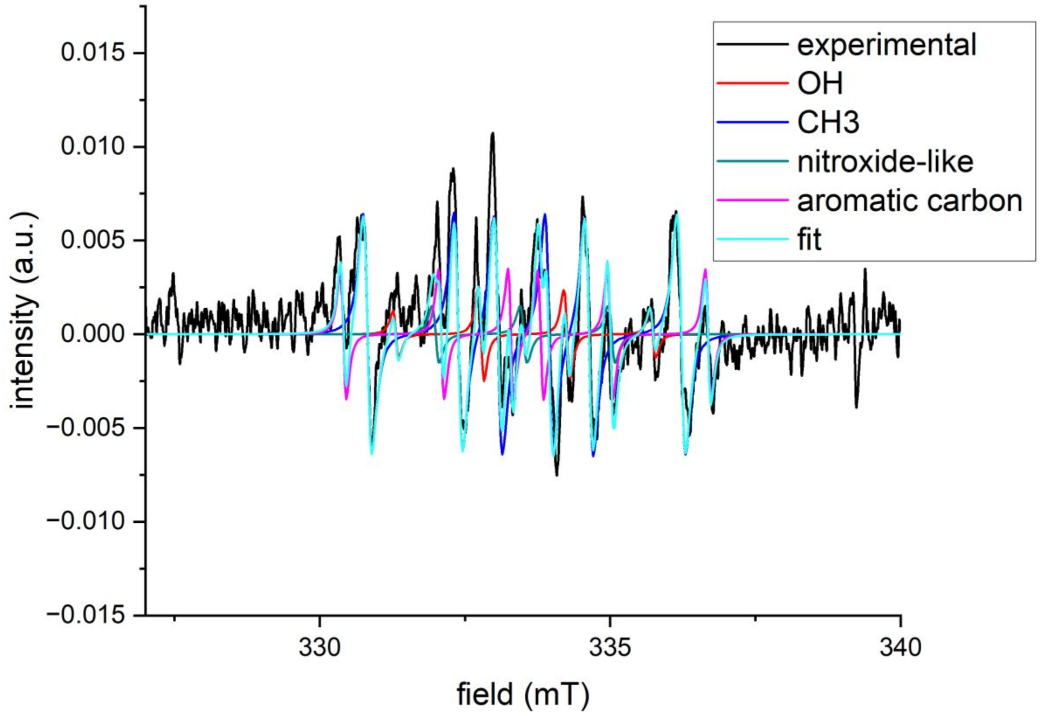

Figure S107. EPR spectrum of Cu, phloroglucinol, Ala-containing peptide and DMPO. The experimental line is shown in black, and the overall fit in cyan. The fit is made up of the following spin-adduct components: DMOH-OH (red), DMPO-CH_3 (blue), nitroxide-line (green) and DMPO-aromatic carbon (magenta). The fitting parameters are given in Table S3. Note that the spectrum is not normalized.

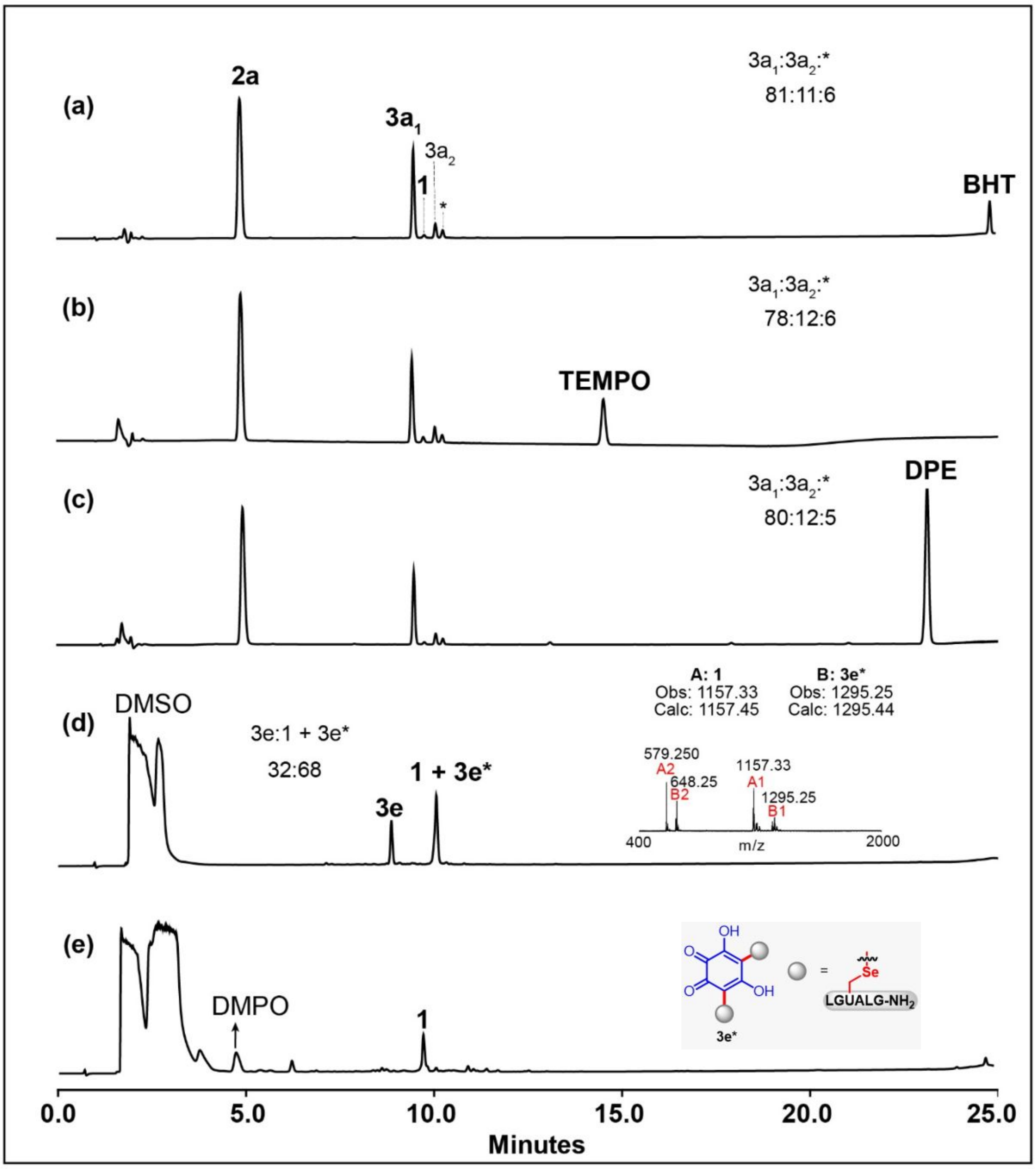

Figure S108. The HPLC of the modification of peptide **1** with **2a (resorcinol)** or **2e** **(**phloroglucinol) in the absence or presence of radical scavengers under standard conditions (standard conditions: 1 mM peptide **1**, 5 mM **2a** or **2e** and 1 mM $Cu(OTf)_2$ in 10 mM phosphate buffer). The reaction progress was monitored by HPLC **method A**. (**a**) The HPLC of the modification of peptide **1** with **2a** in the presence of 5 equiv BHT (butylated hydroxytoluene) under standard conditions. (**b**) The HPLC of the modification of peptide **1** with **2a** in the presence of 5 equiv TEMPO (2,2,6,6-Tetramethylpiperidin-1-oxyl) under standard conditions. (**c**) The HPLC of the modification of peptide **1** with **2a** in the presence of 5 equiv DPE (1,1-diphenylethylene) under standard conditions. (**d**) The modification reaction of peptide **1** with **2e** in DMSO instead of phosphate buffer in the absence of DMPO (5-Diethoxyphosphoryl-5-methyl-1-pyrroline-N-oxyl) under standard conditions. (**e**). The modification reaction of peptide **1** with **2e** in DMSO instead of phosphate buffer in the presence of 150 equiv DMPO.

12. Density Functional Theory (DFT) Calculations

All the calculations were carried out by the Gaussian16 package.[7] The (u)B3LYP hybrid functional was applied for all calculations in combination with the D3BJ dispersion correction.[8] For geometry optimization, the def2-SVP basis set was used.[9] The frequencies were computed analytically at the same level of theory as the geometry optimizations to identify the nature of all stationary points being either minimum (no imaginary frequency) or transition state (only one imaginary frequency) and also to obtain the Gibbs free energy correction at 298.15 K. To obtain better accuracy, the final energies for the fully optimized structures were calculated with the def2-TZVP basis set. Solvation effects were included by employing the PCM solvent model for water in all calculations.[10] These methods have been widely used to rationalize and predict reactivity and selectivity in copper-catalyzed reactions.[11] The final free energies reported in the article are the large basis set single-point energies corrected by the Gibbs free energy correction.

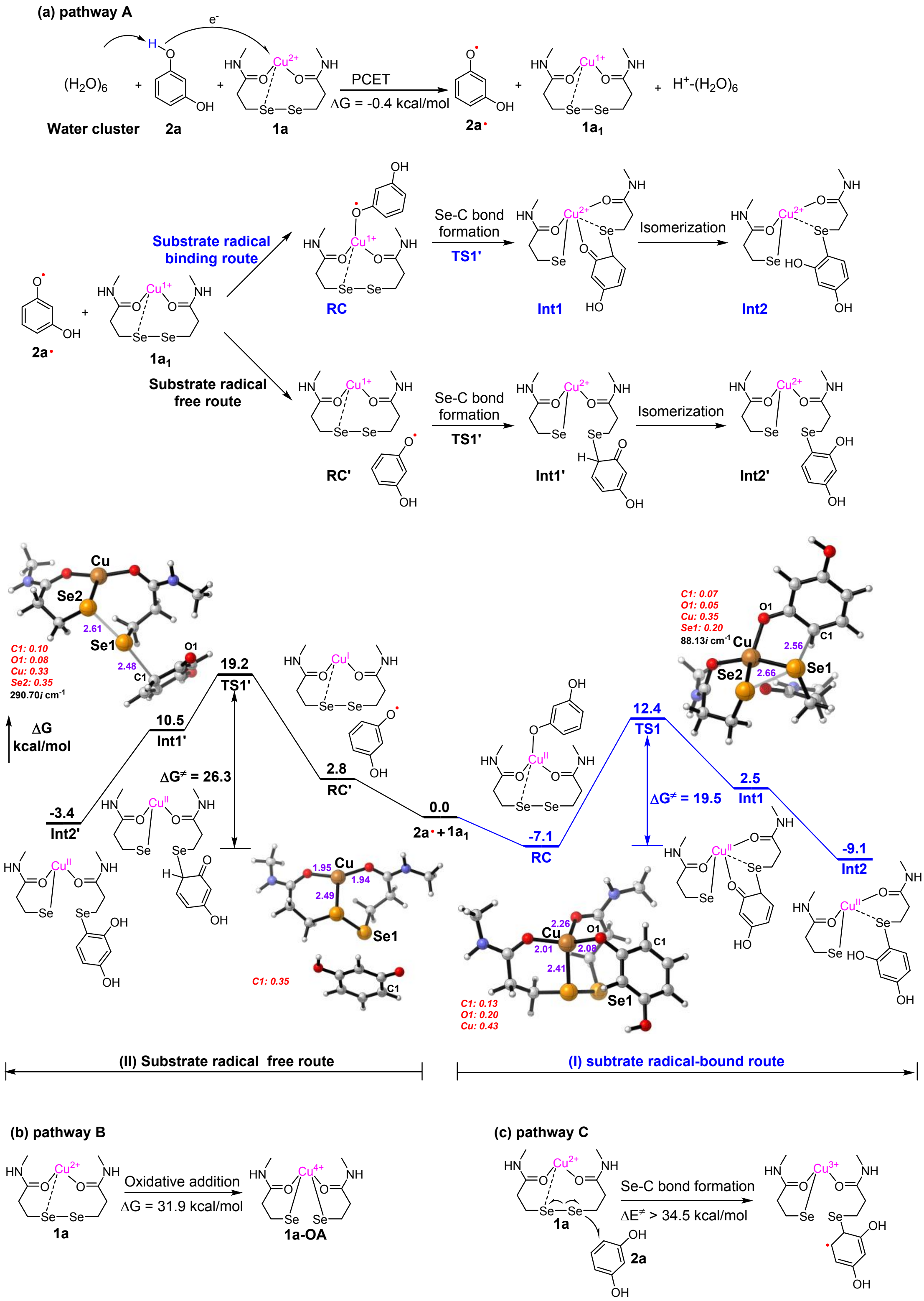

Scheme S8. Proposed three possible reaction mechanisms.

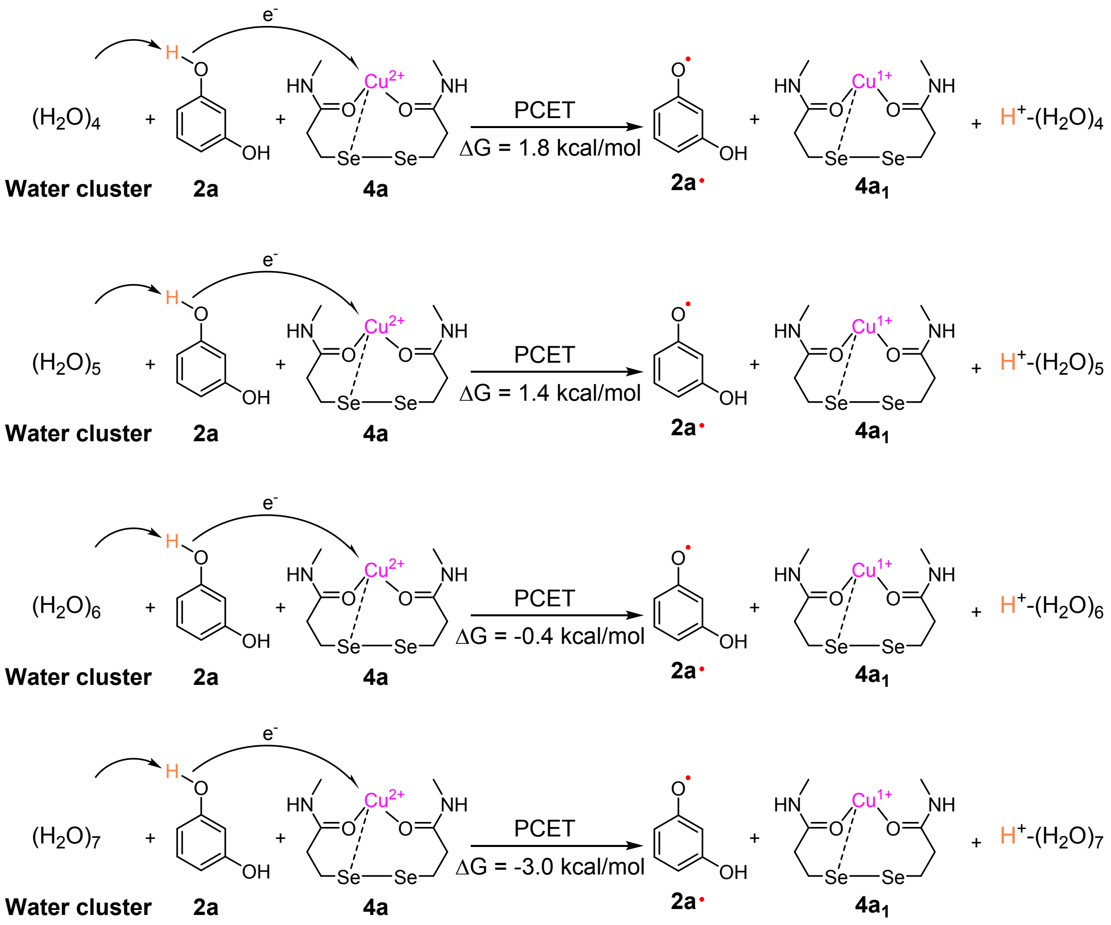

Scheme S9. Calculated PCET process energies of different water models.

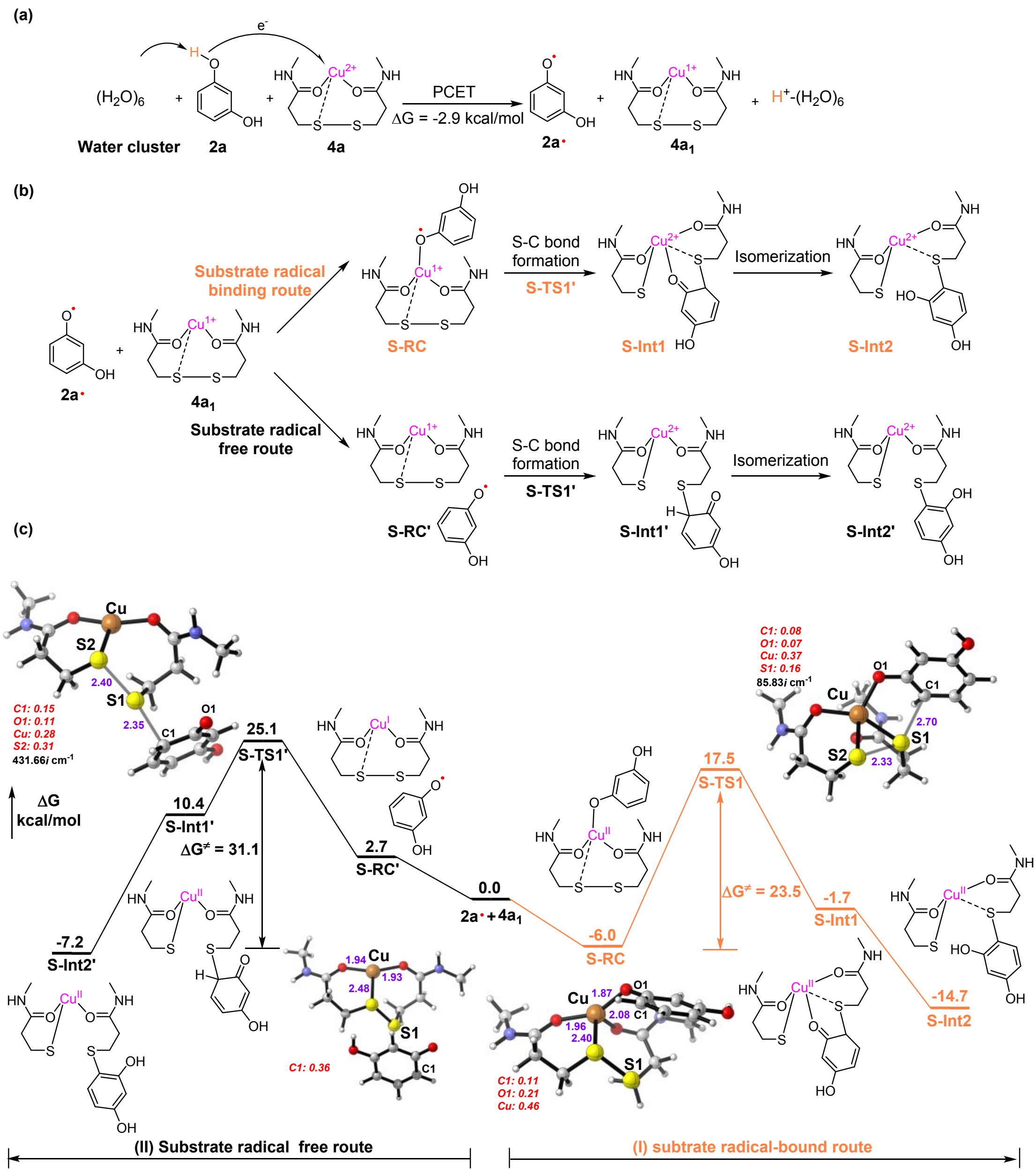

Scheme S10. Proposed reaction mechanisms for Cys dimer. (**a**) Calculated PCET process energy of Cys dimer. (**b**) Proposed mechanism for Cys dimer. (**c**) The DFT-calculated energy profile on Cu(I)-mediated modification reaction of S-peptide. Distances are given in Å. The spin population on key atoms is shown in *red* italics. The imaginary frequencies for transition states are also shown.

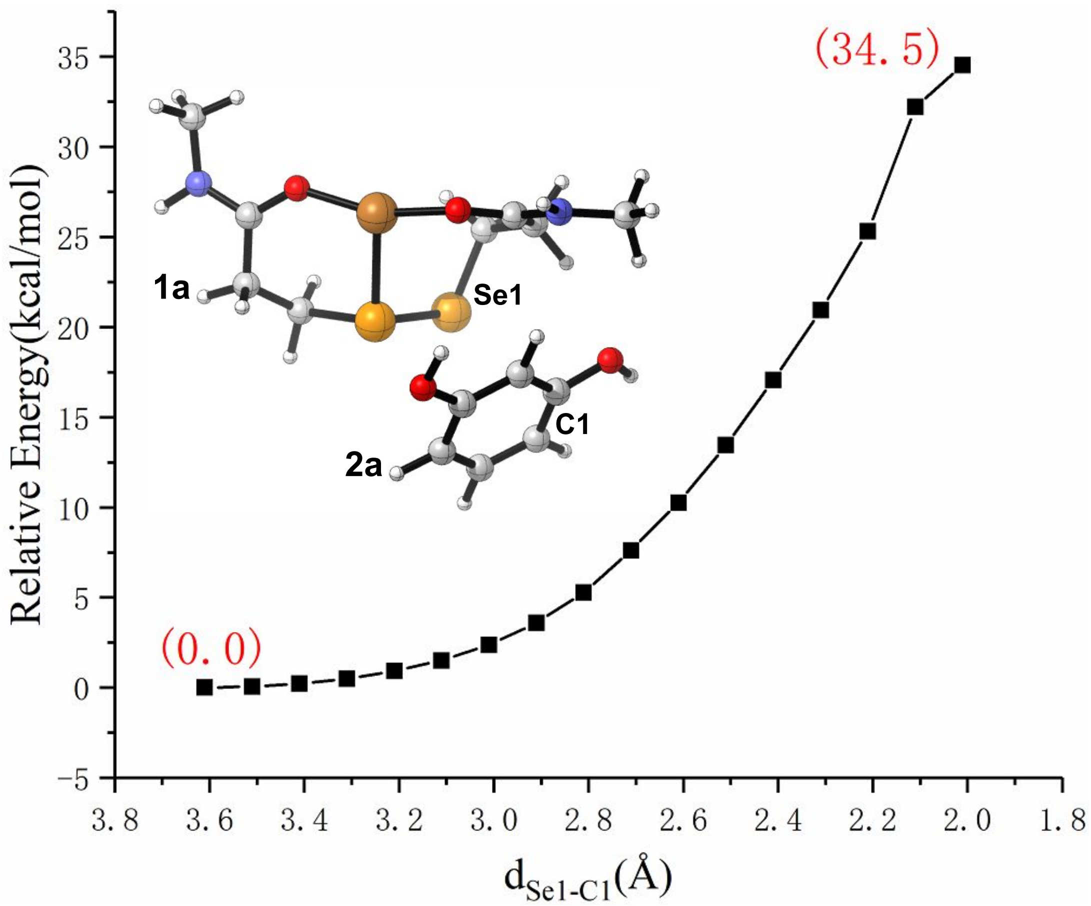

Figure S109. Scanned energy profiles for Se–C bond formation using the Se1–C1 as the reaction coordinate from 1a and 1b complex. Energies are given in kcal/mol relative to the energy of the complex.

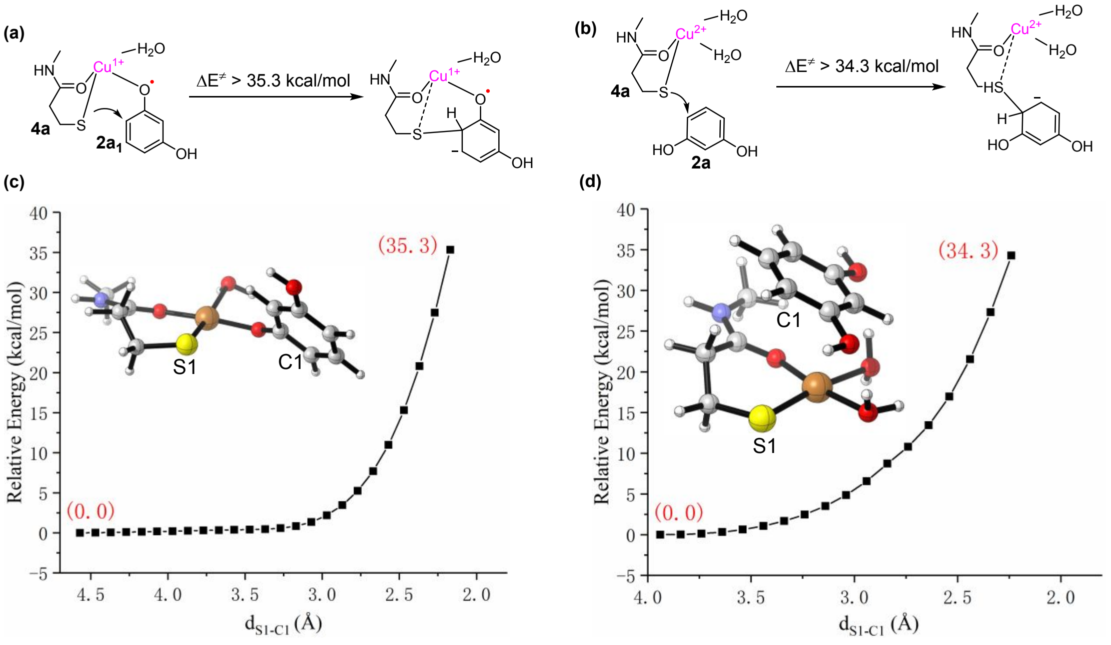

Figure S110. Proposed possible reaction mechanism for free cysteine. (**a**) Proposed 4a coordinated Cu(I) and 2a radical complex structure; (**b**) Proposed 4a coordinated Cu(I)

and 2a complex structure; (**c**) Scanned energy profiles for S–C bond formation using the S1–C1 as the reaction coordinate from 4a coordinated Cu(I) and 2a$_1$ complex. Energies are given in kcal/mol relative to the energy of the complex; (**d**) Scanned energy profiles for S–C bond formation using the S1–C1 as the reaction coordinate from 4a coordinated Cu(I) and 2a complex. Energies are given in kcal/mol relative to the energy of the complex.

Table S4. Optimized structures.

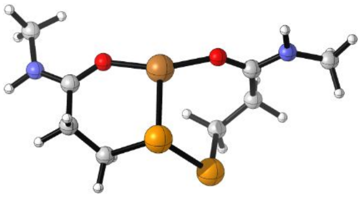 1a	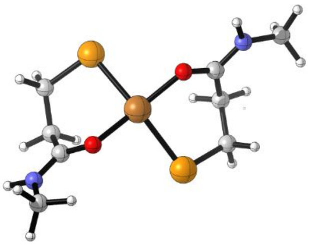 1a-OA	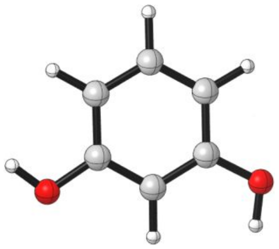 2a
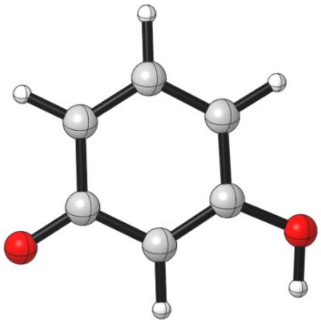 2a·	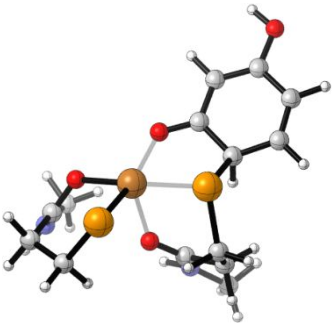 Int1	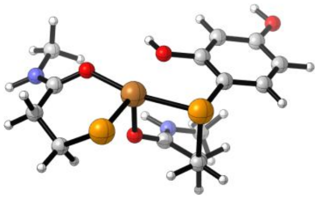 Int2
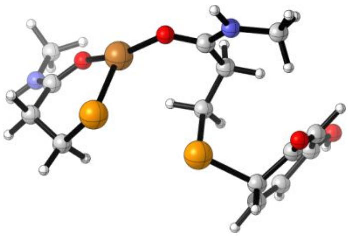 Int1’	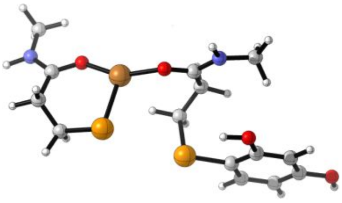 Int2’	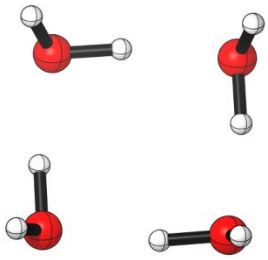 $(H_2O)_4$
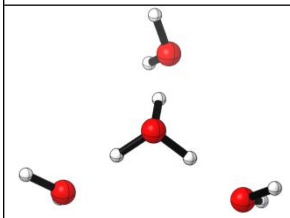 H^+-$(H_2O)_4$	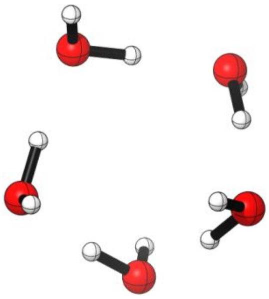 $(H_2O)_5$	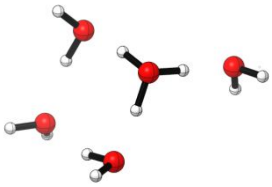 H^+-$(H_2O)_5$

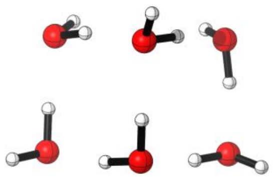

$(H_2O)_6$

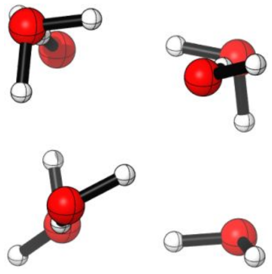

H^+-$(H_2O)_6$

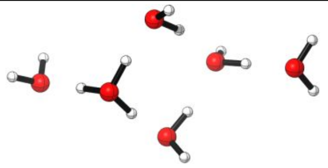

$(H_2O)_7$

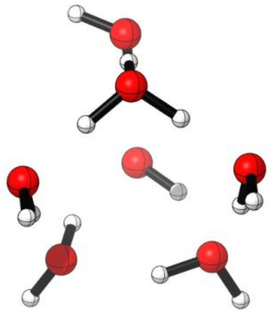

H^+-$(H_2O)_7$

1. Absolute energies and energy corrections

Table S5. Calculated energies (in hartree) and imaginary frequencies.

Stationary point	Gibbs free energy correction	Single point energy	Gibbs free energies (G)	Imaginary frequencies
1a	0.198015	-7018.261045	-7018.06303	
1a-OA	0.194597	-7018.206754	-7018.012157	
1b	0.078727	-382.8811562	-382.8024292	
1b·	0.06501	-382.2369404	-382.1719304	
2a	0.193808	-7018.4662	-7018.272392	
RC	0.281969	-7400.730857	-7400.448888	
TS1	0.285172	-7400.703046	-7400.417874	88.13*i*
Int1	0.284226	-7400.717908	-7400.433682	
Int2	0.286081	-7400.738153	-7400.452072	
RC’	0.275224	-7400.708335	-7400.433111	
TS1’	0.283299	-7400.690285	-7400.406986	290.70*i*
Int1’	0.281445	-7400.702281	-7400.420836	
Int2’	0.280482	-7400.72349	-7400.443008	
S-RC	0.28673	-3393.98156	-3393.69483	
S-TS1	0.289184	-3393.946629	-3393.657445	85.83*i*
S-Int1	0.28949	-3393.977587	-3393.688097	
S-Int2	0.288286	-3393.997101	-3393.708815	
S-RC’	0.278067	-3393.959115	-3393.681048	
S-TS1’	0.286107	-3393.931498	-3393.645391	431.66*i*
S-Int1’	0.286789	-3393.955569	-3393.66878	
S-Int2’	0.285919	-3393.982748	-3393.696829	

2. Cartesian coordinates of Optimized Structures

1a

Center Number	Atomic Number	Atomic Type	Coordinates (Angstroms) X	Y	Z
1	6	0	3.163912	-1.052502	-0.460883
2	1	0	3.203222	-1.014331	-1.563663
3	6	0	1.936999	-1.823073	0.016859
4	6	0	3.254772	0.371529	0.041108
5	1	0	2.058181	-2.895015	-0.185089
6	1	0	1.722819	-1.683356	1.083732
7	8	0	2.267817	1.164001	0.004163
8	7	0	4.418000	0.790165	0.497729
9	1	0	5.184147	0.122647	0.508939
10	6	0	4.695800	2.141485	0.964243
11	1	0	5.124991	2.099191	1.974814
12	34	0	0.313363	-1.313425	-1.009560
13	6	0	-2.618020	0.614238	1.242477
14	1	0	-2.659393	1.394617	2.026032
15	6	0	-1.545435	-0.387521	1.635267
16	6	0	-2.452099	1.349190	-0.066581
17	1	0	-0.542348	0.043467	1.750718
18	1	0	-1.803326	-0.828002	2.608288
19	8	0	-1.336518	1.525042	-0.639706
20	7	0	-3.523890	1.886298	-0.623443
21	1	0	-3.345817	2.403989	-1.482368
22	6	0	-4.890504	1.852166	-0.114946
23	1	0	-4.951394	2.284887	0.893835
24	34	0	-1.429745	-1.969087	0.436532
25	29	0	0.449915	1.044721	-0.385870
26	1	0	-5.516772	2.447726	-0.788285
27	1	0	-5.279960	0.823695	-0.093229
28	1	0	5.412680	2.631570	0.289247
29	1	0	3.761754	2.712332	0.983556
30	1	0	-3.601066	0.125539	1.258239
31	1	0	4.057821	-1.609204	-0.145273

1a-OA

Center Number	Atomic Number	Atomic Type	Coordinates (Angstroms) X	Y	Z
1	6	0	2.861711	-0.017176	-1.102100
2	1	0	2.345682	0.674093	-1.786968
3	6	0	2.099510	-1.338213	-0.999834
4	6	0	3.022553	0.683151	0.225192
5	1	0	2.063962	-1.835572	-1.981785
6	1	0	2.582715	-2.043671	-0.304069
7	8	0	2.020442	1.103008	0.871624
8	7	0	4.240327	0.844442	0.710886
9	1	0	5.015323	0.465168	0.174868
10	6	0	4.542543	1.496551	1.976581
11	1	0	4.788448	0.748417	2.745615

12	34	0	0.232045	-1.255440	-0.429585
13	6	0	-2.236893	2.557513	-1.244116
14	1	0	-1.594297	1.969725	-1.917501
15	6	0	-1.507134	3.820173	-0.777520
16	6	0	-2.640363	1.654965	-0.104971
17	1	0	-1.262367	4.461650	-1.637529
18	1	0	-2.120409	4.424771	-0.088418
19	8	0	-1.762140	1.055487	0.580562
20	7	0	-3.915227	1.489751	0.203197
21	1	0	-4.086296	0.862732	0.987536
22	6	0	-5.073751	2.091331	-0.451057
23	1	0	-5.156193	1.762390	-1.497044
24	34	0	0.189156	3.569833	0.163473
25	29	0	0.156637	1.095803	0.386453
26	1	0	-5.970349	1.763430	0.086649
27	1	0	-5.029596	3.188728	-0.413146
28	1	0	5.400371	2.168930	1.843091
29	1	0	3.668095	2.071942	2.299610
30	1	0	-3.109144	2.872806	-1.829792
31	1	0	3.848111	-0.227008	-1.540119

2a

Center Number	Atomic Number	Atomic Type	Coordinates (Angstroms) X	Y	Z
1	6	0	1.081113	1.172320	-0.000022
2	6	0	-0.184910	1.757065	-0.000047
3	6	0	-1.347239	0.979354	-0.000028
4	6	0	-1.232116	-0.418552	0.000013
5	6	0	0.033015	-1.023222	0.000041
6	6	0	1.185394	-0.228539	0.000026
7	1	0	1.990699	1.774941	-0.000036
8	1	0	-0.272862	2.846368	-0.000085
9	1	0	-2.334372	1.448425	-0.000047
10	1	0	0.102831	-2.113772	0.000077
11	8	0	2.430882	-0.767605	0.000044
12	1	0	2.369890	-1.733571	0.000093
13	8	0	-2.311952	-1.240139	0.000036
14	1	0	-3.122967	-0.711665	0.000060

2a·

Center Number	Atomic Number	Atomic Type	Coordinates (Angstroms) X	Y	Z
1	6	0	1.066654	1.158822	-0.000121
2	6	0	-0.197398	1.770793	0.000001
3	6	0	-1.347275	1.004254	0.000223
4	6	0	-1.268026	-0.453363	0.000078
5	6	0	0.053973	-1.050677	-0.000011
6	6	0	1.186236	-0.262490	-0.000157
7	1	0	1.981470	1.754236	-0.000286
8	1	0	-0.259592	2.861090	0.000000
9	1	0	-2.343378	1.450865	0.000020

10	1	0	0.116396	-2.141557	-0.000031
11	8	0	2.445497	-0.748166	0.000088
12	1	0	2.428663	-1.716973	0.000437
13	8	0	-2.306565	-1.153295	-0.000115

1a$_1$

Center Number	Atomic Number	Atomic Type	Coordinates (Angstroms) X	Y	Z
1	6	0	-3.109888	0.997095	-0.459793
2	1	0	-3.072501	0.947483	-1.562649
3	6	0	-1.887299	1.737308	0.092510
4	6	0	-3.289701	-0.419519	0.053529
5	1	0	-2.031412	2.821303	0.014726
6	1	0	-1.682372	1.481484	1.139726
7	8	0	-2.368046	-1.264332	0.094493
8	7	0	-4.514707	-0.760630	0.453275
9	1	0	-5.246719	-0.059347	0.435009
10	6	0	-4.836998	-2.097931	0.923786
11	1	0	-5.918078	-2.161724	1.093051
12	34	0	-0.251241	1.303521	-0.961162
13	6	0	2.708797	-0.727293	1.237179
14	1	0	2.879453	-1.456122	2.052539
15	6	0	1.591804	0.214039	1.660458
16	6	0	2.450716	-1.558439	-0.005414
17	1	0	0.606001	-0.271816	1.672313
18	1	0	1.786500	0.585563	2.675954
19	8	0	1.304849	-1.898753	-0.369266
20	7	0	3.512397	-1.997332	-0.687993
21	1	0	3.287798	-2.588933	-1.484080
22	6	0	4.916635	-1.760556	-0.386658
23	1	0	5.180626	-2.112704	0.621704
24	34	0	1.444510	1.872738	0.574642
25	29	0	-0.468615	-1.107191	-0.335940
26	1	0	5.520509	-2.317244	-1.112802
27	1	0	5.175097	-0.693819	-0.470654
28	1	0	-4.540618	-2.849633	0.177543
29	1	0	-4.310154	-2.321466	1.864418
30	1	0	3.649305	-0.170228	1.133606
31	1	0	-4.007382	1.585134	-0.218817

RC

Center Number	Atomic Number	Atomic Type	Coordinates (Angstroms) X	Y	Z
1	6	0	3.367197	-1.724611	0.340859
2	1	0	3.125334	-2.502137	-0.405292
3	6	0	2.227551	-1.565883	1.347121
4	6	0	3.726746	-0.462682	-0.420187
5	1	0	2.213571	-2.407618	2.049570
6	1	0	2.306752	-0.629800	1.914125
7	8	0	2.868464	0.305861	-0.910888
8	7	0	5.024277	-0.206543	-0.566075

9	1	0	5.685453	-0.860580	-0.160166
10	6	0	5.555062	0.920468	-1.314778
11	1	0	5.922648	0.598352	-2.301949
12	34	0	0.469281	-1.582564	0.426928
13	6	0	-0.905322	2.314075	1.680180
14	1	0	-0.872216	3.202478	2.335337
15	6	0	-0.183834	1.166514	2.375496
16	6	0	-0.299862	2.724243	0.351935
17	1	0	0.882392	1.146591	2.117918
18	1	0	-0.265777	1.265962	3.465838
19	8	0	0.752208	2.212865	-0.081875
20	7	0	-0.919641	3.696296	-0.324895
21	1	0	-0.479922	3.951964	-1.205184
22	6	0	-2.093329	4.440707	0.104618
23	1	0	-1.893680	5.018872	1.020098
24	34	0	-0.933936	-0.647253	2.073273
25	29	0	0.910281	0.371866	-1.029473
26	1	0	-2.371976	5.138633	-0.693291
27	1	0	-2.948367	3.773727	0.292462
28	1	0	4.758800	1.660416	-1.453692
29	1	0	6.385386	1.374204	-0.756700
30	1	0	-1.975239	2.094629	1.541913
31	1	0	4.253001	-2.090492	0.880278
32	6	0	-4.306483	-1.111790	-0.812283
33	6	0	-4.106430	0.271017	-0.796039
34	6	0	-2.894552	0.827569	-1.200115
35	6	0	-1.840721	-0.011481	-1.644580
36	6	0	-2.058793	-1.412585	-1.686687
37	6	0	-3.273317	-1.955459	-1.264774
38	1	0	-5.248324	-1.556632	-0.486911
39	1	0	-4.912995	0.923545	-0.452750
40	1	0	-2.726917	1.904067	-1.192163
41	1	0	-1.260252	-2.058222	-2.059834
42	8	0	-3.516345	-3.288976	-1.270712
43	1	0	-2.733117	-3.763625	-1.584763
44	8	0	-0.686870	0.507305	-2.004618

TS1

Center Number	Atomic Number	Atomic Type	Coordinates (Angstroms) X	Y	Z
1	6	0	-3.760331	-1.156413	0.125470
2	1	0	-3.886241	-2.188931	0.488984
3	6	0	-2.907661	-1.148475	-1.149711
4	6	0	-3.215139	-0.337569	1.281657
5	1	0	-3.462237	-1.617841	-1.972528
6	1	0	-2.634471	-0.125955	-1.438844
7	8	0	-2.107478	-0.579885	1.820961
8	7	0	-3.974278	0.652940	1.739207
9	1	0	-4.887435	0.802852	1.323875
10	6	0	-3.534616	1.539315	2.803714
11	1	0	-4.146881	2.448764	2.783776
12	34	0	-1.238299	-2.208332	-0.973838
13	6	0	0.060656	2.124750	-1.986052
14	1	0	-0.416650	2.723187	-2.782453

15	6	0	0.119891	0.692132	-2.483130
16	6	0	-0.735288	2.337007	-0.709650
17	1	0	-0.876544	0.300222	-2.715122
18	1	0	0.732325	0.629860	-3.390901
19	8	0	-1.331302	1.406202	-0.141961
20	7	0	-0.768571	3.584122	-0.220840
21	1	0	-1.309210	3.694950	0.632962
22	6	0	-0.138775	4.760955	-0.798911
23	1	0	-0.496273	4.950680	-1.822543
24	34	0	0.911887	-0.664630	-1.261128
25	29	0	-0.462714	-0.550716	0.699014
26	1	0	-0.393506	5.629398	-0.180381
27	1	0	0.957987	4.665220	-0.822408
28	1	0	-3.632953	1.061215	3.791244
29	1	0	-2.479630	1.799776	2.644087
30	1	0	1.067593	2.555052	-1.879276
31	1	0	-4.762461	-0.790095	-0.144666
32	6	0	4.752568	0.302273	-0.013679
33	6	0	3.669061	0.999558	-0.489170
34	6	0	2.394137	0.883228	0.134602
35	6	0	2.233286	0.069694	1.337467
36	6	0	3.385163	-0.602390	1.823467
37	6	0	4.603853	-0.498330	1.161071
38	1	0	5.733601	0.358415	-0.486623
39	1	0	3.776630	1.656669	-1.355320
40	1	0	1.658033	1.672309	-0.005816
41	1	0	3.284445	-1.228536	2.713108
42	8	0	5.707880	-1.142311	1.572653
43	1	0	5.521587	-1.652025	2.376236
44	8	0	1.081912	-0.077450	1.886927

Int1

Center Number	Atomic Number	Atomic Type	Coordinates (Angstroms) X	Y	Z
1	6	0	-4.067699	-0.292011	0.957875
2	1	0	-4.259857	-1.176639	1.585547
3	6	0	-3.664653	-0.737277	-0.452107
4	6	0	-3.007628	0.533550	1.652255
5	1	0	-4.529003	-1.209957	-0.939784
6	1	0	-3.362427	0.128245	-1.057755
7	8	0	-1.875945	0.070617	1.929120
8	7	0	-3.311989	1.791498	1.968086
9	1	0	-4.253828	2.121399	1.787048
10	6	0	-2.353608	2.708506	2.559565
11	1	0	-2.607922	3.734319	2.263129
12	34	0	-2.207548	-2.073989	-0.527714
13	6	0	0.449212	1.423384	-2.496250
14	1	0	0.043783	1.847666	-3.431918
15	6	0	0.368101	-0.084870	-2.629184
16	6	0	-0.334623	2.031080	-1.343806
17	1	0	-0.671418	-0.438316	-2.663063
18	1	0	0.891562	-0.409351	-3.535813
19	8	0	-1.010690	1.343184	-0.564739

20	7	0	-0.267855	3.365102	-1.213018
21	1	0	-0.806250	3.745525	-0.439573
22	6	0	0.457728	4.288168	-2.070353
23	1	0	0.113239	4.230913	-3.114675
24	34	0	1.110180	-1.202575	-1.159768
25	29	0	-0.615387	-0.603532	0.522848
26	1	0	0.282717	5.307959	-1.708001
27	1	0	1.542171	4.097382	-2.048224
28	1	0	-2.356383	2.644639	3.659878
29	1	0	-1.350484	2.456226	2.193731
30	1	0	1.492581	1.776774	-2.471309
31	1	0	-5.005148	0.283441	0.898600
32	6	0	4.828085	-0.268067	0.086939
33	6	0	3.800140	-0.146178	-0.772146
34	6	0	2.407948	0.047746	-0.289731
35	6	0	2.184282	-0.027354	1.216830
36	6	0	3.328302	-0.083838	2.058212
37	6	0	4.593084	-0.221283	1.521950
38	1	0	5.857475	-0.389710	-0.252457
39	1	0	3.969281	-0.148696	-1.850818
40	1	0	2.064072	1.049761	-0.592319
41	1	0	3.170468	-0.085072	3.138481
42	8	0	5.690955	-0.331494	2.257146
43	1	0	5.486502	-0.291271	3.206264
44	8	0	1.017238	-0.019203	1.665854

Int2

Center Number	Atomic Number	Atomic Type	Coordinates (Angstroms) X	Y	Z
1	6	0	-4.133096	1.018892	-0.002411
2	1	0	-4.648011	0.721037	0.926732
3	6	0	-3.749801	-0.227610	-0.799937
4	6	0	-2.970332	1.898328	0.404402
5	1	0	-4.665381	-0.693889	-1.190875
6	1	0	-3.110283	0.032239	-1.654945
7	8	0	-1.933320	1.454185	0.951744
8	7	0	-3.087623	3.207693	0.196156
9	1	0	-3.945988	3.559152	-0.213856
10	6	0	-2.044136	4.152718	0.558586
11	1	0	-2.308652	5.140241	0.163245
12	34	0	-2.835933	-1.646186	0.235712
13	6	0	1.313807	-0.792414	-2.285838
14	1	0	1.255672	-0.923874	-3.381997
15	6	0	0.661893	-2.000897	-1.643394
16	6	0	0.640123	0.529264	-1.959416
17	1	0	-0.414320	-2.058219	-1.851270
18	1	0	1.152576	-2.924938	-1.970759
19	8	0	-0.515610	0.574029	-1.504993
20	7	0	1.336167	1.651444	-2.194613
21	1	0	0.844978	2.510978	-1.964217
22	6	0	2.697059	1.749630	-2.697654
23	1	0	2.824715	1.181406	-3.630578
24	34	0	0.728714	-2.027093	0.358307
25	29	0	-0.944761	-0.224838	0.568649

26	1	0	2.911869	2.803875	-2.909599
27	1	0	3.431745	1.390838	-1.958842
28	1	0	-1.930125	4.219710	1.651732
29	1	0	-1.082333	3.835126	0.129703
30	1	0	2.385123	-0.750963	-2.048996
31	1	0	-4.847451	1.619383	-0.587322
32	6	0	4.638497	-0.479024	0.589006
33	6	0	3.551239	-1.332038	0.443699
34	6	0	2.241495	-0.875306	0.649822
35	6	0	2.043634	0.459476	1.038989
36	6	0	3.123670	1.328855	1.190215
37	6	0	4.425872	0.861188	0.959567
38	1	0	5.657693	-0.827588	0.418892
39	1	0	3.715874	-2.370090	0.148102
40	1	0	0.673178	1.833953	1.372145
41	1	0	2.951830	2.365913	1.488542
42	8	0	5.507614	1.652760	1.082792
43	1	0	5.248185	2.549671	1.340470
44	8	0	0.761724	0.873713	1.264403

RC’

Center Number	Atomic Number	Atomic Type	Coordinates (Angstroms) X	Y	Z
1	6	0	-2.263834	2.611377	-0.447830
2	1	0	-2.233291	2.551368	-1.550176
3	6	0	-0.943056	2.126642	0.158505
4	6	0	-3.496671	1.858431	0.015560
5	1	0	-0.178162	2.908664	0.092738
6	1	0	-1.052645	1.815418	1.204876
7	8	0	-3.593392	0.611041	-0.002509
8	7	0	-4.525473	2.591757	0.440336
9	1	0	-4.429818	3.600680	0.462852
10	6	0	-5.777833	1.996757	0.877530
11	1	0	-6.483864	2.799295	1.120060
12	34	0	-0.206354	0.573084	-0.851234
13	6	0	-0.106380	-3.038383	1.284162
14	1	0	-0.636073	-3.647289	2.041595
15	6	0	-0.107249	-1.589195	1.746480
16	6	0	-0.808253	-3.323714	-0.030039
17	1	0	-1.103096	-1.126812	1.697812
18	1	0	0.226771	-1.536561	2.792103
19	8	0	-1.769752	-2.642673	-0.446832
20	7	0	-0.415312	-4.396124	-0.723592
21	1	0	-0.949866	-4.569930	-1.571156
22	6	0	0.636575	-5.339764	-0.373931
23	1	0	0.450829	-5.811981	0.602379
24	34	0	1.174198	-0.408021	0.790828
25	29	0	-2.259193	-0.761428	-0.396606
26	1	0	0.656556	-6.126822	-1.136832
27	1	0	1.624996	-4.855697	-0.354041
28	1	0	-6.203894	1.365335	0.083697
29	1	0	-5.622876	1.369417	1.768760
30	1	0	0.921954	-3.421946	1.258521
31	1	0	-2.378858	3.678885	-0.209682

32	6	0	3.407655	2.596175	0.906827
33	6	0	4.344642	1.743610	1.514404
34	6	0	4.856130	0.659367	0.828190
35	6	0	4.437150	0.382080	-0.541749
36	6	0	3.463893	1.280423	-1.135783
37	6	0	2.971349	2.356712	-0.426946
38	1	0	2.993244	3.451904	1.442353
39	1	0	4.660287	1.947091	2.539751
40	1	0	5.582527	-0.021037	1.276201
41	1	0	3.138174	1.069939	-2.156369
42	8	0	2.057369	3.224702	-0.917197
43	1	0	1.780377	2.950024	-1.804742
44	8	0	4.899207	-0.594722	-1.172815

TS1'

Center Number	Atomic Number	Atomic Type	Coordinates (Angstroms) X	Y	Z
1	6	0	3.919454	-1.341225	-0.921191
2	1	0	4.366752	-0.635108	-1.642269
3	6	0	2.433338	-1.540995	-1.222869
4	6	0	4.224044	-0.813708	0.464861
5	1	0	2.322416	-2.158373	-2.123135
6	1	0	1.904352	-2.035352	-0.396902
7	8	0	3.625244	0.171356	0.971484
8	7	0	5.188722	-1.417779	1.145166
9	1	0	5.668388	-2.193641	0.700002
10	6	0	5.640277	-1.001051	2.463684
11	1	0	6.508368	-0.327323	2.387563
12	34	0	1.502531	0.158925	-1.632355
13	6	0	-1.284785	1.053665	1.317059
14	1	0	-1.077473	1.096997	2.401542
15	6	0	-0.803702	-0.301135	0.799659
16	6	0	-0.615051	2.263910	0.714313
17	1	0	0.241813	-0.507592	1.061647
18	1	0	-1.407079	-1.099662	1.252258
19	8	0	0.640696	2.403818	0.713557
20	7	0	-1.362249	3.238661	0.213116
21	1	0	-0.830800	4.029124	-0.146511
22	6	0	-2.818482	3.316651	0.155614
23	1	0	-3.256089	3.325756	1.165109
24	34	0	-0.959219	-0.554115	-1.157934
25	29	0	2.069284	1.155393	0.509050
26	1	0	-3.081862	4.258464	-0.339758
27	1	0	-3.251304	2.482383	-0.420007
28	1	0	4.825040	-0.474761	2.973572
29	1	0	5.926034	-1.888349	3.043051
30	1	0	-2.370472	1.115942	1.202481
31	1	0	4.437608	-2.300944	-1.063990
32	6	0	-3.285769	-1.417118	-1.096271
33	6	0	-3.152882	-2.576432	-0.281655
34	6	0	-3.544253	-2.568684	1.036709
35	6	0	-4.174275	-1.397375	1.580543
36	6	0	-4.395430	-0.278922	0.804727
37	6	0	-4.011827	-0.234844	-0.588278

38	1	0	-3.236219	-1.514467	-2.183088
39	1	0	-2.702786	-3.475925	-0.707956
40	1	0	-3.409413	-3.448997	1.669743
41	1	0	-4.904083	0.586515	1.233173
42	8	0	-4.248131	0.748446	-1.323003
43	1	0	-4.357665	-2.218709	3.299724
44	8	0	-4.563635	-1.374683	2.871925

Int1’

Center Number	Atomic Number	Atomic Type	Coordinates (Angstroms) X	Y	Z
1	6	0	-4.346858	0.761908	-1.035535
2	1	0	-4.932797	-0.173069	-1.021655
3	6	0	-3.070027	0.563374	-1.849688
4	6	0	-4.111987	1.157915	0.404276
5	1	0	-3.336064	0.496196	-2.914576
6	1	0	-2.380683	1.411648	-1.732926
7	8	0	-3.276793	0.571876	1.145649
8	7	0	-4.838362	2.152584	0.893396
9	1	0	-5.512703	2.590316	0.273536
10	6	0	-4.771706	2.623296	2.268170
11	1	0	-5.601436	2.210999	2.863454
12	34	0	-2.092308	-1.109174	-1.475232
13	6	0	1.277559	-0.433589	1.546180
14	1	0	0.963473	-0.032751	2.527558
15	6	0	0.850030	0.558746	0.465933
16	6	0	0.664627	-1.809732	1.450012
17	1	0	-0.227792	0.772709	0.493535
18	1	0	1.354185	1.520811	0.629607
19	8	0	-0.580123	-1.984861	1.314119
20	7	0	1.438345	-2.879355	1.568627
21	1	0	0.937089	-3.764922	1.534894
22	6	0	2.882795	-2.924142	1.778928
23	1	0	3.162293	-2.431767	2.722020
24	34	0	1.207875	0.011991	-1.395277
25	29	0	-1.984208	-0.778275	0.879268
26	1	0	3.176871	-3.978131	1.843504
27	1	0	3.425116	-2.457622	0.943198
28	1	0	-3.821004	2.304308	2.709980
29	1	0	-4.834252	3.719504	2.279656
30	1	0	2.368956	-0.500017	1.574130
31	1	0	-4.974332	1.526595	-1.517875
32	6	0	3.128229	0.683056	-1.538648
33	6	0	3.089770	2.153375	-1.394596
34	6	0	3.591672	2.766386	-0.301852
35	6	0	4.275736	1.988646	0.723011
36	6	0	4.468676	0.634903	0.598538
37	6	0	4.001944	-0.087517	-0.560620
38	1	0	3.352663	0.361983	-2.565843
39	1	0	2.596367	2.731256	-2.178940
40	1	0	3.521157	3.849929	-0.178214
41	1	0	5.025762	0.091483	1.363208
42	8	0	4.238430	-1.282725	-0.754705
43	1	0	4.552631	3.561417	1.774836

44	8	0	4.745314	2.612084	1.809515

Int2'

Center Number	Atomic Number	Atomic Type	Coordinates (Angstroms) X	Y	Z
1	6	0	-4.898747	1.168856	-0.288253
2	1	0	-5.080623	0.737814	-1.287737
3	6	0	-3.650289	2.047857	-0.318789
4	6	0	-4.812034	0.014816	0.683490
5	1	0	-3.879627	2.968996	-0.873995
6	1	0	-3.341913	2.338274	0.695283
7	8	0	-3.806393	-0.743153	0.745536
8	7	0	-5.851643	-0.197983	1.477725
9	1	0	-6.632601	0.446981	1.406928
10	6	0	-5.958543	-1.287338	2.435318
11	1	0	-6.844716	-1.899177	2.211696
12	34	0	-2.102293	1.272869	-1.267729
13	6	0	1.010294	-1.489170	0.924203
14	1	0	0.600094	-2.137611	1.721215
15	6	0	0.617964	-0.048407	1.210350
16	6	0	0.498617	-2.055531	-0.380190
17	1	0	-0.467732	0.119447	1.189065
18	1	0	0.976174	0.240435	2.206342
19	8	0	-0.635317	-1.766837	-0.849879
20	7	0	1.246900	-2.929521	-1.042252
21	1	0	0.818313	-3.290317	-1.892124
22	6	0	2.563508	-3.440187	-0.677145
23	1	0	2.575959	-3.807291	0.358073
24	34	0	1.351331	1.271547	-0.074569
25	29	0	-2.186974	-0.860399	-0.219400
26	1	0	2.792787	-4.281470	-1.341503
27	1	0	3.333626	-2.668274	-0.811610
28	1	0	-5.059083	-1.908407	2.367436
29	1	0	-6.049921	-0.887201	3.455914
30	1	0	2.101409	-1.591811	0.981730
31	1	0	-5.777058	1.783256	-0.038556
32	6	0	3.212163	0.939744	0.299781
33	6	0	3.852932	1.473509	1.425095
34	6	0	5.194303	1.206529	1.688437
35	6	0	5.921659	0.393837	0.800081
36	6	0	5.302065	-0.141340	-0.334892
37	6	0	3.953873	0.129890	-0.589389
38	1	0	2.464372	-0.116427	-1.733450
39	1	0	3.285949	2.107353	2.110601
40	1	0	5.681238	1.626291	2.571909
41	1	0	5.869839	-0.769106	-1.022774
42	8	0	3.393311	-0.422913	-1.688440
43	1	0	7.545260	0.522810	1.803366
44	8	0	7.227129	0.098030	0.993534

13. ICP-MS

We thank Ofir Tirosh for the copper ICP-MS analysis of purified peptide conjugates **3a$_1$**, **5f**, **16a** and **20e**. The analysis was run on an Aglient 8900 triple quadrupole ICP-MS. This evaluation helped quantify the copper concentration (ppb) in each of the purified Sec/Cys arylation products. For each of the purified peptide conjugates was lyophilized, re-dissolved in 68% nitric acid at 90 ^{o}C for 4 h, and analyzed by ICP-MS. The results obtained are summarized in Table S2.

Table S6. Cu concentration remained on purified peptide conjugates **3a$_1$**, **5f**, **16a** and **20e**.

Sample	Weights	Cu concentration
**3a$_1$**	2 mg	1.34 ppb
**5f**	1 mg	2.13 ppb
**16a**	1 mg	1.97 ppb
**20e**	1 mg	3.16 ppb

14. NMR Spectra

^{1}H NMR Spectrum of **3a$_1$**.

^{1}H NMR (500 MHz, DMSO-d_6) δ 9.63 (s, 1H), 9.39 (s, 1H), 8.73 (t, J = 5.6 Hz, 1H), 8.32 (d, J = 8.1 Hz, 1H), 8.24 (d, J = 7.1 Hz, 1H), 8.12 (d, J = 5.1 Hz, 2H), 8.02 (t, J = 5.8 Hz, 1H), 7.94 (d, J = 7.6 Hz, 1H), 7.18 (d, J = 2.2 Hz, 1H), 7.16 (d, J = 8.4 Hz, 1H), 7.10 – 7.03 (m, 1H), 6.35 (d, J = 2.5 Hz, 1H), 6.19 (dd, J = 8.3, 2.5 Hz, 1H), 4.46 (td, J = 8.4, 5.7 Hz, 1H), 4.32 – 4.17 (m, 2H), 3.83 (ddq, J = 16.8, 10.8, 5.6 Hz, 3H), 3.61 (qd, J = 16.8, 5.8 Hz, 2H), 3.02 (dd, J = 12.0, 5.7 Hz, 1H), 1.69 (dt, J = 13.4, 6.7 Hz, 1H), 1.64 – 1.52 (m, 3H), 1.48 (t, J = 7.3 Hz, 2H), 1.21 (d, J = 7.1 Hz, 3H), 0.90 (dd, J = 8.2, 6.4 Hz, 6H), 0.83 (dd, J = 17.4, 6.6 Hz, 6H).

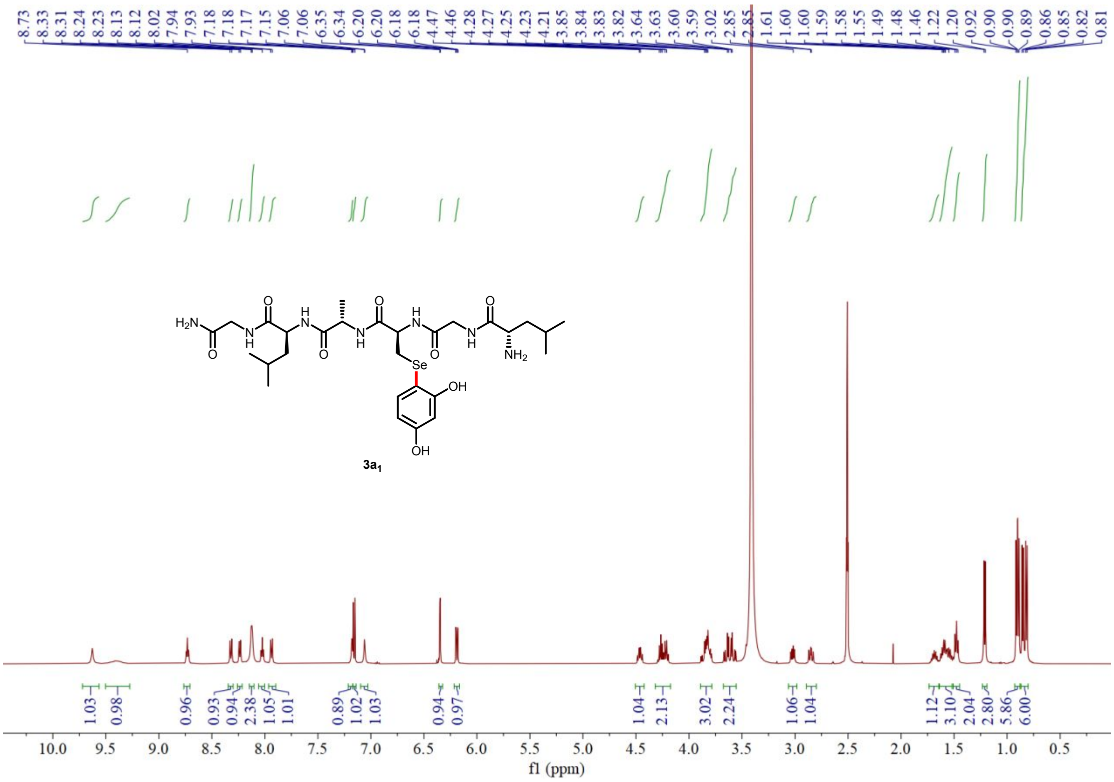

Figure S111. ^{1}H NMR Spectrum of **3a$_1$**.

COSY NMR Spectrum of **3a**$_1$.

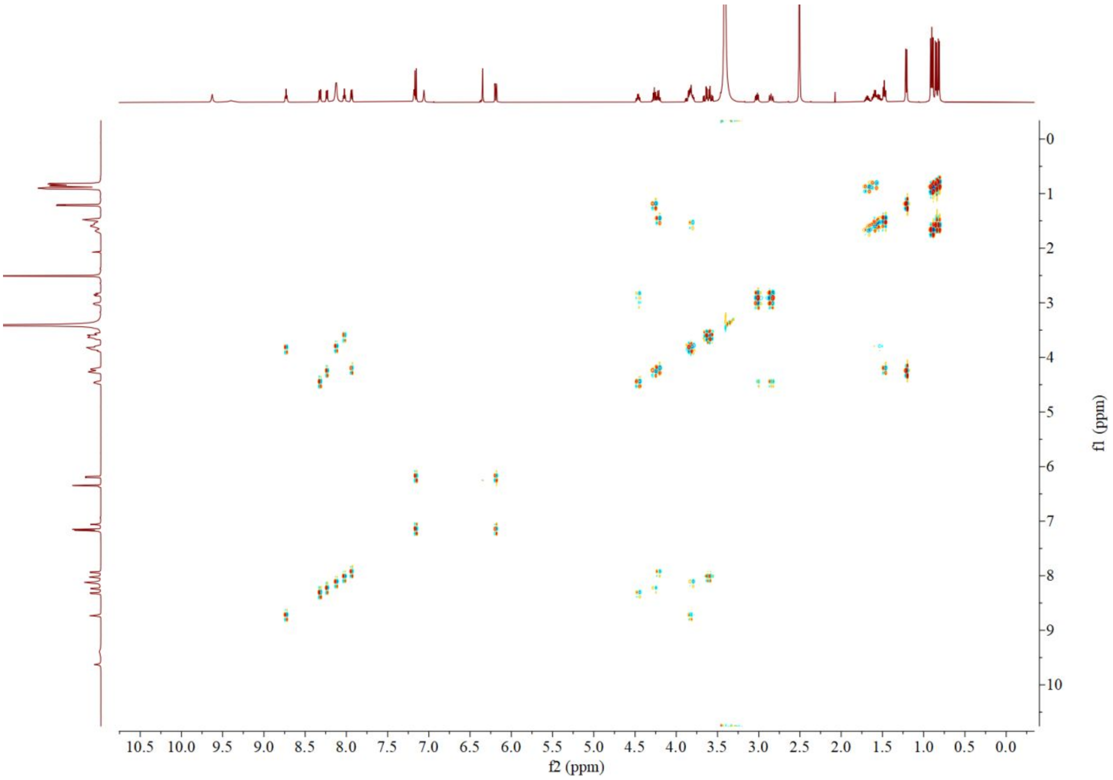

Figure S112. COSY NMR Spectrum of **3a**$_1$.

HSQC NMR Spectrum of **3a**$_1$.

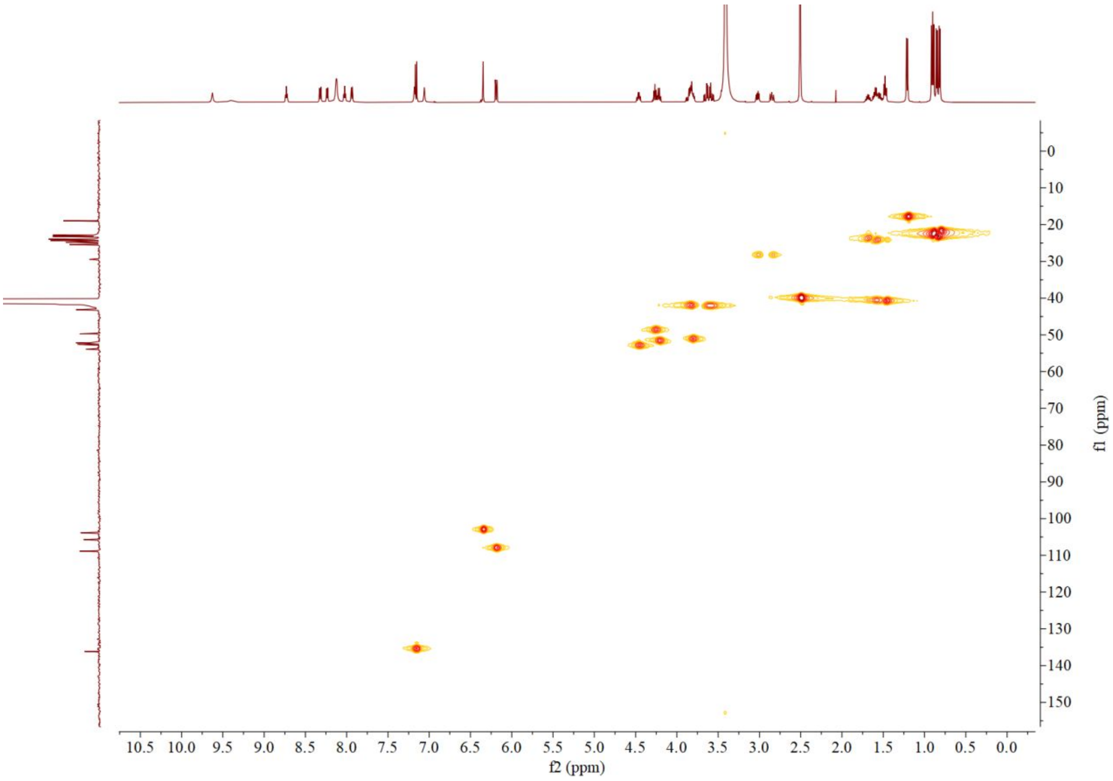

Figure S113. HSQC NMR Spectrum of **3a**$_1$.

HMBC NMR Spectrum of **3a₁**.

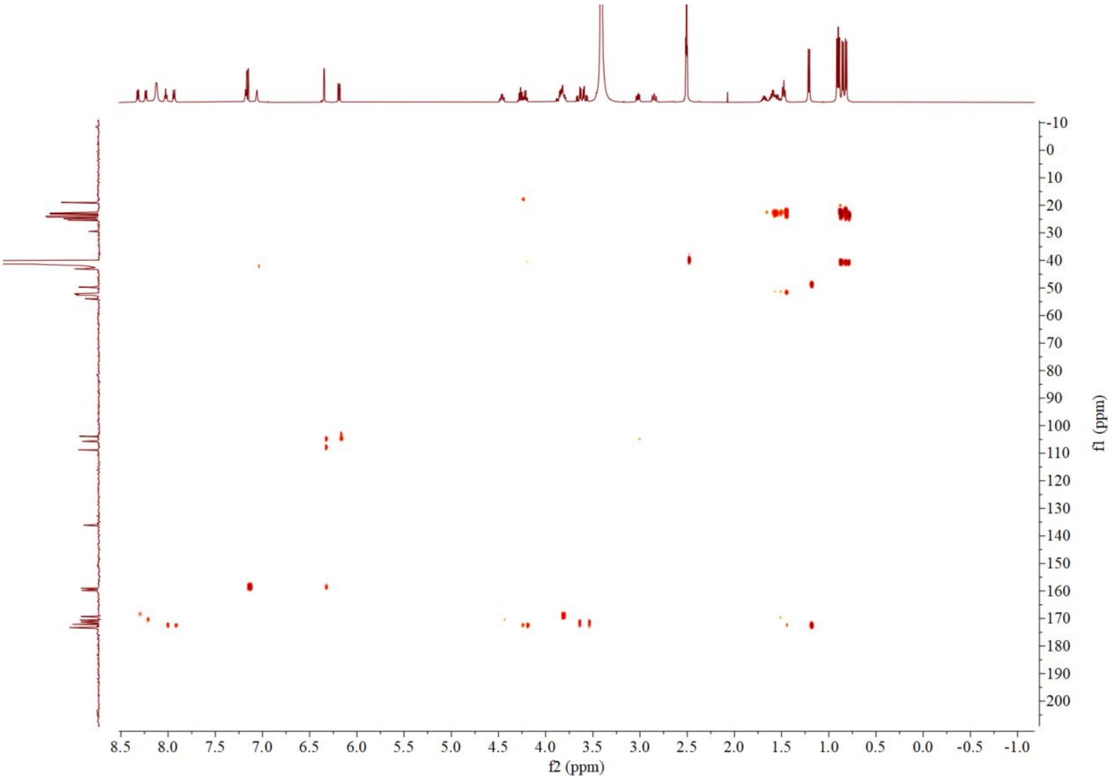

Figure S114. HMBC NMR Spectrum of **$3a_1$**.

^{1}H NMR Spectrum of **3a$_2$**.

^{1}H NMR (500 MHz, DMSO-d_6) δ 9.97 (s, 2H), 8.78 (t, J = 5.7 Hz, 2H), 8.40 (d, J = 8.1 Hz, 2H), 8.25 (d, J = 7.1 Hz, 2H), 8.19 – 8.16 (m, 4H), 8.09 – 8.01 (m, 4H), 7.44 (s, 1H), 7.28 – 7.24 (m, 2H), 7.14 – 7.09 (m, 2H), 6.56 (s, 1H), 4.55 (td, J = 8.3, 5.8 Hz, 2H), 4.33 (q, J = 7.0 Hz, 2H), 4.26 (q, J = 7.6 Hz, 2H), 3.89 (dt, J = 13.6, 6.2 Hz, 6H), 3.68 (dd, J = 14.1, 5.7 Hz, 4H), 3.08 (dd, J = 11.8, 5.6 Hz, 2H), 2.96 (dd, J = 11.8, 8.7 Hz, 2H), 1.73 (dt, J = 13.3, 6.6 Hz, 2H), 1.68 – 1.58 (m, 6H), 1.53 (t, J = 7.3 Hz, 4H), 1.27 (d, J = 7.0 Hz, 6H), 0.95 (dd, J = 8.1, 6.5 Hz, 12H), 0.88 (dd, J = 18.5, 6.5 Hz, 12H).

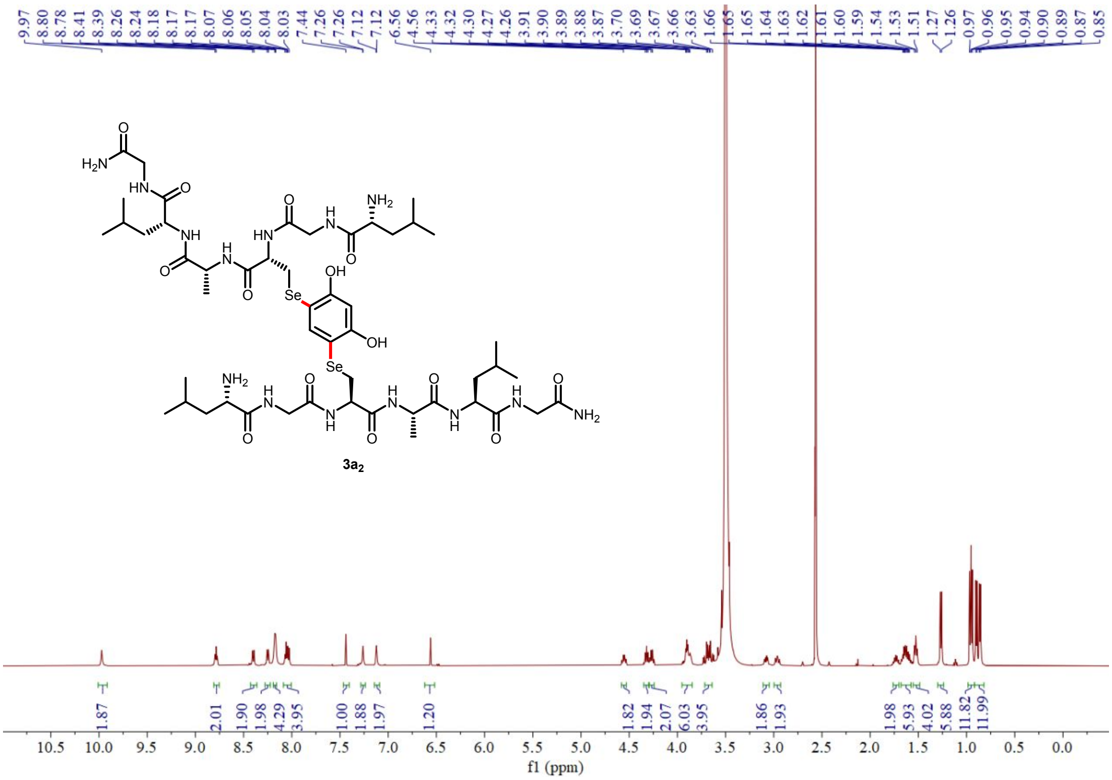

Figure S115. ^{1}H NMR Spectrum of **3a$_2$**.

^{1}H NMR Spectrum of **3b₁**.

^{1}H NMR (500 MHz, DMSO-d_6) δ 9.42 – 9.20 (m, 2H), 8.74 (t, *J* = 5.7 Hz, 1H), 8.32 (d, *J* = 8.1 Hz, 1H), 8.23 (d, *J* = 7.1 Hz, 1H), 8.13 (d, *J* = 5.2 Hz, 2H), 8.03 (t, *J* = 5.8 Hz, 1H), 7.93 (d, *J* = 7.7 Hz, 1H), 7.18 (d, *J* = 2.1 Hz, 1H), 7.06 (d, *J* = 0.9 Hz, 2H), 6.40 (s, 1H), 4.46 (td, *J* = 8.3, 5.9 Hz, 1H), 4.24 (dq, *J* = 18.6, 7.4 Hz, 2H), 3.84 (qd, *J* = 16.8, 5.6 Hz, 3H), 3.61 (qd, *J* = 16.8, 5.8 Hz, 3H), 3.01 (dd, *J* = 12.0, 5.9 Hz, 1H), 2.85 (dd, *J* = 12.0, 8.6 Hz, 1H), 1.98 (s, 3H), 1.74 – 1.64 (m, 1H), 1.64 – 1.52 (m, 3H), 1.48 (t, *J* = 7.3 Hz, 2H), 1.21 (d, *J* = 7.1 Hz, 3H), 0.90 (dd, *J* = 8.3, 6.5 Hz, 6H), 0.83 (dd, *J* = 17.0, 6.6 Hz, 6H).

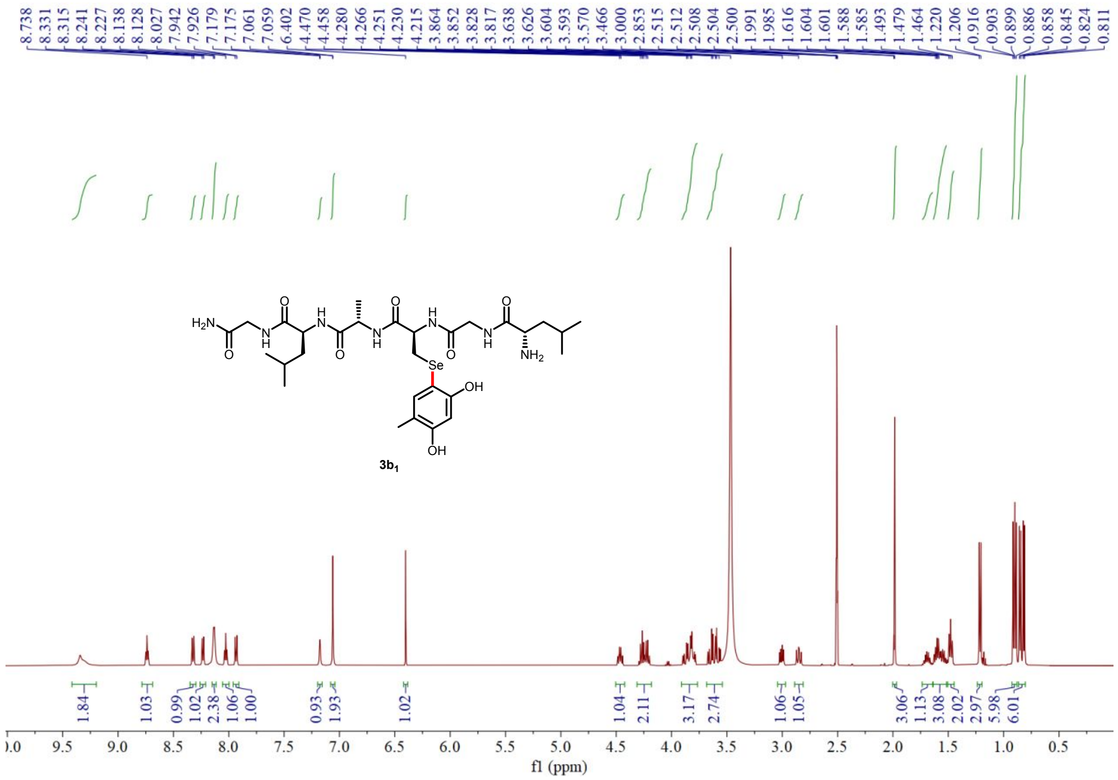

Figure S116. ^{1}H NMR Spectrum of **3b₁**.

COSY NMR Spectrum of **3b$_1$**.

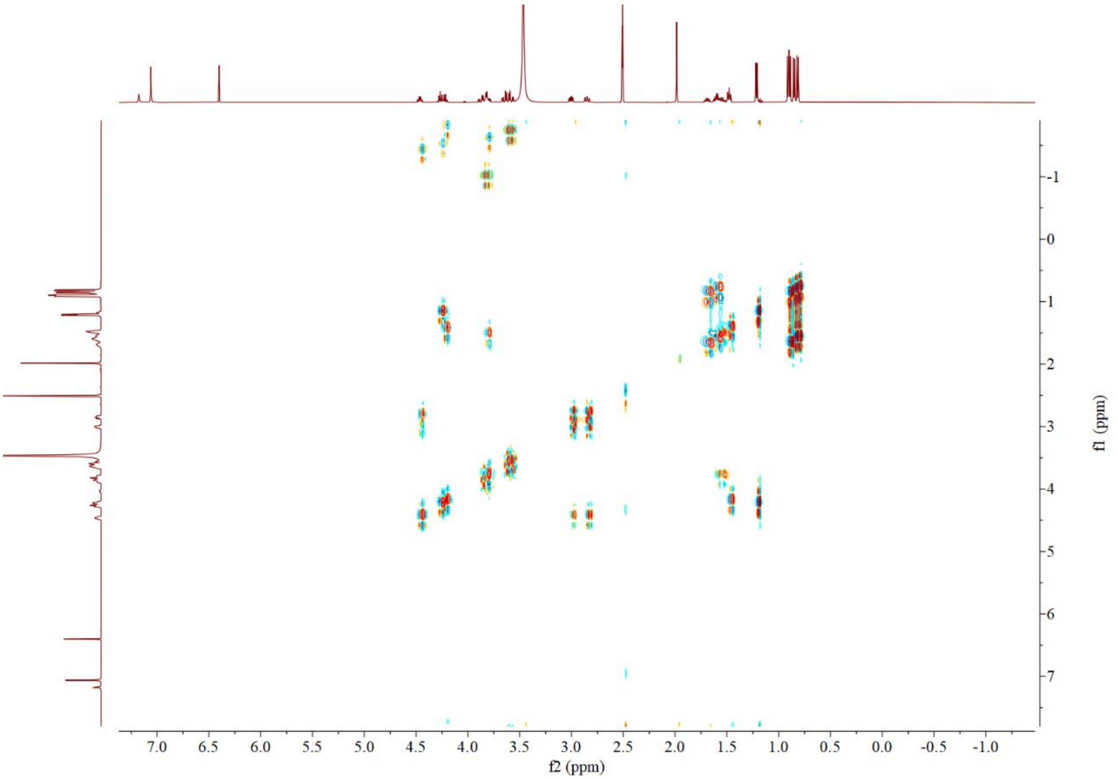

Figure S117. COSY NMR Spectrum of **3b$_1$**.

HSQC NMR Spectrum of **3b$_1$**.

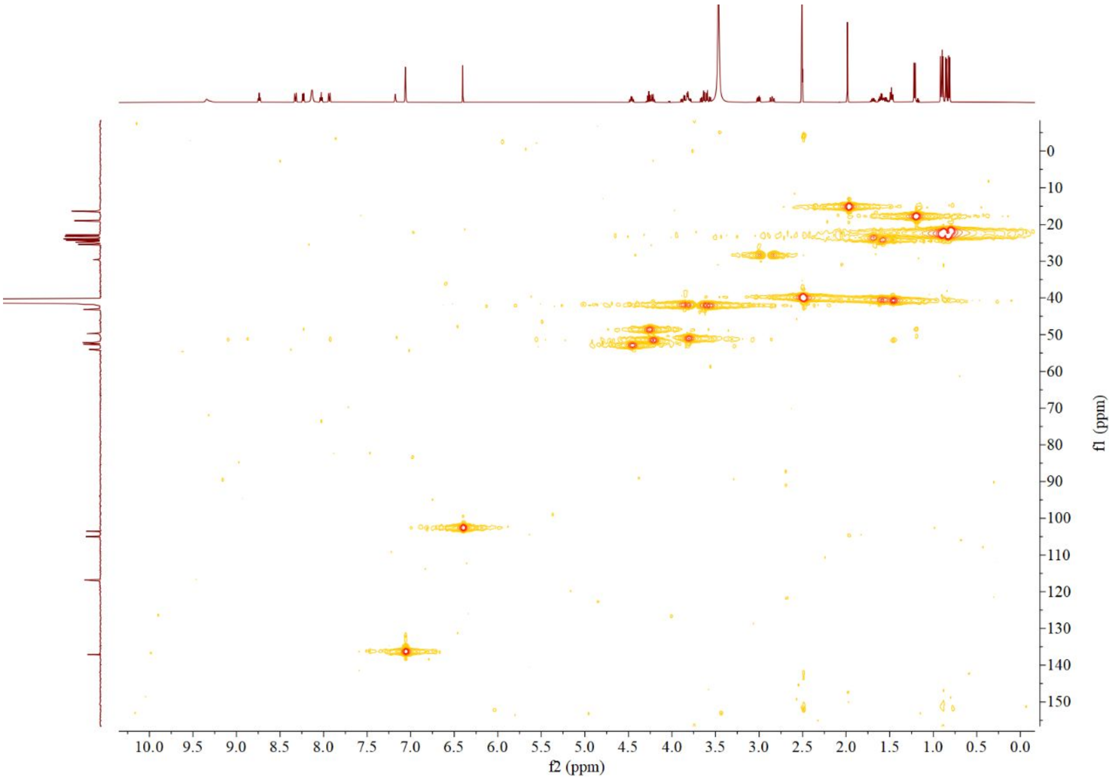

Figure S118. HSQC NMR Spectrum of **3b$_1$**.

^{1}H NMR Spectrum of **3c$_1$**.

^{1}H NMR (500 MHz, DMSO-*d6*) δ 9.33 (s, 1H), 9.25 (s, 1H), 8.68 (t, *J* = 5.6 Hz, 1H), 8.27 (d, *J* = 8.1 Hz, 1H), 8.12 (d, *J* = 7.1 Hz, 1H), 8.07 (d, *J* = 5.0 Hz, 2H), 7.99 (t, *J* = 5.8 Hz, 1H), 7.94 (d, *J* = 7.7 Hz, 1H), 7.16 (d, *J* = 2.3 Hz, 1H), 7.03 (s, 1H), 6.22 – 6.15 (m, 2H), 4.40 – 4.32 (m, 1H), 4.28 – 4.14 (m, 3H), 3.81 (qd, *J* = 16.7, 5.6 Hz, 3H), 3.59 (qd, *J* = 16.8, 5.8 Hz, 3H), 2.91 (dd, *J* = 11.9, 5.6 Hz, 1H), 2.75 (dd, *J* = 11.9, 9.0 Hz, 1H), 2.28 (s, 3H), 1.70 – 1.62 (m, 1H), 1.60 – 1.48 (m, 3H), 1.47 – 1.39 (m, 2H), 1.18 (d, *J* = 7.1 Hz, 3H), 0.88 (dd, *J* = 8.1, 6.4 Hz, 6H), 0.80 (dd, *J* = 17.8, 6.5 Hz, 6H).

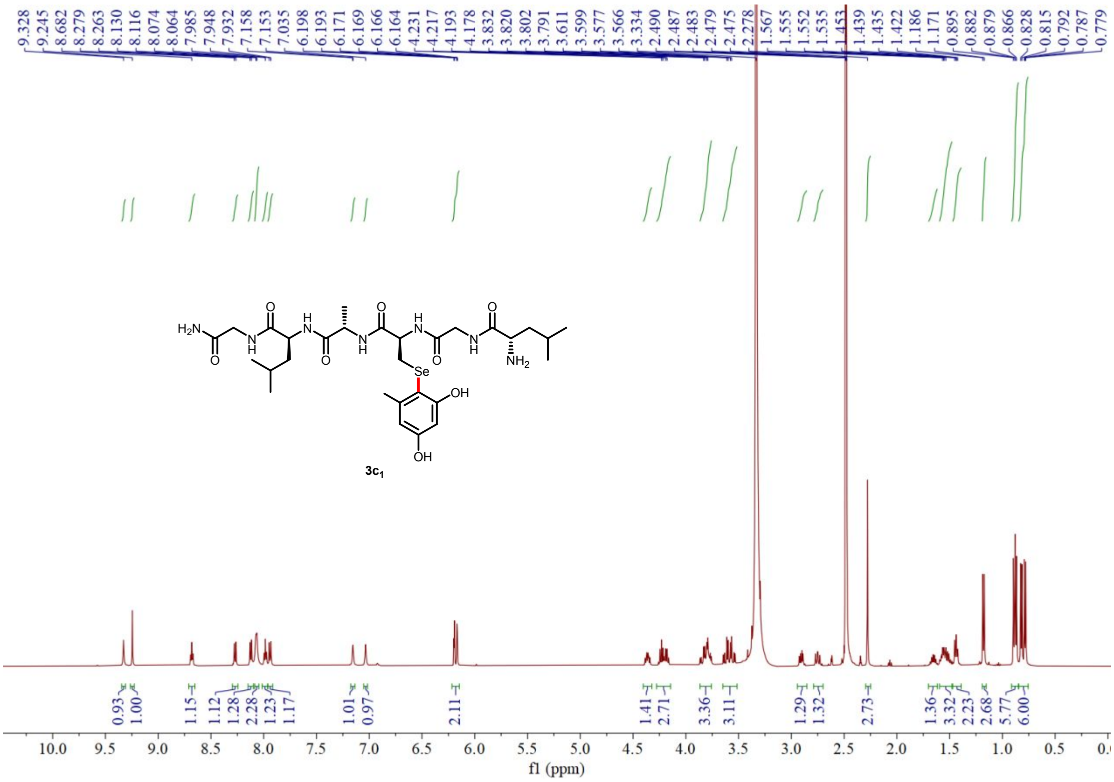

Figure S119. ^{1}H NMR Spectrum of **3c$_1$**.

NOESY Spectrum of **3c$_1$**.

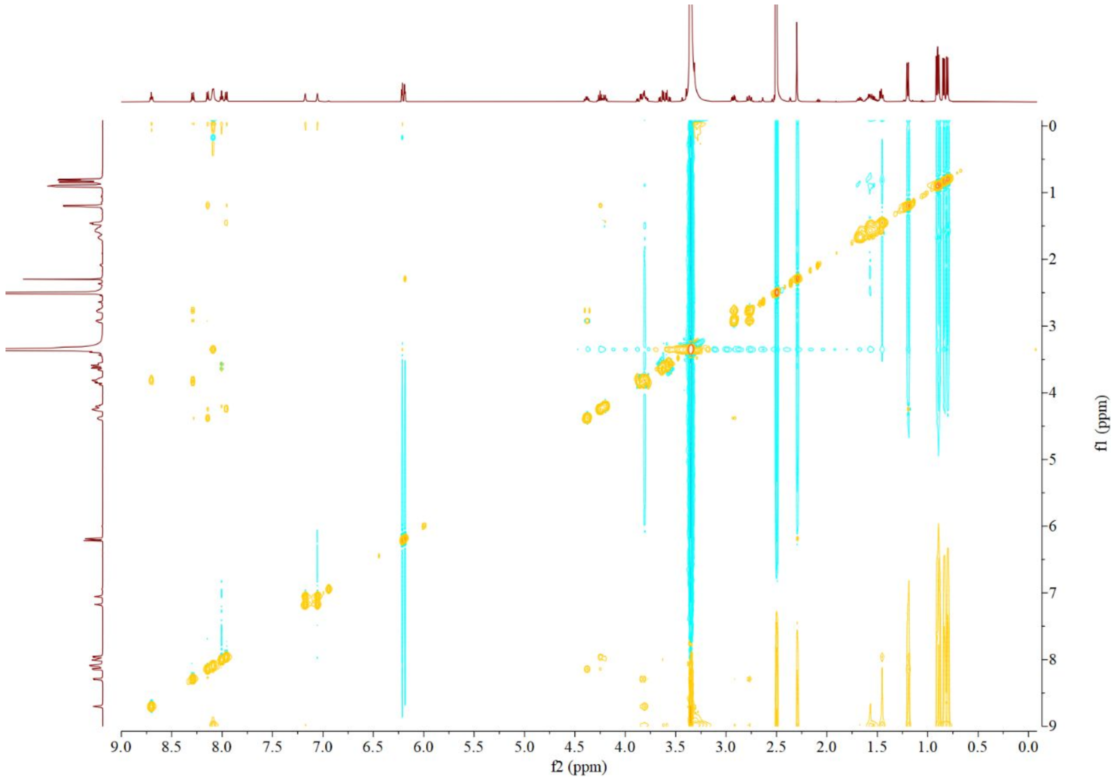

Figure S120. NOESY Spectrum of **3c$_1$**.

^{1}H-^{1}H ROESY NMR Spectrum of **3c$_1$**.

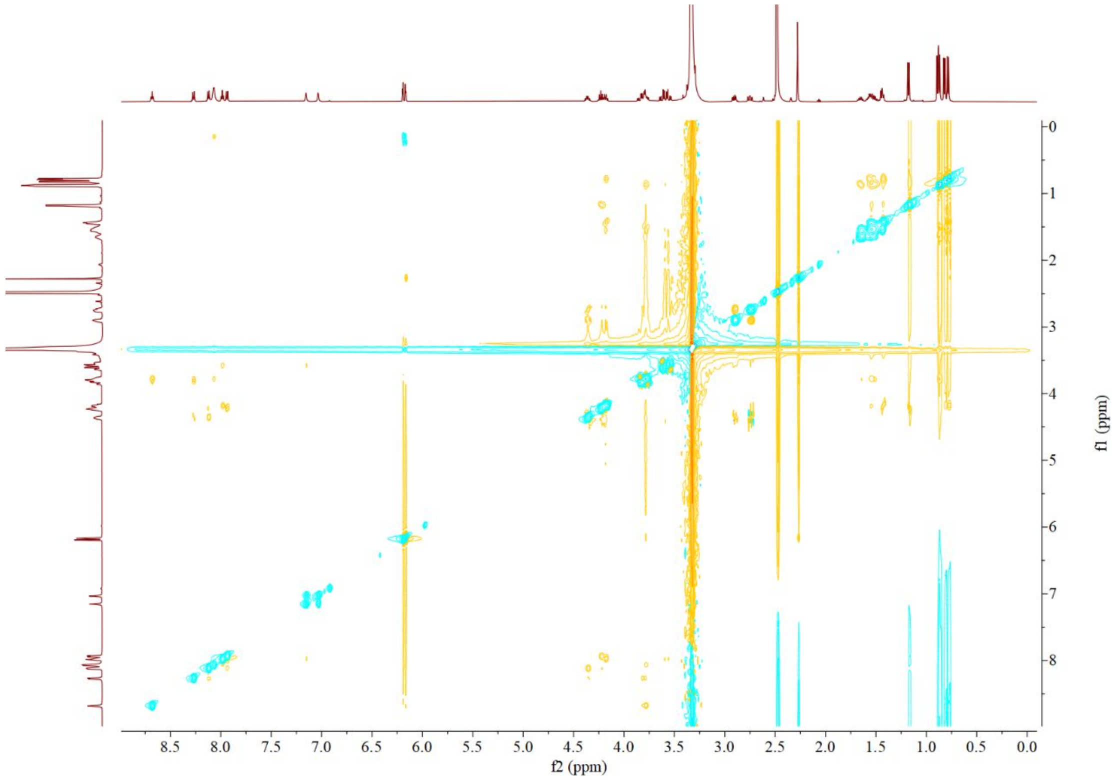

Figure S121. ^{1}H-^{1}H ROESY NMR Spectrum of **3c$_1$**.

^{1}H NMR Spectrum of **3d$_1$**.

^{1}H NMR (500 MHz, DMSO-d_6) δ 9.41 (s, 2H), 8.72 (t, J = 5.7 Hz, 1H), 8.29 (d, J = 8.0 Hz, 1H), 8.19 (d, J = 6.9 Hz, 1H), 8.11 (d, J = 5.2 Hz, 2H), 8.02 – 7.96 (m, 2H), 7.18 (d, J = 2.2 Hz, 1H), 7.07 (d, J = 2.2 Hz, 1H), 5.98 (d, J = 36.7 Hz, 2H), 4.40 (q, J = 7.5 Hz, 1H), 4.28 – 4.16 (m, 2H), 3.83 (ddq, J = 16.7, 11.3, 5.6 Hz, 3H), 3.66 (s, 1H), 3.65 (s, 3H), 3.60 (td, J = 16.9, 5.8 Hz, 2H), 2.86 (d, J = 7.3 Hz, 2H), 1.68 (dt, J = 13.4, 6.7 Hz, 1H), 1.57 (dddd, J = 24.2, 13.8, 7.2, 1.9 Hz, 3H), 1.48 (dd, J = 8.2, 6.5 Hz, 2H), 1.22 (d, J = 7.1 Hz, 3H), 0.90 (dd, J = 7.9, 6.5 Hz, 6H), 0.83 (dd, J = 15.6, 6.5 Hz, 6H).

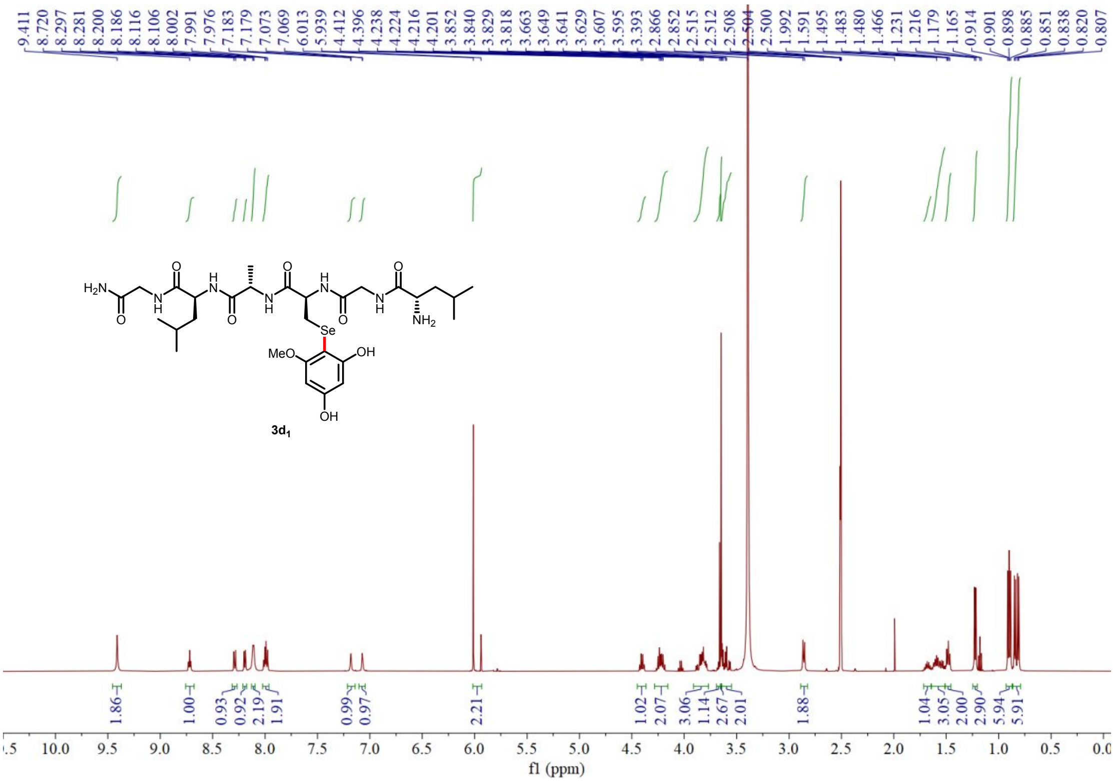

Figure S122. ^{1}H NMR Spectrum of **3d$_1$**.

^{1}H NMR Spectrum of **3g**.

^{1}H NMR (500 MHz, DMSO-d_6) δ 8.77 (t, J = 5.7 Hz, 1H), 8.70 (s, 2H), 8.23 (d, J = 7.6 Hz, 1H), 8.16 (d, J = 5.2 Hz, 2H), 8.07 (t, J = 5.8 Hz, 1H), 7.96 (dd, J = 8.3, 1.7 Hz, 1H), 7.63 (t, J = 7.8 Hz, 1H), 7.33 (t, J = 7.6 Hz, 2H), 7.21 (s, 1H), 7.12 (s, 1H), 4.43 (q, J = 7.3 Hz, 1H), 4.33 – 4.24 (m, 2H), 3.95 – 3.83 (m, 4H), 3.67 (qd, J = 16.8, 5.8 Hz, 3H), 3.00 (dd, J = 12.2, 6.8 Hz, 1H), 2.87 (s, 1H), 1.71 (dq, J = 13.2, 6.5 Hz, 2H), 1.66 – 1.51 (m, 4H), 1.31 (d, J = 7.1 Hz, 3H), 0.94 (dd, J = 8.9, 6.4 Hz, 6H), 0.88 (t, J = 6.6 Hz, 6H).

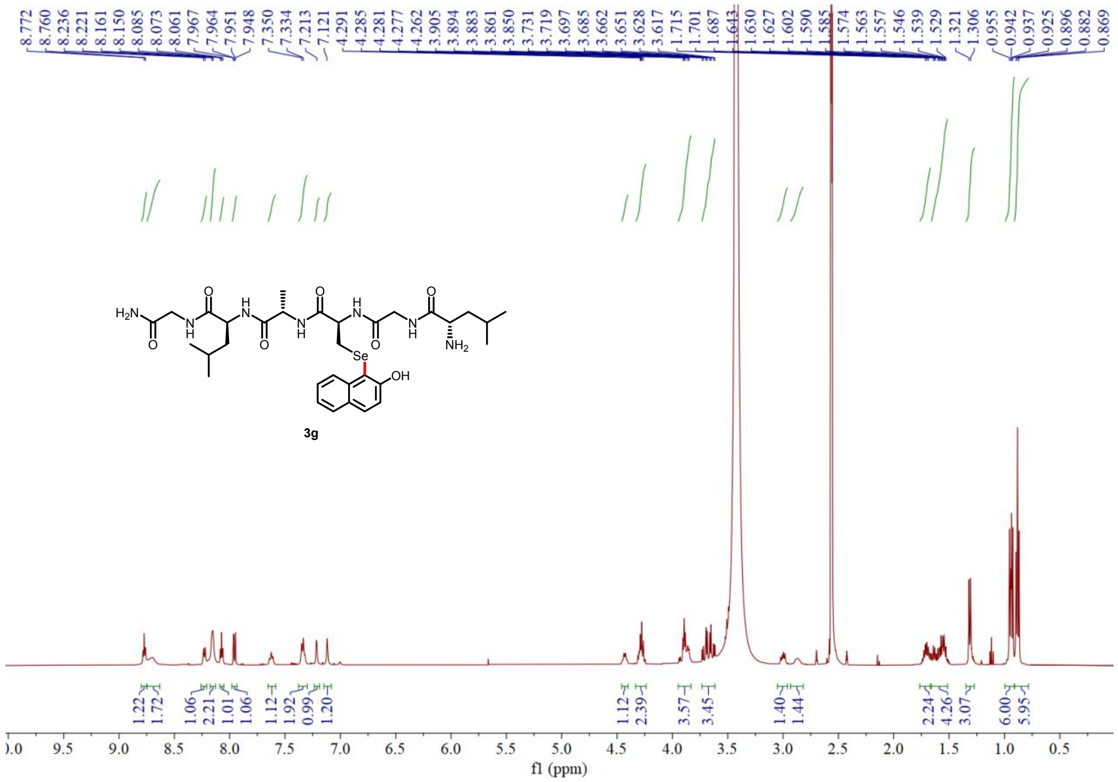

Figure S123. ^{1}H NMR Spectrum of **3g**.

^{1}H NMR Spectrum of **2h**.

^{1}H NMR (500 MHz, DMSO-d_6) δ 8.85 (s, 1H), 6.84 – 6.78 (m, 1H), 6.05 – 6.02 (m, 2H), 5.98 – 5.95 (m, 1H), 4.89 (s, 2H).

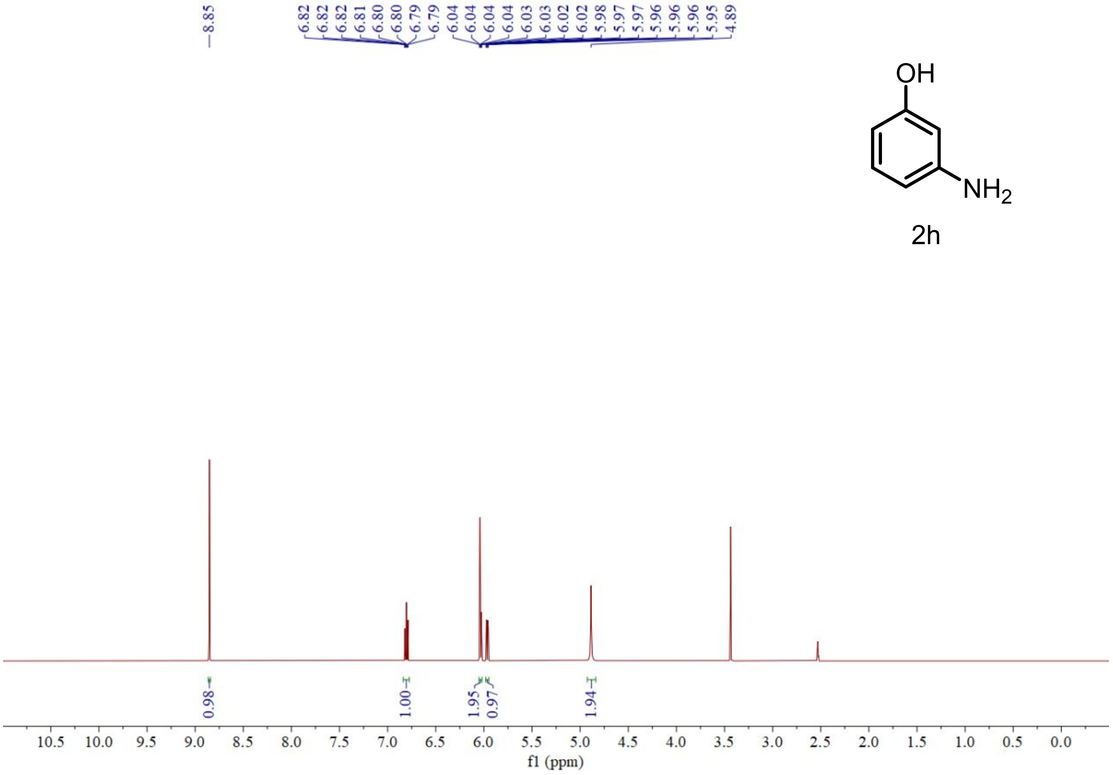

Figure S124. ^{1}H NMR Spectrum of **2h**.

^{1}H NMR Spectrum of **3h**.

^{1}H NMR (500 MHz, DMSO-d_6) δ 9.99 (s, 1H), 8.79 (t, J = 5.6 Hz, 1H), 8.42 (d, J = 8.0 Hz, 1H), 8.33 (d, J = 7.2 Hz, 1H), 8.21 – 8.16 (m, 3H), 8.08 (t, J = 5.6 Hz, 1H), 8.00 (d, J = 7.6 Hz, 1H), 7.30 – 7.21 (m, 2H), 7.12 (s, 1H), 6.52 (d, J = 2.4 Hz, 1H), 6.42 (dd, J = 8.4, 2.0 Hz, 1H), 4.55 (td, J = 8.4, 5.6 Hz, 1H), 4.33 (p, J = 6.8 Hz, 1H), 4.27 (t, J = 7.6 Hz, 1H), 3.97 – 3.83 (m, 3H), 3.74-3.65 (m, 3H), 3.12 (dd, J = 12.0, 5.8 Hz, 1H), 2.94 (dd, J = 12.0, 8.8 Hz, 1H), 1.74 (dp, J = 13.6, 6.8 Hz, 1H), 1.69 – 1.57 (m, 3H), 1.54 (q, J = 7.2, 6.4 Hz, 2H), 1.27 (d, J = 7.2 Hz, 3H), 0.98 – 0.94 (m, 6H), 0.89 (dd, J = 17.6, 6.4 Hz, 6H).

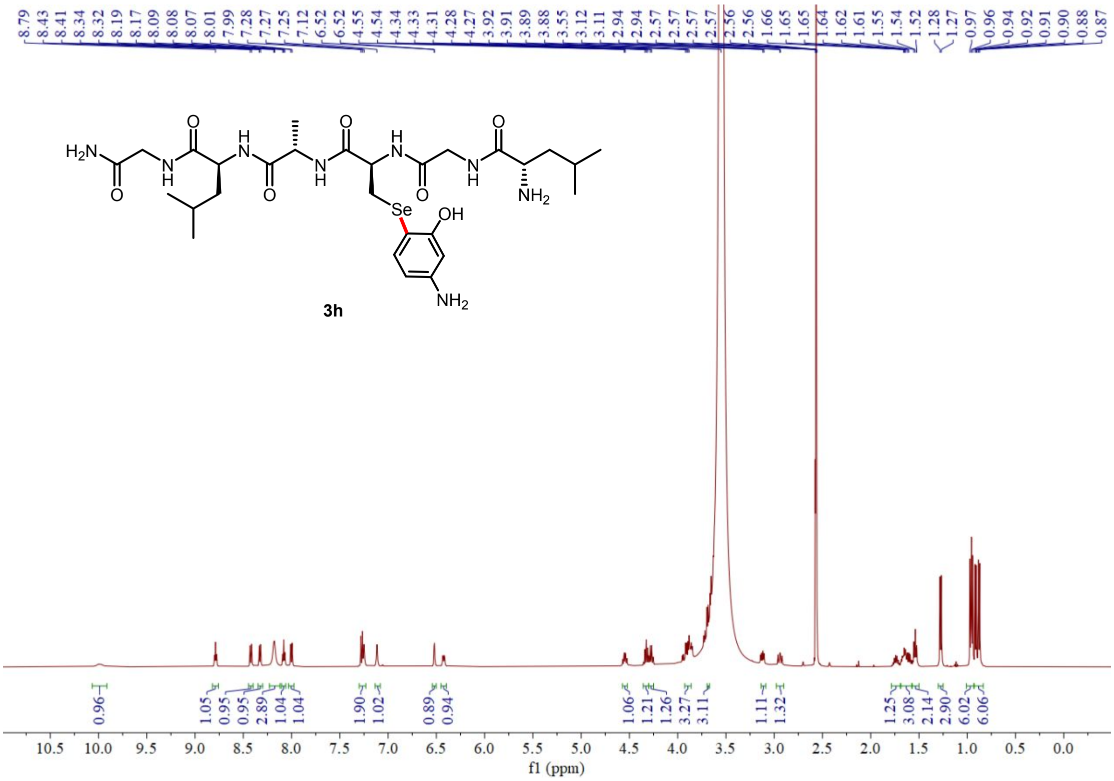

Figure S125. ^{1}H NMR Spectrum of **3h**.

^{1}H NMR Spectrum of **3k**.

^{1}H NMR (500 MHz, DMSO-d_6) δ 9.36 (s, 1H), 8.67 (t, J = 5.7 Hz, 1H), 8.27 (d, J = 8.1 Hz, 1H), 8.11 (d, J = 7.1 Hz, 1H), 8.08 – 8.05 (m, 2H), 7.98 (t, J = 5.8 Hz, 1H), 7.94 (d, J = 7.7 Hz, 1H), 7.16 (s, 1H), 7.04 (s, 1H), 6.53 (s, 2H), 4.38 (q, J = 7.7 Hz, 1H), 4.27 – 4.21 (m, 1H), 4.20 – 4.15 (m, 1H), 3.80 (qd, J = 16.6, 5.6 Hz, 3H), 3.66 – 3.52 (m, 3H), 3.01 (dd, J = 12.2, 5.5 Hz, 1H), 2.87 – 2.81 (m, 1H), 2.32 (d, J = 1.6 Hz, 3H), 2.14 (d, J = 1.6 Hz, 3H), 1.66 (hept, J = 6.5 Hz, 1H), 1.55 (tt, J = 15.4, 7.0 Hz, 3H), 1.44 (t, J = 7.4 Hz, 2H), 1.18 (dd, J = 7.0, 1.8 Hz, 3H), 0.88 (t, J = 6.5 Hz, 6H), 0.80 (dd, J = 18.1, 6.5 Hz, 6H).

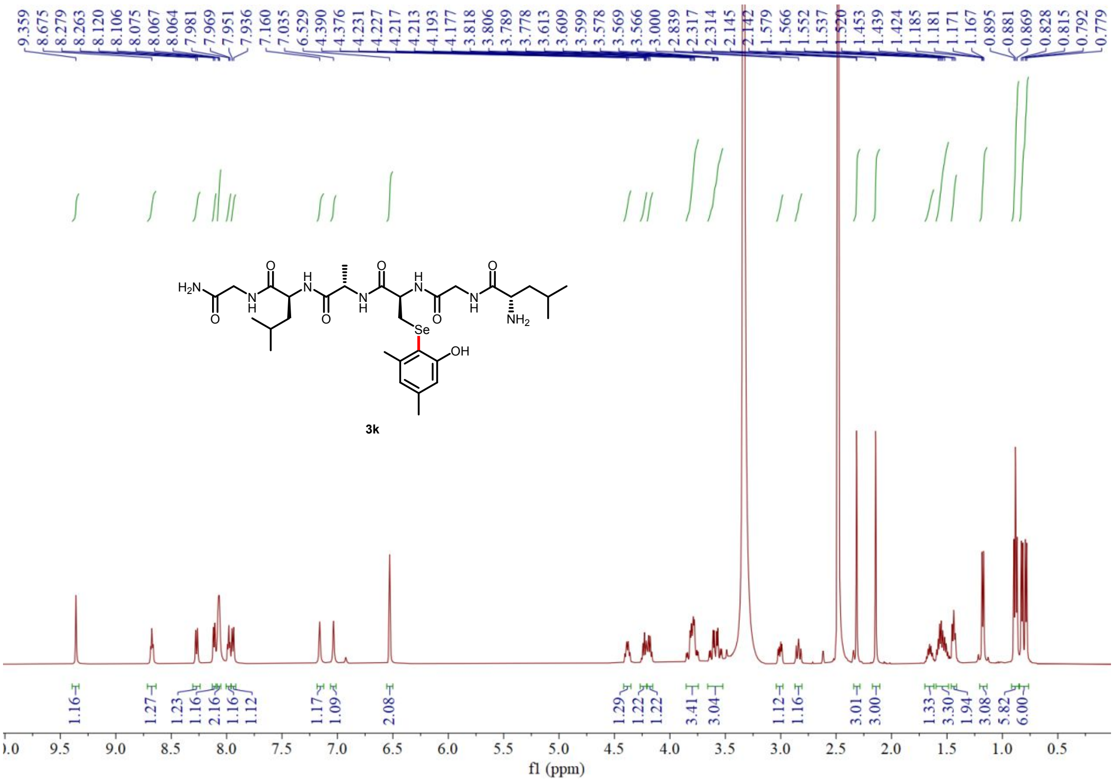

Figure S126. ^{1}H NMR Spectrum of **3k**.

^{1}H-^{1}H ROESY NMR Spectrum of **3k**.

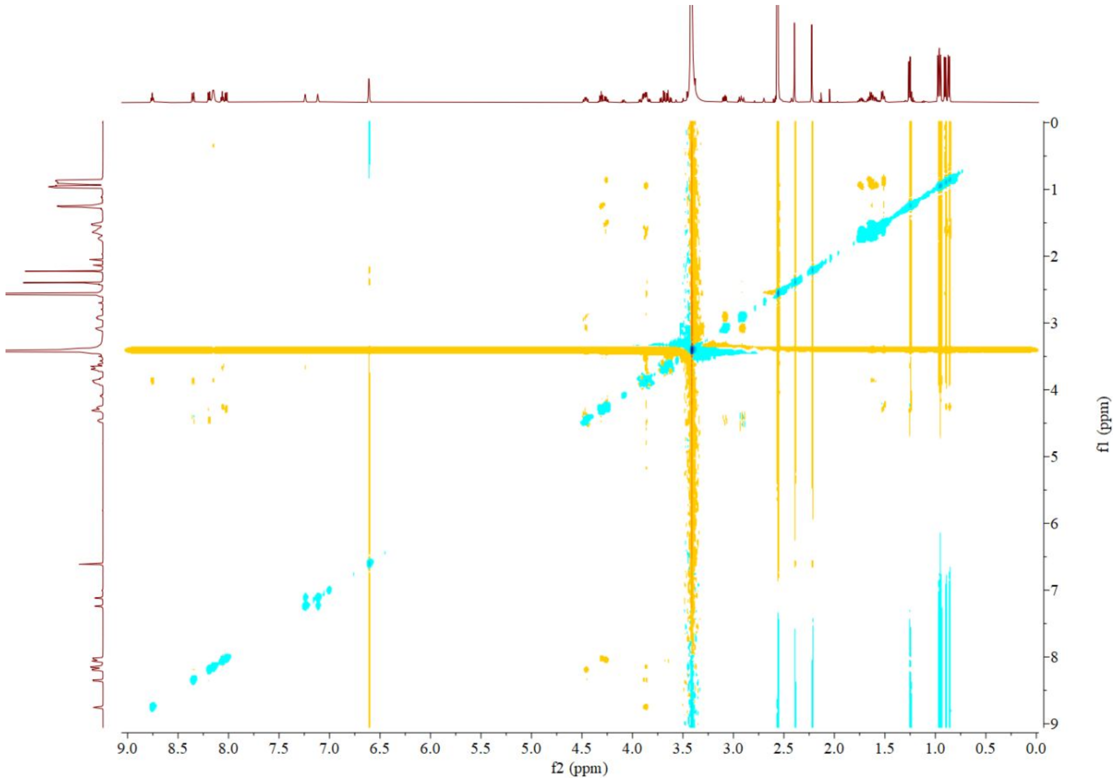

Figure S127. ^{1}H-^{1}H ROESY NMR Spectrum of **3k**.

^{1}H NMR Spectrum of **5a**.

^{1}H NMR (500 MHz, DMSO-d_6) δ 9.62 (s, 1H), 9.52 (s, 1H), 8.75 (t, J = 5.6 Hz, 1H), 8.32 (d, J = 8.1 Hz, 1H), 8.24 (d, J = 7.1 Hz, 1H), 8.14 (s, 2H), 8.04 (t, J = 5.8 Hz, 1H), 7.96 (d, J = 7.6 Hz, 1H), 7.22 – 7.19 (m, 1H), 7.14 (d, J = 8.4 Hz, 1H), 7.09 (s, 1H), 6.37 (d, J = 2.5 Hz, 1H), 6.22 (dd, J = 8.4, 2.5 Hz, 1H), 4.36 (td, J = 8.4, 5.8 Hz, 1H), 4.32 – 4.26 (m, 1H), 4.23 (q, J = 7.4 Hz, 1H), 3.87 (qd, J = 16.7, 7.4 Hz, 3H), 3.70 – 3.58 (m, 2H), 3.03 (dd, J = 13.1, 5.7 Hz, 1H), 2.81 (dd, J = 13.1, 8.8 Hz, 1H), 1.70 (dq, J = 12.7, 6.4 Hz, 1H), 1.65 – 1.54 (m, 3H), 1.49 (dd, J = 8.2, 6.4 Hz, 2H), 1.23 (d, J = 7.0 Hz, 3H), 0.93 (td, J = 8.0, 7.2, 1.8 Hz, 6H), 0.88 – 0.82 (m, 6H).

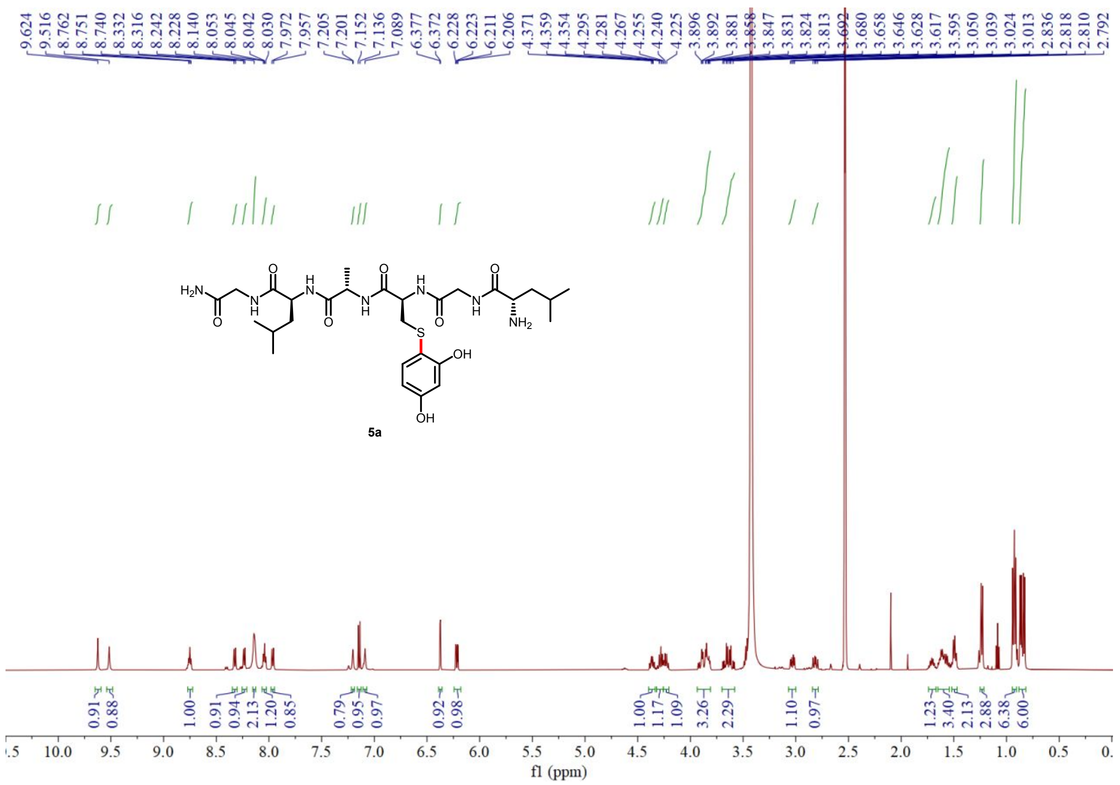

Figure S128. ^{1}H NMR Spectrum of **5a**.

^{1}H NMR Spectrum of **5b**.

^{1}H NMR (500 MHz, DMSO-d_6) δ 9.41 (s, 1H), 9.32 (s, 1H), 8.73 (t, J = 5.7 Hz, 1H), 8.30 (d, J = 8.0 Hz, 1H), 8.21 (d, J = 7.0 Hz, 1H), 8.13 – 8.10 (m, 2H), 8.01 (t, J = 5.8 Hz, 1H), 7.94 (d, J = 7.7 Hz, 1H), 7.17 (d, J = 2.1 Hz, 1H), 7.06 (d, J = 2.1 Hz, 1H), 7.01 (d, J = 0.9 Hz, 1H), 6.40 (s, 1H), 4.34 (td, J = 8.3, 5.8 Hz, 1H), 4.28 – 4.23 (m, 1H), 4.23 – 4.18 (m, 1H), 3.92 – 3.78 (m, 3H), 3.68 – 3.55 (m, 2H), 2.98 (dd, J = 13.1, 5.8 Hz, 1H), 2.79 (dd, J = 13.1, 8.7 Hz, 1H), 1.97 (d, J = 0.6 Hz, 3H), 1.73 – 1.64 (m, 1H), 1.57 (dddd, J = 21.8, 15.3, 8.7, 4.0 Hz, 3H), 1.46 (dd, J = 8.6, 6.7 Hz, 2H), 1.21 (d, J = 7.0 Hz, 3H), 0.90 (dd, J = 8.7, 6.5 Hz, 6H), 0.82 (dd, J = 15.0, 6.6 Hz, 6H).

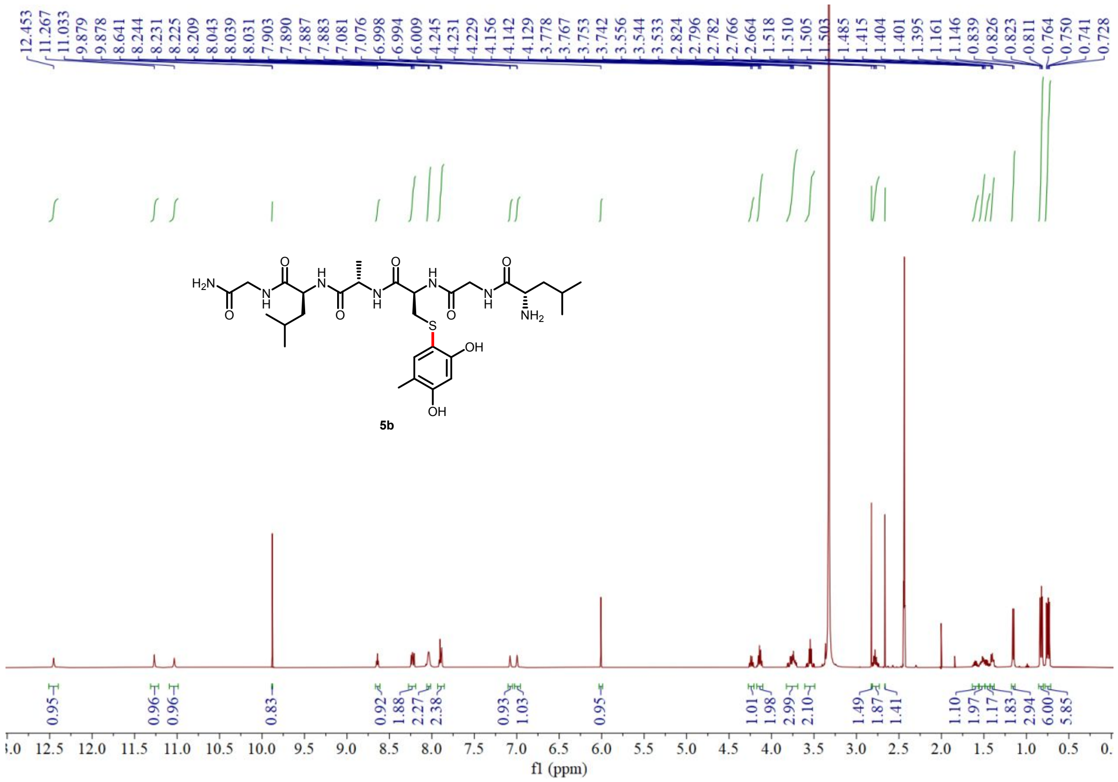

Figure S129. ^{1}H NMR Spectrum of **5b**.

^{1}H NMR Spectrum of **6c**.

^{1}H NMR (500 MHz, DMSO-d_6) δ 11.02 (s, 1H), 7.50 (dq, J = 8.0, 0.8 Hz, 1H), 7.36 (dq, J = 8.0, 0.8 Hz, 1H), 7.29 (dd, J = 3.2, 2.4 Hz, 1H), 7.04 (ddd, J = 8.0, 7.2, 1.2 Hz, 1H), 6.95 (ddd, J = 8.0, 7.2, 1.2 Hz, 1H), 6.39 (ddd, J = 3.2, 2.0, 0.8 Hz, 1H).

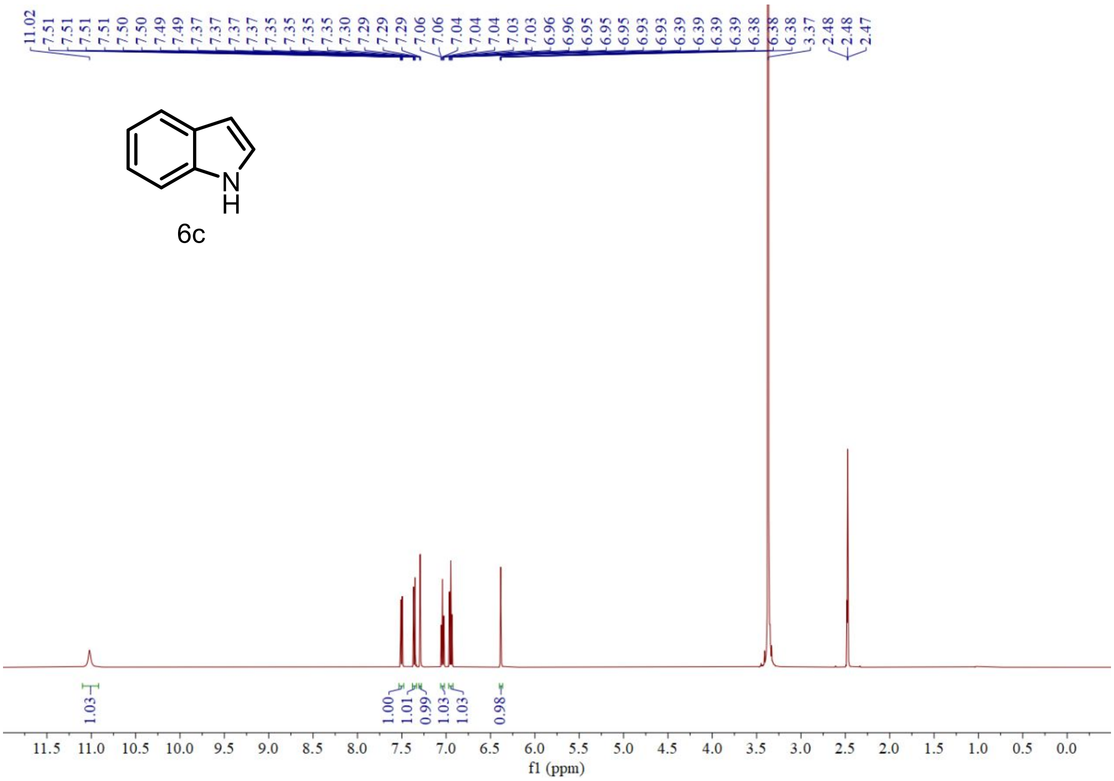

Figure S130. ^{1}H NMR Spectrum of **6c**.

^{1}H NMR Spectrum of **7c**.

^{1}H NMR (500 MHz, DMSO-d_6) δ 11.39 (d, J = 2.4 Hz, 1H), 8.69 (t, J = 5.6 Hz, 1H), 8.32 (d, J = 8.4 Hz, 1H), 8.20 (d, J = 7.2 Hz, 1H), 8.08 (d, J = 5.2 Hz, 2H), 7.99 (t, J = 6.0 Hz, 1H), 7.89 (d, J = 7.6 Hz, 1H), 7.54 (dd, J = 8.0, 1.2 Hz, 1H), 7.49 (d, J = 2.4 Hz, 1H), 7.39 (dt, J = 8.0, 1.2 Hz, 1H), 7.14 (s, 1H), 7.12 (ddd, J = 8.0, 7.2, 1.2 Hz, 1H), 7.06 (ddd, J = 8.0, 7.2, 1.2 Hz, 1H), 7.03 – 7.00 (m, 1H), 4.41 (td, J = 8.8, 5.6 Hz, 1H), 4.23 (q, J = 7.2 Hz, 1H), 4.17 (q, J = 7.6 Hz, 1H), 3.89 – 3.76 (m, 2H), 3.57 (qd, J = 16.8, 5.3 Hz, 2H), 2.90 (dd, J = 12.0, 5.6 Hz, 1H), 2.70 (dd, J = 12.0, 9.2 Hz, 1H), 1.67 – 1.63 (m, 1H), 1.61 – 1.47 (m, 4H), 1.41 (dd, J = 8.7, 6.4 Hz, 2H), 1.16 (d, J = 7.2 Hz, 3H), 0.87 (dd, J = 10.0, 6.4 Hz, 6H), 0.75 (dd, J = 17.2, 6.4 Hz, 6H).

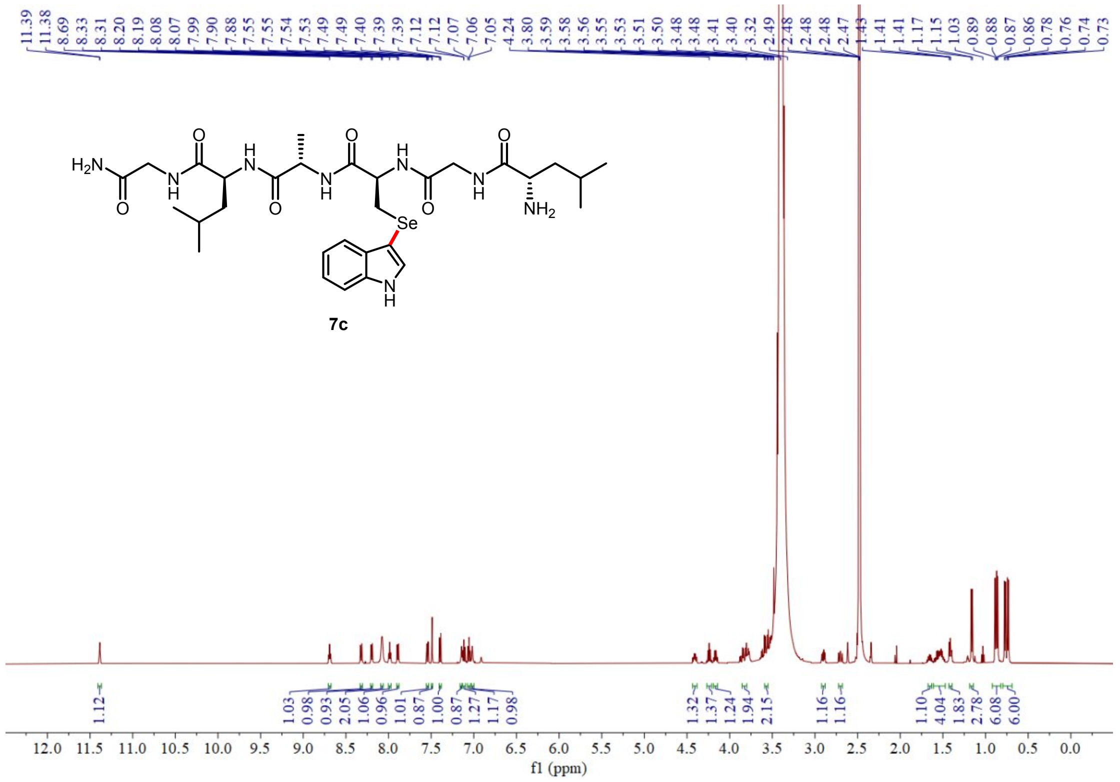

Figure S131. ^{1}H NMR Spectrum of **7c**.

^{1}H NMR Spectrum of **7d**.

^{1}H NMR (500 MHz, DMSO-d_6) δ 11.32 (s, 1H), 8.69 (t, *J* = 5.6 Hz, 1H), 8.33 (d, *J* = 8.1 Hz, 1H), 8.10 (d, *J* = 7.4 Hz, 1H), 8.07 (d, *J* = 4.5 Hz, 2H), 7.98 (t, *J* = 5.8 Hz, 1H), 7.90 (d, *J* = 7.7 Hz, 1H), 7.46 – 7.40 (m, 1H), 7.29 – 7.23 (m, 1H), 7.15 – 7.11 (m, 1H), 7.04 – 6.96 (m, 3H), 4.33 (ddd, *J* = 9.9, 8.1, 4.9 Hz, 1H), 4.22 (p, *J* = 7.0 Hz, 1H), 4.19 – 4.13 (m, 1H), 3.89 – 3.76 (m, 3H), 3.56 (qd, *J* = 16.8, 5.8 Hz, 3H), 2.85 (dd, *J* = 11.9, 4.8 Hz, 1H), 2.64 (dd, *J* = 12.0, 9.9 Hz, 1H), 2.43 (s, 3H), 1.70 – 1.62 (m, 1H), 1.60 – 1.47 (m, 3H), 1.43 – 1.34 (m, 2H), 1.14 (d, *J* = 7.0 Hz, 3H), 0.88 (dd, *J* = 9.1, 6.5 Hz, 6H), 0.76 (dd, *J* = 21.0, 6.5 Hz, 6H).

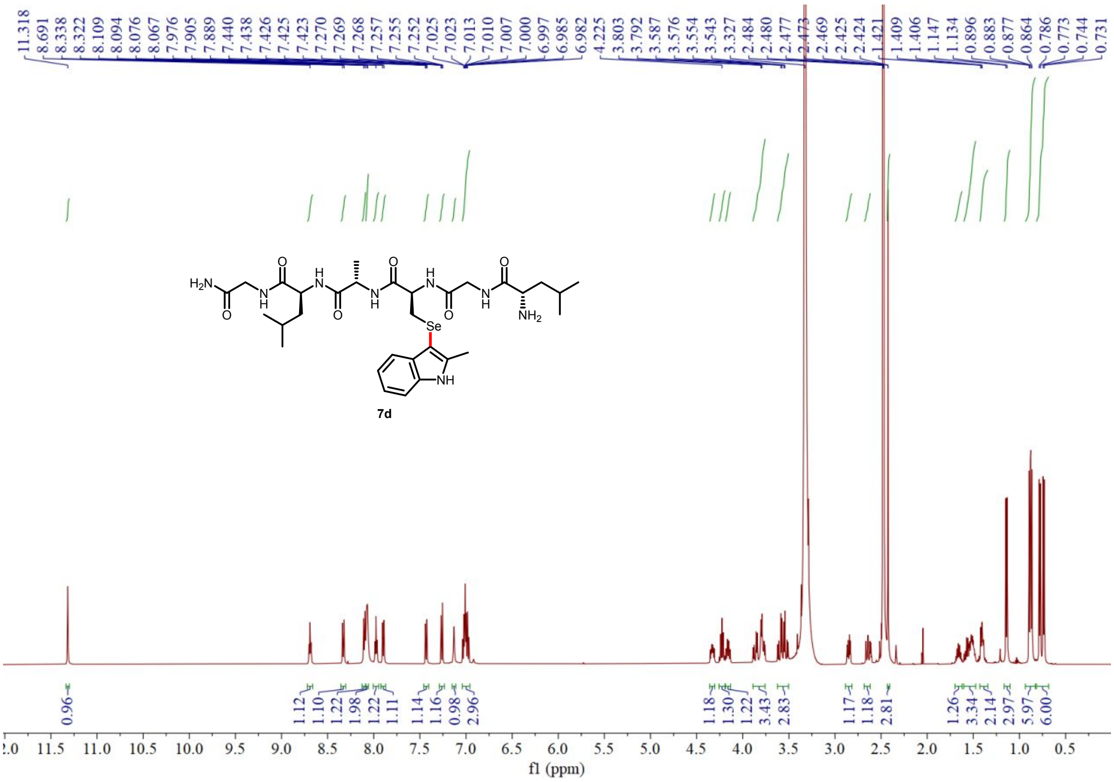

Figure S132. ^{1}H NMR Spectrum of **7d**.

^{1}H NMR Spectrum of **7g**.

^{1}H NMR (500 MHz, DMSO-d_6) δ 9.95 (s, 1H), 8.75 (t, J = 5.6 Hz, 1H), 8.42 (d, J = 8.1 Hz, 1H), 8.40 (dd, J = 8.5, 1.0 Hz, 1H), 8.27 (d, J = 7.2 Hz, 1H), 8.16 (s, 2H), 8.08 – 8.02 (m, 1H), 7.90 – 7.84 (m, 2H), 7.56 (ddd, J = 8.4, 6.8, 1.4 Hz, 1H), 7.37 (ddd, J = 8.0, 6.8, 1.1 Hz, 1H), 7.30 (d, J = 8.8 Hz, 1H), 7.26 – 7.21 (m, 1H), 7.11 (d, J = 2.1 Hz, 1H), 4.55 (td, J = 8.5, 5.6 Hz, 1H), 4.35 – 4.28 (m, 1H), 4.28 – 4.23 (m, 1H), 3.86 (dd, J = 5.9, 1.6 Hz, 3H), 3.72 – 3.60 (m, 2H), 3.10 (dd, J = 11.8, 5.5 Hz, 1H), 3.00 (dd, J = 11.8, 9.1 Hz, 1H), 1.78 – 1.69 (m, 1H), 1.68 – 1.55 (m, 3H), 1.50 (t, J = 7.3 Hz, 2H), 1.25 (d, J = 7.1 Hz, 3H), 0.96 (dd, J = 9.3, 6.5 Hz, 6H), 0.84 (dd, J = 17.1, 6.6 Hz, 6H).

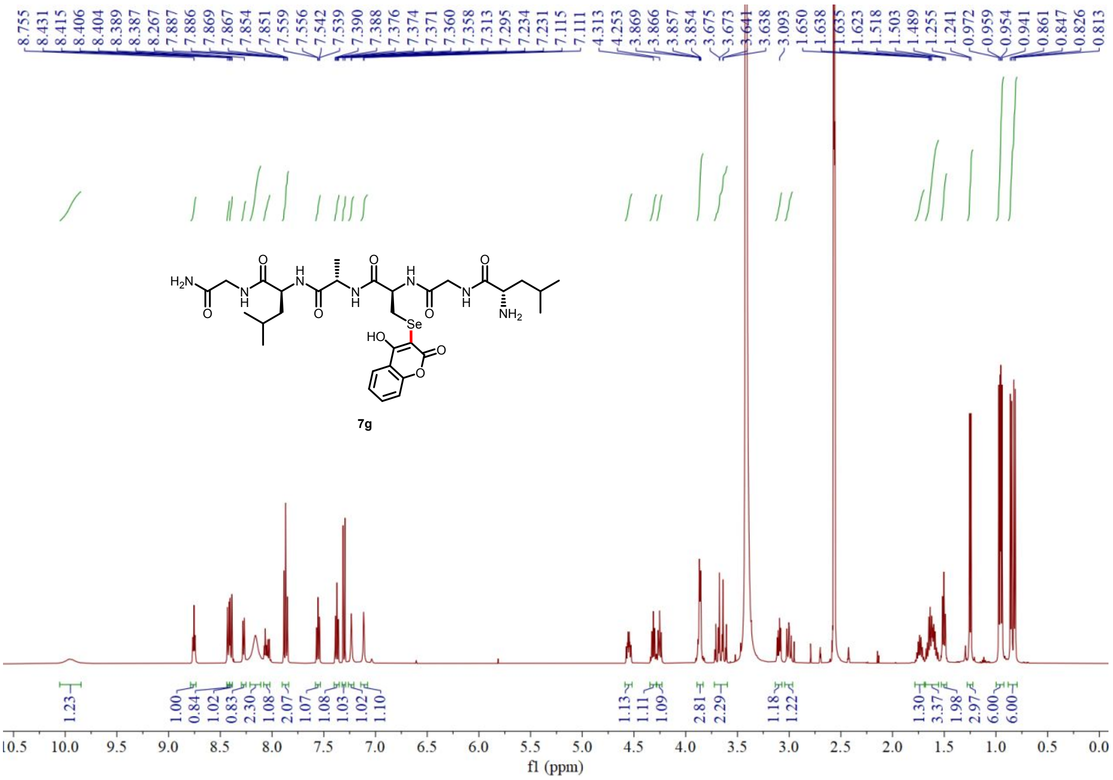

Figure S133. ^{1}H NMR Spectrum of **7g**.

^1H NMR Spectrum of **6h**.

^1H NMR (500 MHz, DMSO-d_6) δ 6.98 (t, J = 8.0 Hz, 1H), 5.59 (d, J = 8.0 Hz, 2H), 5.25 (s, 4H).

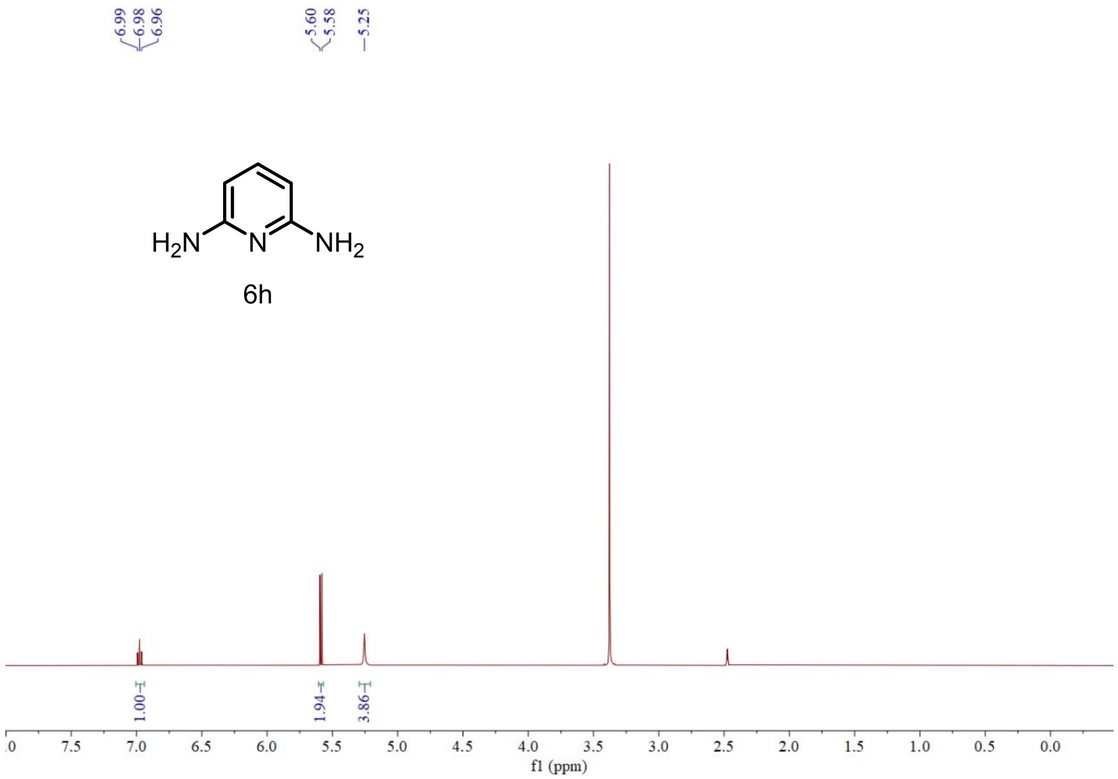

Figure S134. ^1H NMR Spectrum of **6h**.

^{1}H NMR Spectrum of **7h**.

^{1}H NMR (500 MHz, DMSO-d_6) δ 8.73 (t, J = 5.6 Hz, 1H), 8.32 (d, J = 8.4 Hz, 1H), 8.24 (d, J = 7.2 Hz, 1H), 8.13 – 8.07 (m, 2H), 8.01 – 7.96 (m, 2H), 7.70 (d, J = 8.4 Hz, 1H), 7.43 (s, 2H), 7.21 – 7.13 (m, 2H), 7.03 (d, J = 2.0 Hz, 1H), 5.86 (d, J = 8.4 Hz, 1H), 4.40 (td, J = 8.8, 5.2 Hz, 1H), 4.26 – 4.16 (m, 2H), 3.86 (dd, J = 16.8, 6.0 Hz, 1H), 3.81-3.75 (m, 1H), 3.61 – 3.57 (m, 2H), 2.91 (dd, J = 12.4, 5.2 Hz, 1H), 2.70 (dd, J = 12.4, 9.2 Hz, 1H), 1.67 (dt, J = 13.2, 6.8 Hz, 1H), 1.62-1.48 (m, 3H), 1.47-1.41 (m, 2H), 1.23 (t, J = 6.8 Hz, 3H), 1.19 (d, J = 7.2 Hz, 2H), 0.88 (dd, J = 10.0, 6.4 Hz, 6H), 0.81 (dd, J = 22.4, 6.4 Hz, 6H).

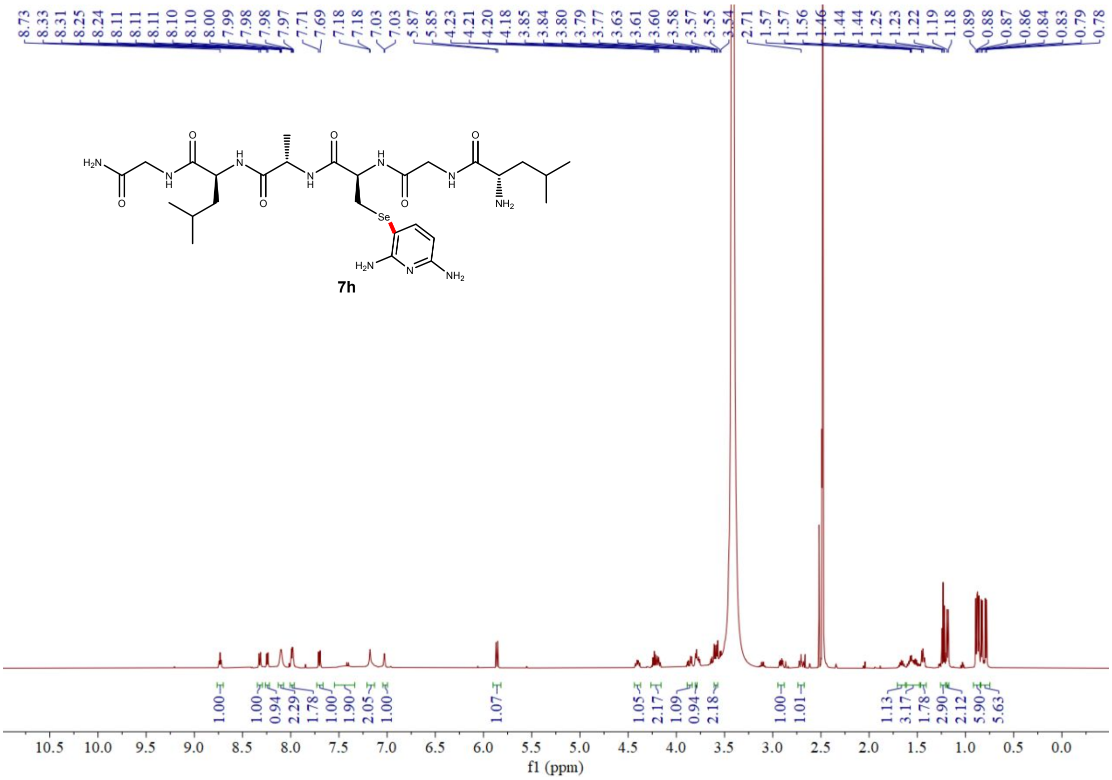

Figure S135. ^{1}H NMR Spectrum of **7h**.

^{1}H NMR Spectrum of **10a$_1$**.

^{1}H NMR (500 MHz, DMSO-d_6) δ 9.57 (s, 1H), 9.48 (s, 1H), 9.46 (s, 1H), 8.66 (t, J = 5.5 Hz, 1H), 8.33 (d, J = 8.0 Hz, 1H), 8.18 (d, J = 7.1 Hz, 1H), 8.07 (s, 2H), 7.98 (t, J = 5.8 Hz, 1H), 7.94 (d, J = 7.7 Hz, 1H), 7.52 (d, J = 16.2 Hz, 1H), 7.37 (d, J = 8.7 Hz, 2H), 7.15 (s, 1H), 7.08 – 7.02 (m, 1H), 6.82 (d, J = 16.2 Hz, 1H), 6.75 (d, J = 8.7 Hz, 2H), 6.59 (d, J = 2.5 Hz, 1H), 6.31 (d, J = 2.5 Hz, 1H), 4.45 (td, J = 8.4, 5.7 Hz, 1H), 4.23 (p, J = 7.1 Hz, 1H), 4.17 (q, J = 7.5 Hz, 1H), 3.81 (dd, J = 5.5, 3.6 Hz, 3H), 3.66 – 3.52 (m, 3H), 2.93 (dd, J = 11.8, 5.6 Hz, 1H), 2.81 (dd, J = 11.8, 9.0 Hz, 1H), 1.68 – 1.59 (m, 1H), 1.59 – 1.47 (m, 3H), 1.42 (t, J = 7.3 Hz, 2H), 1.19 (d, J = 7.1 Hz, 3H), 0.86 (dd, J = 9.0, 6.4 Hz, 6H), 0.77 (dd, J = 17.9, 6.5 Hz, 6H).

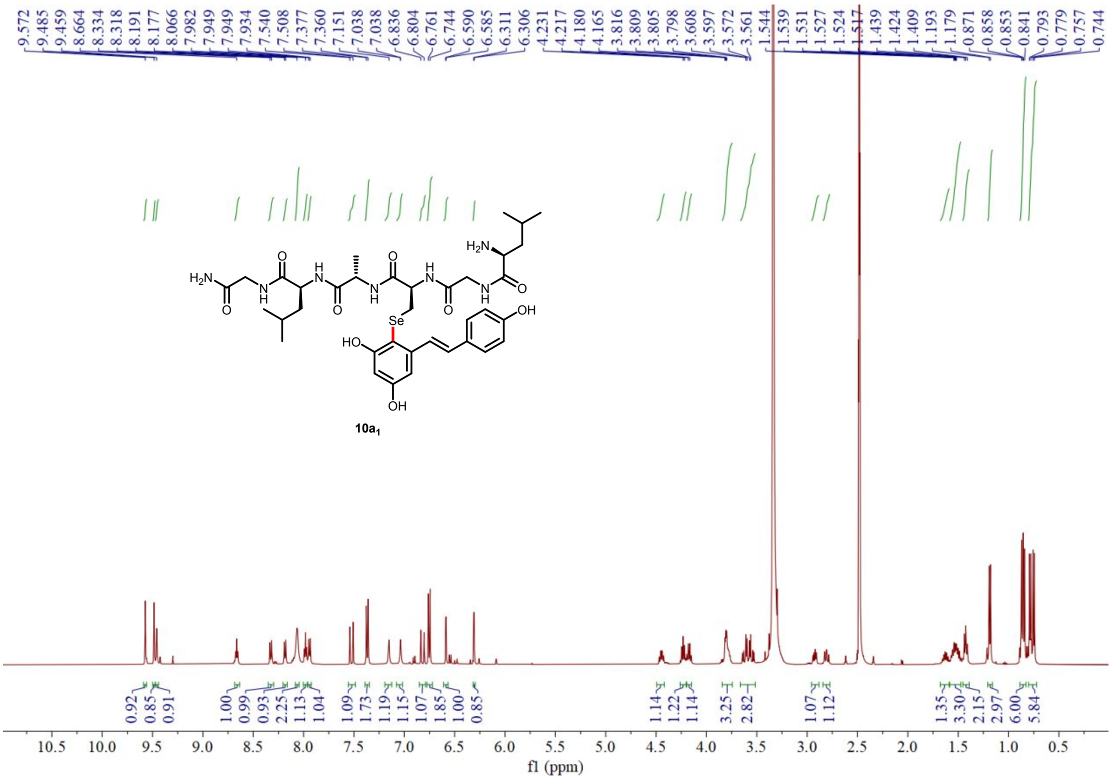

Figure S136. ^{1}H NMR Spectrum of **10a$_1$**.

^{1}H NMR Spectrum of **10b$_1$**.

^{1}H NMR (500 MHz, DMSO-d_6) δ 13.94 (s, 1H), 11.36 (s, 1H), 9.70 (s, 2H), 8.76 (t, J = 5.6 Hz, 1H), 8.42 (s, 1H), 8.38 (d, J = 8.2 Hz, 1H), 8.34 (d, J = 6.8 Hz, 1H), 8.15 (d, J = 5.3 Hz, 2H), 8.05 – 8.00 (m, 2H), 7.43 (d, J = 8.6 Hz, 2H), 7.23 (d, J = 2.1 Hz, 1H), 7.12 (d, J = 2.1 Hz, 1H), 6.91 – 6.87 (m, 2H), 6.61 (s, 1H), 4.53 (td, J = 8.2, 6.7 Hz, 1H), 4.27 (qd, J = 7.3, 3.0 Hz, 2H), 3.91 – 3.83 (m, 3H), 3.76 – 3.59 (m, 3H), 3.13 (dq, J = 7.9, 4.5, 3.7 Hz, 2H), 1.76 – 1.69 (m, 1H), 1.69 – 1.57 (m, 3H), 1.57 – 1.52 (m, 2H), 1.27 (d, J = 7.1 Hz, 3H), 0.95 (dd, J = 7.5, 6.4 Hz, 6H), 0.88 (dd, J = 15.7, 6.5 Hz, 6H).

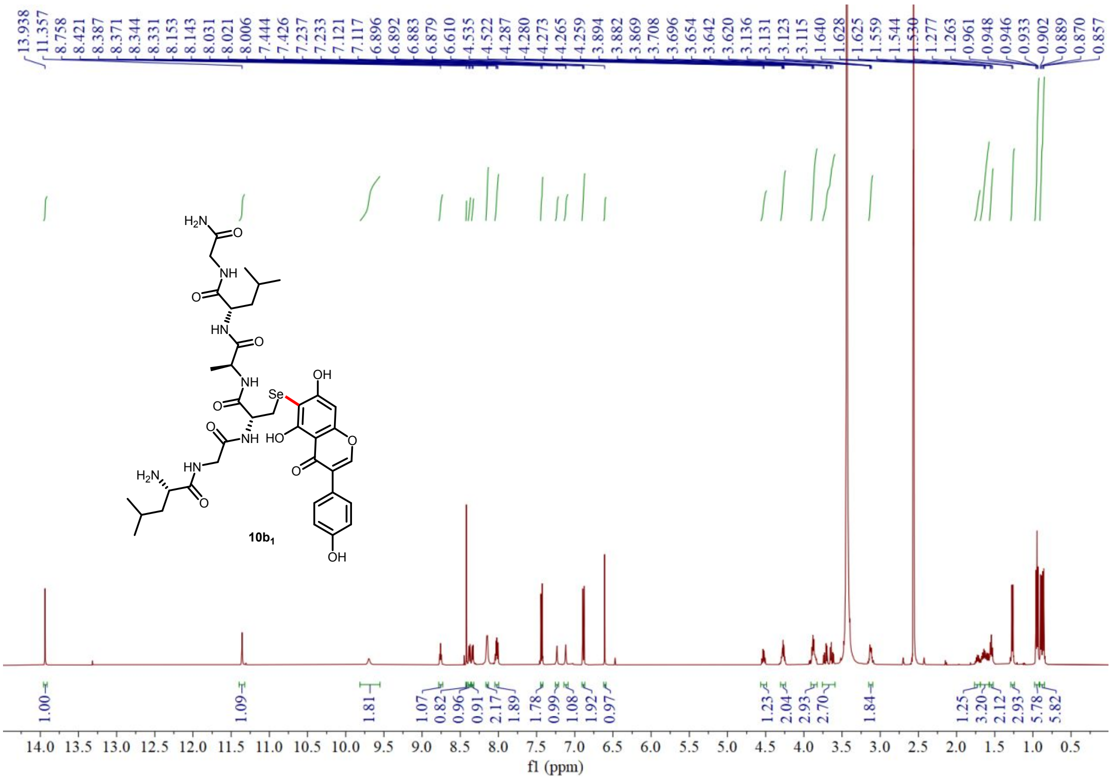

Figure S137. ^{1}H NMR Spectrum of **10b$_1$**.

HSQC NMR Spectrum of **10b$_1$**.

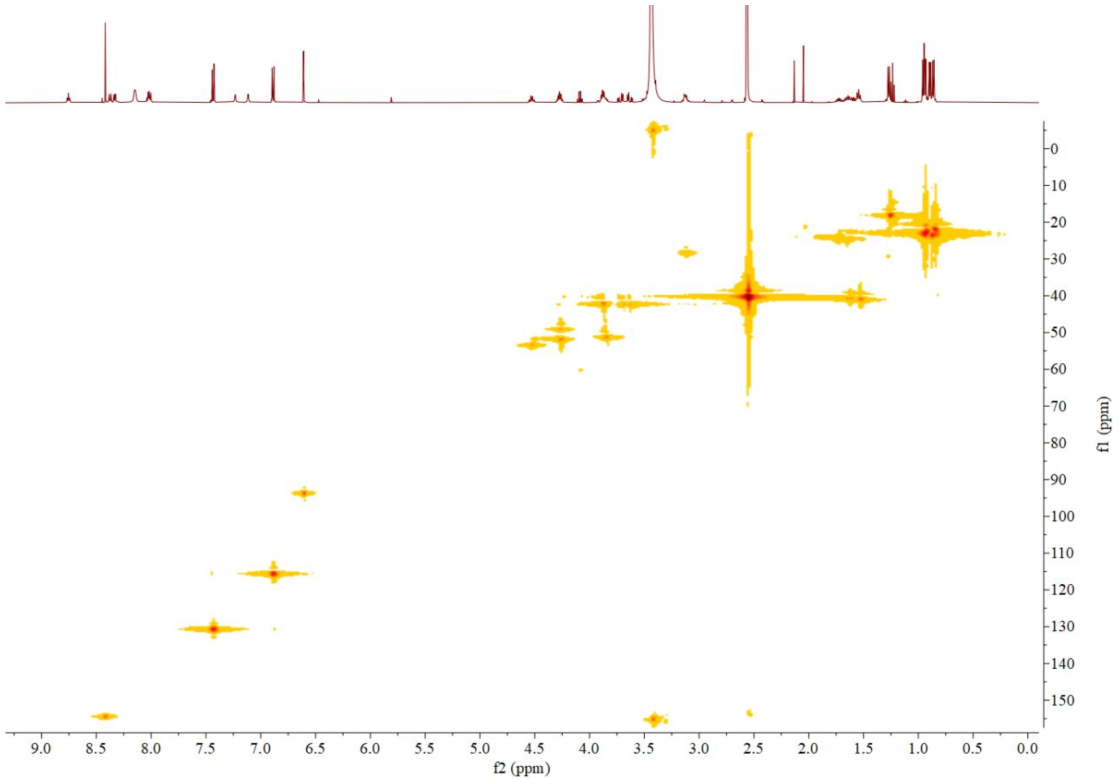

Figure S138. HSQC NMR Spectrum of **10b$_1$**.

HMBC NMR Spectrum of **$10b_1$**.

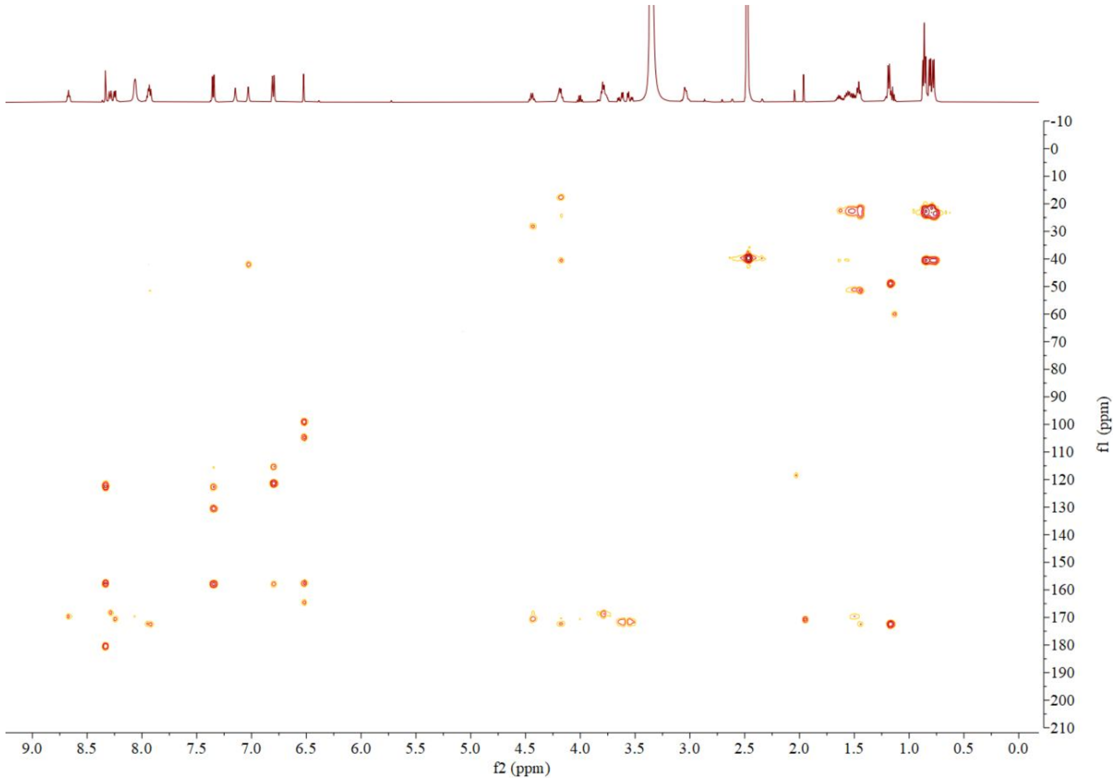

Figure S139. HMBC NMR Spectrum of **$10b_1$**.

^{1}H NMR Spectrum of **9d**.

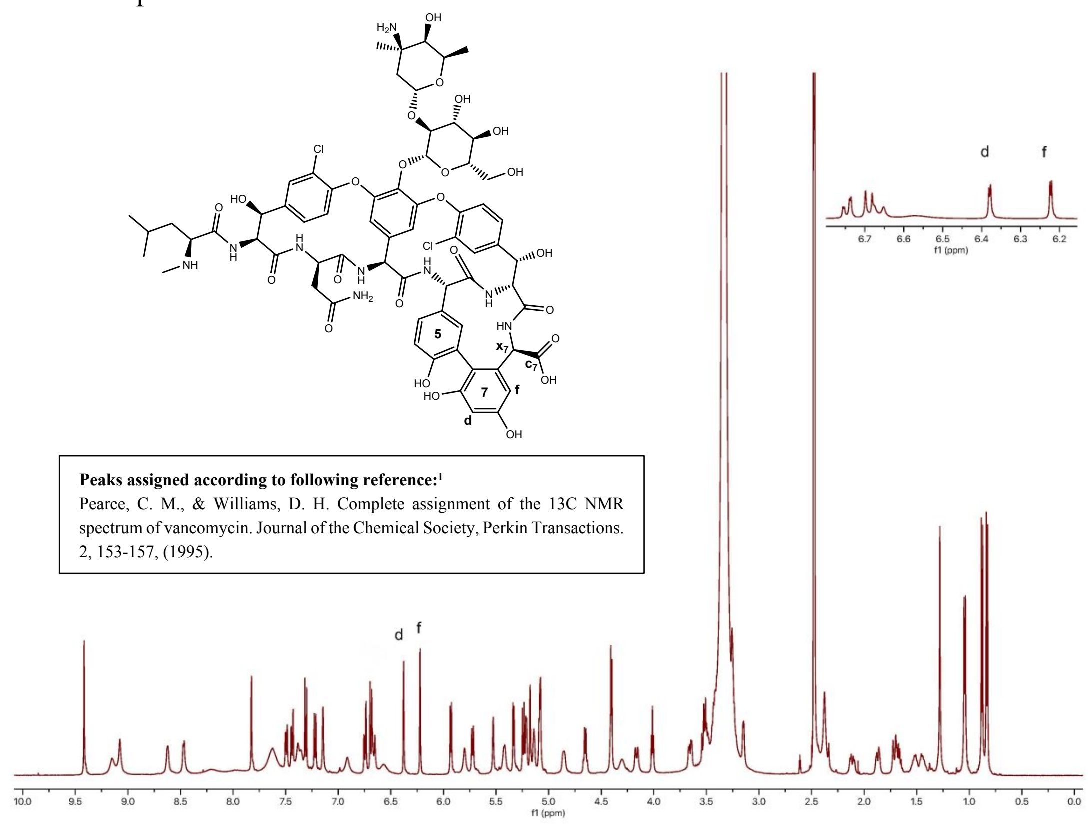

Figure S140. ^{1}H NMR Spectrum of **9d**.

^{1}H NMR Spectrum of **10d**.

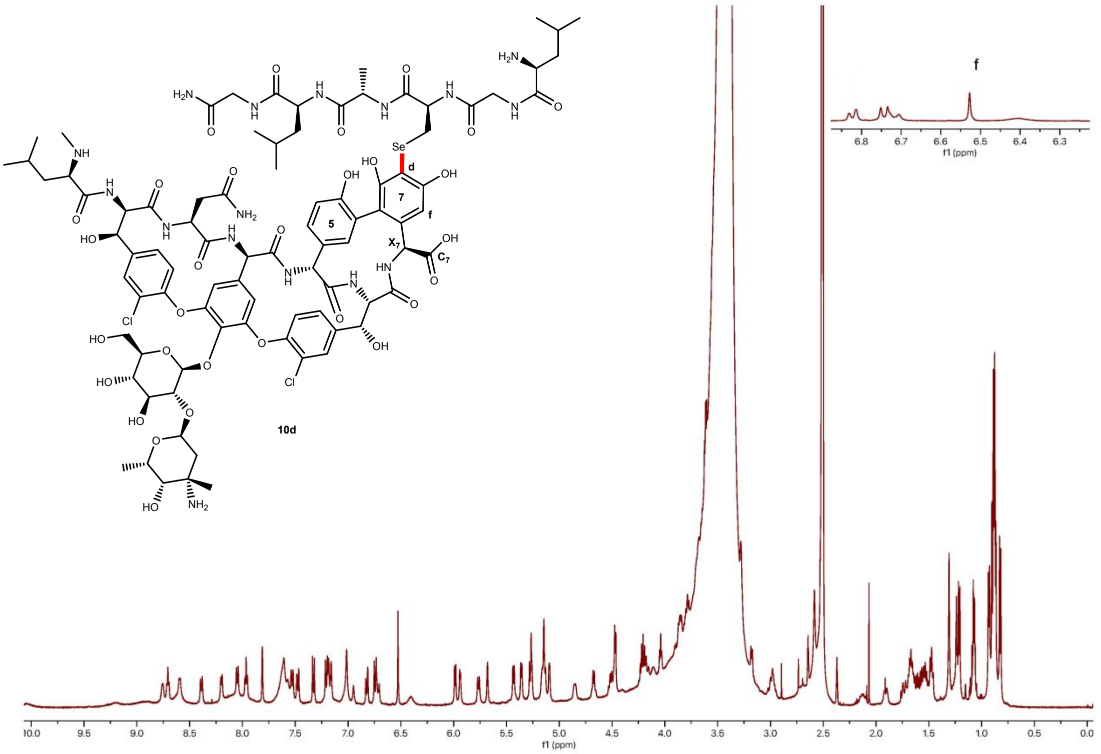

Figure S141. ^{1}H NMR Spectrum of **10d**.

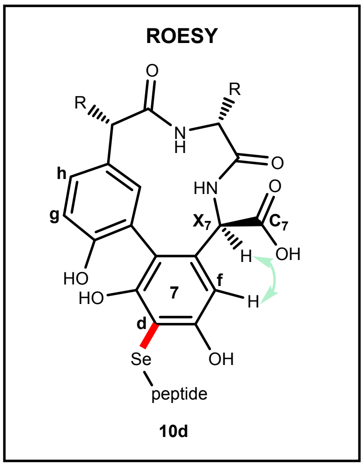

^{1}H-^{1}H ROESY NMR Spectrum of **10d**.

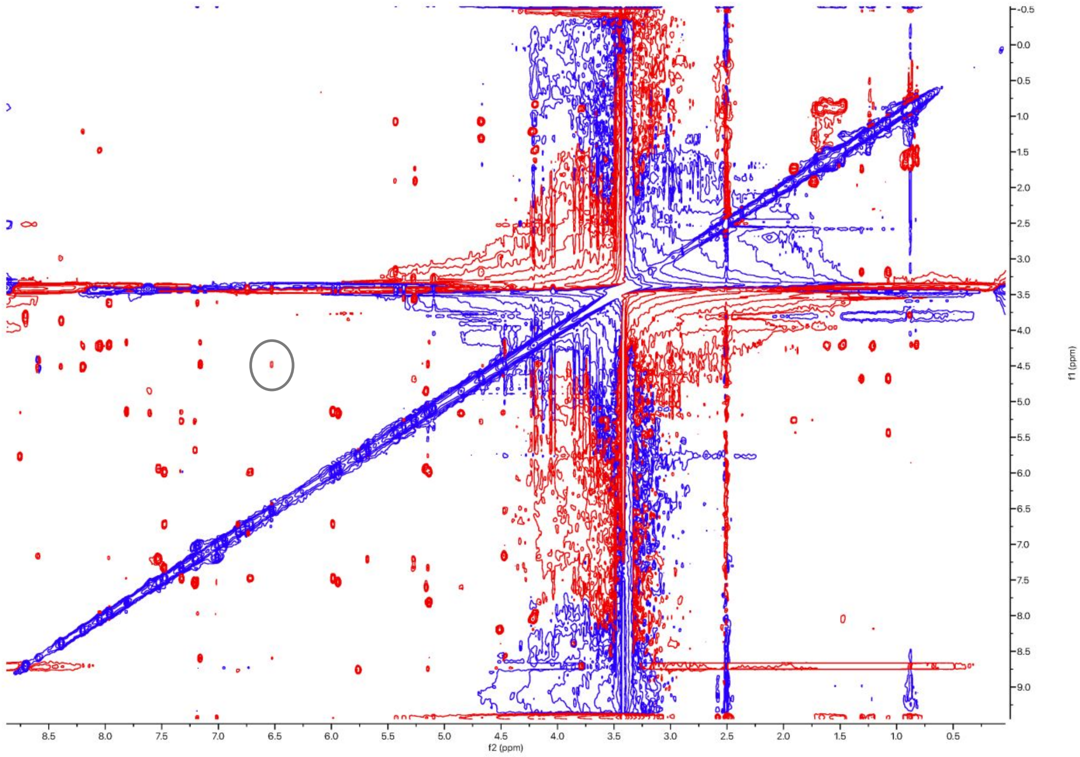

Figure S142. ^{1}H-^{1}H ROESY NMR of **10d**.

^{1}H NMR Spectrum of **10e**.

^{1}H NMR (500 MHz, DMSO-d_6) δ 10.98 (d, J = 2.7 Hz, 1H), 8.93 (s, 1H), 8.79 (t, J = 5.7 Hz, 1H), 8.42 (d, J = 8.0 Hz, 1H), 8.23 (d, J = 7.0 Hz, 1H), 8.17 (d, J = 5.3 Hz, 2H), 8.13 (d, J = 7.6 Hz, 1H), 8.05 (t, J = 5.8 Hz, 1H), 7.80 (d, J = 5.8 Hz, 2H), 7.29 (d, J = 8.6 Hz, 1H), 7.28 – 7.26 (m, 1H), 7.23 (d, J = 2.6 Hz, 1H), 7.13 (d, J = 2.1 Hz, 1H), 6.85 (d, J = 8.6 Hz, 1H), 4.61 (td, J = 8.1, 6.2 Hz, 1H), 4.33 (p, J = 7.1 Hz, 1H), 4.26 (q, J = 7.5 Hz, 1H), 3.88 (dd, J = 5.8, 2.1 Hz, 2H), 3.74 – 3.62 (m, 2H), 3.33 – 3.27 (m, 2H), 3.22 – 3.16 (m, 2H), 3.08 (dd, J = 11.9, 6.1 Hz, 1H), 3.01 (dd, J = 11.8, 8.2 Hz, 1H), 1.73 (dq, J = 13.5, 6.7 Hz, 1H), 1.69 – 1.55 (m, 3H), 1.52 (t, J = 7.3 Hz, 2H), 1.28 (d, J = 7.1 Hz, 3H), 0.95 (dd, J = 7.8, 6.5 Hz, 6H), 0.87 (dd, J = 18.1, 6.6 Hz, 6H).

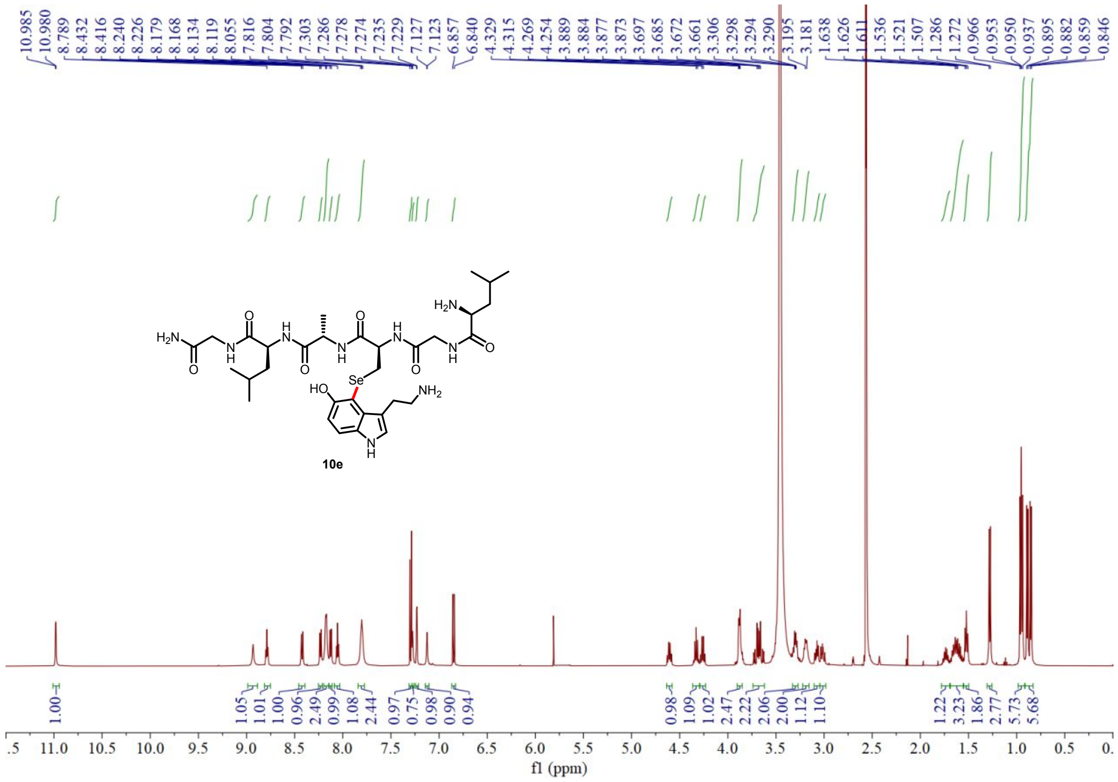

Figure S143. ^{1}H NMR Spectrum of **10e**.

^{1}H NMR Spectrum of **10f**.

^{1}H NMR (500 MHz, DMSO-d_6) δ 14.97 (s, 2H), 12.03 (s, 1H), 10.89 (s, 1H), 8.70 (t, J = 5.7 Hz, 1H), 8.42 – 8.37 (m, 3H), 8.28 (dd, J = 7.8, 4.3 Hz, 2H), 8.15 (d, J = 7.7 Hz, 1H), 8.08 (s, 2H), 7.99 (t, J = 5.7 Hz, 1H), 7.20 (d, J = 2.2 Hz, 1H), 7.06 (d, J = 8.7 Hz, 1H), 7.04 – 7.00 (m, 1H), 4.42 (q, J = 7.7 Hz, 1H), 4.30 – 4.21 (m, 2H), 3.86 (dd, J = 16.8, 6.0 Hz, 1H), 3.76 (dd, J = 16.7, 5.3 Hz, 2H), 3.66 – 3.56 (m, 2H), 3.08 (dd, J = 12.2, 7.7 Hz, 1H), 3.02 – 2.96 (m, 4H), 1.68 – 1.56 (m, 3H), 1.56 – 1.43 (m, 4H), 1.17 (d, J = 7.1 Hz, 3H), 0.84 (dd, J = 11.3, 6.5 Hz, 6H), 0.82 – 0.72 (m, 6H).

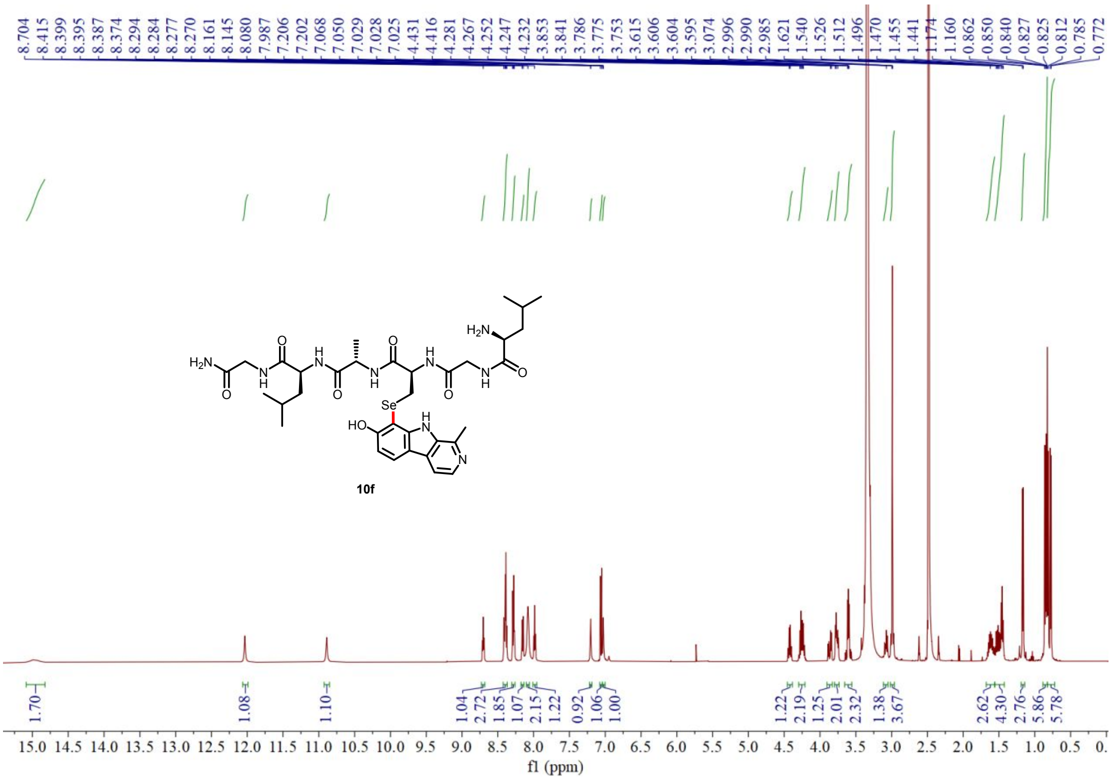

Figure S144. ^{1}H NMR Spectrum of **10f**.

COSY NMR Spectrum of **10f**.

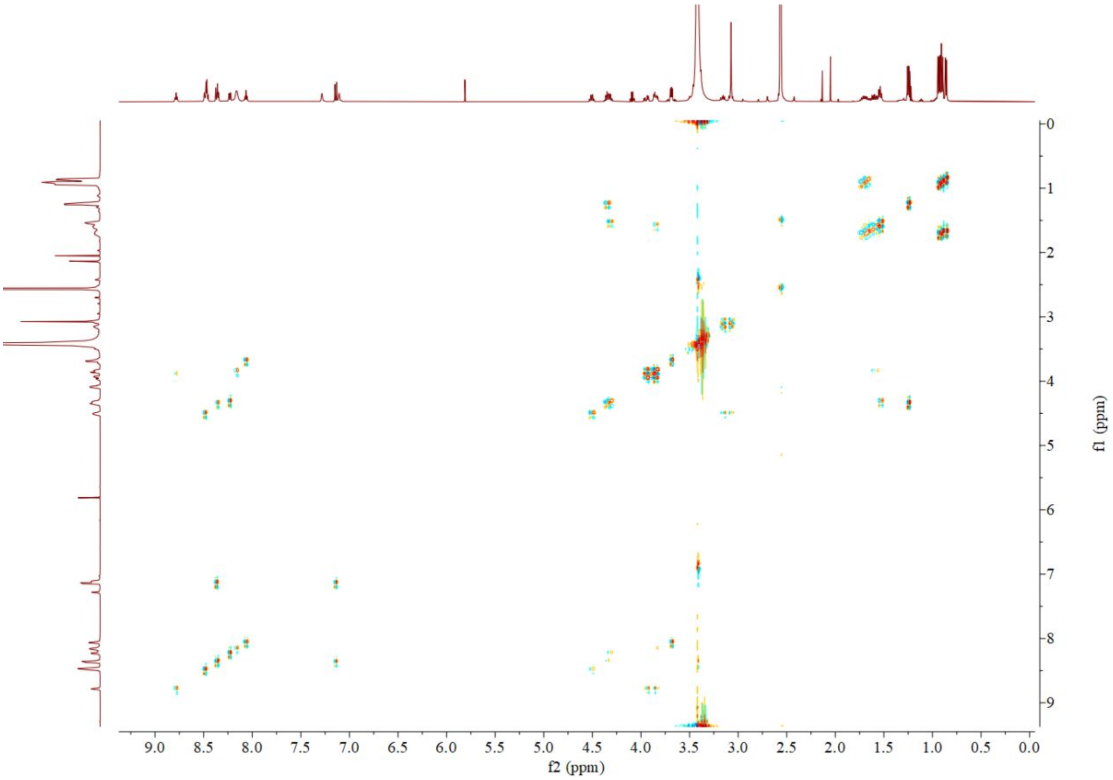

Figure S145. COSY NMR Spectrum of **10f**.

15. HRMS of Protein and Protein Conjugates

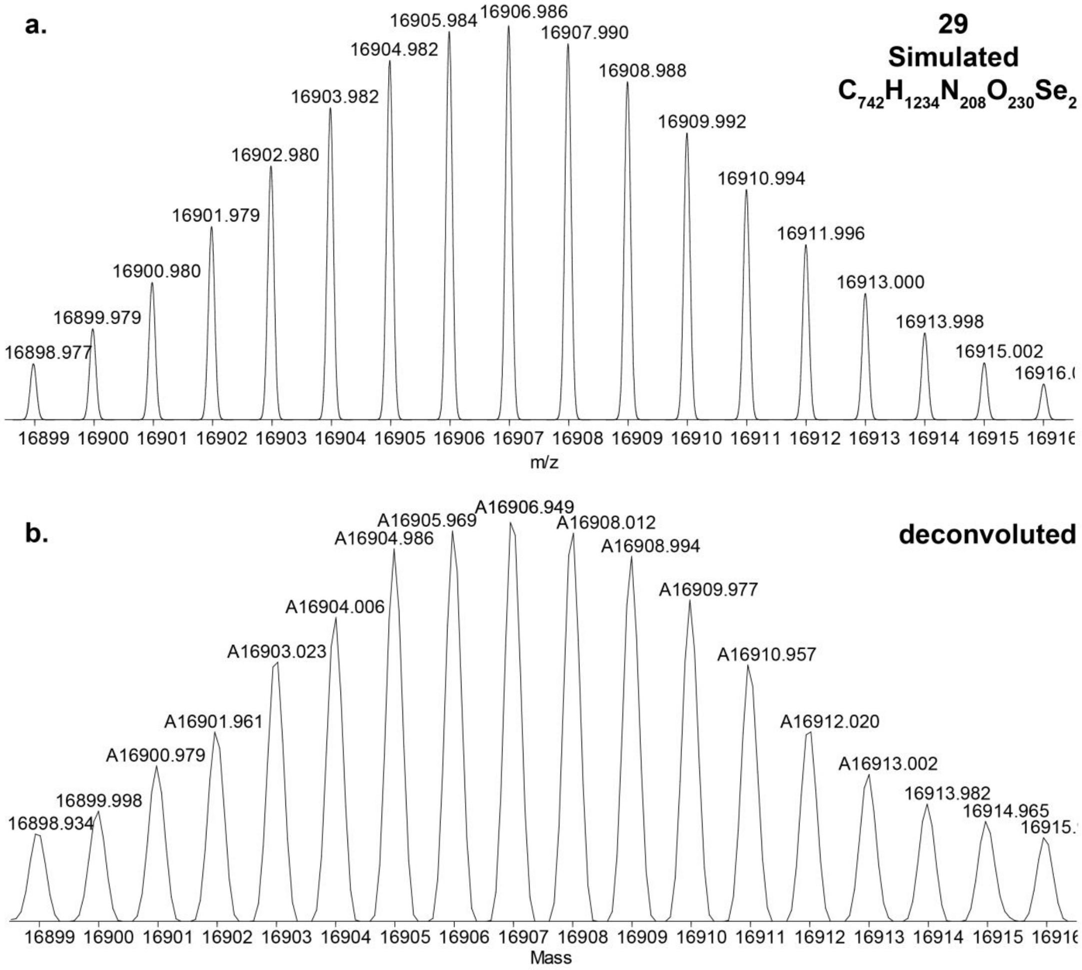

Figure S146. HR-MS analysis of Ub(2-76)(Q2U), **29**. **a**. The simulated HR-MS of reduced form of Ub(2-76)(Q2U), **29**, with chemical formula $C_{742}H_{1234}N_{208}O_{230}Se_2$ is shown; **b**. The deconvoluted HR-MS of **29**.

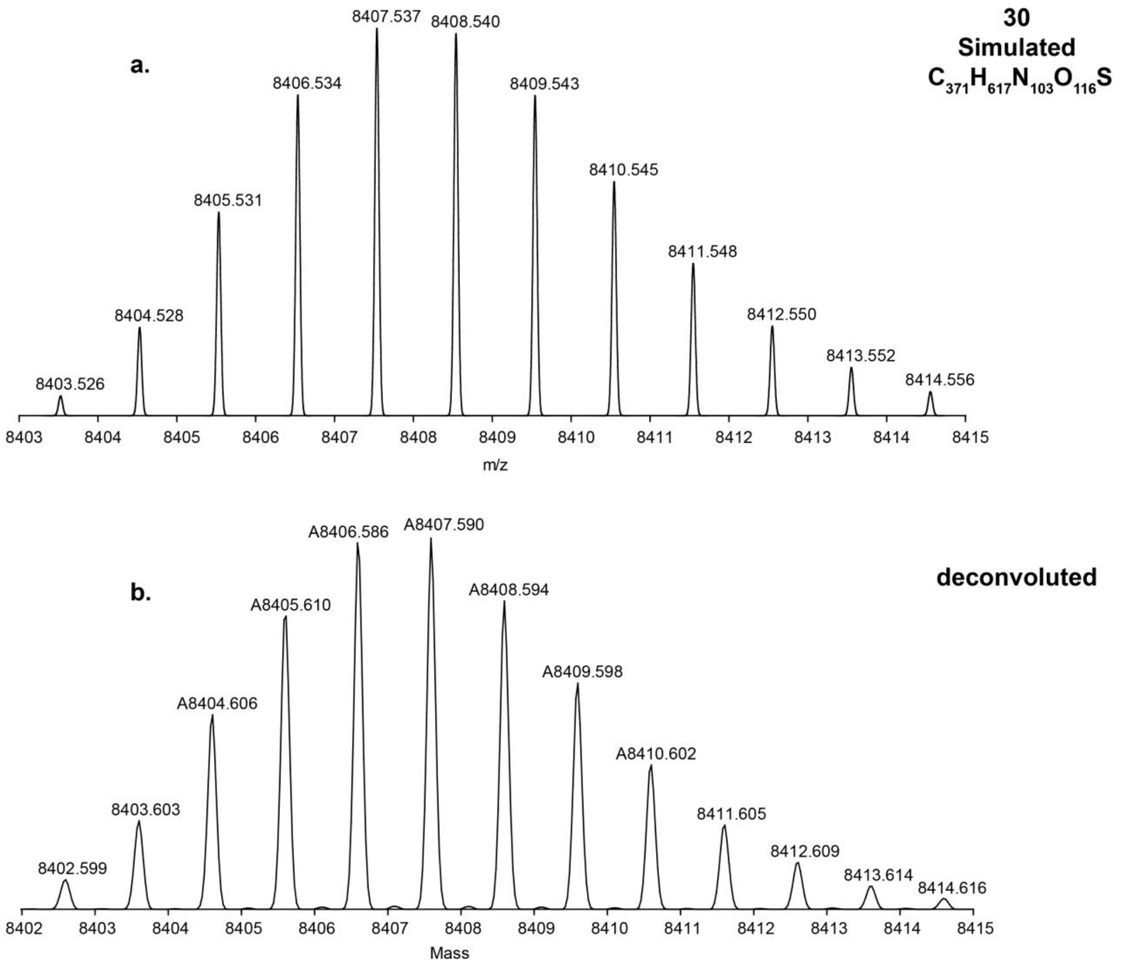

Figure S147. HR-MS analysis of Ub(2-76)(Q2C), **30**. **a**. The simulated HR-MS of reduced form of Ub(2-76)(Q2C), **30**, with chemical formula $C_{371}H_{617}N_{103}O_{116}S$ is shown; **b**. The deconvoluted HR-MS of **30**.

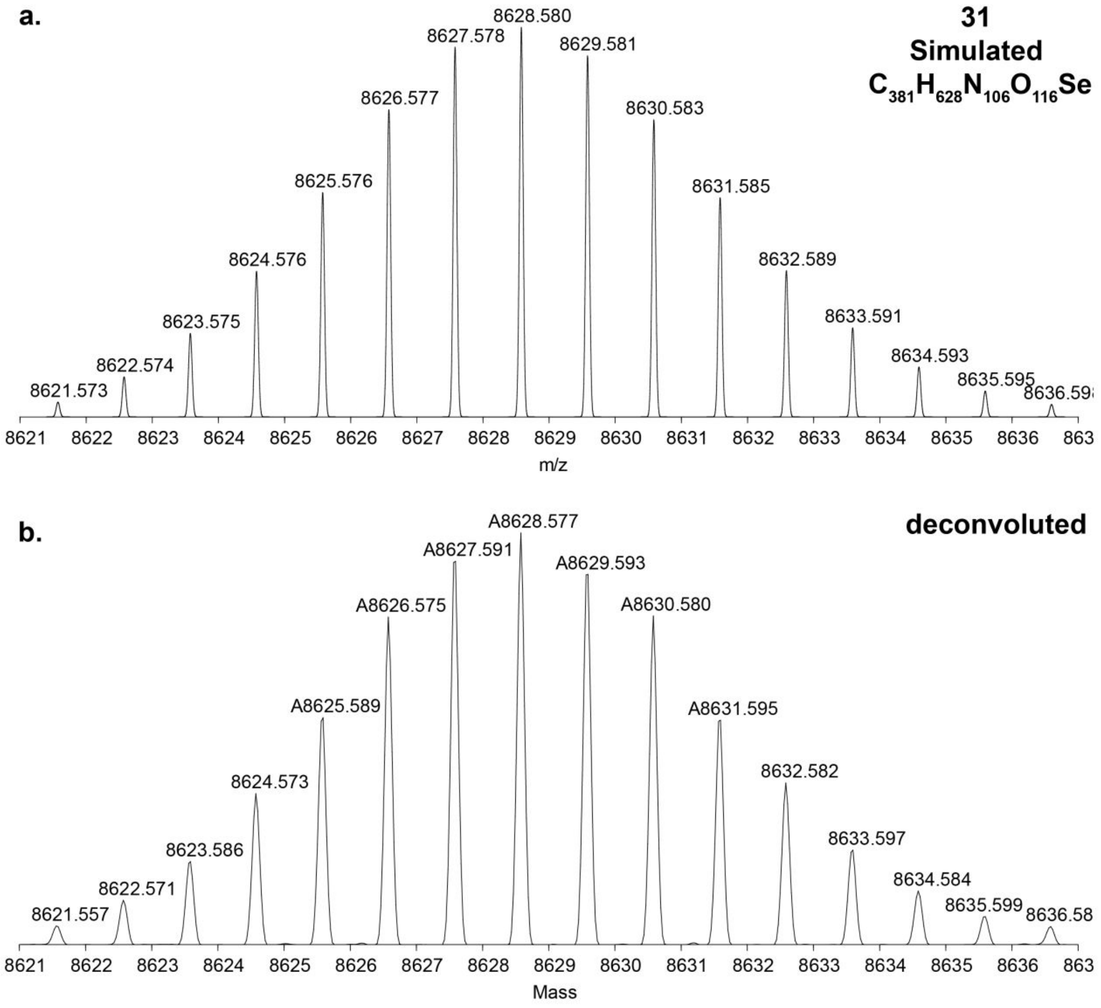

Figure S148. HR-MS analysis of serotoninated-Ub(2-76)(Q2U), **31**. **a**. The simulated HR-MS of reduced form of serotoninated-Ub(2-76)(Q2U), **31**, with chemical formula $C_{381}H_{628}N_{106}O_{116}Se$ is shown; **b**. The deconvoluted HR-MS of **31**.

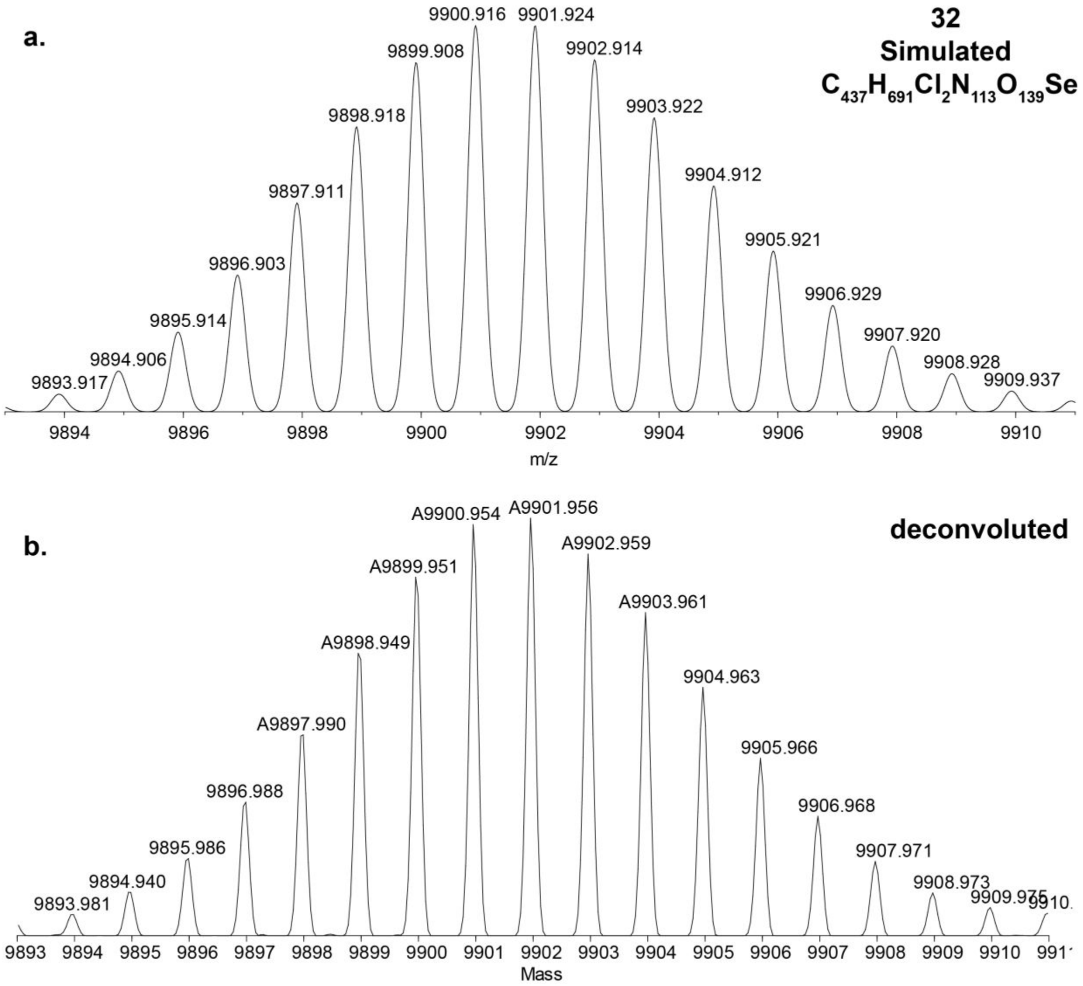

Figure S149. HR-MS analysis of vancomycinated-Ub(2-76)(Q2U), **32**. **a**. The simulated HR-MS of reduced form of vancomycinated-Ub(2-76)(Q2U), **32**, with chemical formula $C_{437}H_{691}Cl_2N_{113}O_{139}Se$ is shown; **b**. The deconvoluted HR-MS of **32**.

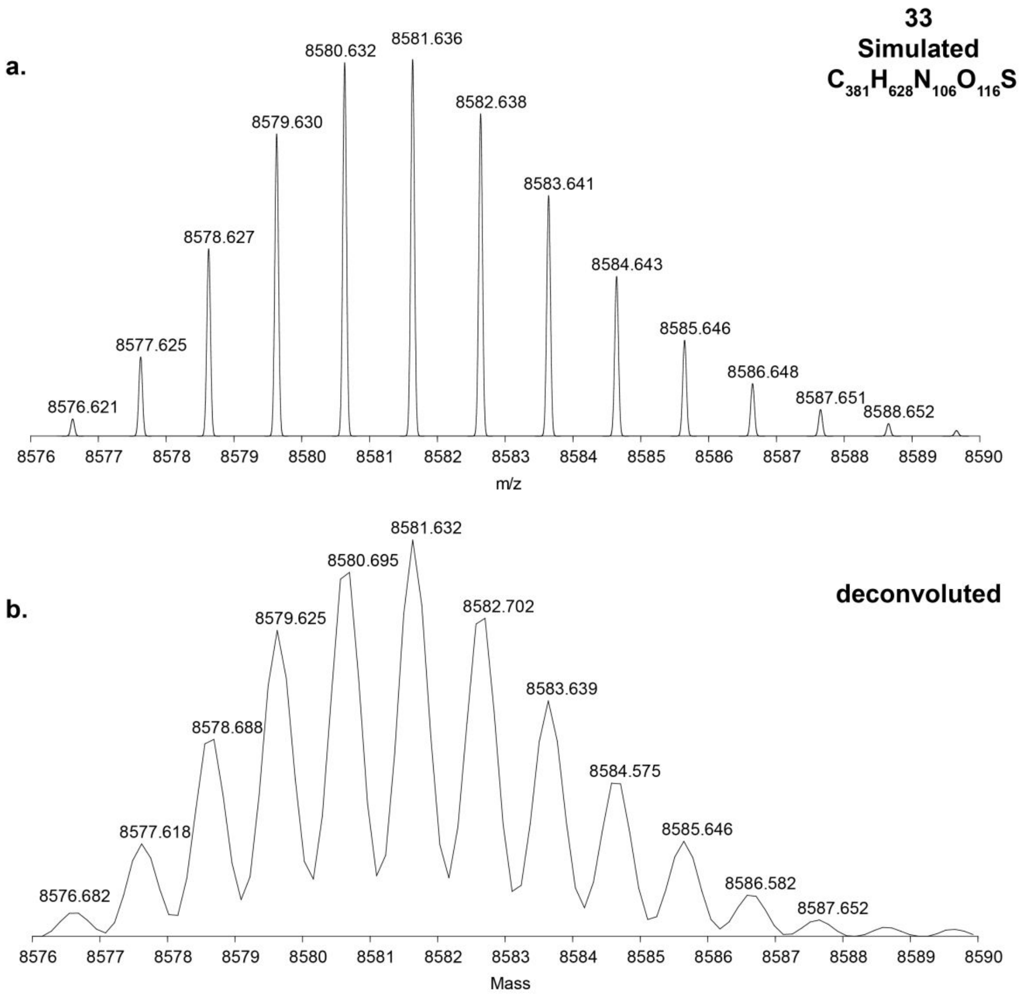

Figure S150. HR-MS analysis of serotoninated-Ub(2-76)(Q2C), **33**. **a**. The simulated HR-MS of reduced form of serotoninated-Ub(2-76)(Q2C), **33**, with chemical formula $C_{381}H_{628}N_{106}O_{116}S$ is shown; **b**. The deconvoluted HR-MS of **33**.

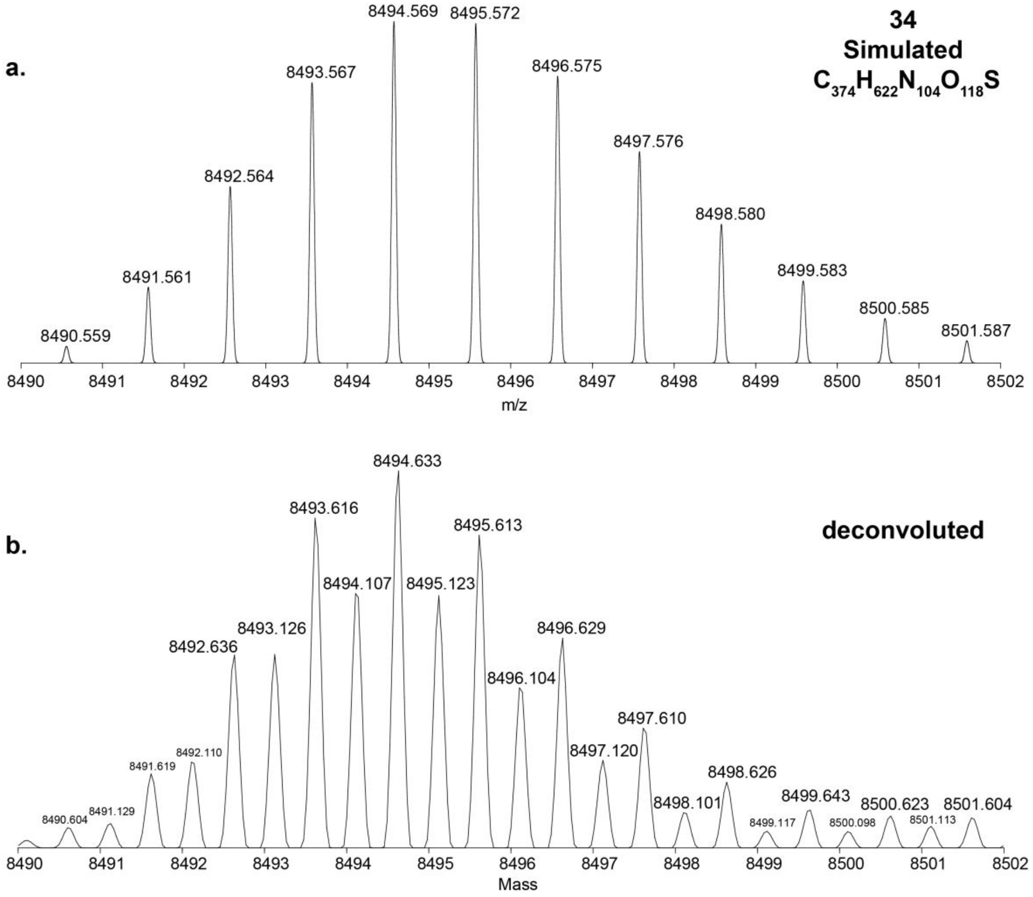

Figure S151. HR-MS analysis of expressed Ub(2-76)(Q2C, M1S), **34**. **a**. The simulated HR-MS of reduced form of expressed Ub(2-76)(Q2C, M1S), **34**, with chemical formula $C_{374}H_{622}N_{104}O_{118}S$ is shown; **b**. The deconvoluted HR-MS of **34**.

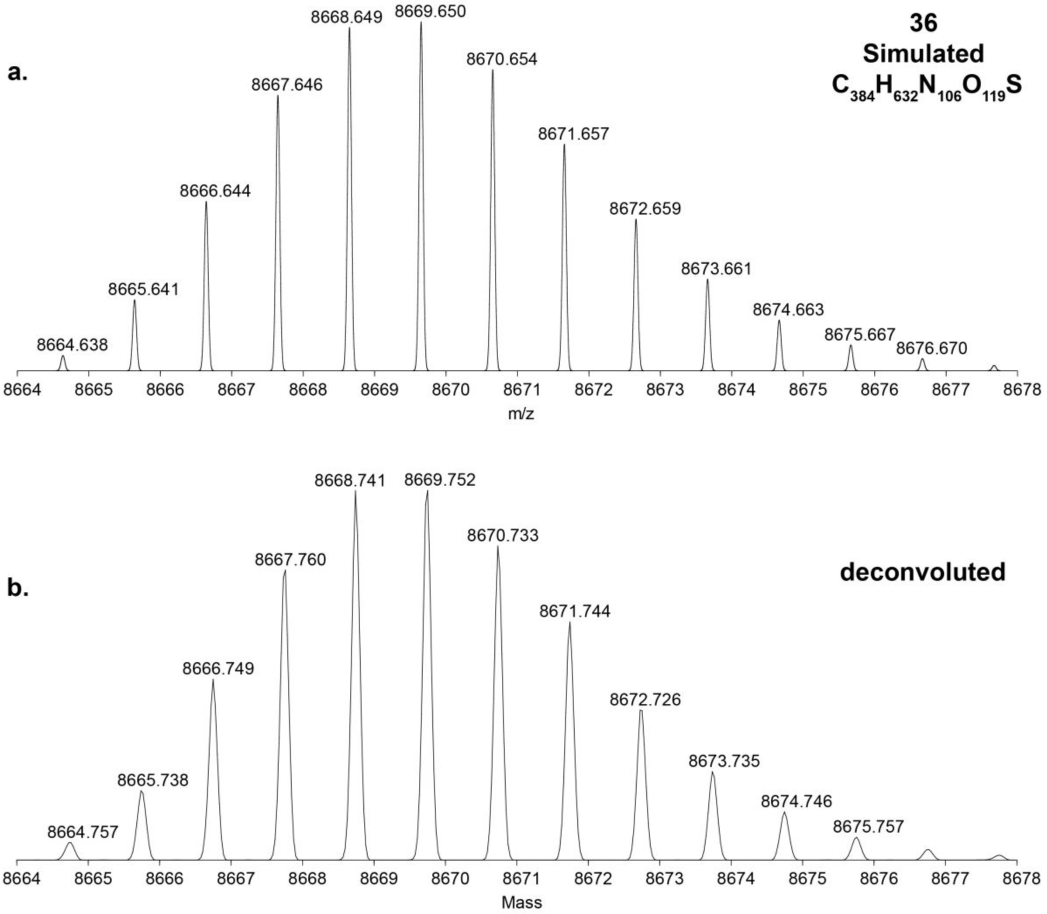

Figure S152. HR-MS analysis of serotoninated-Ub(2-76)(Q2C, M1S), **36**. **a**. The simulated HR-MS of reduced form of expressed serotoninated-Ub(2-76)(Q2C, M1S), **36**, with chemical formula $C_{384}H_{632}N_{106}O_{119}S$ is shown; **b**. The deconvoluted HR-MS of **36**.

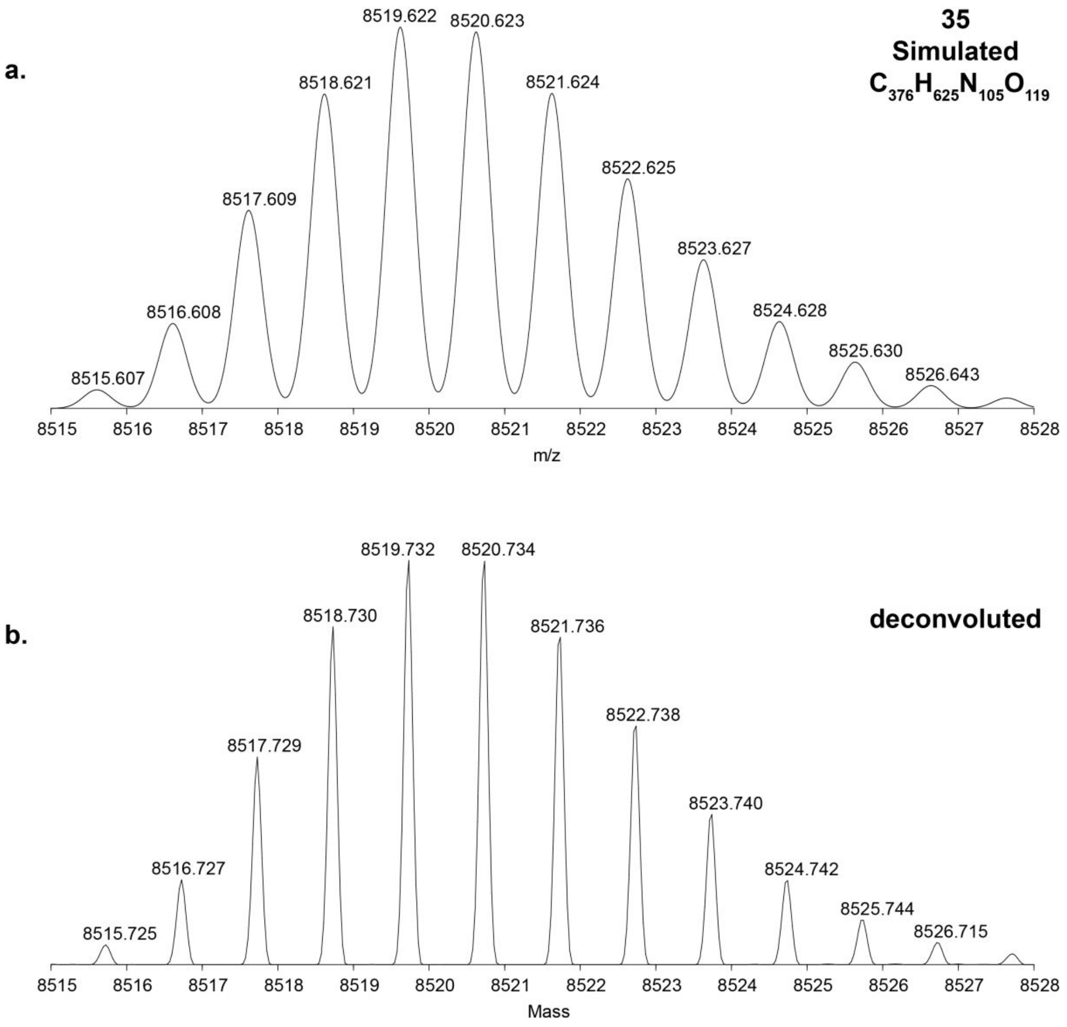

Figure S153. HR-MS analysis of expressed Ub(1-76)(M1S) WT, **35**. **a**. The simulated HR-MS of reduced form of expressed expressed Ub(1-76)(M1S) WT, **35**, with chemical formula $C_{384}H_{632}N_{106}O_{119}S$ is shown; **b**. The deconvoluted HR-MS of **35**.

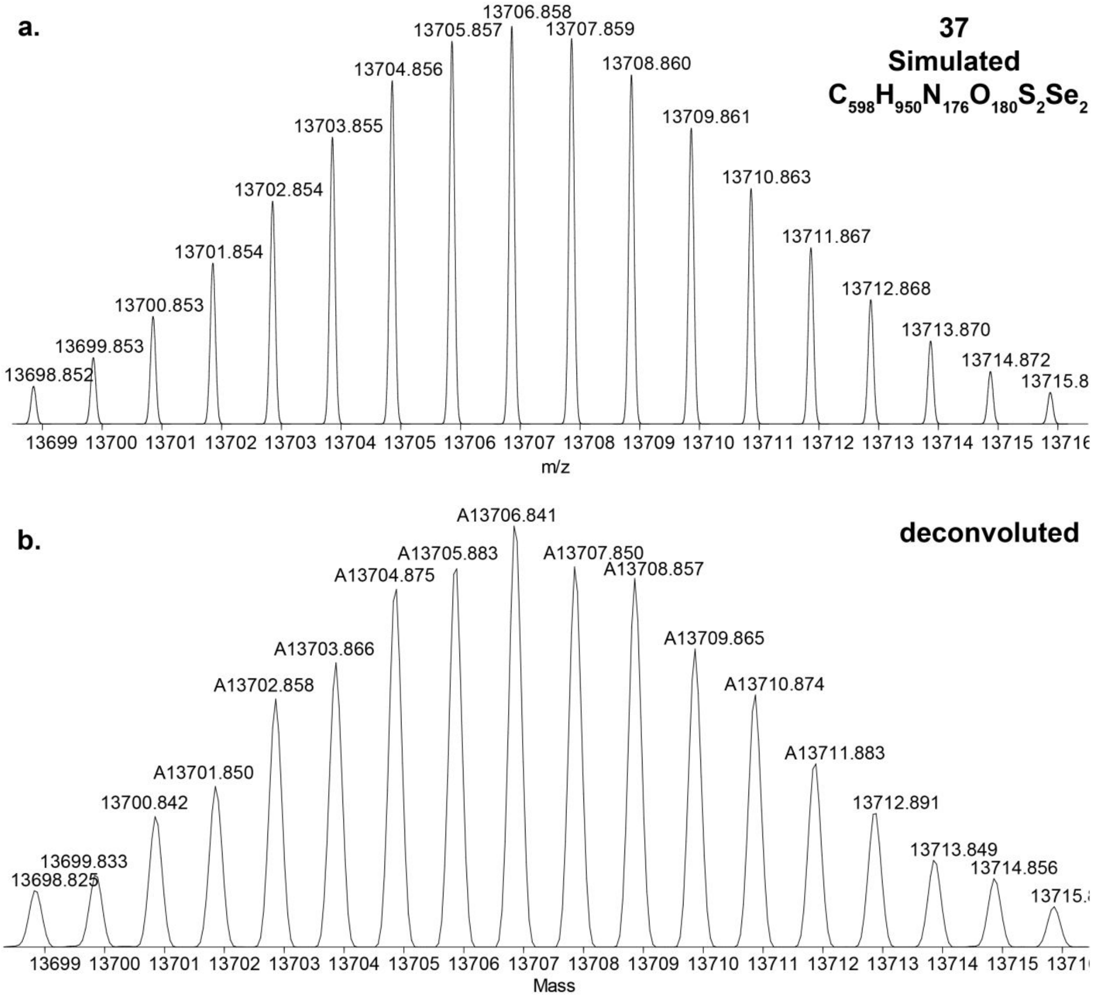

Figure S154. HR-MS analysis of Sec-ZHER2 affibody (dimer), **37**. **a**. The simulated HR-MS of reduced form of Sec-ZHER2 affibody, **37**, with chemical formula $C_{598}H_{950}N_{176}O_{180}S_2Se_2$ is shown; **b**. The deconvoluted HR-MS of **37**.

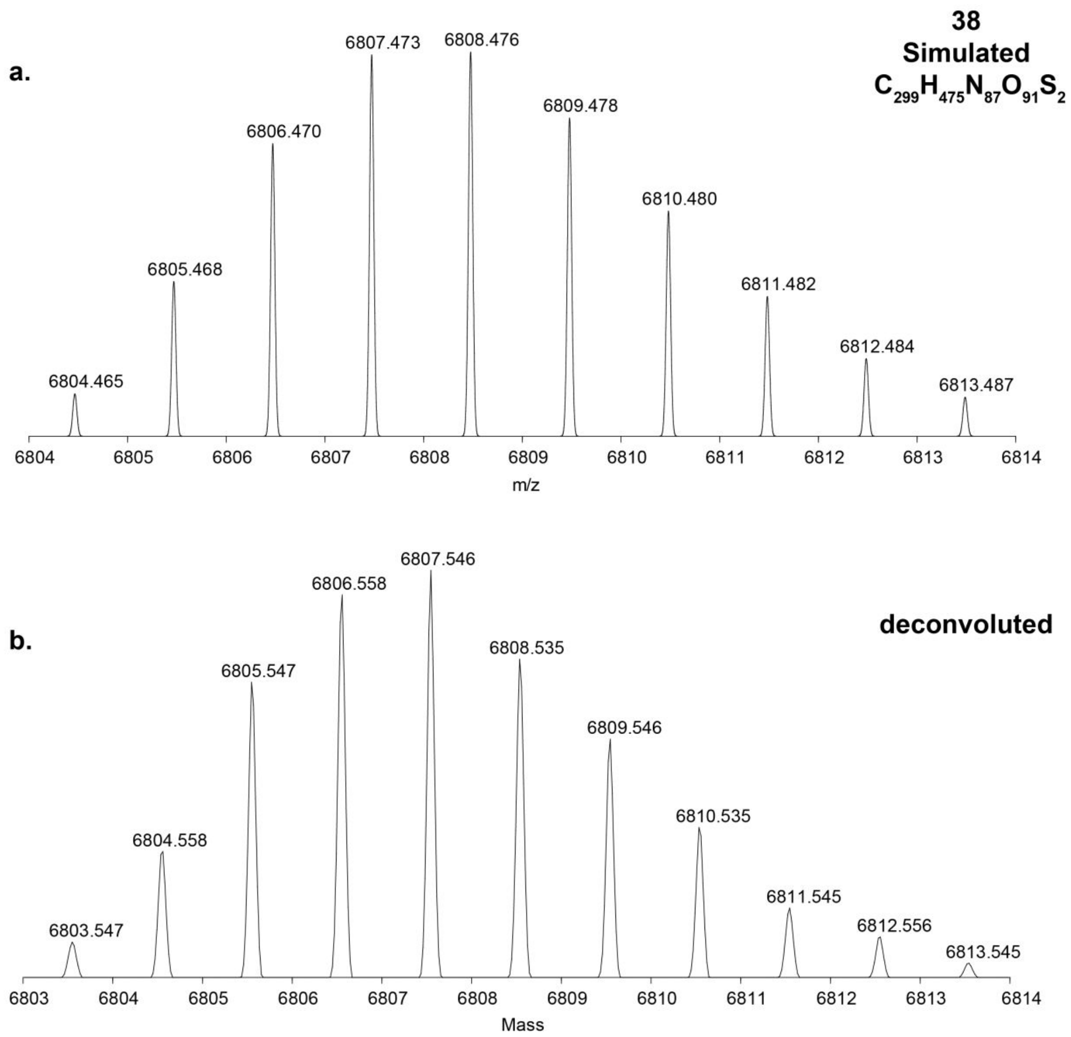

Figure S155. HR-MS analysis of Cys-ZHER2 affibody, **38**. **a**. The simulated HR-MS of reduced form of Cys-ZHER2 affibody, **38**, with chemical formula $C_{299}H_{475}N_{87}O_{91}S_2$ is shown; **b**. The deconvoluted HR-MS of **38**.

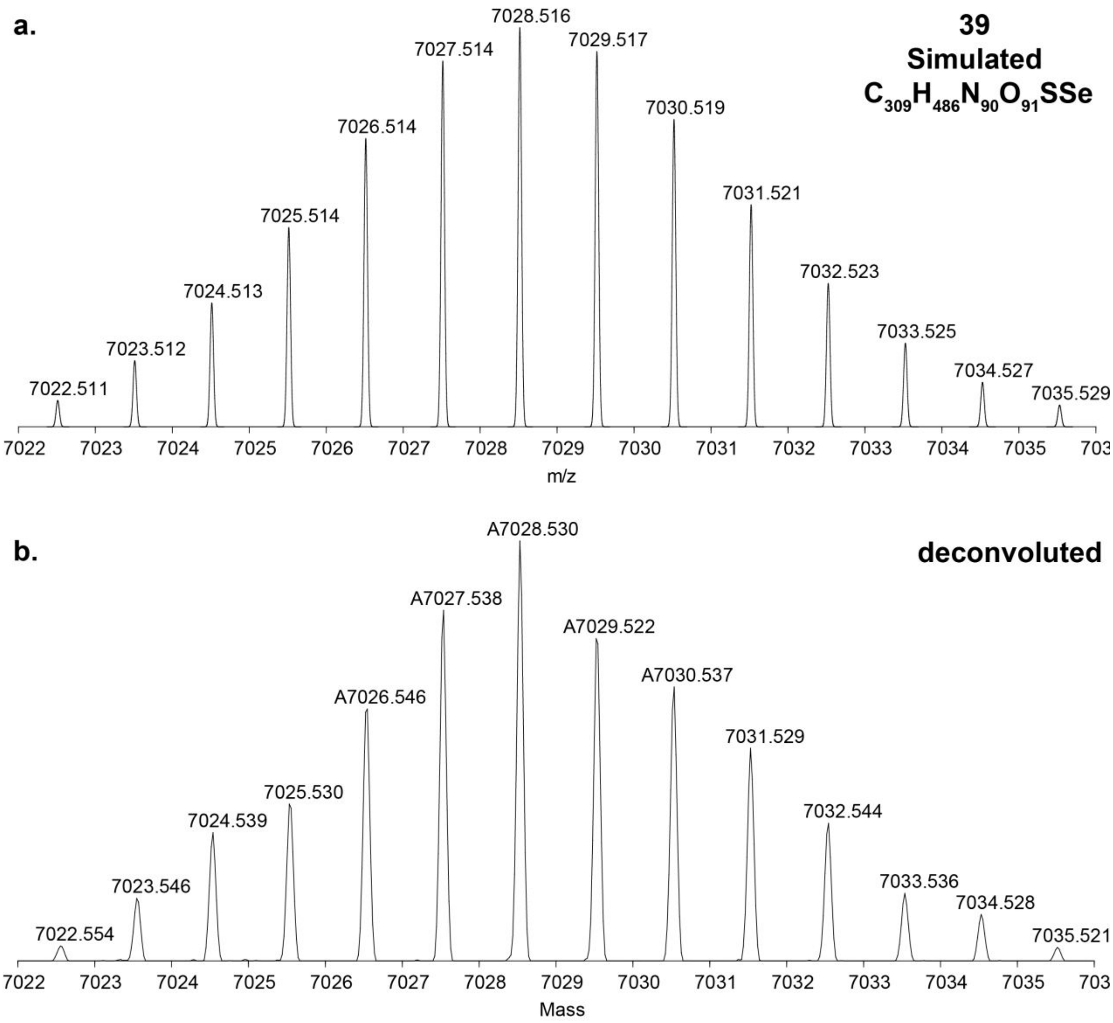

Figure S156. HR-MS analysis of serotoninated-Sec-ZHER2 affibody, **39**. **a**. The simulated HR-MS of reduced form of serotoninated-Sec-ZHER2 affibody, **39**, with chemical formula $C_{309}H_{486}N_{90}O_{91}SSe$ is shown; **b**. The deconvoluted HR-MS of **39**.

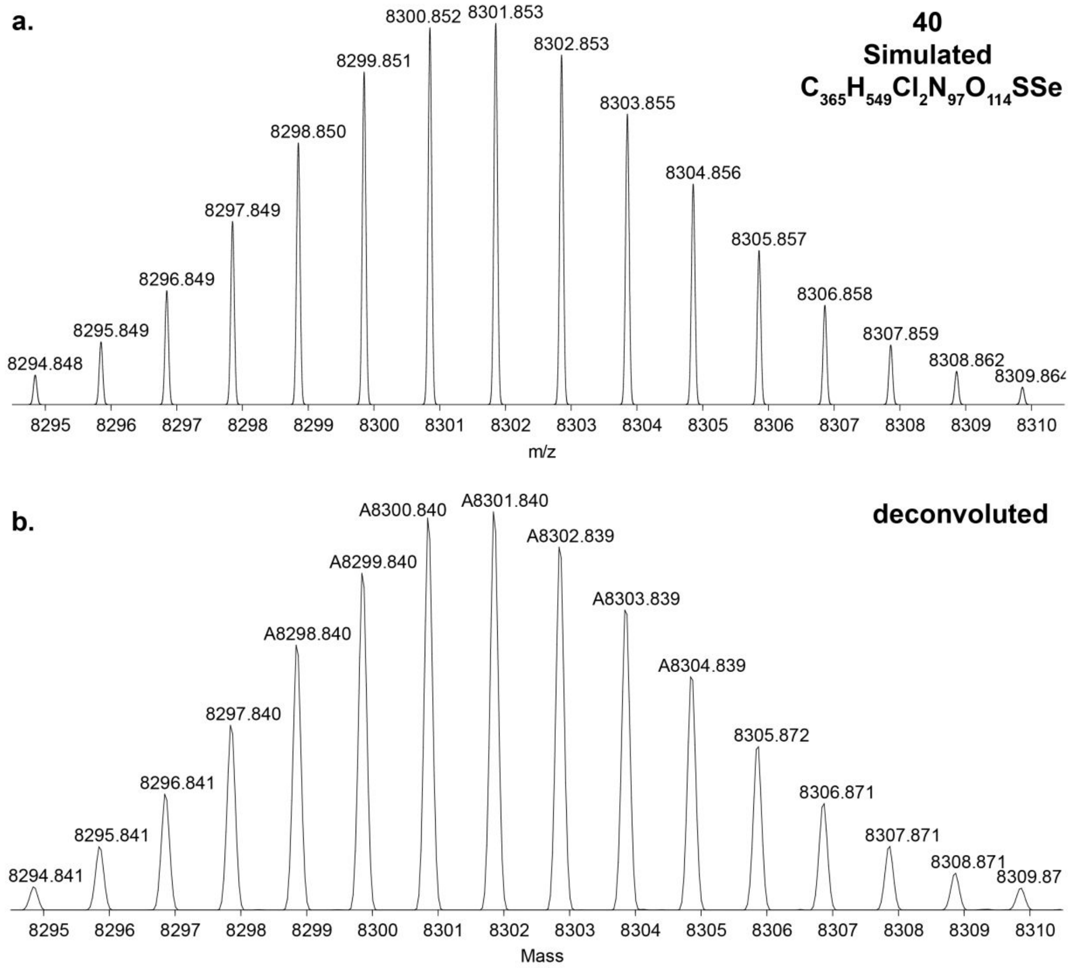

Figure S157. HR-MS analysis of vancomycinated-Sec-ZHER2 affibody, **40**. **a**. The simulated HR-MS of reduced form of vancomycinated-Sec-ZHER2 affibody, **40**, with chemical formula $C_{365}H_{549}Cl_2N_{97}O_{114}SSe$ is shown; **b**. The deconvoluted HR-MS of **40**.

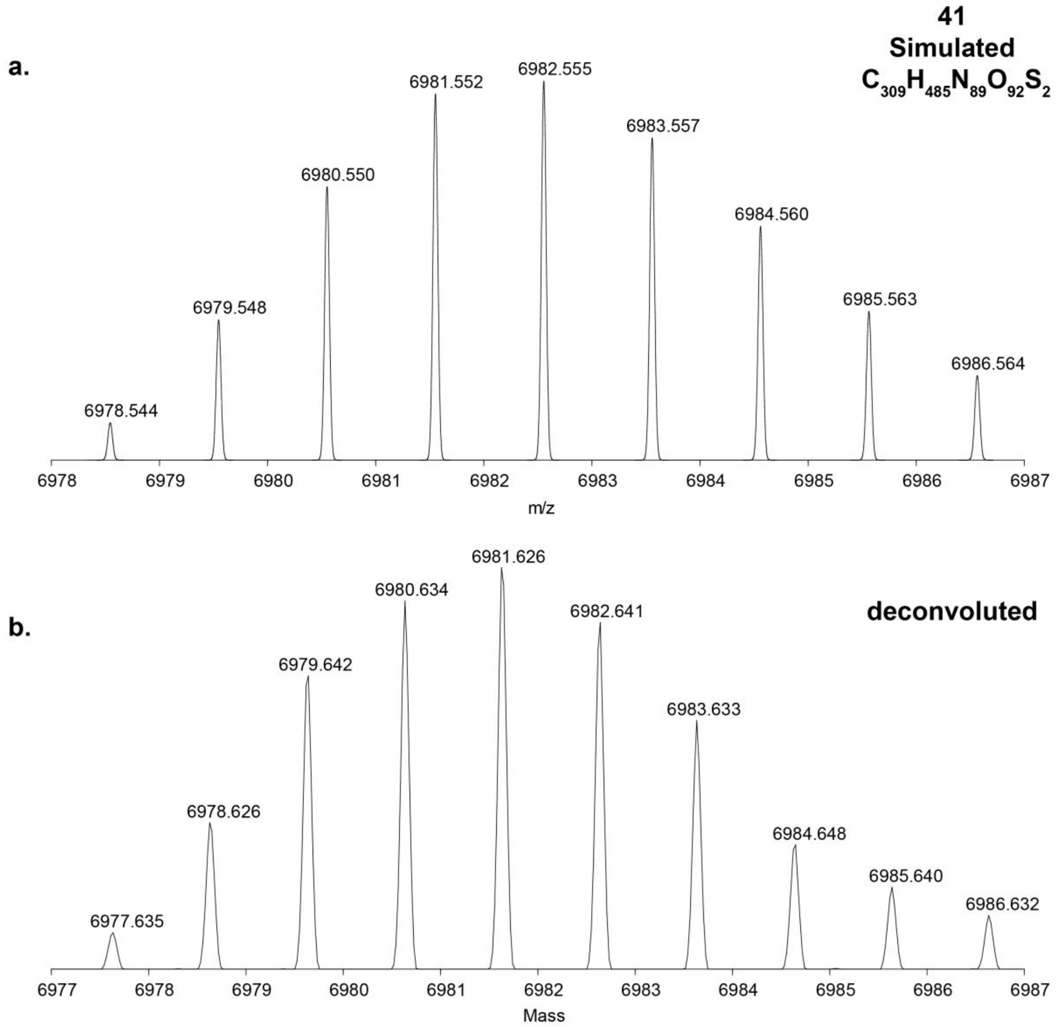

Figure S158. HR-MS analysis of serotoninated-Cys-ZHER2 affibody, **41**. **a**. The simulated HR-MS of reduced form of serotoninated-Cys-ZHER2 affibody, **41**, with chemical formula $C_{309}H_{485}N_{89}O_{92}S_2$ is shown; **b**. The deconvoluted HR-MS of **41**.

References

(1) Pearce, C. M.; Williams, D. H. Complete assignment of the 13C NMR spectrum of vancomycin. *Journal of The Chemical Society-perkin Transactions 1* **1995**, 153-157.

(2) Gieselman, M. D.; Xie, L.; van der Donk, W. A. Synthesis of a Selenocysteine-Containing Peptide by Native Chemical Ligation. *Org. Lett.* **2001**, *3* (9), 1331-1334. DOI: 10.1021/ol015712o.

(3) Zheng, J.-S.; Tang, S.; Qi, Y.-K.; Wang, Z.-P.; Liu, L. Chemical synthesis of proteins using peptide hydrazides as thioester surrogates. *Nat. Protoc.* **2013**, *8* (12), 2483-2495. DOI: 10.1038/nprot.2013.152.

(4) Flood, D. T.; Hintzen, J. C. J.; Bird, M. J.; Cistrone, P. A.; Chen, J. S.; Dawson, P. E. Leveraging the Knorr Pyrazole Synthesis for the Facile Generation of Thioester Surrogates for use in Native Chemical Ligation. *Angewandte Chemie International Edition* **2018**, *57* (36), 11634-11639. DOI: https://doi.org/10.1002/anie.201805191.

(5) Stoll, S.; Schweiger, A. EasySpin, a comprehensive software package for spectral simulation and analysis in EPR. *J. Magn. Reson.* **2006**, *178* (1), 42-55. DOI: https://doi.org/10.1016/j.jmr.2005.08.013.

(6) Buettner, G. R. Spin Trapping: ESR parameters of spin adducts 1474 1528V. *Free Radical Biology and Medicine* **1987**, *3* (4), 259-303. DOI: https://doi.org/10.1016/S0891-5849(87)80033-3.

(7) *Gaussian 16 Rev. A.01*; Wallingford, CT, 2016. (accessed.

(8) Becke, A. D. Density-functional thermochemistry. III. The role of exact exchange. *J. Chem. Phys.* **1993**, *98* (7), 5648-5652. DOI: 10.1063/1.464913 (acccessed 3/30/2024). Grimme, S.;

Ehrlich, S.; Goerigk, L. Effect of the damping function in dispersion corrected density functional theory. *J. Comput. Chem.* **2011**, *32* (7), 1456-1465, https://doi.org/10.1002/jcc.21759. DOI: https://doi.org/10.1002/jcc.21759 (acccessed 2023/03/19).

(9) Weigend, F.; Ahlrichs, R. Balanced basis sets of split valence, triple zeta valence and quadruple zeta valence quality for H to Rn: design and assessment of accuracy. *Phys. Chem. Chem. Phys.* **2005**, *7* (18), 3297-3305. DOI: 10.1039/b508541a.

(10) Miertuš, S.; Scrocco, E.; Tomasi, J. Electrostatic interaction of a solute with a continuum. A direct utilizaion of AB initio molecular potentials for the prevision of solvent effects. *Chem. Phys.* **1981**, *55* (1), 117-129. DOI: https://doi.org/10.1016/0301-0104(81)85090-2. Tomasi, J.; Mennucci, B.; Cammi, R. Quantum Mechanical Continuum Solvation Models. *Chem. Rev.* **2005**, *105* (8), 2999-3094. DOI: 10.1021/cr9904009.

(11) Wang, J.-J.; Huang, H.; Sun, H.-L.; Yang, F.; Wen, J.; Zhu, R. Mimicking hydrogen-atom-transfer-like reactivity in copper-catalysed olefin hydrofunctionalization. *Nat. Catal.* **2024**. DOI: 10.1038/s41929-024-01182-9. Ueda, Y.; Masuda, Y.; Iwai, T.; Imaeda, K.; Takeuchi, H.; Ueno, K.; Gao, M.; Hasegawa, J.-y.; Sawamura, M. Photoinduced Copper-Catalyzed Asymmetric Acylation of Allylic Phosphates with Acylsilanes. *J. Am. Chem. Soc.* **2022**, *144* (5), 2218-2224. DOI: 10.1021/jacs.1c11526. Zeng, X.; Yan, W.; Paeth, M.; Zacate, S. B.; Hong, P.-H.; Wang, Y.; Yang, D.; Yang, K.; Yan, T.; Song, C.; et al. Copper-Catalyzed, Chloroamide-Directed Benzylic C–H Difluoromethylation. *J. Am. Chem. Soc.* **2019**, *141* (50), 19941-19949. DOI: 10.1021/jacs.9b11549. Deng, X.; Dang, Y.; Wang, Z.-X.; Wang, X. How Does an Earth-Abundant Copper-Based Catalyst Achieve Anti-Markovnikov Hydrobromination of Alkynes? A DFT Mechanistic Study. *Organometallics* **2016**, *35* (11), 1923-1930. DOI:

10.1021/acs.organomet.6b00246.
